# Exploring the chemical design space of metal–organic frameworks for photocatalysis†

**DOI:** 10.1039/d5sc01100k

**Published:** 2025-05-13

**Authors:** Beatriz Mourino, Sauradeep Majumdar, Xin Jin, Fergus McIlwaine, Joren Van Herck, Andres Ortega-Guerrero, Susana Garcia, Berend Smit

**Affiliations:** a Laboratory of Molecular Simulation (LSMO), Institut des Sciences et Ingénierie Chimiques, Valais Ecole Polytechnique Fédérale de Lausanne (EPFL) Rue de l’Industrie 17 CH-1951 Sion Valais Switzerland berend.smit@epfl.ch; b The Research Centre for Carbon Solutions (RCCS), School of Engineering and Physical Sciences, Heriot-Watt University EH144AS Edinburgh UK; c Nanotech@Surfaces Laboratory, Empa - Swiss Federal Laboratories for Materials Science and Technology 8600 Dübendorf Switzerland

## Abstract

In this work, we introduce a combined DFT and machine learning approach to obtain insights into the chemical design of metal–organic framework (MOF) photocatalysts for hydrogen (HER) and oxygen (OER) evolution reactions. To train our machine learning models, we evaluated a dataset of 314 MOFs using a dedicated DFT workflow that computes a set of five descriptors for both closed and open shell MOFs. Our dataset is composed of a diverse selection of the QMOF database and experimentally reported MOF photocatalysts. In addition, to ensure a balanced dataset, we designed a set of MOFs (CDP–MOF) inspired by insights obtained regarding different types of photocatalytic materials. Our machine-learning approach allowed us to screen the entire QMOF and CDP–MOF databases for promising candidates. Our analysis of the chemical design space shows that we have many materials with a suitable spatial overlap of electron and hole, band gap, band-edge alignment to HER, and charge-carrier effective masses. However, we have identified in the QMOF database only a very small percentage of materials that also have the right band-edge alignment to OER. With the CDP–MOF database, we successfully targeted building blocks that potentially have the correct OER band alignment, and indeed obtained a larger percentage of materials that obey these criteria. Among those, a few motifs stood out, such as Au-pyrazolate, Ti clusters and rod-shaped metal nodes, and a particular MOF designed with the Mn_4_Ca cluster, which mimics the OER center in the photosystem II of photosynthesis.

## Abstract 2

In this work, we introduce a combined DFT and machine learning approach to obtain insights into the chemical design of metal–organic framework (MOF) photocatalysts for hydrogen (HER) and oxygen (OER) evolution reactions.

## Graphical abstract

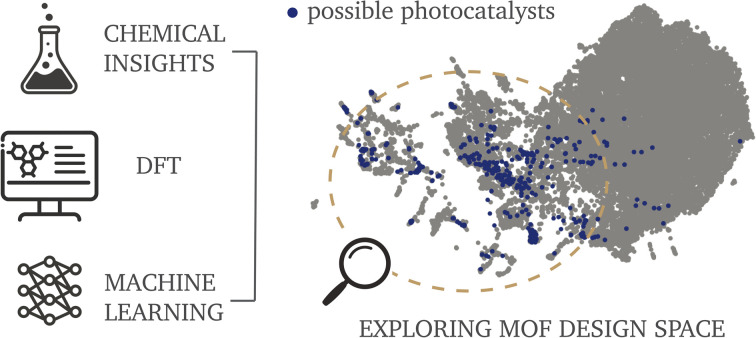

## Introduction

Photocatalysis-based green energy is a promising alternative towards sustainable solutions.^1–4^ A photocatalytic process involves the excitation of materials under light radiation, ideally generating free charge carriers that can engage in surface reactions. All steps are closely tied to the chemistry and optoelectronic properties of the materials.^5,6^ The intriguing optoelectronic properties of metal–organic frameworks (MOFs) and their tunability make these materials attractive photocatalysts.^6–8^ The building-block nature of MOFs gives rise to countless design possibilities, culminating in a vast chemical design space.^9,10^

When searching for an optimal photocatalyst, we can explore this design space by relying on chemical insights.^11–13^ This translates to constructing MOFs, experimentally or *in silico* based on identified building blocks that could contribute to enhanced photocatalytic properties.^14,15^ While insightful, this approach is time-consuming and thus impractical on a larger scale.

Alternatively, one can rely on computer simulations to expand the search for promising materials.^16–18^ In particular, first-principles methods provide an accurate way of assessing photocatalytic properties.^19–21^ However, the required calculations are resource-consuming and often prohibitive.^6,20^ This limits our ability to carry out high-throughput screening and consequently hinders the exploration of the MOF design space in the context of photocatalysis.

By offering a cost-effective solution, machine-learning approaches could aid such exploration,^22,23^ but they come with their challenges. Machine-learning models are typically successful when trained on large, well-balanced, and diverse datasets,^24^ which are difficult to obtain for photocatalysis.^6^ Indeed, good MOF photocatalysts are scarce; we carried out some preliminary calculations on structures selected from the QMOF database,^25^ and our success rate was very low. Hence, even if we were to compute the photocatalytic properties of many MOFs, we hypothesize that the resulting dataset would likely be imbalanced and lacking diversity due to the current focus of existing MOF databases on gas storage and separation.

In this work, we developed a threefold strategy that synergistically combines chemical insights, first-principles calculations, and machine learning. We used chemical insights to generate *in silico* MOFs that are aimed at filling gaps in the chemical design space in areas relevant to photocatalysis. These materials contributed to an increase in the diversity metrics of linker and metal node chemistry. We then calculated DFT-based photocatalytic descriptors of a balanced dataset comprising our generated MOFs, MOFs from the QMOF database, and experimental MOF photocatalysts.^6,20,26^ With this dataset, we were able to fine-tune pre-trained machine-learning methods—MOFTransformer,^27^ and GPT-J^28^—that reduced the cost of our exploration by predicting binary outcomes of each photocatalytic descriptor on a much larger number of MOFs. We focus on MOFs for overall water splitting, often referred to as the “Holy Grail” in the energy landscape.^29,30^ Throughout this process, our aim was to gain insight into the structure–property relationship and identify trends in the MOF design space.

## Evaluation of MOFs for photocatalysis

### Overview on photocatalytic descriptors

Ideally, a viable MOF photocatalyst offers separated charge carriers that live long enough to promote the desired photoredox reactions upon light absorption in the visible region. Long-living charge carriers can be achieved through high electron and hole mobilities and/or spatial electron–hole separation, often facilitated by low-lying charge-transfer excitations. To drive a photoredox reaction thermodynamically, a MOF photocatalyst should have its ionization potential and electron affinity properly aligned with the redox potentials of such a reaction. Focusing on the case study of water splitting, we have translated these observations into six descriptors that we can compute to evaluate our MOFs for photocatalysis: charge carrier mobility, charge separation, charge-transfer character, visible light absorption, and band alignment to the hydrogen (HER) and oxygen (OER) evolution reactions.

As a proxy for computationally demanding *charge carrier mobility* calculations, we compute the charge carrier effective masses (m*). Low effective masses are usually associated with higher charge carrier mobilities. m* is determined based on the curvature of the valence and conduction band edges.^20^

To assess *charge separation*, we computed the weighted average of the spatial overlap (*Λ*) between ground-state unrestricted Kohn–Sham (UKS) DFT calculations for charged doublets, that is, −1 for electron injection and +1 for hole injection.^20^ This descriptor is computed at or empirically adjusted to PBE0-TC-LRC^31,32^ (coulomb-truncated hybrid with long-range correction) DFT calculations.

Likewise, we evaluated the *charge transfer character* (in particular, linker-to-metal node or LMCT) by computing the weighted average of the spatial overlap constrained to atoms in the linker and separately in the metal node.

Visible light absorption and band alignment to the photoredox reactions comprise the three *energy-based descriptors*, which are computed at or empirically adjusted to PBE0-TC-LRC^31,32^ (coulomb-truncated hybrid with long-range correction) DFT calculations. The thermodynamic feasibility of a material to drive *HER* and *OER* reactions is evaluated by means of vacuum level alignment.

Lastly, a MOF is said to absorb visible light if the optical gap is within the range of 1.6 eV to 3.2 eV. We used PBE0-TC-LRC^31,32^ hybrid functional-level Kohn–Sham gaps (*E*_BG_, empirically adjusted or directly computed) to assess *visible light absorption*. A correct assessment of visible light absorption should be done by computing the optical gaps, which are only obtained with appropriate excited-state methods such as time-dependent DFT or GW/BSE. However, performing such calculations on our dataset would be unfeasible due to the associated high computational cost. We note that, in general, the experimental optical gap values of MOFs often lie somewhere between PBE (Perdew–Burke–Ernzerhof semi-local functional^33^) and PBE0 values (see Table S2 of Fumanal *et al.*^20^). With that in mind, and aiming for consistency among our energy-based descriptors, we choose PBE0 values as our reference to assess visible light absorption. PBE0 shows improved treatment of localized electronic states when compared to PBE, which is crucial for the other energy-based descriptors of MOFs, where localization of d orbitals lead to artifacts in the PBE results. We highlight that this is a first screening level, and further excited-state calculations should be done to correctly compute optical gaps for the promising shortlisted MOFs. More details can be found in the ESI.†

### Database design and evaluation

Initial tests on the QMOF database^25^ showed that finding a MOF that would pass most of the descriptors to be promising is very low (less than 10% on a diverse subset of 154 MOFs, see ESI†). Therefore, we developed an alternative approach, using our knowledge (and intuition) to build potential MOF photocatalysts. We refer to our database as CDP–MOF, where CDP stands for Chemical insights-based Diversity-driven Photocatalyst.

#### Design criteria

In what follows, we illustrate our rationale for identifying promising and diverse building blocks that could enhance one or more photocatalytic properties.

##### Light absorption

Ideally, for solar-derived alternative energies, it is desired that photocatalysts absorb visible light and, therefore, have an optical gap within the visible range.

Therefore, the first selection criterion is to select building blocks, especially linkers, that are known to absorb visible light. A logical choice is linkers composed of known chromophores with conjugated π-systems (*e.g.*, porphyrin and pyrene). Selected linkers thus contain, for example, porphyrin (ol50, see Fig. S14†), and thiazole (ol64, see Fig. S15†). Both are expected to be active in the visible range due to highly conjugated π orbitals and high electronic density.^34,35^ Among others, we included pyrazolate-, triazolate-, and thiolate-based metal-linker bond chemistry to generate structures going beyond the conventionally used carboxylate-based chemistry in literature.

##### Alignment to HER and OER

In addition to having an optical gap in the visible range, the electron removal and addition energies should be aligned to the redox potentials of the desired reactions. This alignment ensures that the process can happen thermodynamically.

Therefore, we chose building blocks that are known to properly align with the case study of oxygen (OER) and hydrogen (HER) evolution reactions. At pH 0, the redox potentials of HER and OER are, respectively, −4.4 eV and −5.63 eV w.r.t. vacuum.^36^ For example, we have Ti clusters that individually tend to align well with HER.^37,38^ MOFs are a good platform to tune the band gap of Ti-based clusters using building block selection,^39,40^ which we explore in this work. For OER, as an example, we selected an artificial cluster (mn39, with Mn(iii)/Mn(iv)) mimicking the OER center in chlorophyll. This cluster is associated with the pivotal 4-electron transfer step responsible for generating O_2_ during photosynthesis.^41,42^

##### Charge separation and charge-transfer character

Another aspect to look at is the choice of building blocks that could promote more efficient charge separation. This is important to reduce the possibility of charge recombination after excitation. In this regard, the nature of MOFs is particularly interesting because they can display metal node-to-linker or linker-to-metal node charge transfer. A necessary condition for the latter is the presence of low-lying metal states in the band structure.^20^ We chose Mn, Co, and Fe-based clusters with an open shell character that could contribute to a charge-transfer mechanism. Moreover, we designed MOFs with one-dimensional (1D) SBUs (rod-shaped, or rod MOFs) that can contribute to the effective separation of excited electron and hole.^43^ Rod MOFs are highlighted in Table S1† and represented in Fig. S4 and S5.†

##### Charge transport

Lastly, in an ideal photocatalytic process, it is beneficial to have mobile charge carriers, which could help prevent charge recombination and promote faster migration to the catalytic active sites.^20,44^

Generally, in a band-like transport with highly dispersive bands, charge carrier mobilities are higher at lower temperatures than hopping mechanisms.^45^ However, in most MOFs, hopping mechanisms are predominant.^20^ We can take inspiration from conductive MOFs to design more mobile charge carriers. MOFs with high conductivity often display enhanced charge delocalization and/or continuous charge transport pathways.^46^ The latter can be tuned by choosing linkers that tend to form π–π stacking, whereas the former can be achieved by selecting softer, more electropositive linkers and/or continuous SBUs (such as 1D metal nodes) where metals and ligands have matching energy levels and good orbital overlap.^46^ This is often a trade-off with charge separation. As an example, linkers containing thiophene groups and N atoms coordinating the metals were chosen (ol31, see Fig. S11†).

#### Generation of structures

To ensure that structures are generated correctly, we followed a protocol that uses the metal node as the starting point. Whenever we proposed a new metal node to construct the MOFs with, we manually inspected the possible oxidation states of the metals and charges of the metal node. We looked for the same information in previous experimental works employing the metal node in question, including the original article linked in the CSD database from which the node was taken. Then, from our previously assembled linkers, we selected, for each metal node, the ones that would be suitable when considering charge neutrality and connectivity. We used oximachine^47^ and an in-house tool to corroborate our predictions regarding oxidation state and MOF sanity. We manually flagged the problematic structures and double-checked them.

**Fig. 1:**
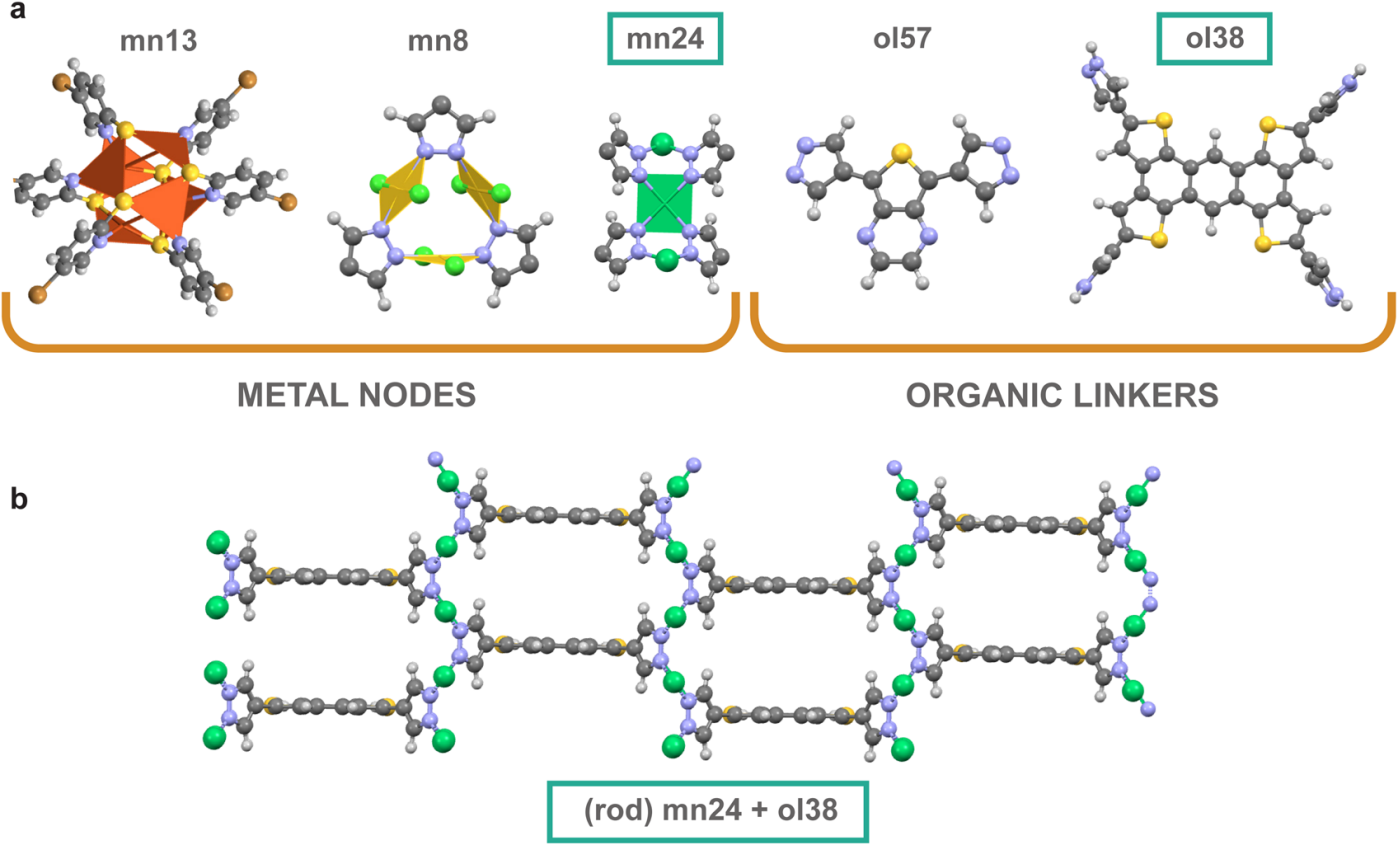
(a) Structures of some of the diverse metal nodes (mn) and organic linkers (ol) utilized in this work, (b) along with an example of a MOF designed with a 1D Ni(ii) metal node (mn24) and an anthratetrathiophene-containing linker (ol38), highlighting the 1D-rod-shaped network connectivity.

Fig. 1a shows examples of metal nodes and organic linkers utilized to generate MOFs. These representative building blocks target diverse chemistry and possible enhancement in photocatalytic properties: Cu(i), Au(iii), rod-shaped Ni(ii) cluster, and pyrazolate and thiol groups as the linker-metal node bond chemistry. The full list of metal nodes, organic linkers, and topologies used to design CDP–MOF is provided in the ESI (Fig. S2 to S12, Tables S1 and S3†). Further details about the structure generation process and diversity analysis are also provided in the ESI section.†

The full space of CDP–MOF comprises around 1000 structures. We grouped them by metal node and chose the smallest MOFs per metal node group to comprise the subset of 146 structures for evaluation at the DFT level.

### DFT calculations

We adapted a workflow originally designed for covalent organic frameworks (COFs) to assess the potential of the generated MOFs as prospective photocatalysts.^26^ This workflow calculates six density-functional theory (DFT)-based photocatalytic descriptors, as outlined previously. The challenge for MOFs, if compared to COFs, arises from the theoretical complexity that the presence of the metal can introduce, namely, the possibility of having unpaired electrons in open-shell metals and the fact that the delocalization error in DFT affects metals differently.

For closed-shell structures, all calculations were kept at the level of a generalized gradient approximation (GGA) functional (PBE^33^), and the energy-based and charge separation descriptors were empirically adjusted to reproduce more accurate hybrid functional (PBE0-TC-LRC,^31,32^ a coulomb-truncated hybrid with long-range correction) values. Such an empirical adjustment was firstly developed for a set of similar MOFs containing mostly Zn, based on the systematic employment of 25% of Hartree–Fock exact exchange in PBE0 functionals.^6,48,49^ To verify the validity of this approach, we have selected one MOF for each metal node to be evaluated at a higher level of theory (with a hybrid functional, PBE0). The full list of all evaluated metal nodes is highlighted in Table S1.†

Fig. S23† shows that the empirical correlation between PBE (lower level of theory) and the hybrid PBE0 values previously evaluated for a set of Zn-based MOFs^6^ can be extended to the closed-shell structures that are evaluated in this database. The correlation for ionization potential (IP), electron affinity (EA), and band gap energies (*R*^2^ rounded values of 0.85, 0.92, and 0.94, respectively) remains relatively high when compared to literature values (*R*^2^ rounded values of 0.92, 0.98, and 0.93, respectively).^6^ The high correlations that persist upon the inclusion of our structures allow us to compute the energy-based descriptors at a lower level of theory (GGA functional, PBE) for closed-shell systems and later adjust empirically to more accurate PBE0 values.

For open-shell systems with partially occupied d orbitals, however, the transferability of the workflow required the energy-based descriptors to be computed directly at the PBE0 level to avoid inaccurate capturing of the electronic properties, *e.g.*, self-interaction error and failure to detect a band gap.^19,25^ Indeed, Rosen *et al.*^25^ shows that, for open-shell MOFs, the distribution of PBE gaps is shifted to values very close to 0, which is corrected when adding some amount of HF exchange. Further calculations for open-shell MOFs, including the band structure to compute effective masses, are performed with a GGA-based DFT + Hubbard functional (see details in the ESI and Table S4† for *U* values).

### Machine learning

The DFT calculations we need to perform to identify whether a MOF is a promising photocatalyst are relatively expensive. We evaluated 314 materials comprising CDP–MOFs (146), QMOFs (a diverse subset of 154 structures, see SI for selection procedure), and reported experimental structures (14, see ESI†^20,50^) within a reasonable computational budget. Conventional machine-learning approaches would have difficulty making reliable predictions with such a low number of training data.

In this work, we show that we can leverage the MOF-transformer model of Kang *et al.*^27^ and large language model (LLM) using the approach of Jablonka *et al.*,^28^ to obtain surprisingly accurate predictions using a small amount of data. Both approaches used a subset of the evaluated structures as a test set.

MOFTransformer is a multi-modal Transformer model pre-trained on 1 million MOFs. It uses atom-based graphs and energy-grid embeddings to capture local and global features respectively. This model can be fine-tuned with smaller datasets to predict a wide range of properties, *e.g.*, gas adsorption, diffusion, and electronic properties.^27^ Likewise, LLMs have recently emerged as a promising alternative to predict various properties with a small training dataset through prompting.^28,51^

For more details on both approaches, we refer the reader to the ESI† and the original publications.^27,28^

#### MOFTransformer

For the MOFTransformer, we fine-tuned the base model developed by Kang *et al.*^27^ to predict the photocatalytic descriptors in a binary classification fashion.

The MOFTransformer uses an energy-grid embedding (using CH_4_ as a probe of which the energy is computed at each grid point) and an atom-based graph embedding to represent a MOF. The idea behind the MOFTranformer is to leverage its pretraining on a large data set of MOFs.

We used 20% of the data as a holdout test set for model evaluation. 16% of the data was used as a validation set to initiate early stopping. A maximum of 10 epochs was used, with a batch size of 8.

#### Large language model (LLM)

To fine-tune an LLM (GPT-J), we used the framework developed by Jablonka *et al.*^28^ as a starting point. The LLM models obtained from fine-tuning of GPT-J can compete with many state-of-the-art models.^51^ Its simplicity in representing MOFs *via* text strings, such as SMILES or chemical formulas, makes it an attractive alternative to field-specific features.

We used training prompts in the format “What is the <property> of <presentation of chemical structure>?” and their respective answers were used to fine-tune the base model. Thereafter, prompting similar questions for unseen examples gave chemically relevant predictions of the structure's property.

In this work, we used the MOFid^52^ as a chemical description of the structures. The MOFid is a string comprising the chemical composition of the metal node, organic linker, and the topology of the structure. The reported average metrics were taken over seven experiments. The number of epochs and learning rate were set to 25 and 0.0003, respectively, for all runs.

### Chemical/feature space analysis

In this work, we also use our machine-learning model to analyze which part of the chemical design space we can find the most promising materials.

For this, we define a contextualized feature space using the MOFTransformer. The underlying idea is that the MOFTransformer gives us the attention score for a specific prediction task. This attention score, combined with the vector representation of the MOF, allows us to define a similarity metric in which MOFs with similar performance are separated by a relatively short distance in feature space.

The process of generating the contextualized feature space is as follows. We assume our MOFTransformer is fine-tuned on a target using a small training dataset.

Firstly, each MOF in the QMOF and CDP databases (total of ≈ 21 000 structures) is featurized, and a forward pass of the model is used. Then, each forward pass gives us the contextual embeddings and the predicted target. The contextual embeddings (a vector with 768 dimensions for each MOF) are reduced to 2 dimensions using UMAP. This process is then repeated for each target.

The UMAP representation allows us to visualize where MOFs with similar performance are located in the feature space. The exact process can be done without fine-tuning, which will return the embeddings of a MOF that are not contextualized on any target and represent a general representation of the MOF learned during pre-training. We call these embeddings the general feature space.

## Results and discussion

As an application of our approach, we focus on overall water splitting (OWS, or simultaneous HER and OER).

### DFT evaluation

We calculated the DFT descriptors for a total of 314 structures from CDP–MOF database, QMOF database (see ESI† for selection procedure), and experimentally reported photocatalysts. An overview of the distribution of all the computed descriptors for both closed (empirically adjusted to PBE0 values) and open (computed at PBE0 level) shell MOFs can be seen in Fig. S24 of the ESI.†

**Fig. 2:**
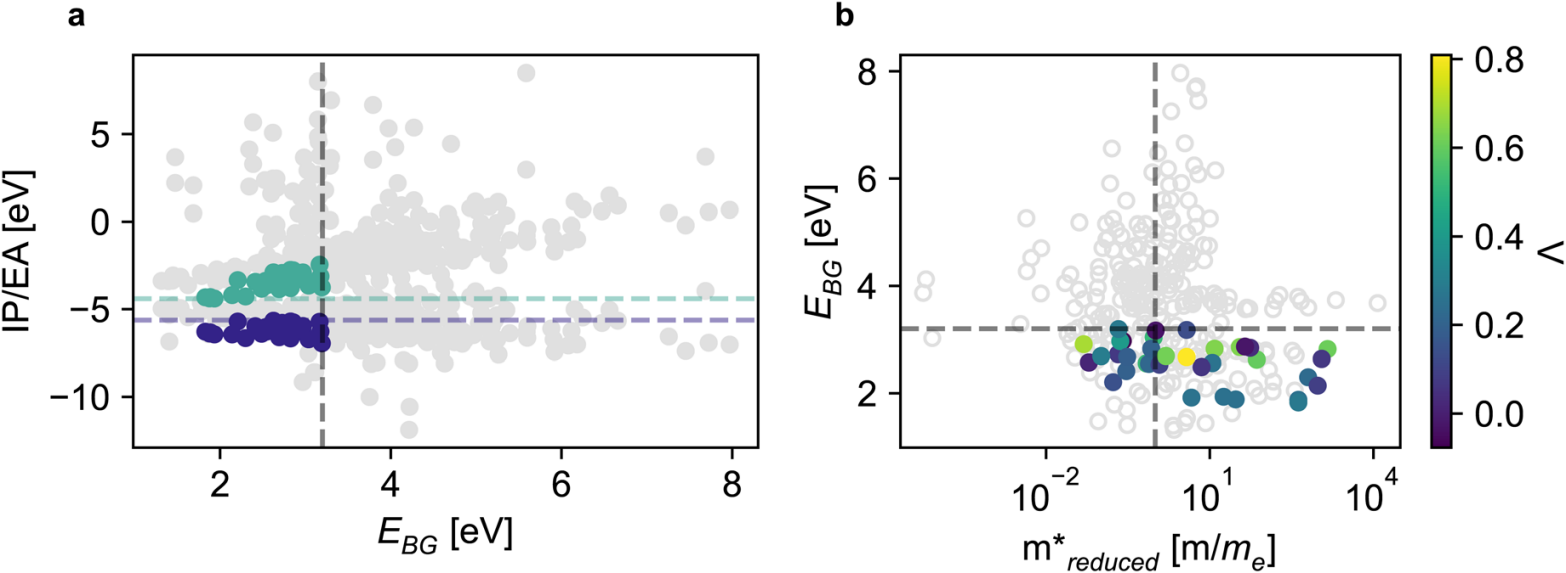
(a) Assessing thermodynamic feasibility based on vacuum-aligned IP/EA alignment with OER/HER (indigo blue/jungle green) potentials, respectively. (b) Simultaneous evaluation of DFT-based descriptors, with colored dots representing alignment to HER/OER, and color gradient indicating charge recombination likelihood. Horizontal line: visible light absorption limit (3.2 eV). Vertical line: 
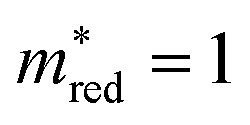
 threshold for mobile charge carriers.

The evaluated MOFs were classified based on visible light absorption and the thermodynamic feasibility of desired redox reactions. Fig. 2a displays the alignment of the structures based on their band gaps and ionization potential/electron affinity (IP/EA) alignment. Specifically, two points in the same vertical line correspond to the IP and EA for the same structure. Structures with a band gap in the visible range (1.6 eV < *E*_BG_ < 3.2 eV) are located to the left of the grey dashed line, accounting for 41% of the evaluated structures (among which 80% are CDP–MOFs).

Out of all MOFs we evaluated, approximately 11% (out of which 91% are CDP–MOFs) exhibit proper simultaneous alignment of their IP and EA with the redox potential needed for HER and OER. This means that the addition of CDP–MOFs provided most of the true positives to the machine learning training and test sets. Without those structures, the model performance would likely have been much poorer.

The subset of MOFs with adequate band alignment for HER and OER consists of 34% of the closed shell structures and 10% of the open shell structures, forming the list of the filtered MOFs with the potential to facilitate overall water splitting.

Fig. 2b shows the simultaneous analysis of all four photocatalytic descriptors. The colored points represent structures that have favorable IP/EA for the case study of overall water splitting and band gaps in the visible light (lower than 3.2 eV, that is, below the horizontal dashed line). Structures located to the left of the vertical dashed line exhibit 
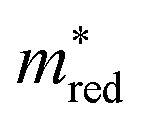
 lower than 1 *m*_e_, suggesting enhanced mobility of charge carriers. Notably, green and blue points correspond to lower *Λ*, indicating reduced probability of electron and hole recombination posterior to excitation.

Table 1 highlights some of the most promising candidates for OWS amidst the evaluated MOFs. Reference values are included and were computed using the same workflow for a reported photocatalytically active MOF.^50^ A noteworthy observation is that most candidates are rod-like MOFs, which aligns with our rationale for designing MOFs featuring low-dimensional clusters as potential photocatalysts. MOFs with Ti-based metal nodes (*e.g.*, mn21 or mn23) also stand out. Strikingly, the MOF with the Mn(iii)/Mn(iv) (mn39) cluster is the only open shell structure in the list. This cluster was selected to mimic the OER center in chlorophyll,^41,42^ and to the best of our knowledge has not yet been used as a metal node in a MOF. It is responsible for the pivotal 4-electron transfer step responsible for generating O_2_ during photosynthesis.^41^

**Table 1:** Best candidates for overall water splitting among DFT-evaluated QMOF and CDP–MOF based on filters: visible light absorption (1.6 eV < *E*_BG_ < 3.2 eV), thermodynamic feasibility (redox potentials alignment with vacuum), effective masses 
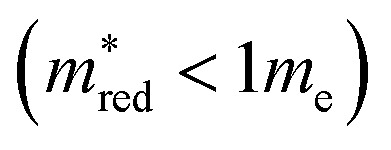
 and charge separation descriptor (*Λ* < 0.5). Metal node and organic linker names match our database. Visual representations of metal nodes and linkers are in ESI (Fig. S2–S12). Computed descriptors for NTU-9 (a filtered MOF from photocatalytically active experimental MOFs) are included.^20,50^ H_4_DOBDC stands for 2,5-dihydroxyterephthalic acid^a^

Name	Metal node	Linker	*E*_BG_	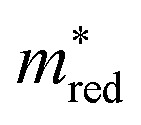	*Λ*	CT*
mn21-ol36	mn21 (ref. 37)	ol36	2.57	0.77	0.22	Yes
mn21-ol23	mn21 (ref. 37)	ol23	2.83	0.84	0.20	Yes
mog-ol15-mn24	mn24^⋄^^53^	ol15	2.97	0.25	0.08	No
mog-ol38-mn24	mn24^⋄^^53^	ol38	2.73	0.21	0.07	No
mog-ol21-mn24	mn24^⋄^^53^	ol21	2.21	0.17	0.14	No
mog-ol50-mn24	mn24^⋄^^53^	ol50	2.97	0.23	0.41	No
mn23-ol87	mn23^⋄^^38^	ol87	2.41	0.29	0.20	No
mn23-ol15	mn23^⋄^^38^	ol15	2.67	0.31	0.19	No
vcs-mn39-ol3	mn39^•^^42^	ol13	2.20	0.27	0.13	Yes
qmof-2e3e058	—	—	2.69	0.10	0.29	—
qmof-8b5a121	—	—	3.19	0.21	0.28	—
NTU-9 (ref. 50)	Ti	H_4_DOBDC	2.69	0.64	0.62	—

a ^⋄^ Rod-like MOFs, ^•^ open shell (PBE0 calculations for energy-based descriptors and *Λ*), * denotes the likelihood of LMCT based on cube analysis for electron and hole injection, but further excited-state calculations should be performed to confirm.

Overall, the predominance of CDP–MOFs in Table 1 indicates success in populating the MOF design space with prospective MOF photocatalysts through *in silico* design. To further validate our design criteria, we investigated how each descriptor is affected by the presence of each building block.

Among metal nodes, Au(iii)-pyrazolate (mn8), Ti(iv)/Zr(iv) (mn21), V(iv) (mn33) and Co(iii) (mn34) clusters stood out by meeting multiple criteria. Most MOFs with Au(iii)-pyrazolate clusters met the criteria for visible light absorption (Fig. S45a†), charge separation (Fig. S45b†), and alignment for HER and OER (Fig. S33†). MOFs with Ti(iv)/Zr(iv) metal node (mn21) displayed lower electron effective masses (Fig. S47b†), adequate alignment for HER and OER (Fig. S33†), and band gap in the visible range (Fig. S47a†). The designed V(iv) MOFs (with mn33) met the criteria for visible light absorption (Fig. S49a†), alignment for HER (Fig. S33†), and mobile charge carriers (hole, in particular, see Fig. S49b†). Lastly, MOFs with the Co(iii) metal node mn34 displayed adequate alignment to HER (Fig. S33†), band gap in the visible range (Fig. S50a†), and lower effective masses for both electron and hole on average than MOFs without this node (Fig. S50b and c†).

When considering the effect of the linker on the photocatalytic descriptors, we noticed that MOFs with thiophene (in ol3) and thiadiazole (in ol48) groups in the linker also met multiple criteria. MOFs containing thiophene (ol3) displayed band gap in the visible range (Fig. S51a†), alignment to HER (Fig. S37†), and lower electron effective masses (Fig. S51b†). MOFs with thiadiazole (ol48) met the criteria for visible light absorption (Fig. S53a†), alignment to HER (Fig. S37†), and charge separation (Fig. S53b†).

Finally, we observed that, in general, CDP–MOFs with lower effective masses showed, in detriment, higher chances of charge recombination. This means that whenever the charge transport descriptor is in the desired range, the charge separation descriptor is not. However, rod MOFs (Fig. S58†) and open shell MOFs (Fig. S59†) stood out by meeting both charge transport and charge separation criteria simultaneously. Additionally, a few MOFs are predicted to have low-lying linker-to-metal node charge transfer (*e.g.*, mn21-ol36, with the Ti/Zr metal node, and vcs-mn39-ol3, with the Mn_4_Ca cluster), and also contributed to meeting both criteria at the same time.

### Machine learning

#### Model performance

To evaluate the ∼20 000 remaining MOFs in QMOF and CDP–MOF, we make use of two pre-trained machine-learning models, MOFTransformer and GPT-J to predict five photocatalytic descriptors—charge transport 
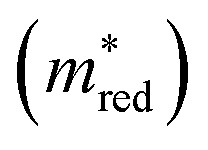
, charge separation (*Λ*), visible light absorption (VIS), and alignment to the photoredox reactions (HER and OER). For each of these properties, we develop a binary classification model to predict whether a given MOF meets the criteria (see ESI† for thresholds).

To fine-tune these ML models, we evaluated 314 MOFs using the DFT calculations described in the previous section. These MOFs include the 146 CDP–MOFs discussed in the previous section, 154 structures from the QMOF database,^25^ and 14 experimentally evaluated MOF photocatalysts^20^ (see ESI†). We highlight the role of the CDP–MOFs in enhancing the number of true positives in the training and test sets, especially for HER and OER: as discussed above, 91% of MOFs that exhibit proper alignment for HER and OER are CDP–MOFs.

The fine-tuned MOFTransformer and the GPT-J model can reasonably accurately predict the five descriptors. For almost all trained models, the F1 test scores are larger than 75%. Moreover, the models' reliability can be corroborated by the consistent, high (>70%) agreements between both models for all property predictions on the QMOF and the CDP–MOF structures (see Fig. S29 in the ESI†). A notable exception is the lower performance of models predicting the binary class of 
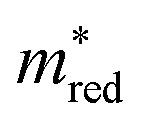
, which should thus be interpreted carefully.

We computed the DFT descriptors of MOFs predicted by either MOFTransformer or GPT-J to meet all the criteria. The distribution of the DFT computed properties can be found in the ESI (Fig. S32†). Given that our strategy was to train one model per criteria, the selection of MOFs predicted to meet all of them suffers from an accumulation of errors. Therefore, we do not expect that a high number of structures would meet all the criteria. Instead, we hoped to get at least a few from the list, which we obtain cost-effectively rather than through a brute-force screening of the whole QMOF and CDP–MOF databases (total of ≈21k MOFs). Indeed, we were able to confirm four MOFs that meet all the criteria: qmof-d2f08f6, qmof-b46b341, qmof-72626ed, and qmof-99cef49. Another work also predicted the latter as a good candidate for water splitting.^54^

#### Exploring the chemical design space

Wang *et al.*^54^ screened the QMOF database for photocatalysis using a hierarchical approach, where subsequent filters were used, and a machine-learning model was trained to predict the band gap.

The remaining photocatalytic properties were computed for a much smaller set of the QMOF database. As we have a machine learning model for all descriptors, we can analyze the complete chemical design space.

For this, we use the MOFTransformer, which allows us to interpret relationships between MOFs in the chemical design space. For this, we use the MOFTransformer to project a MOF structure onto a vector of length 768. Upon fine-tuning, the entries of this vector change. If the distance between two vectors in this high-dimensional space is small, the predicted properties are expected to be similar. This similarity in a 768-dimensional space can be visualized in 2D using the UMAP projection. These UMAP plots help us visualize regions in the design space where high-performing materials for a given property are concentrated.

**Fig. 3:**
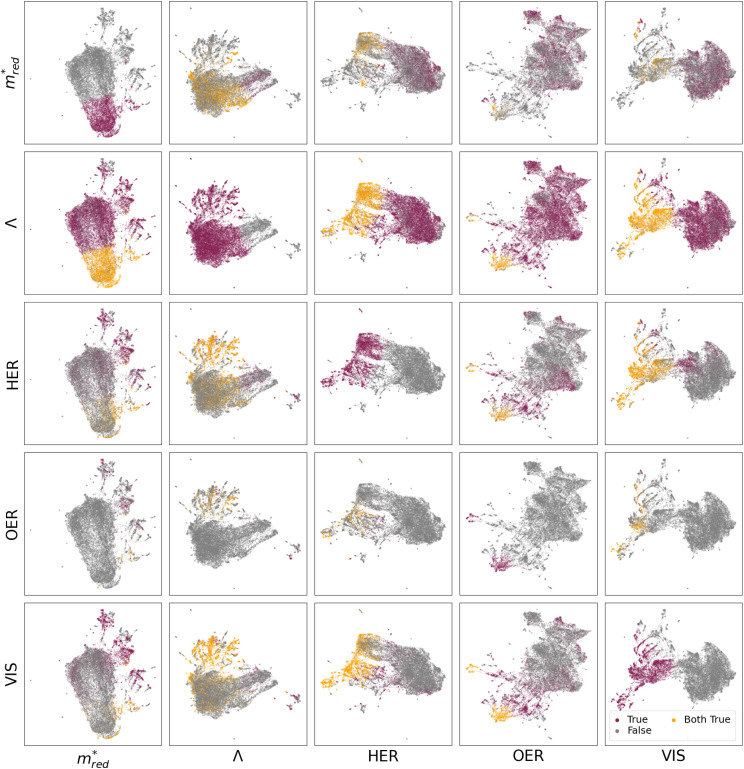
The five descriptors, charge transport 
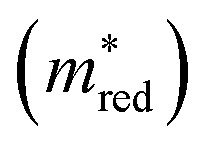
, charge separation (*Λ*), visible light absorption (VIS), and alignment to the photoredox reactions (HER and OER), shown in chemical design space. These figures show a UMAP projection of the 768-dimensional vector characterizing the similarity of MOF fine-tuned on one of the descriptors. All figures in a column are fine-tuned on the same descriptor X. Each row represents a different descriptor Y; a MOF is represented with a grey dot if the criteria of descriptor Y are not met and a purple dot if the criteria are met. A MOF is represented with an orange dot if the MOF meets both criteria Y and X. Hence, the diagonal (X, X) has only grey and purple dots, and the number of orange dots in figures (Y, X) and (X, Y) is by definition equal.

Fig. 3 displays the different UMAP projects of the five descriptors. For a full picture, see Fig. S26a–S28a,† where a distinction is made between QMOF and CDP–MOF in the chemical design space.

Let us first focus on the diagonal of Fig. 3. In these figures, we plot MOFs in the combined QMOF and CDP–MOF databases that meet the corresponding criteria in purple and those that do not meet the criteria in grey. Let us look at the effective mass 
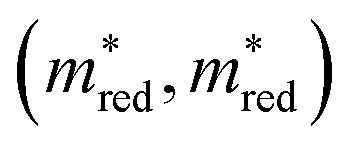
, spatial overlap (*Λ*, *Λ*), hydrogen evolution reaction (HER, HER), and visible light absorption (VIS, VIS). Their UMAPs show that a large fraction of the chemical design space has materials that meet the criteria for these descriptors. For OER (OER, OER), however, the materials that meet this criteria occupy smaller pockets in the design space.

It is interesting to study which combination of properties is a potential bottleneck in designing an optimal photocatalytic material. We must inspect the off-diagonal entries in Fig. 3 to see this.

For example, figure 
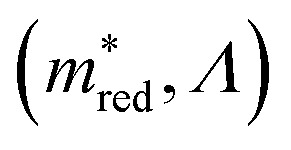
 displays in purple those materials that meet the *Λ* criteria, plotted on a UMAP fine-tuned on the effective mass 
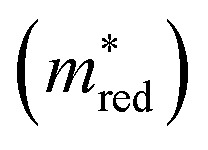
. The materials that obey both criteria are plotted in orange. The sum of orange and purple materials in 
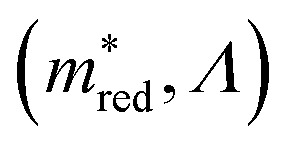
 is thus equal to the number of purple materials in 
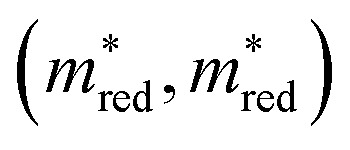
. Figure 
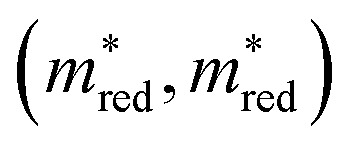
 shows that the materials that meet the 
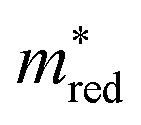
 criteria are concentrated in the bottom part. Figure 
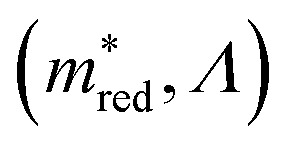
 projects those MOFs that meet the *Λ* criterion on the 
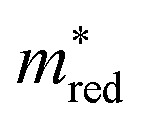
-UMAP. These materials almost uniformly cover the entire 
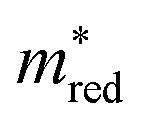
-UMAP. Hence, at the bottom of this graph, we see the orange materials that meet both criteria.

Equivalently, we can also look at figure 
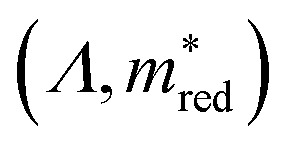
. By definition, the number of orange dots is the same as in 
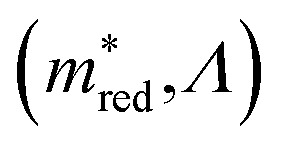
. The total number of materials that meet the 
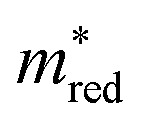
 criterion is less than those that meet the *Λ* criterion; we have fewer purple dots, but also, here, they cover most of the design space. These observations show that there is little correlation between these two criteria.

The situation is very different for OER. The diagonal (OER, OER) already indicates that the number of MOFs that meet this criterion is small; we only see two pockets in the design space. If we then look at the off-diagonal, we see, for example, in the figure (OER, VIS), that both pockets are orange, but in the 
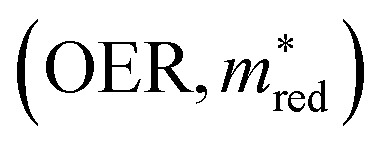
, we see that only one pocket stays orange.

From these figures, we can conclude that many materials meet both the *Λ* and 
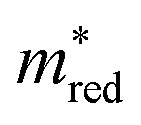
 criteria. This number decreases if we also require VIS and HER, but the real bottleneck is the combination with OER. Indeed, it is well established in the literature that the evolution of O_2_ is more challenging than that of H_2_. The main reason for this is based on kinetics, where the transfer of 4 electrons involved in OER makes it a slower process than that of 2 electrons for HER.^55^ Additionally, the redox potential for OER is 1.23 eV higher than that of HER. The latter could explain the challenge of finding materials with suitable band alignment for OER. As an alternative, other oxidation half-reactions have already been proposed to couple with HER.^55^

Arguably, one of the most valuable insights we can derive from the UMAPs in Fig. 3 is the structural similarity and clustering patterns. Let us focus on the OER criteria, which, as discussed above, is the main bottleneck. Specifically, we focus on the OER row, where the plots show structures that meet the OER requirement in the contextualized feature spaces of all criteria 
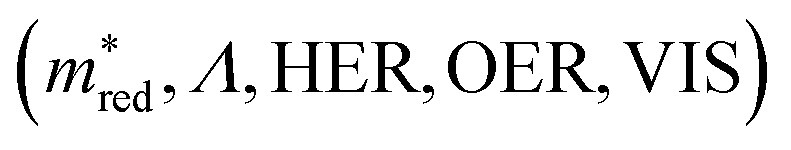
. Across this row, we notice the clustering of MOFs that meet the OER criteria. This means there should be some structural similarity between the MOFs predicted to align with OER. If we analyze the organic linkers associated with those MOFs, we see a trend, as displayed in Fig. S77–S80.† In particular, the presence of the following motifs in the organic linker could be correlated with the predicted alignment to OER: benzocyclobutene, thienothiadiazole, 2,4-hexadiynedioic acid (with alternating C–C triple bonds), and thieno[3,4-*b*]pyrazine.

**Fig. 4:**
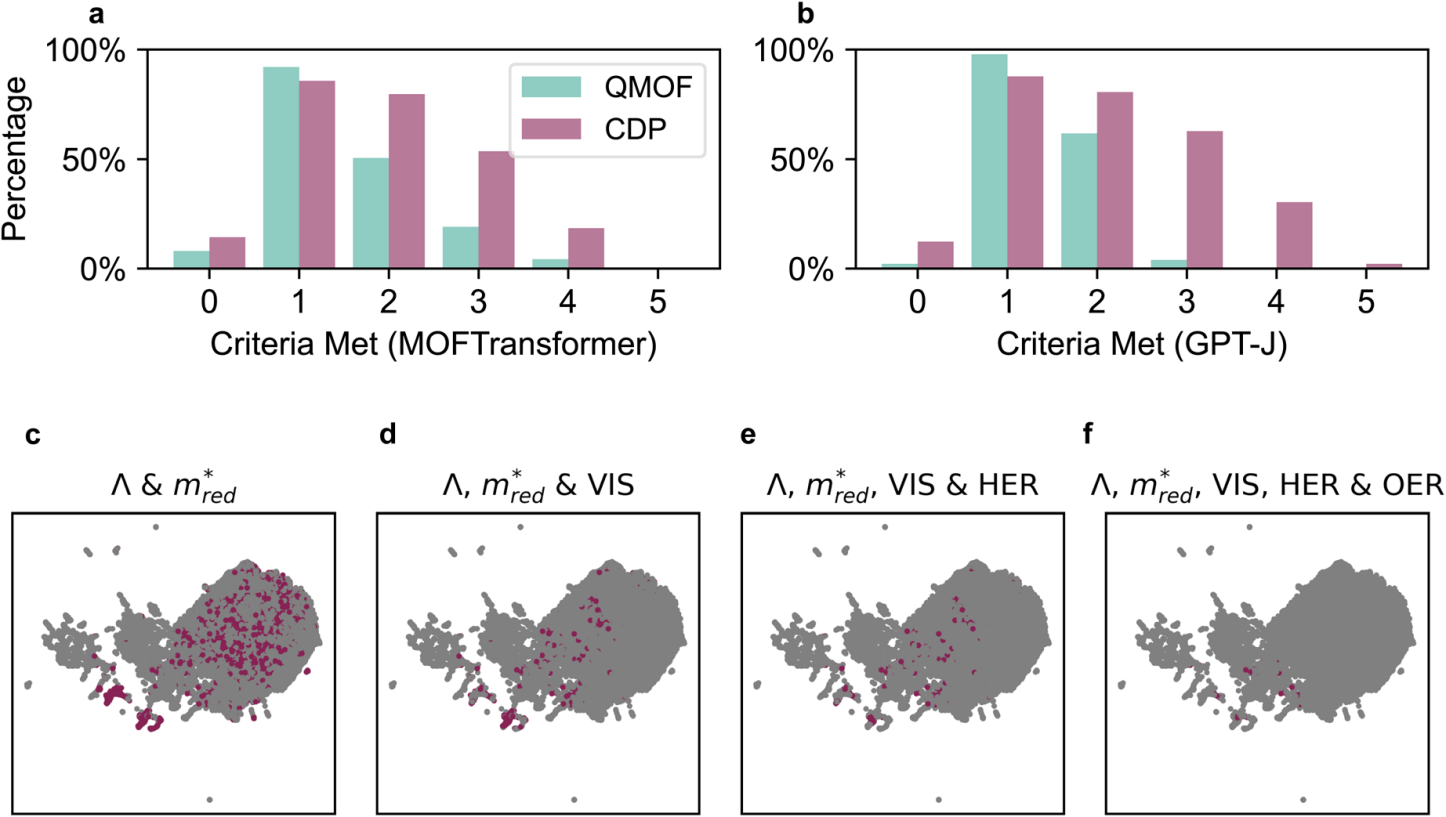
Comparison between QMOF and CDP regarding the number of criteria met (cumulative count) for (a) MOFTransformer and (b) GPT-J predictions. (c–f) Visualization of MOFs' unweighted chemical design space as UMAP projections, highlighting the number of structures simultaneously meeting different photocatalytic descriptors (from more to less common regarding positive outcomes).

We can also summarize these results by plotting the UMAP projection of the 768-dimensional vector that has not been fine-tuned to any of the descriptors. In the sequence in Fig. 4c–f, we first plot in purple those materials that meet both the *Λ* (<0.5) and 
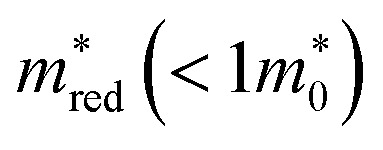
 criteria. The next figures are MOFs that also meet the VIS (1.23 eV < *E*_BG_ < 3.2 eV) criteria, followed by HER (−4.4 eV w.r.t. vacuum at pH 0). The last figure displays the structures that meet all criteria (including OER, −5.63 eV w.r.t. vacuum at pH 0).

It is interesting to compare these numbers for the QMOF and CDP–MOFs. We note that the CDP–MOFs database has indeed a significantly higher percentage of structures that meet two or more criteria, based on both MOFTransformer (Fig. 4a) and GPT-J (Fig. 4b) predictions. QMOF, on the other hand, has a higher percentage of structures meeting one criterion. This is likely associated with a high percentage of QMOFs with true predictions for *Λ* by both MOFTransformer (84%) and GPT-J (90%). For more details on the percentage and total number of true predictions per criteria, see Fig. S31.†

### Structural analysis

We performed structural analysis to gain more insights into the machine-learning predictions. Using MOFid^52^ fragmentation and bootstrapped effect sizes,^56^ we evaluated which building blocks and structural motifs could be correlated with a better performance for each predicted descriptor. For the following discussion, we consider only the motifs for which both GPT-J and MOFTransformer predictions agree. We focused on motifs present in more than 50 MOFs, allowing us to gain statistical insights.

Among metal nodes, metal halide motifs stood out for 
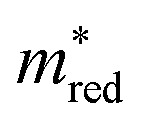
 and HER descriptors. In fact, recent studies point out that introducing metal halide motifs in MOFs can enhance the carrier transport properties and promote light-driven HER.^57,58^ Mn and Zn also correlate with improved band alignment for HER. Regarding the charge separation descriptor (*Λ*), Ba, Co, and Nd stood out. Recent studies on a Ba-MOF reported LMCT bands with increased exciton radiative lifetimes.^59^ Also, a Nd-MOF is reported to have an improved charge transfer rate when compared to its Fe analogue.^60^

**Fig. 5:**
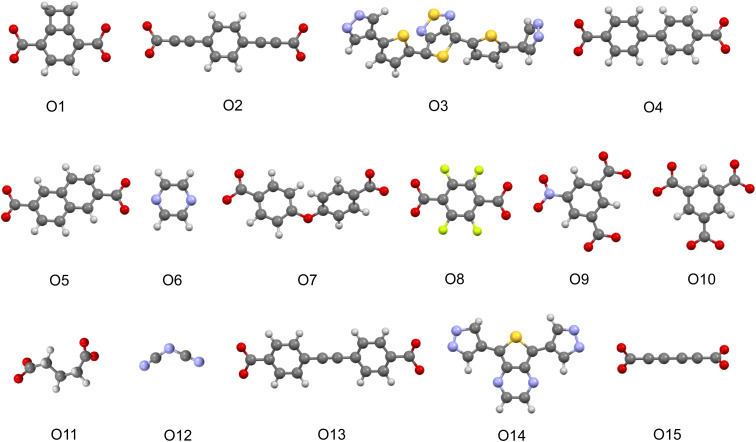
Visualization of highlighted linker motifs.

If we turn our attention to the organic linkers, we see on Fig. 5 the presence of thiophene, pyrazine, and azole-containing functional groups among relevant motifs. Those groups are common among the organic linkers we chose to design MOF photocatalyst candidates. In previous studies, thiadiazole showed a correlation with lower band gap and favorable charge recombination descriptor values for COFs.^26^ Thiophene groups, on the other hand, are known for their high π-electron density and have been widely used in MOFs and other photoactive materials (Table 2).^61^

**Table 2:** Relevant motifs with agreement between the two ML models, for each target property predictions in QMOF + CDP–MOF

	Metal node	Linker^a^
VIS	—	O1–4
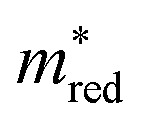	Cl[Zn]Cl, [Cs]	O5–8
*Λ*	[OH_2_][Nd][OH_2_], [OH_2_][Co][OH_2_], [Ba]	O9–12
HER	[OH_2_][Zn], [OH_2_][Mn], [Zn], I[Cu][Cu]I	O1, O3–4, O13–14
OER	—	O1, O3, O14–15

a See Fig. 5 for visualization.

## Conclusions

By combining three pillars—MOF design based on chemical insights, property evaluation with a DFT workflow, and machine-learning predictions—, we developed an efficient strategy to advance the exploration of the MOF design space for photocatalytic applications. We could obtain a reasonably accurate machine-learning model that can be used to predict the photocatalytic potential of a MOF using a relatively small training set. This is a nice illustration of the power of the MOFTransformer model, in which general knowledge is leveraged by fine-tuning relatively small datasets. However, for this to work optimally, one does need a balanced dataset. We used chemical insights and intuition to create our CDP–MOF database to obtain such a balanced dataset.

In this work, we focused on hydrogen and oxygen evolution reactions. Some descriptors are specific for these reactions (band-edge alignment), and some descriptors need to be fulfilled for any photocatalytic reaction (suitable band gap, low spatial overlap of electron and hole, and low charge-carrier effective masses). Therefore, for other redox reactions, only models for band-edge alignment should be fine-tuned accordingly, and our results for the three general criteria still hold. We showed that there is an area in the chemical design space of MOFs where these three general criteria are met. This area can be enriched by focusing on designing materials with suitable charge-carrier effective masses and band gaps, given that the spatial overlap criterion is more widely met.

The most important conclusion of this work is that the bottleneck in designing MOFs for overall water splitting lies in the alignment to OER. Our study shows that it is relatively easy to identify a large number of MOFs that have suitable band gaps and band alignment to HER. However, the total number of these MOFs that also have appropriate alignment to OER dropped significantly. Hence, efforts should focus on generating more structures in the region of the chemical design space where OER aligns.

## Data availability

All the MOF structures, the features used for diversity analysis, and their photocatalytic descriptors are available on the Zenodo platform at https://doi.org/10.5281/zenodo.14727983.^62^

## Author contributions

S. M. and B. M. contributed equally to this work. A. O-G., S. M., B. M., and B. S. designed the project. A. O-G., S. M., B. M., and X. J. performed building block selection and curation. S. M. and X. J. generated the *in silico* MOF structures, and S. M. performed diversity analysis. B. M. and A. O-G. developed the open-shell workflow and performed all the DFT calculations. B. M. performed structural analysis and literature review on the selected building blocks. F. M. and J. V. H. performed the supervised machine-learning classifications. B. S. and S. G. led the project and provided directions. The manuscript was written with contributions from all authors. All authors have approved the final version of the manuscript.

## Conflicts of interest

The authors declare no competing interests.

## Supplementary Material

SC-OLF-D5SC01100K-s001

SC-OLF-D5SC01100K-s002
