# Supplementary figures and images for: Exploring the chemical design space of metal–organic frameworks for photocatalysis (part 2 of 2)
Source: Chem Sci. 2025 May 13;16(25):11434–46. doi: 10.1039/d5sc01100k (PMC12107286; doi:10.1039/d5sc01100k)

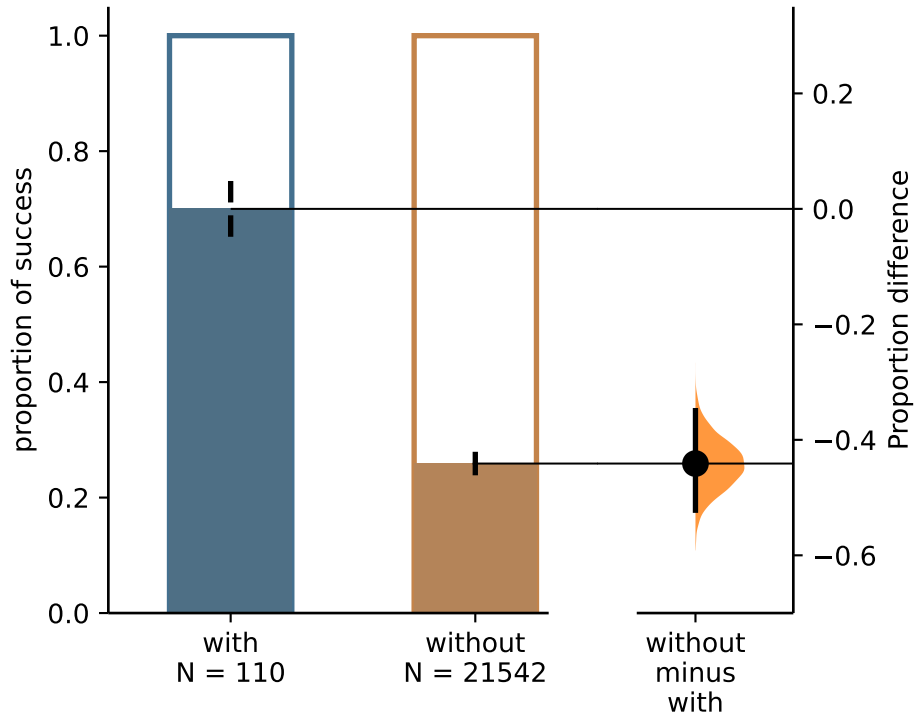

Supplement: SC-016-D5SC01100K-s001 [file SC-016-D5SC01100K-s001.zip › ESI/si_images/structural_analysis/mred_moft_mn15_mean_diff.pdf]

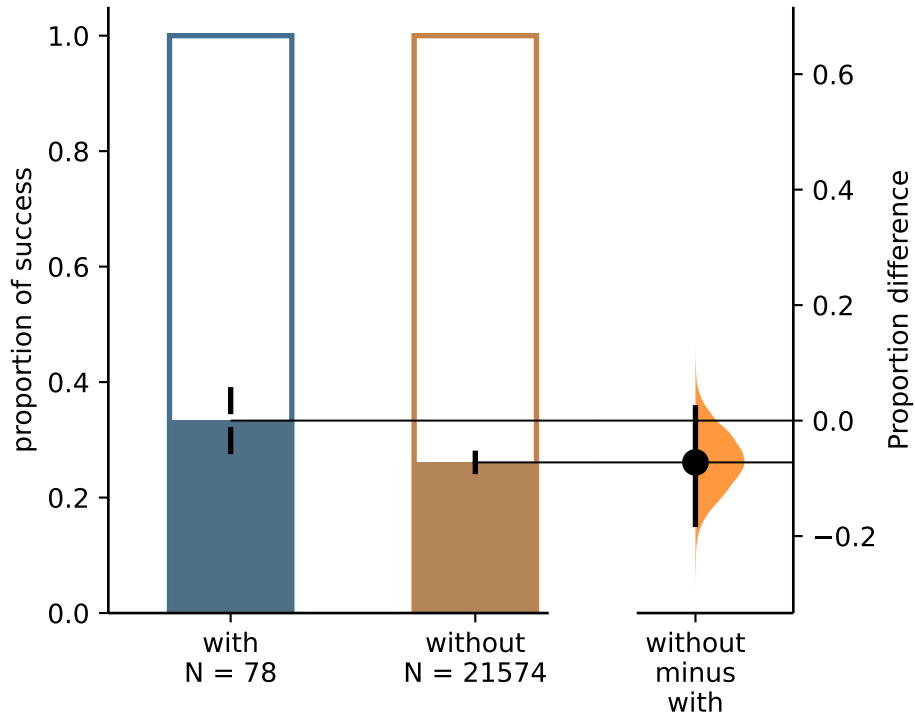

Supplement: SC-016-D5SC01100K-s001 [file SC-016-D5SC01100K-s001.zip › ESI/si_images/structural_analysis/mred_moft_mn7_mean_diff.pdf]

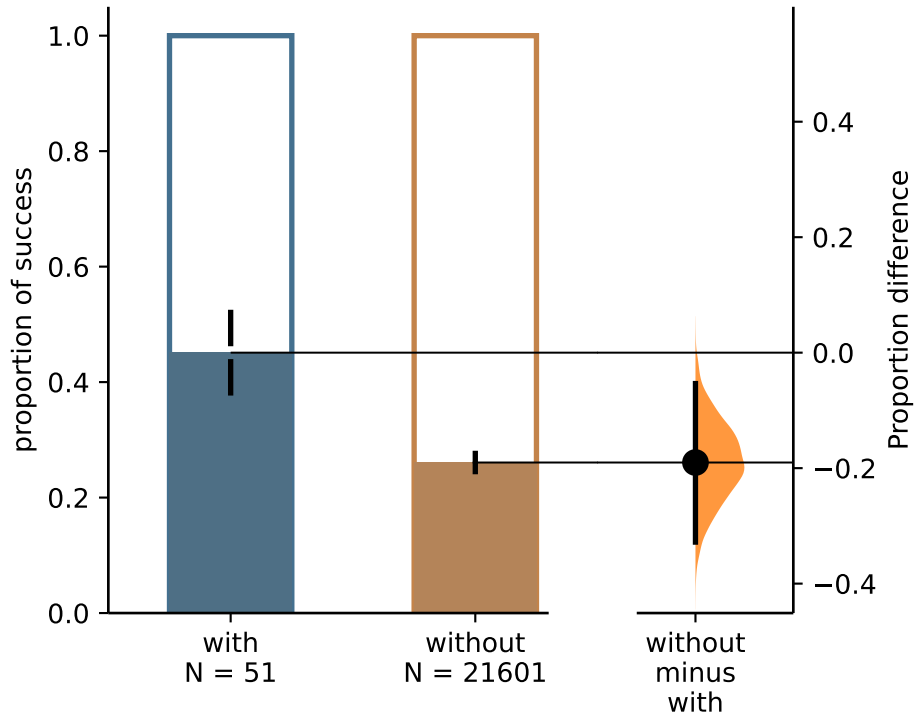

Supplement: SC-016-D5SC01100K-s001 [file SC-016-D5SC01100K-s001.zip › ESI/si_images/structural_analysis/mred_moft_ol18_mean_diff.pdf]

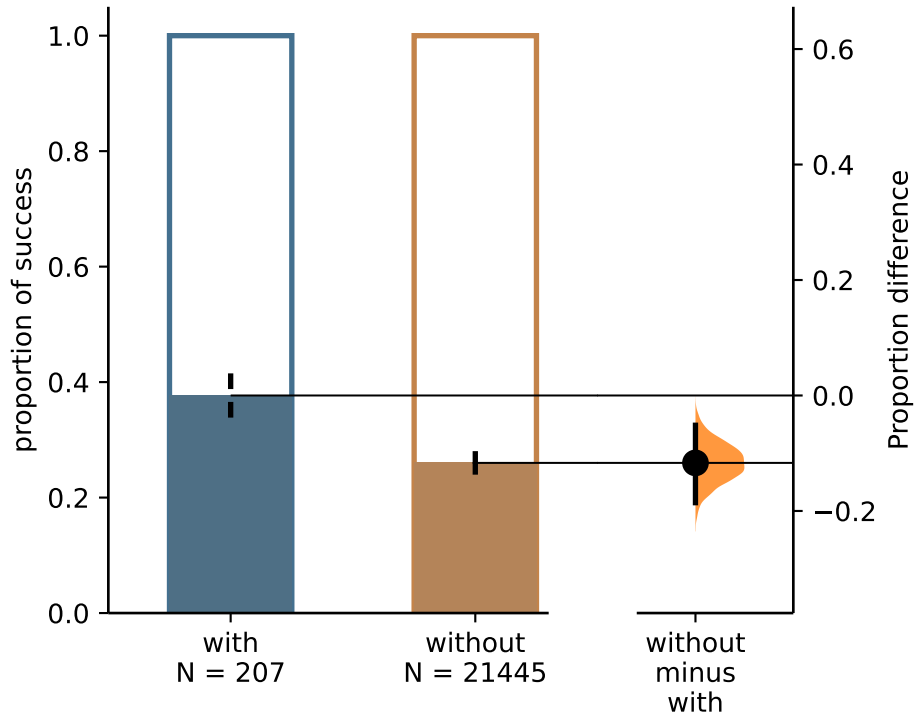

Supplement: SC-016-D5SC01100K-s001 [file SC-016-D5SC01100K-s001.zip › ESI/si_images/structural_analysis/mred_moft_ol48_mean_diff.pdf]

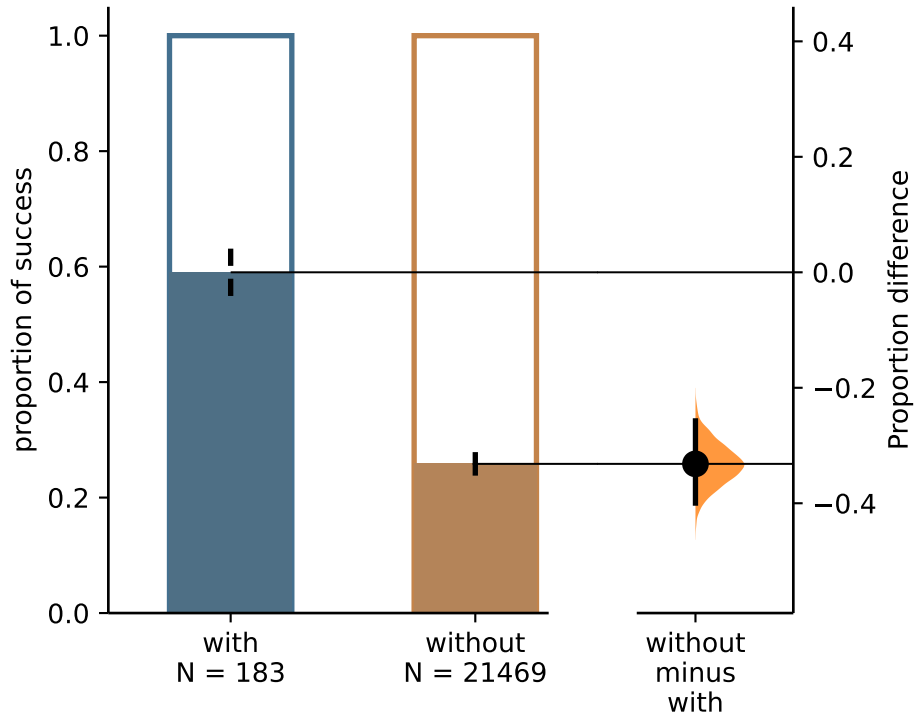

Supplement: SC-016-D5SC01100K-s001 [file SC-016-D5SC01100K-s001.zip › ESI/si_images/structural_analysis/mred_moft_ol58_mean_diff.pdf]

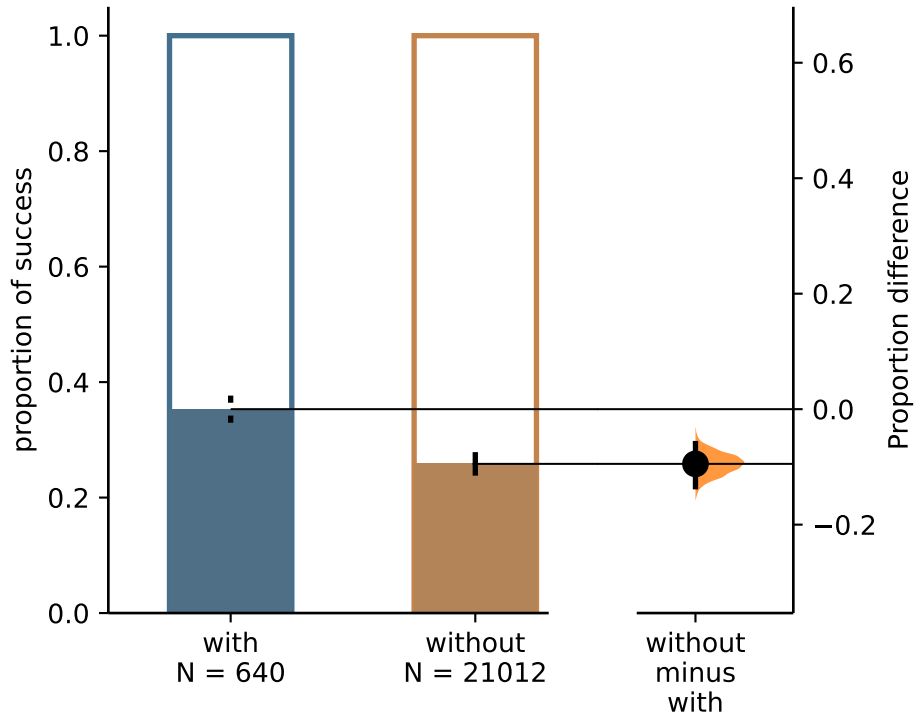

Supplement: SC-016-D5SC01100K-s001 [file SC-016-D5SC01100K-s001.zip › ESI/si_images/structural_analysis/mred_moft_ol70_mean_diff.pdf]

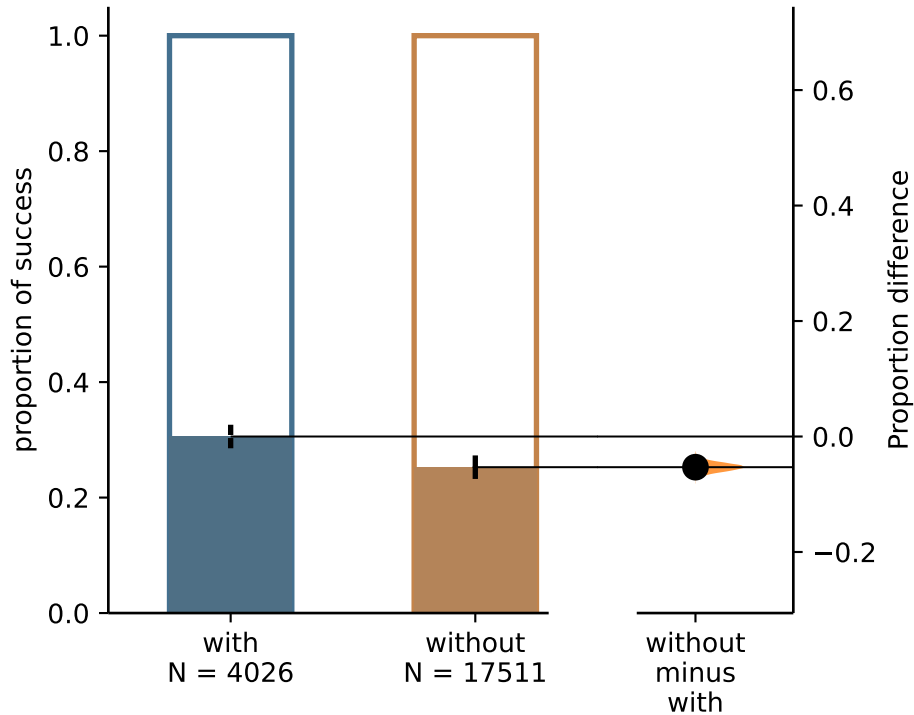

Supplement: SC-016-D5SC01100K-s001 [file SC-016-D5SC01100K-s001.zip › ESI/si_images/structural_analysis/mred_moft_tp17_mean_diff.pdf]

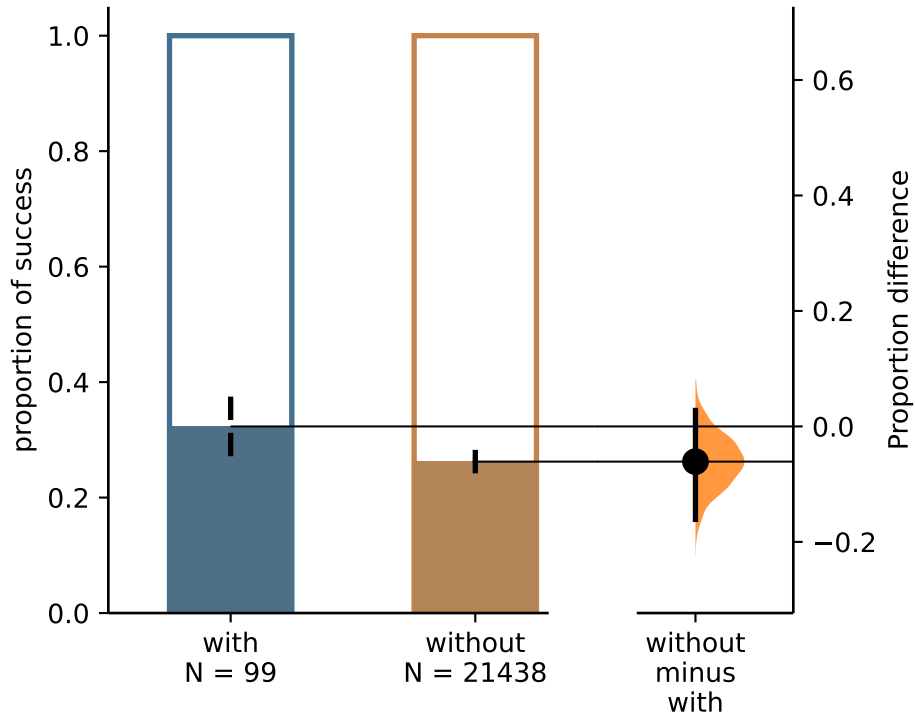

Supplement: SC-016-D5SC01100K-s001 [file SC-016-D5SC01100K-s001.zip › ESI/si_images/structural_analysis/mred_moft_tp28_mean_diff.pdf]

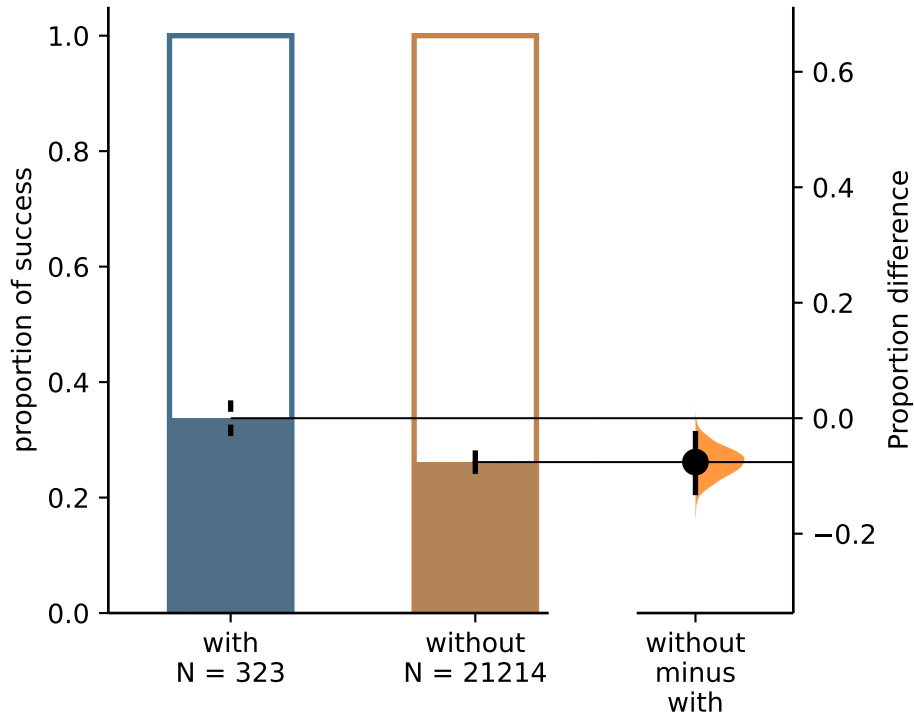

Supplement: SC-016-D5SC01100K-s001 [file SC-016-D5SC01100K-s001.zip › ESI/si_images/structural_analysis/mred_moft_tp2_mean_diff.pdf]

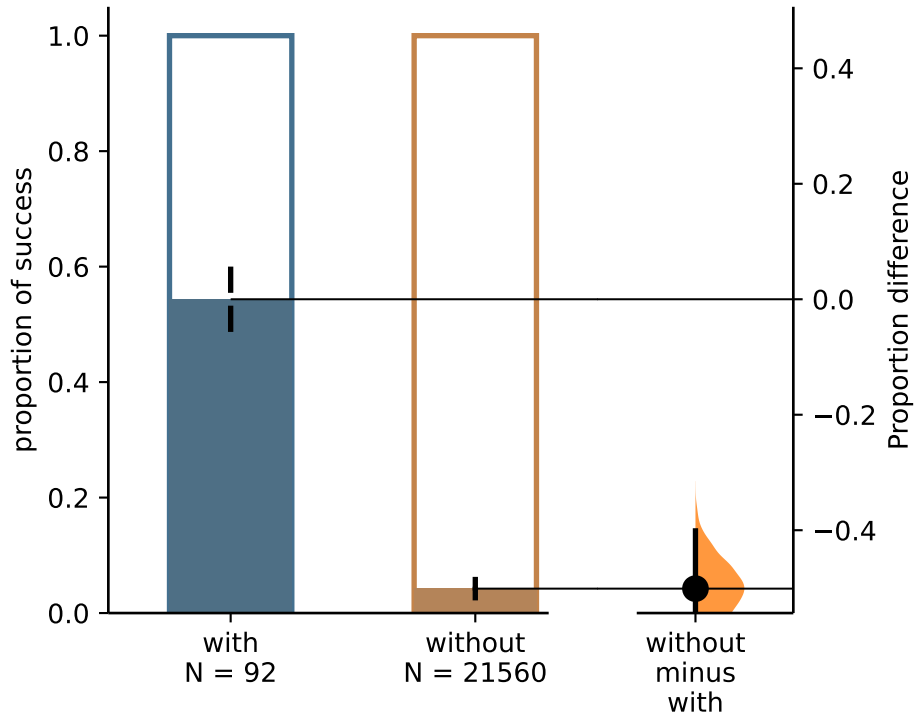

Supplement: SC-016-D5SC01100K-s001 [file SC-016-D5SC01100K-s001.zip › ESI/si_images/structural_analysis/oer_gpt_ol17_mean_diff.pdf]

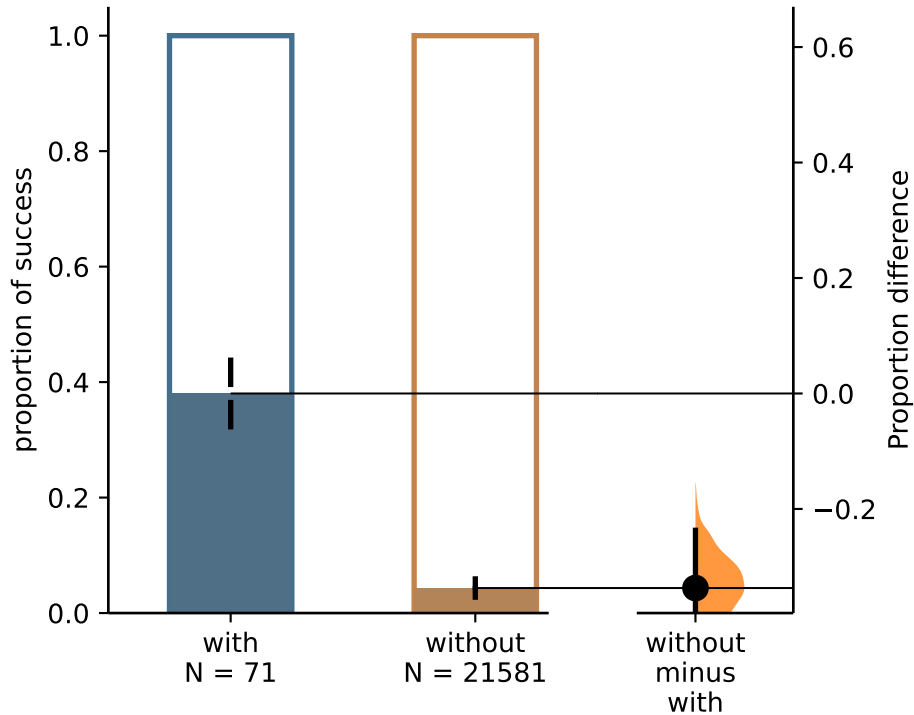

Supplement: SC-016-D5SC01100K-s001 [file SC-016-D5SC01100K-s001.zip › ESI/si_images/structural_analysis/oer_gpt_ol1_mean_diff.pdf]

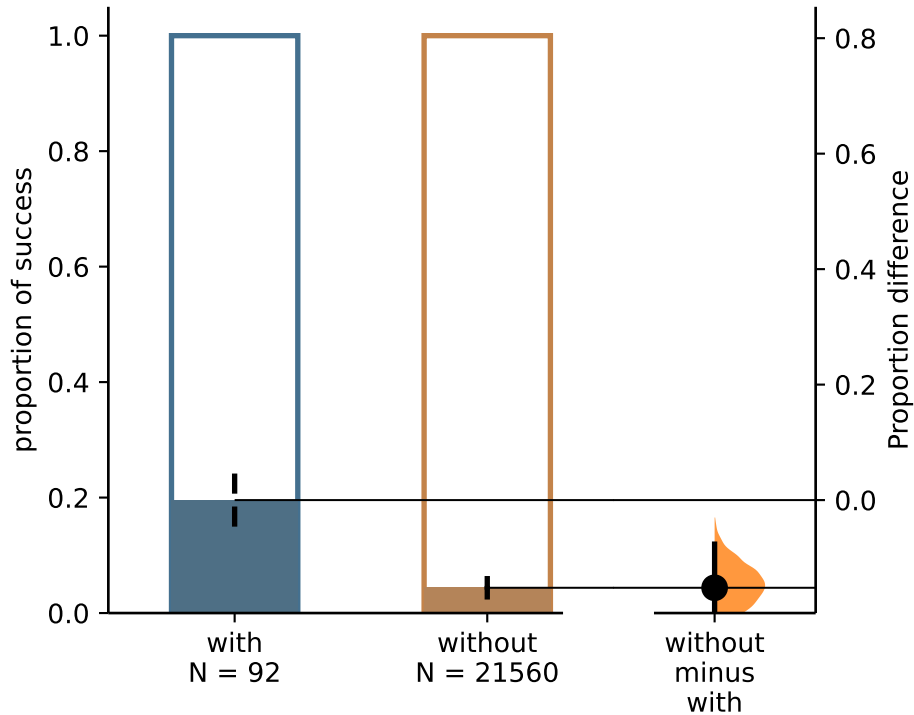

Supplement: SC-016-D5SC01100K-s001 [file SC-016-D5SC01100K-s001.zip › ESI/si_images/structural_analysis/oer_gpt_ol20_mean_diff.pdf]

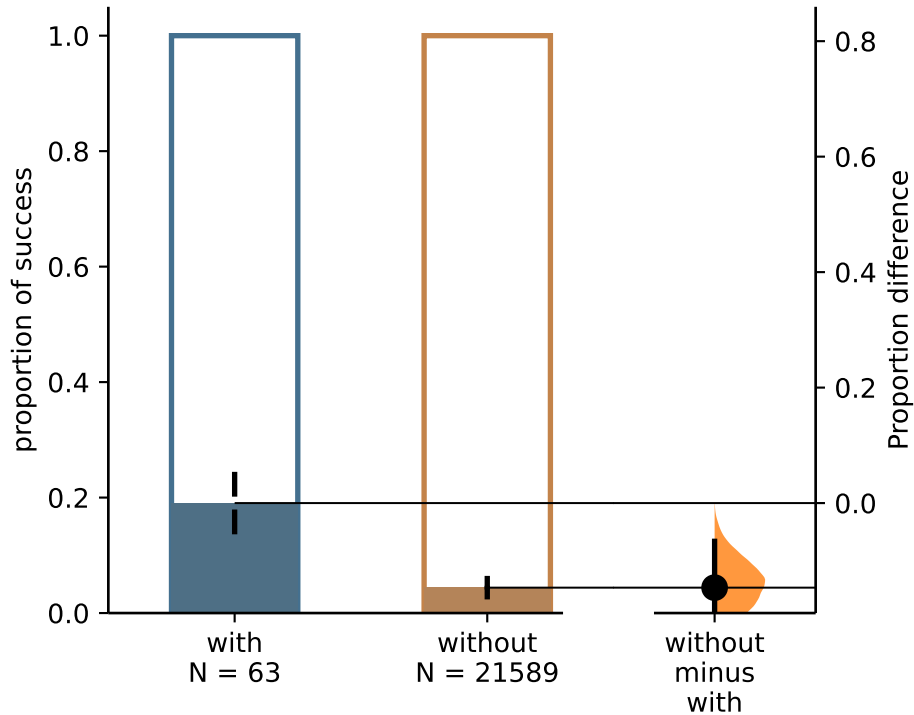

Supplement: SC-016-D5SC01100K-s001 [file SC-016-D5SC01100K-s001.zip › ESI/si_images/structural_analysis/oer_gpt_ol30_mean_diff.pdf]

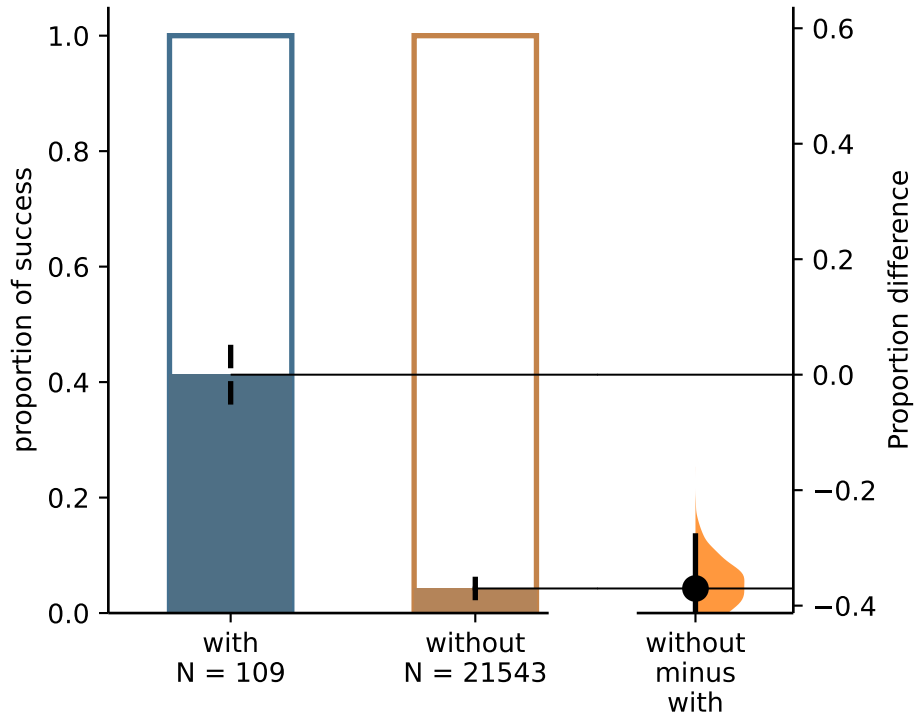

Supplement: SC-016-D5SC01100K-s001 [file SC-016-D5SC01100K-s001.zip › ESI/si_images/structural_analysis/oer_gpt_ol5_mean_diff.pdf]

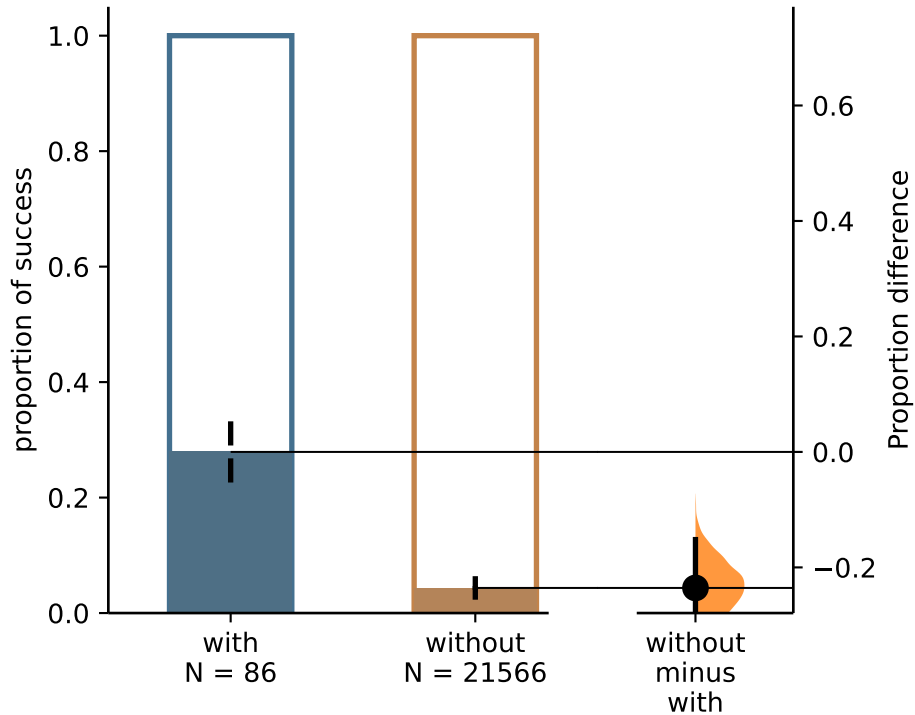

Supplement: SC-016-D5SC01100K-s001 [file SC-016-D5SC01100K-s001.zip › ESI/si_images/structural_analysis/oer_gpt_ol78_mean_diff.pdf]

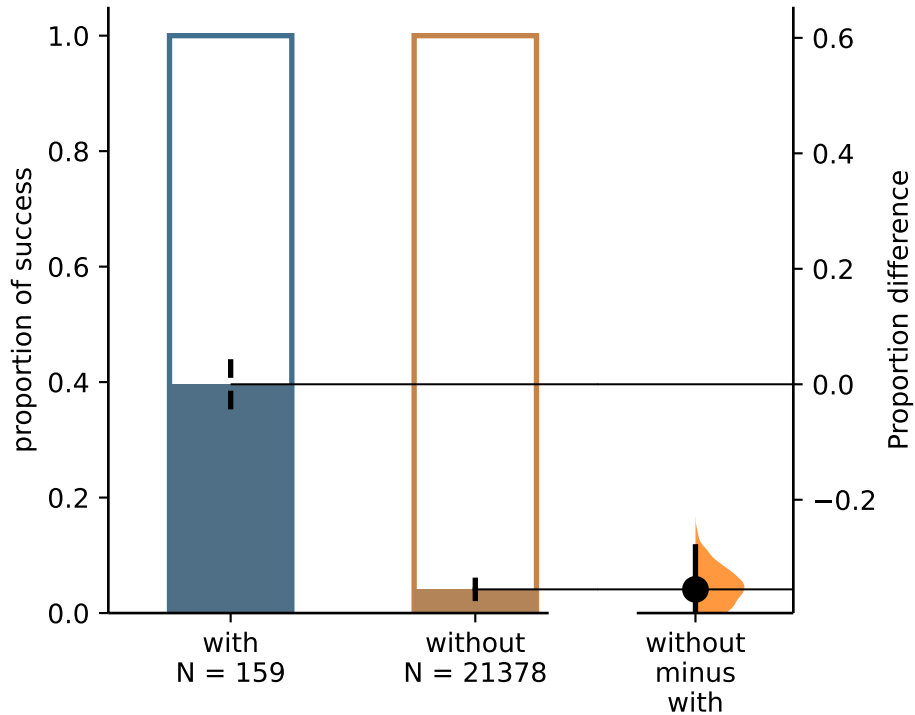

Supplement: SC-016-D5SC01100K-s001 [file SC-016-D5SC01100K-s001.zip › ESI/si_images/structural_analysis/oer_gpt_tp15_mean_diff.pdf]

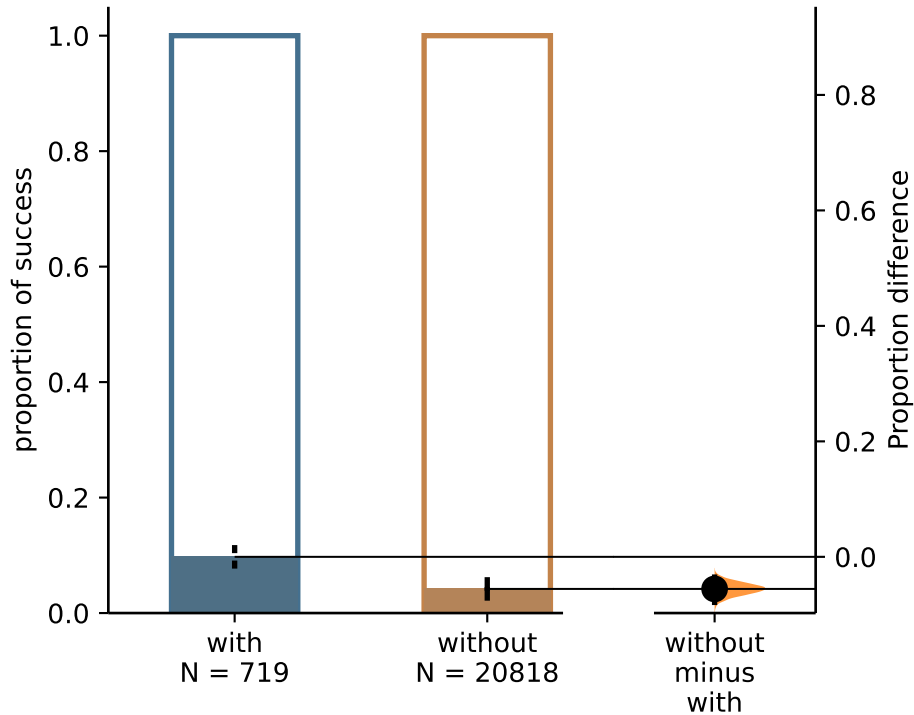

Supplement: SC-016-D5SC01100K-s001 [file SC-016-D5SC01100K-s001.zip › ESI/si_images/structural_analysis/oer_gpt_tp20_mean_diff.pdf]

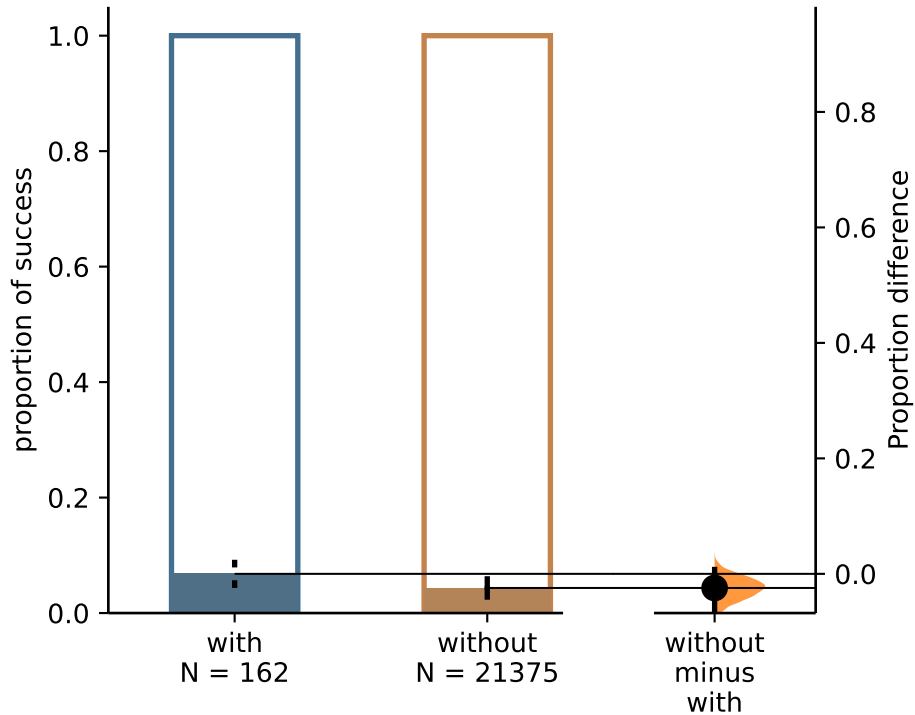

Supplement: SC-016-D5SC01100K-s001 [file SC-016-D5SC01100K-s001.zip › ESI/si_images/structural_analysis/oer_gpt_tp8_mean_diff.pdf]

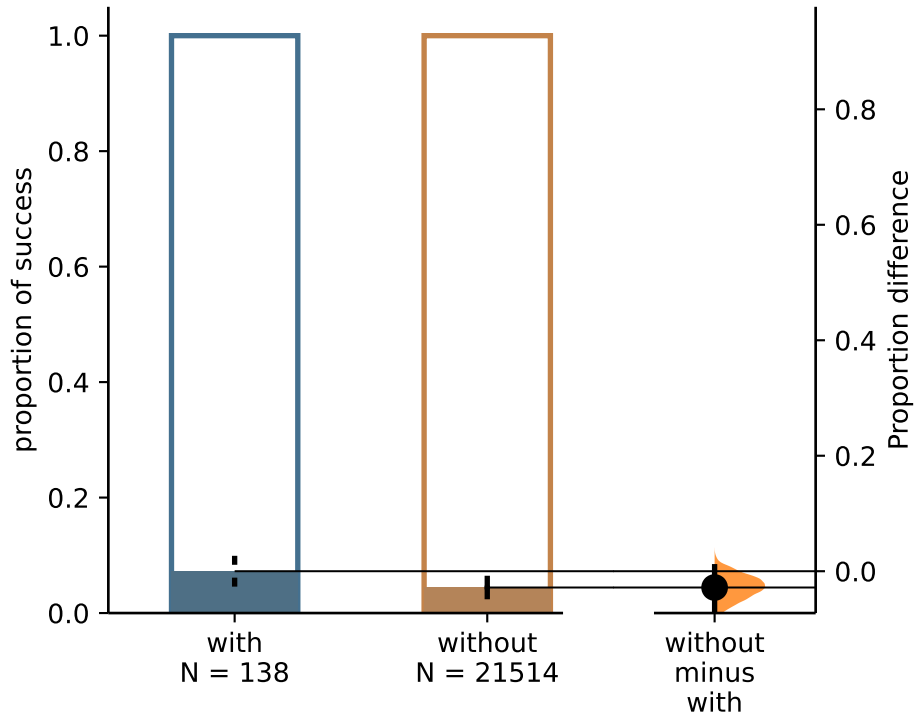

Supplement: SC-016-D5SC01100K-s001 [file SC-016-D5SC01100K-s001.zip › ESI/si_images/structural_analysis/oer_moft_ol17_mean_diff.pdf]

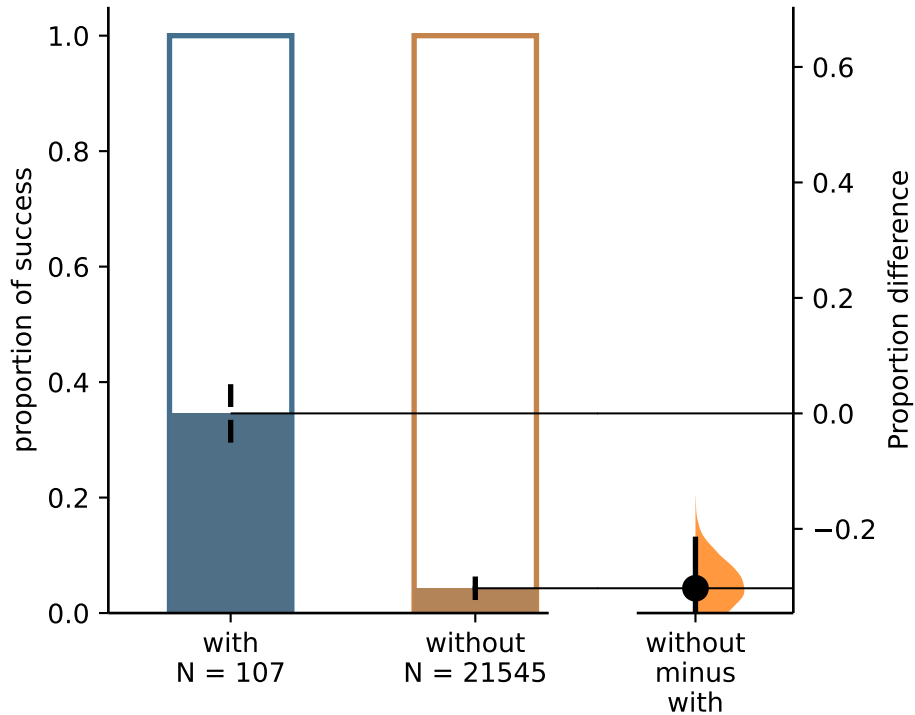

Supplement: SC-016-D5SC01100K-s001 [file SC-016-D5SC01100K-s001.zip › ESI/si_images/structural_analysis/oer_moft_ol1_mean_diff.pdf]

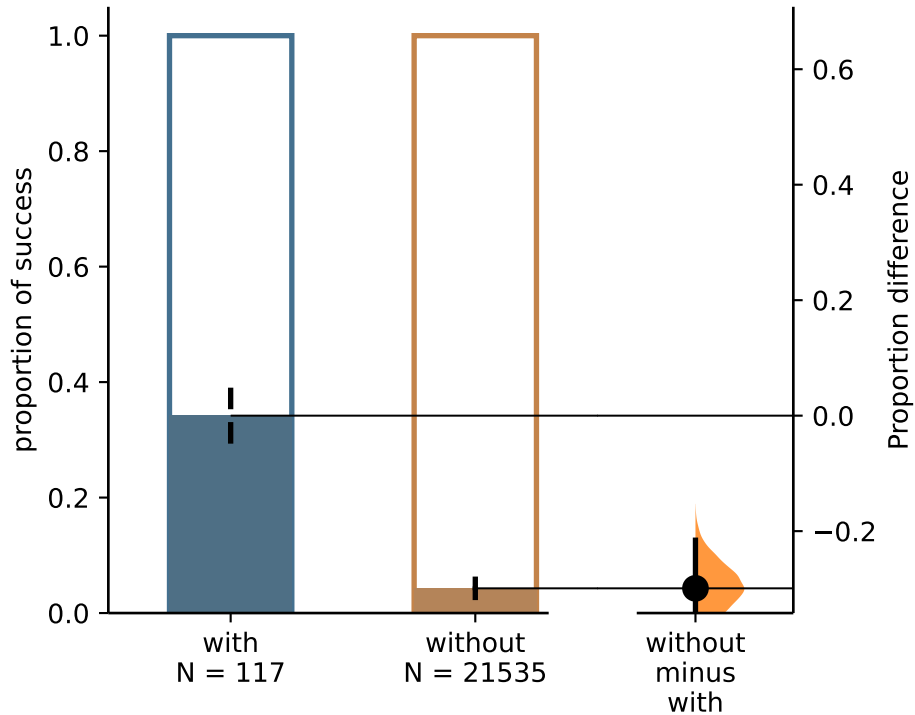

Supplement: SC-016-D5SC01100K-s001 [file SC-016-D5SC01100K-s001.zip › ESI/si_images/structural_analysis/oer_moft_ol20_mean_diff.pdf]

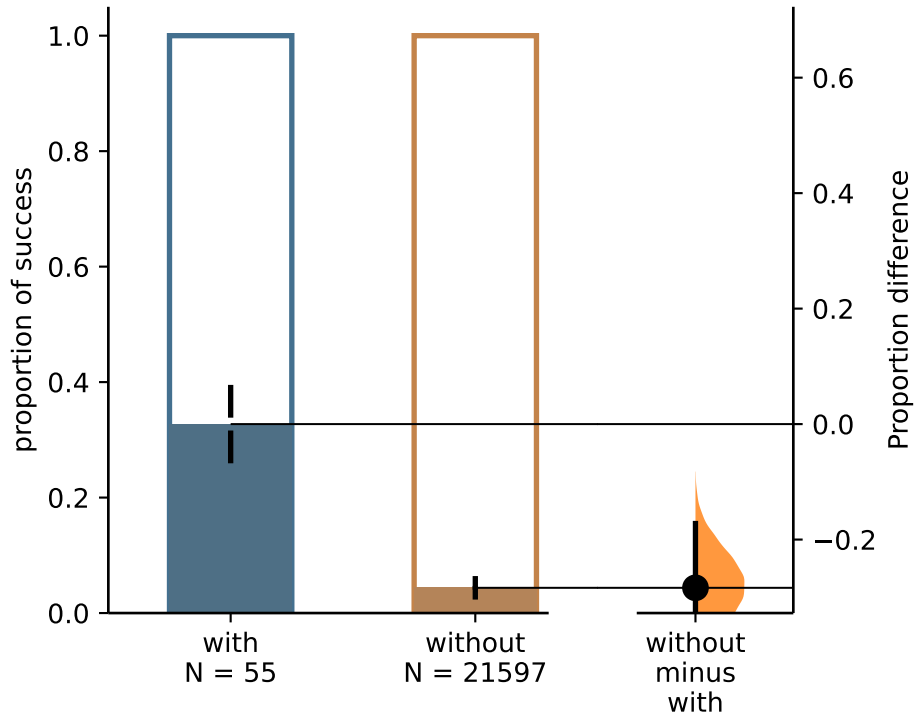

Supplement: SC-016-D5SC01100K-s001 [file SC-016-D5SC01100K-s001.zip › ESI/si_images/structural_analysis/oer_moft_ol30_mean_diff.pdf]

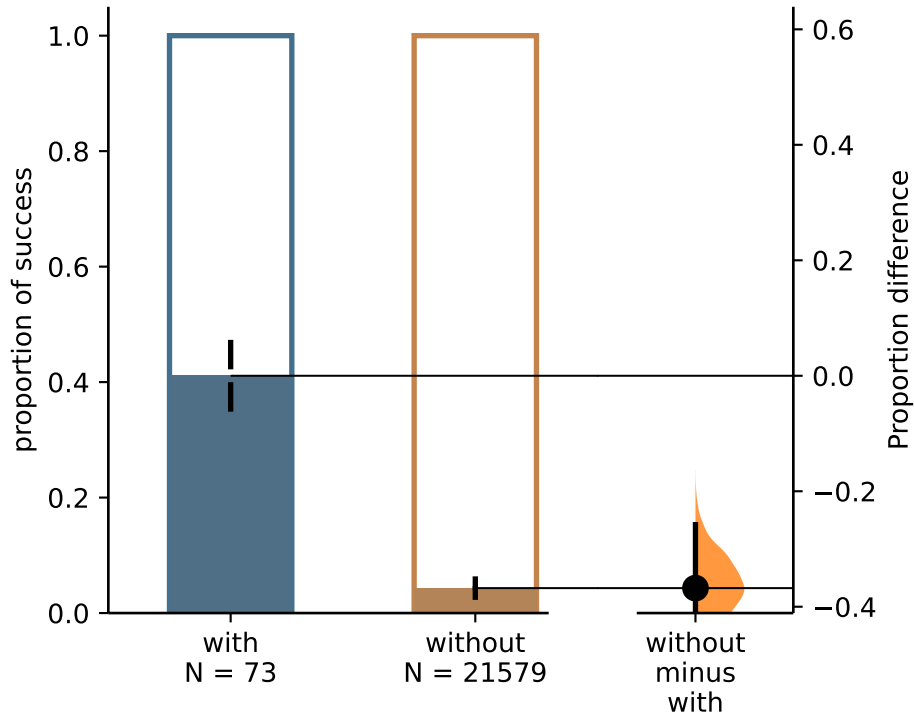

Supplement: SC-016-D5SC01100K-s001 [file SC-016-D5SC01100K-s001.zip › ESI/si_images/structural_analysis/oer_moft_ol5_mean_diff.pdf]

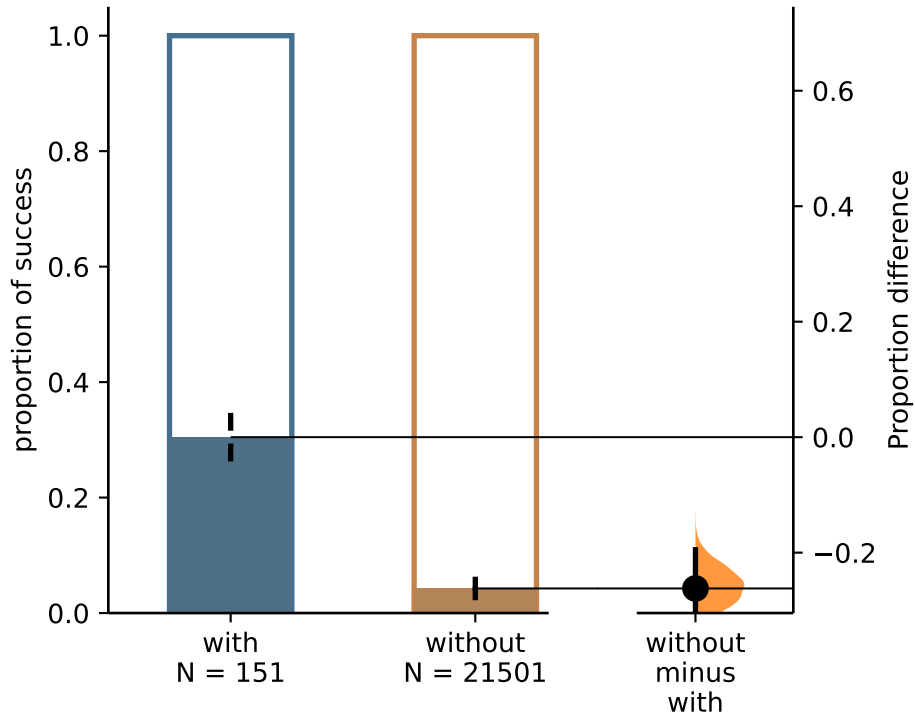

Supplement: SC-016-D5SC01100K-s001 [file SC-016-D5SC01100K-s001.zip › ESI/si_images/structural_analysis/oer_moft_ol78_mean_diff.pdf]

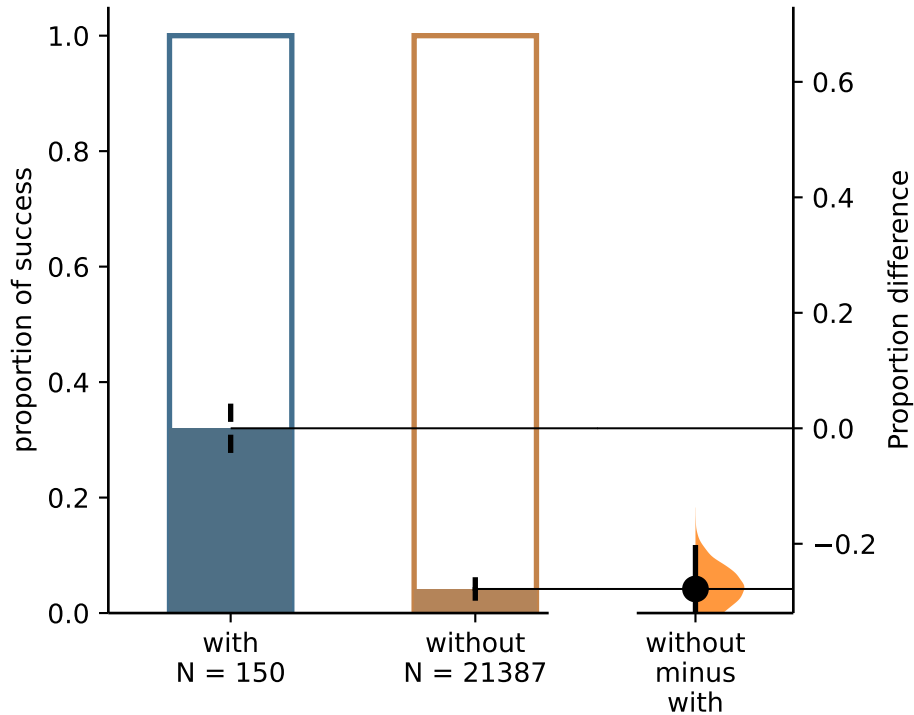

Supplement: SC-016-D5SC01100K-s001 [file SC-016-D5SC01100K-s001.zip › ESI/si_images/structural_analysis/oer_moft_tp15_mean_diff.pdf]

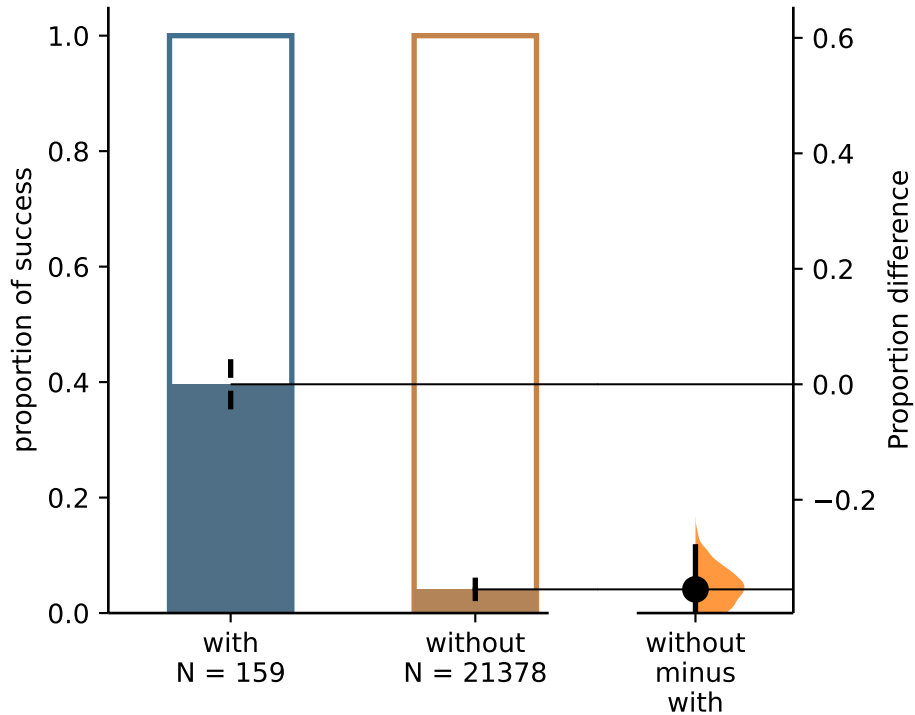

Supplement: SC-016-D5SC01100K-s001 [file SC-016-D5SC01100K-s001.zip › ESI/si_images/structural_analysis/oer_moft_tp20_mean_diff.pdf]

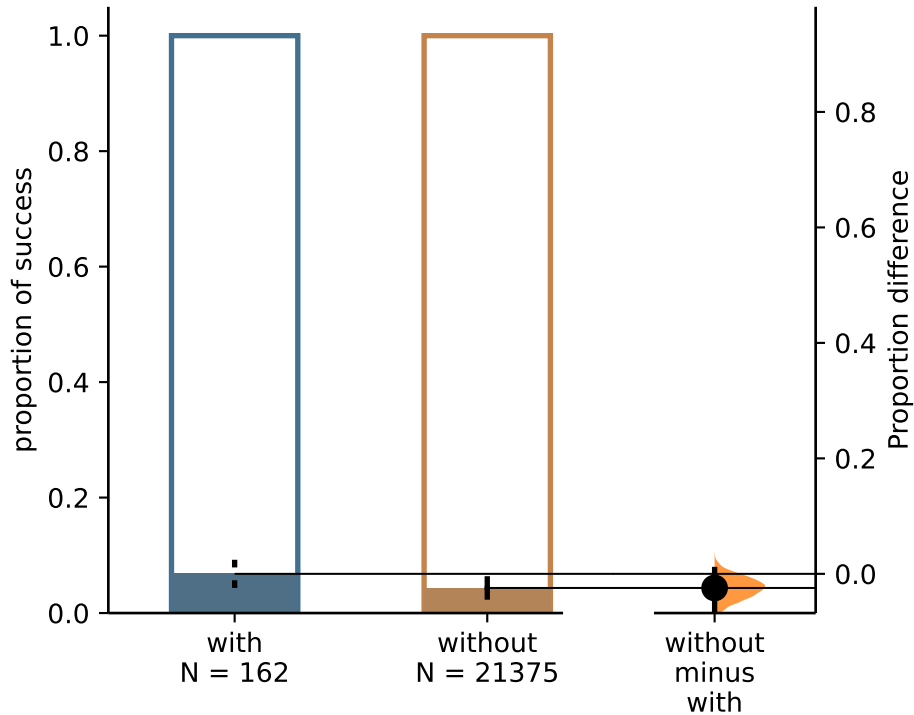

Supplement: SC-016-D5SC01100K-s001 [file SC-016-D5SC01100K-s001.zip › ESI/si_images/structural_analysis/oer_moft_tp8_mean_diff.pdf]

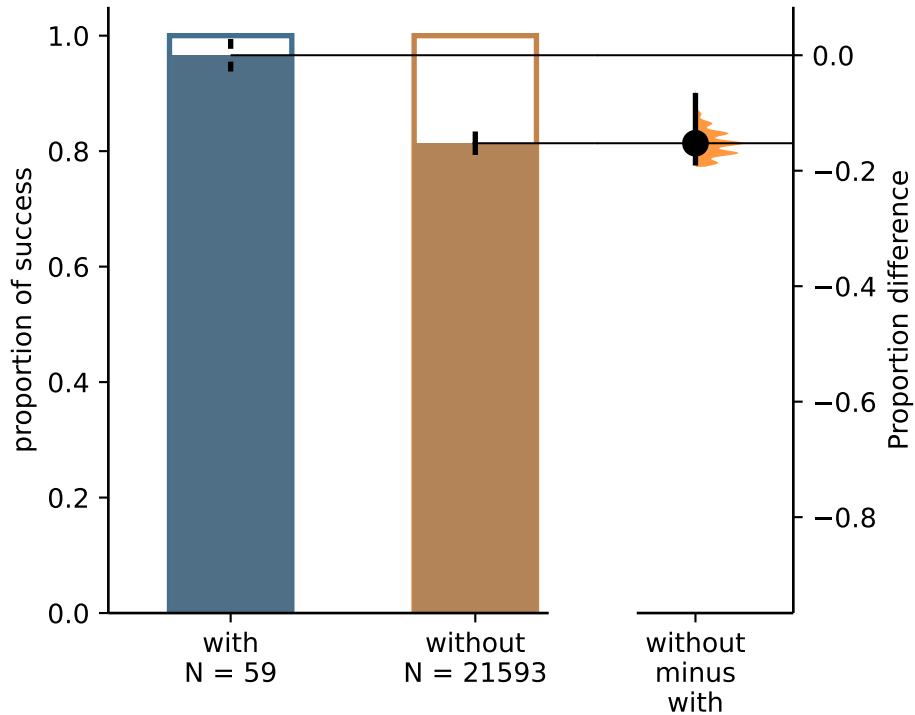

Supplement: SC-016-D5SC01100K-s001 [file SC-016-D5SC01100K-s001.zip › ESI/si_images/structural_analysis/ovlp_gpt_mn29_mean_diff.pdf]

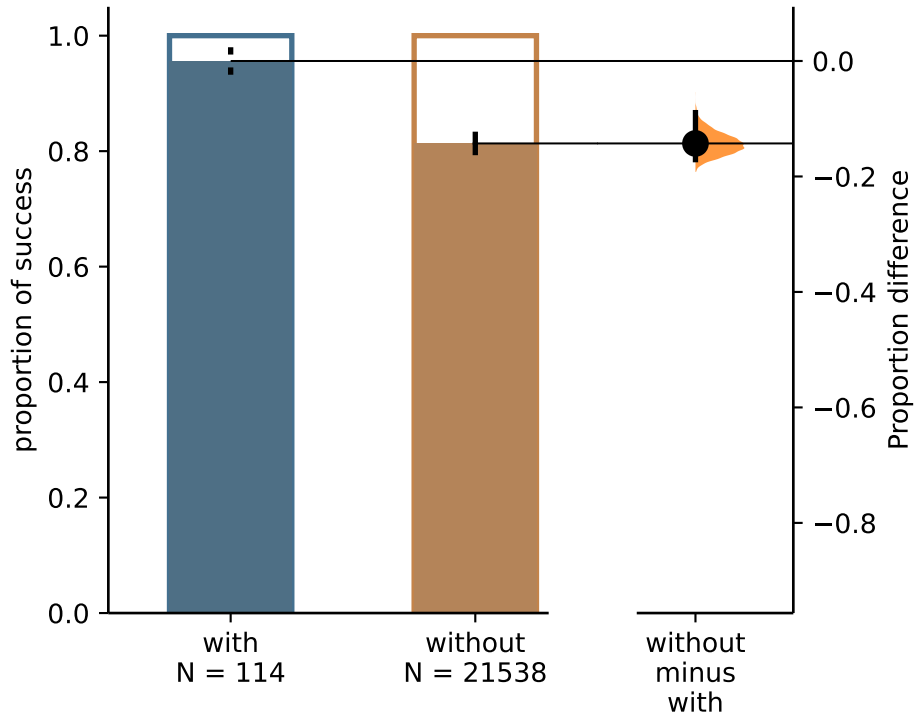

Supplement: SC-016-D5SC01100K-s001 [file SC-016-D5SC01100K-s001.zip › ESI/si_images/structural_analysis/ovlp_gpt_mn30_mean_diff.pdf]

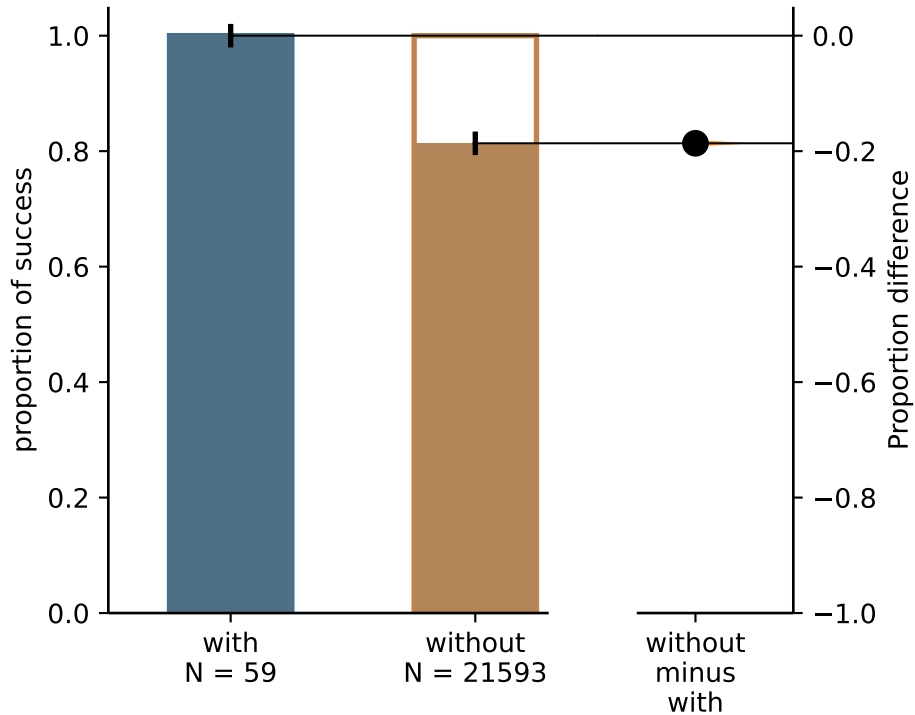

Supplement: SC-016-D5SC01100K-s001 [file SC-016-D5SC01100K-s001.zip › ESI/si_images/structural_analysis/ovlp_gpt_mn52_mean_diff.pdf]

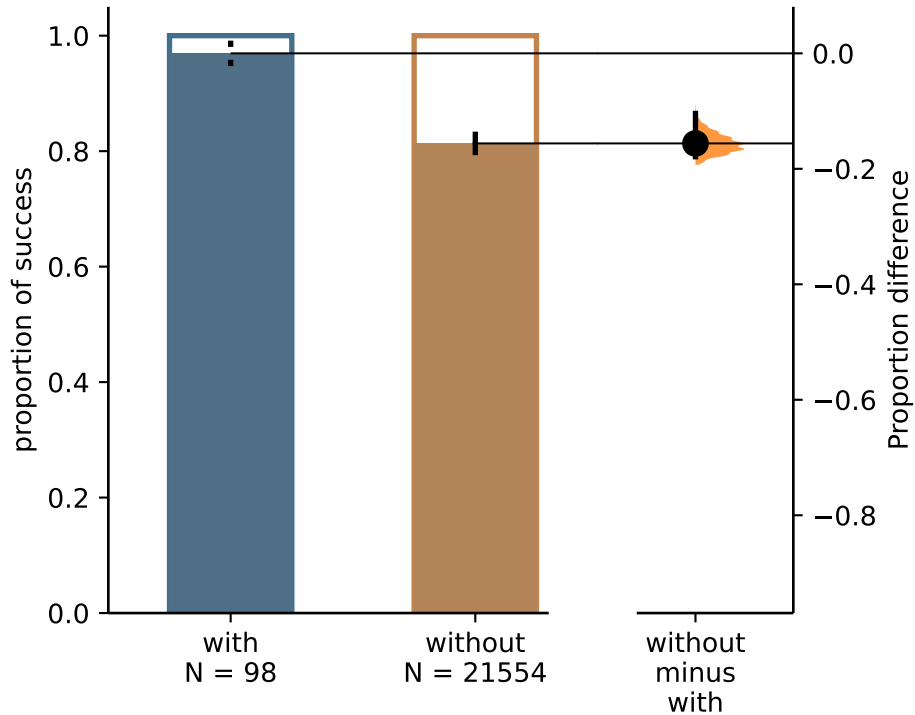

Supplement: SC-016-D5SC01100K-s001 [file SC-016-D5SC01100K-s001.zip › ESI/si_images/structural_analysis/ovlp_gpt_ol21_mean_diff.pdf]

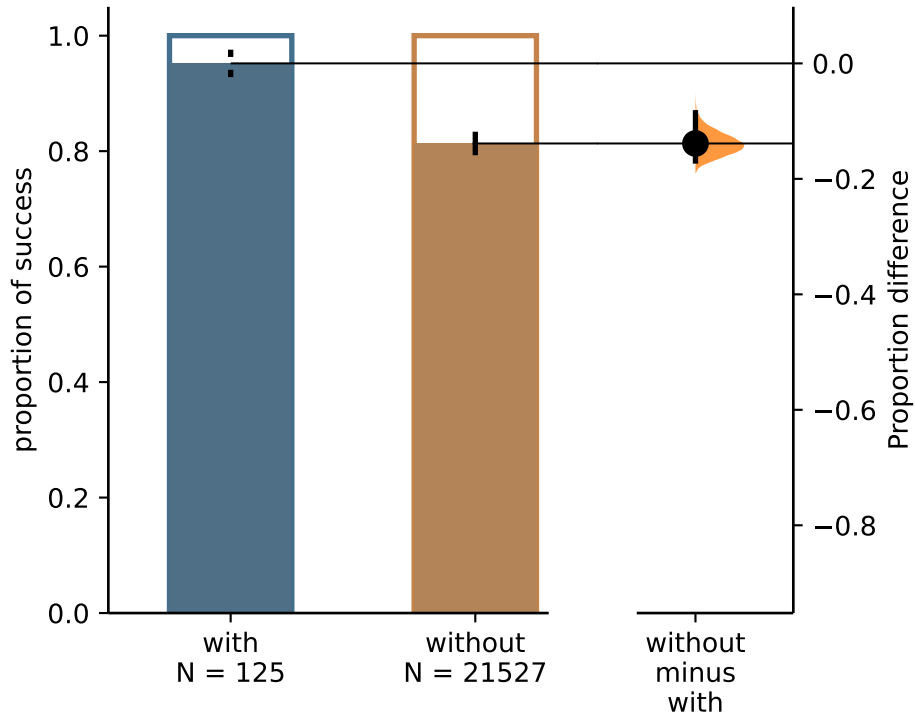

Supplement: SC-016-D5SC01100K-s001 [file SC-016-D5SC01100K-s001.zip › ESI/si_images/structural_analysis/ovlp_gpt_ol46_mean_diff.pdf]

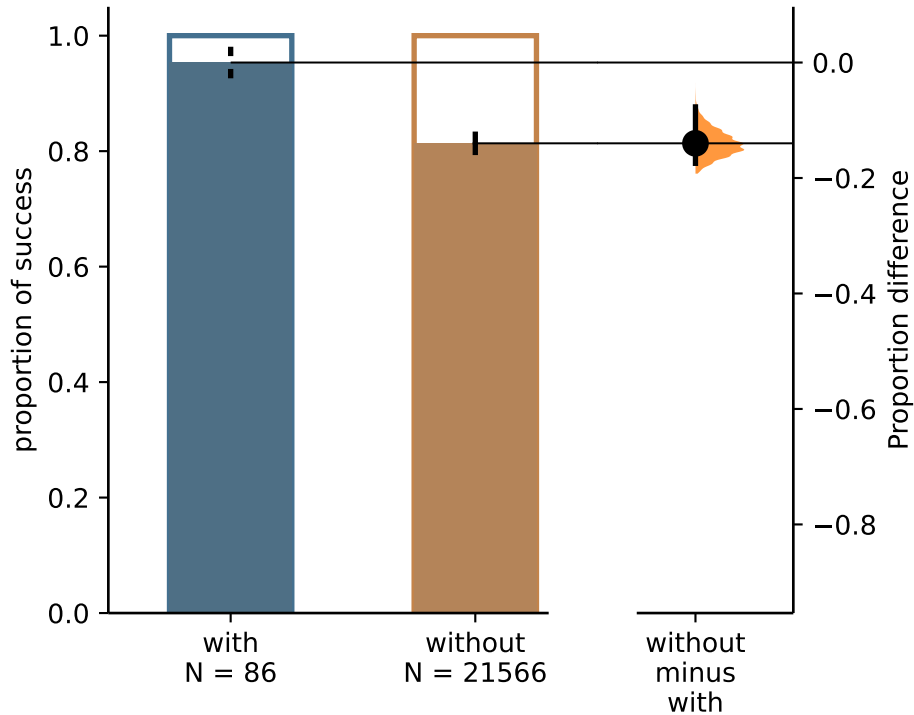

Supplement: SC-016-D5SC01100K-s001 [file SC-016-D5SC01100K-s001.zip › ESI/si_images/structural_analysis/ovlp_gpt_ol47_mean_diff.pdf]

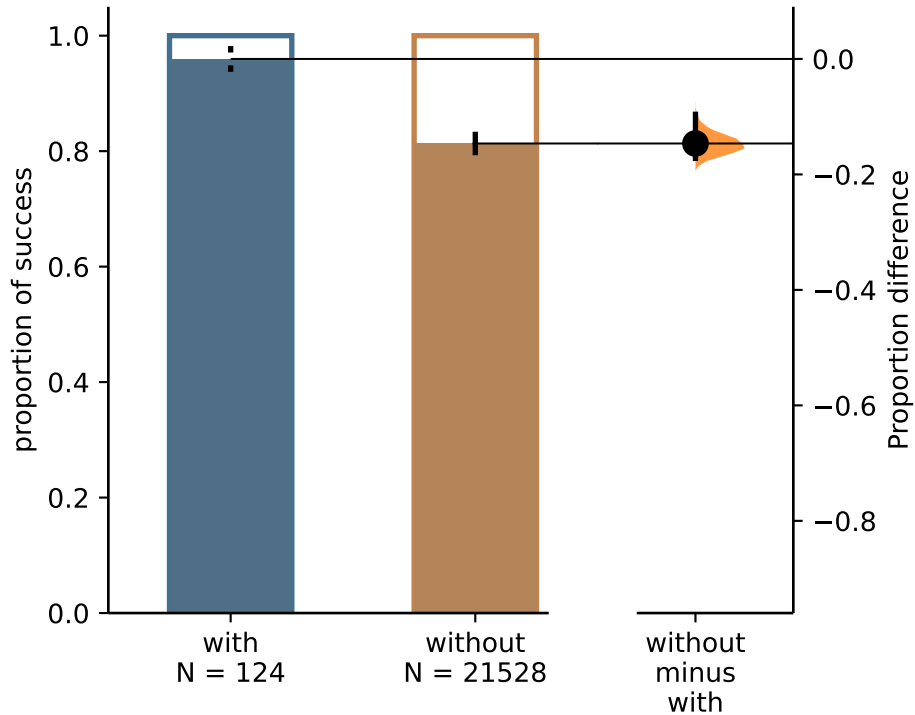

Supplement: SC-016-D5SC01100K-s001 [file SC-016-D5SC01100K-s001.zip › ESI/si_images/structural_analysis/ovlp_gpt_ol67_mean_diff.pdf]

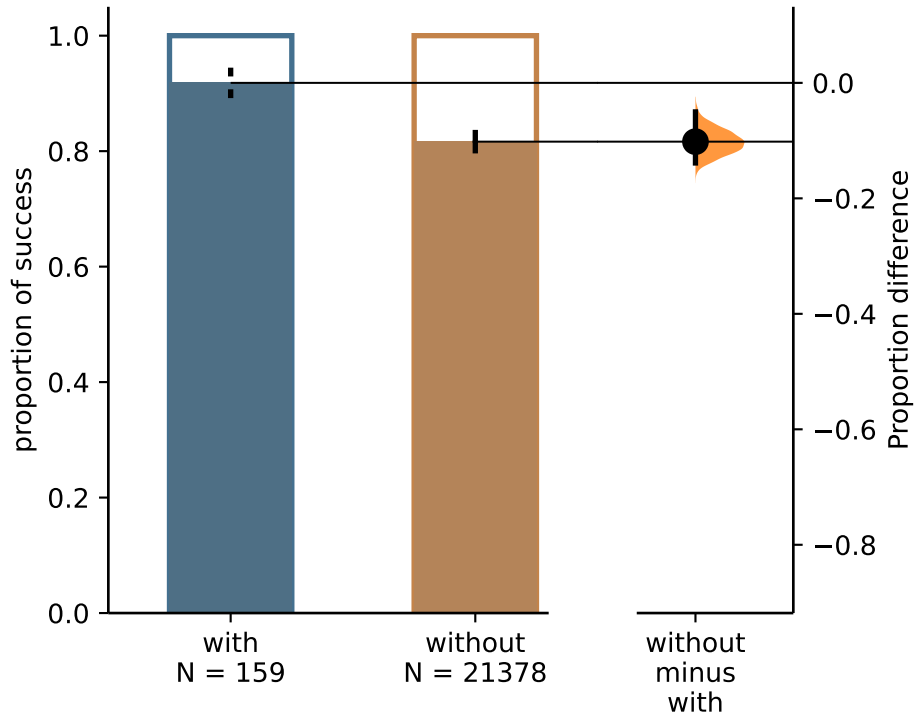

Supplement: SC-016-D5SC01100K-s001 [file SC-016-D5SC01100K-s001.zip › ESI/si_images/structural_analysis/ovlp_gpt_tp15_mean_diff.pdf]

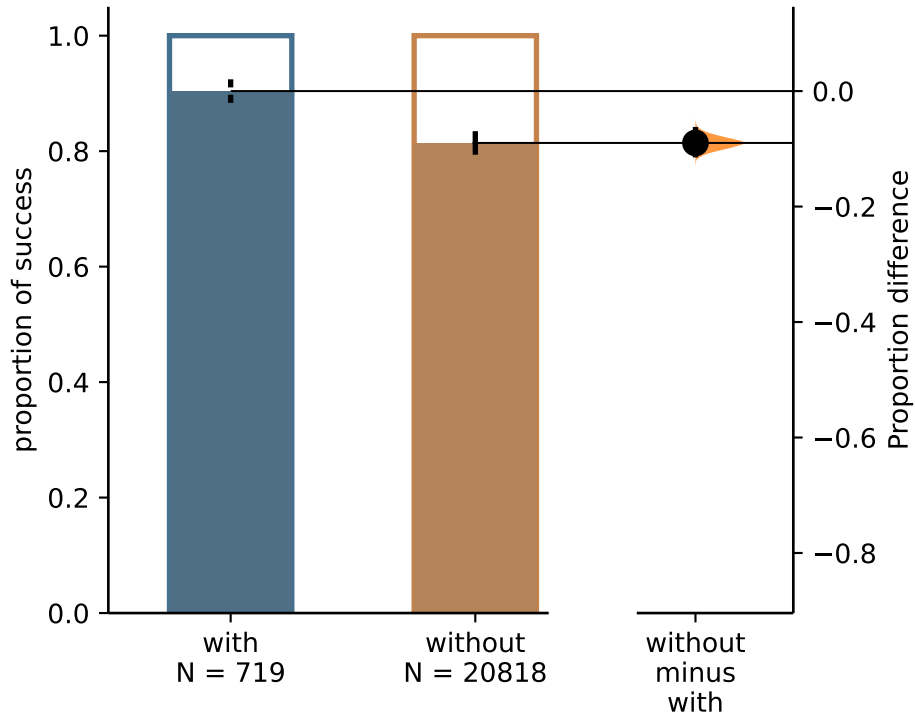

Supplement: SC-016-D5SC01100K-s001 [file SC-016-D5SC01100K-s001.zip › ESI/si_images/structural_analysis/ovlp_gpt_tp20_mean_diff.pdf]

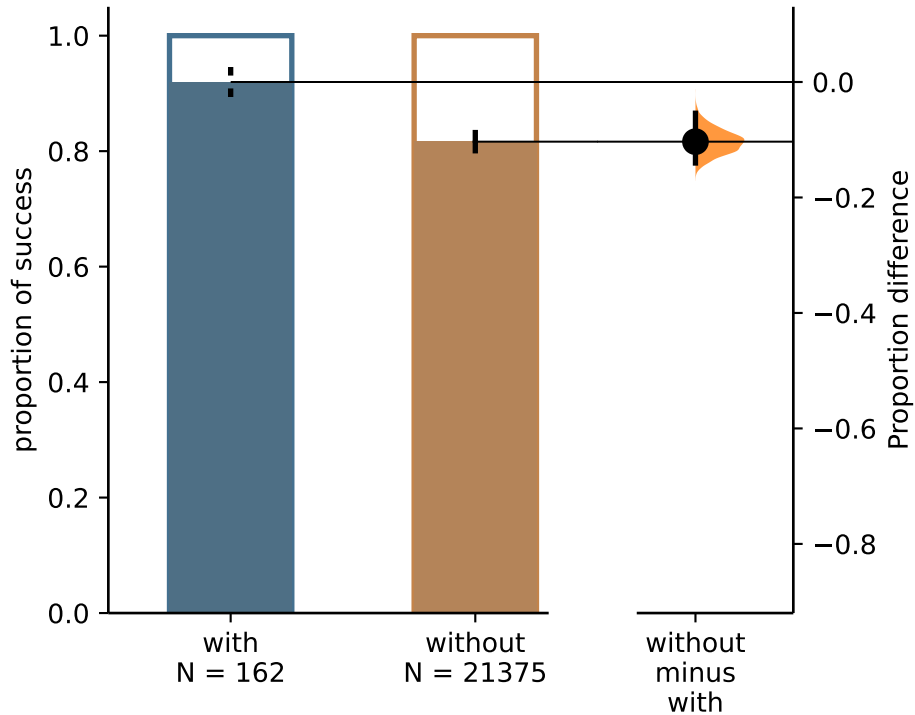

Supplement: SC-016-D5SC01100K-s001 [file SC-016-D5SC01100K-s001.zip › ESI/si_images/structural_analysis/ovlp_gpt_tp8_mean_diff.pdf]

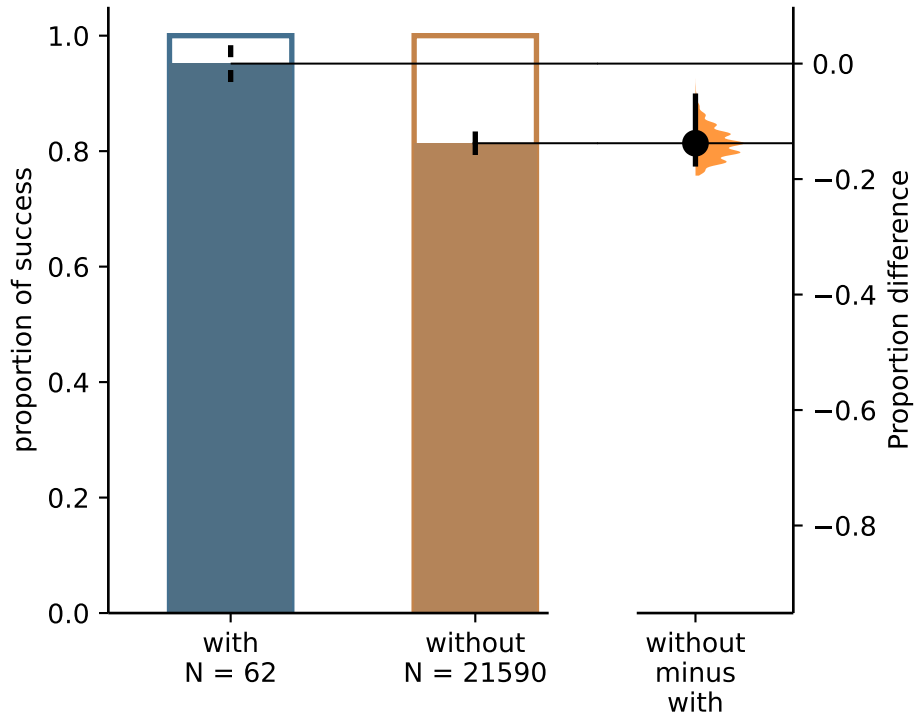

Supplement: SC-016-D5SC01100K-s001 [file SC-016-D5SC01100K-s001.zip › ESI/si_images/structural_analysis/ovlp_moft_mn29_mean_diff.pdf]

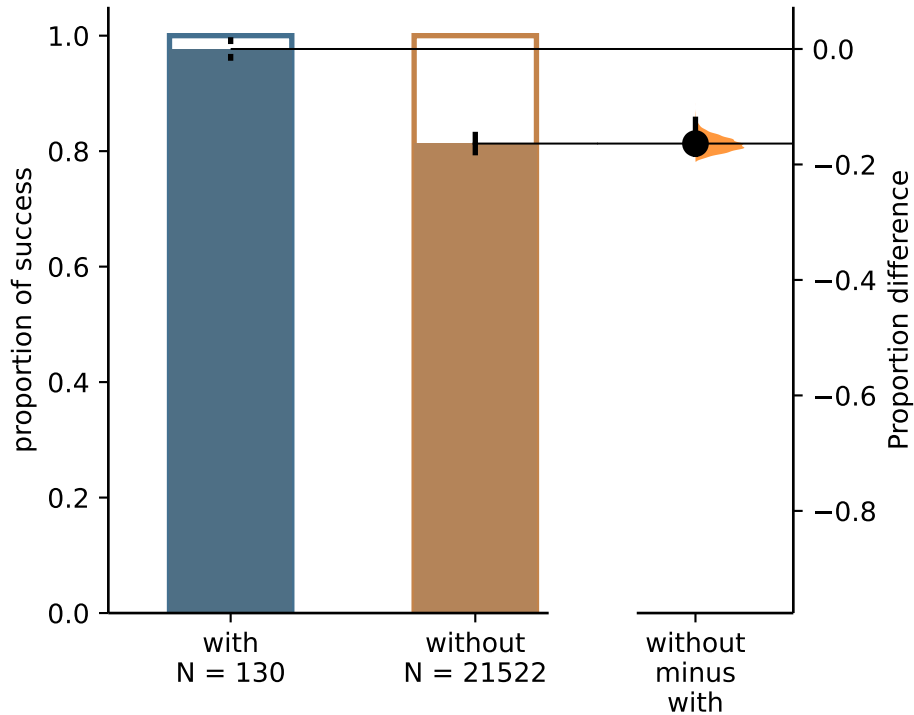

Supplement: SC-016-D5SC01100K-s001 [file SC-016-D5SC01100K-s001.zip › ESI/si_images/structural_analysis/ovlp_moft_mn30_mean_diff.pdf]

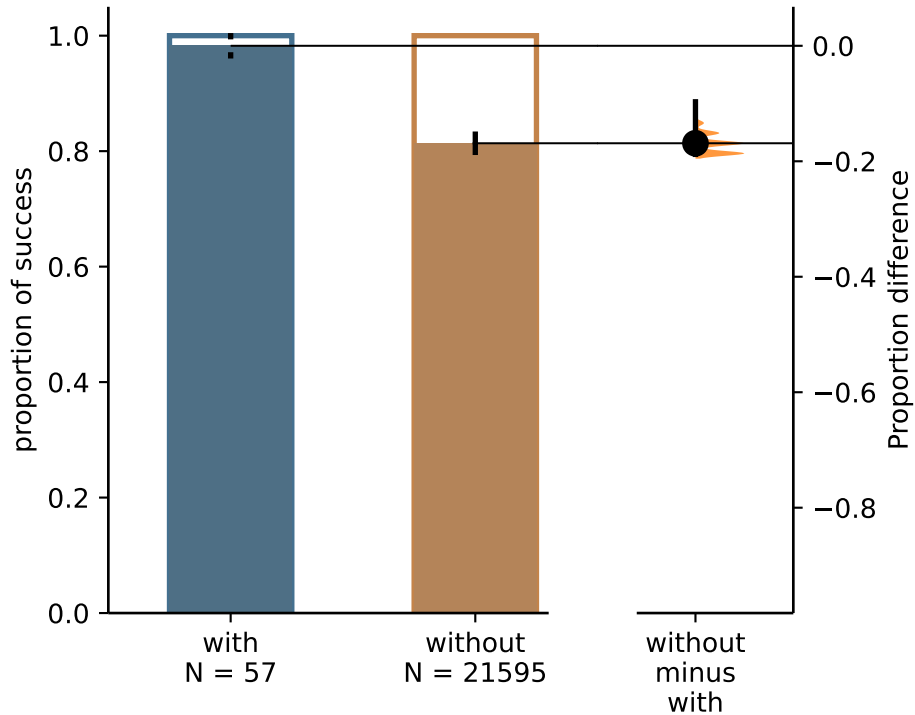

Supplement: SC-016-D5SC01100K-s001 [file SC-016-D5SC01100K-s001.zip › ESI/si_images/structural_analysis/ovlp_moft_mn52_mean_diff.pdf]

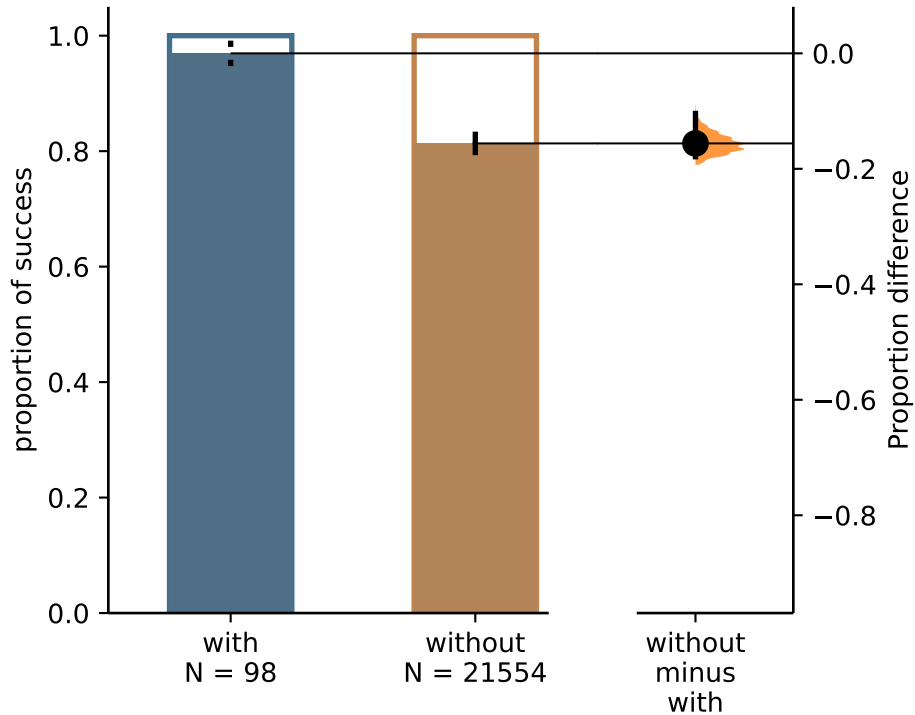

Supplement: SC-016-D5SC01100K-s001 [file SC-016-D5SC01100K-s001.zip › ESI/si_images/structural_analysis/ovlp_moft_ol21_mean_diff.pdf]

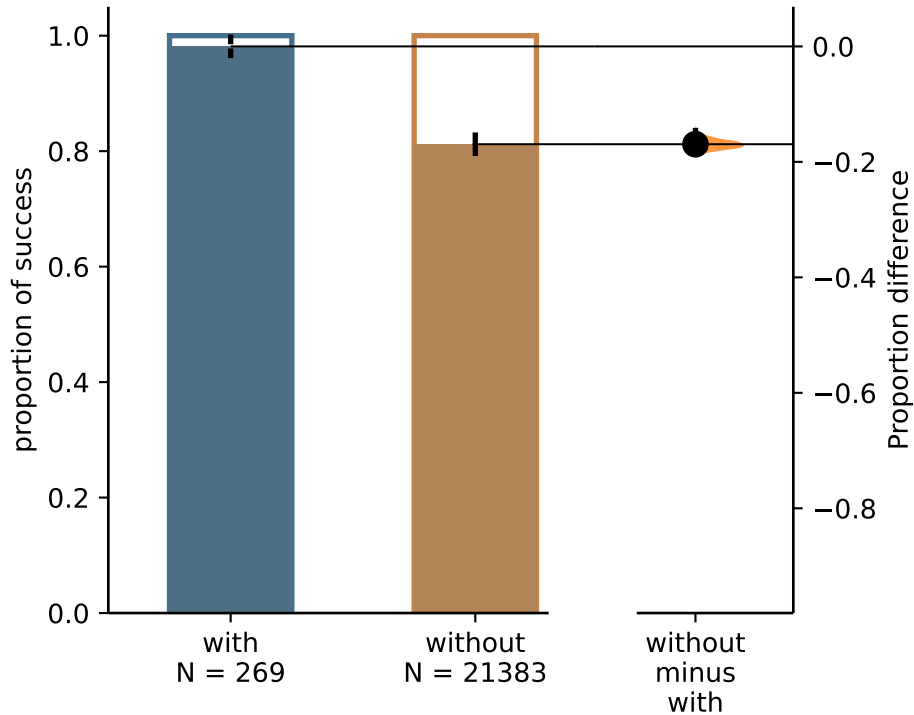

Supplement: SC-016-D5SC01100K-s001 [file SC-016-D5SC01100K-s001.zip › ESI/si_images/structural_analysis/ovlp_moft_ol46_mean_diff.pdf]

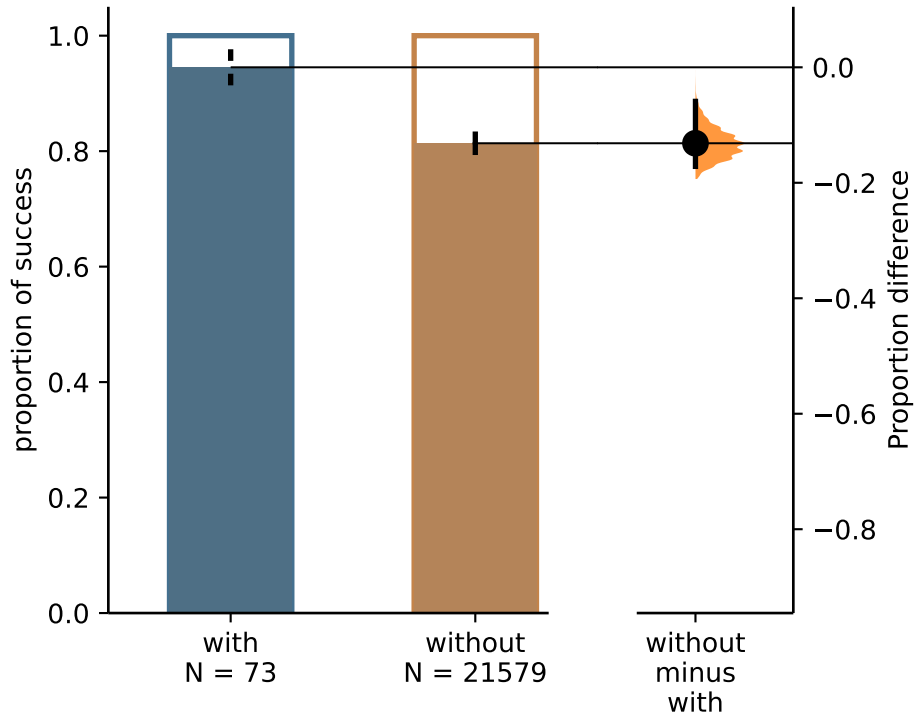

Supplement: SC-016-D5SC01100K-s001 [file SC-016-D5SC01100K-s001.zip › ESI/si_images/structural_analysis/ovlp_moft_ol47_mean_diff.pdf]

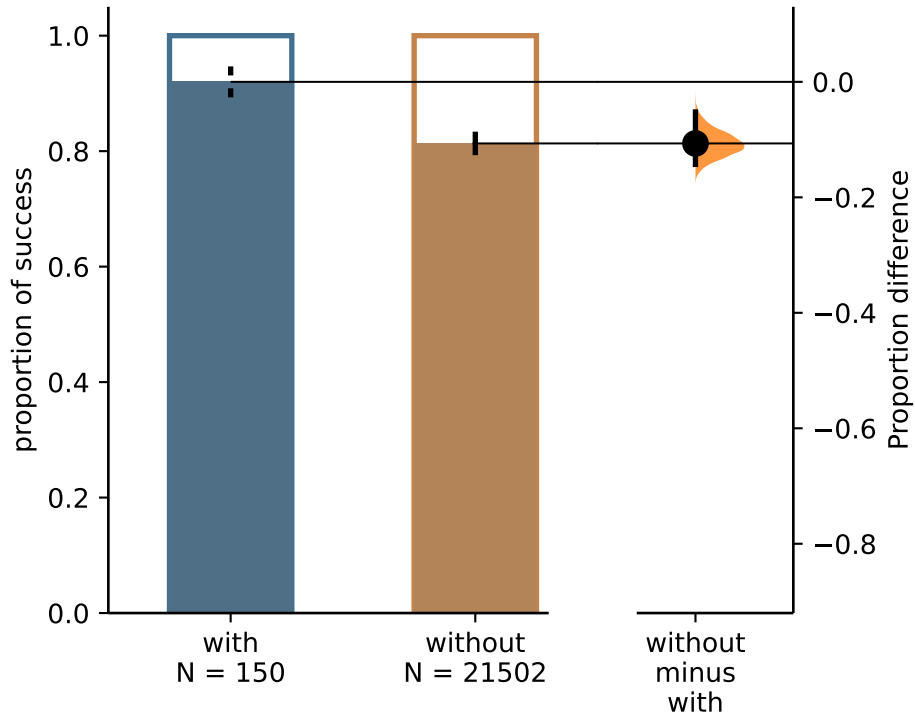

Supplement: SC-016-D5SC01100K-s001 [file SC-016-D5SC01100K-s001.zip › ESI/si_images/structural_analysis/ovlp_moft_ol67_mean_diff.pdf]

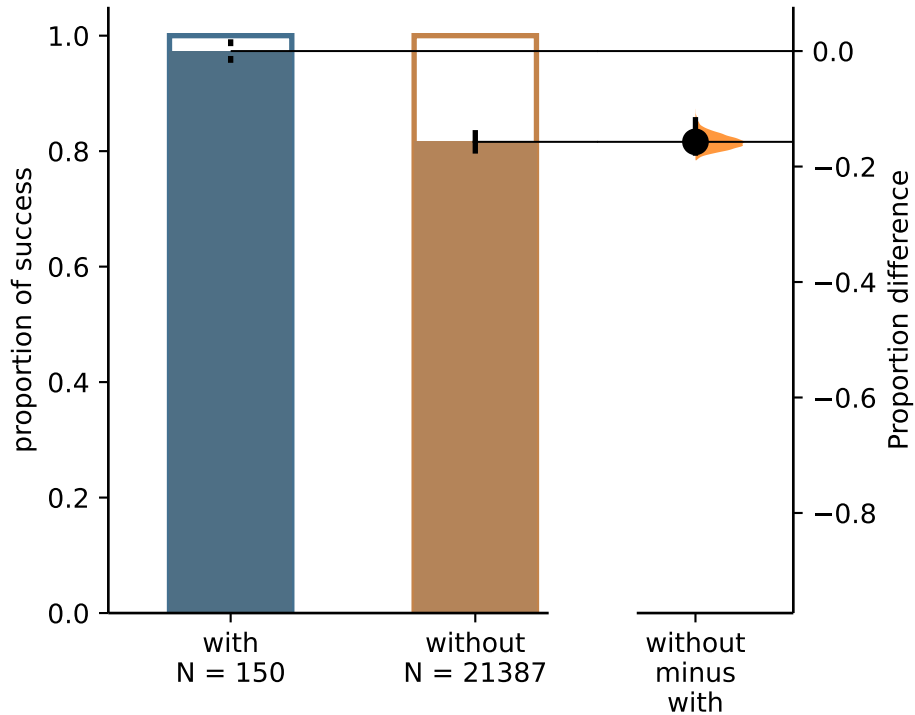

Supplement: SC-016-D5SC01100K-s001 [file SC-016-D5SC01100K-s001.zip › ESI/si_images/structural_analysis/ovlp_moft_tp15_mean_diff.pdf]

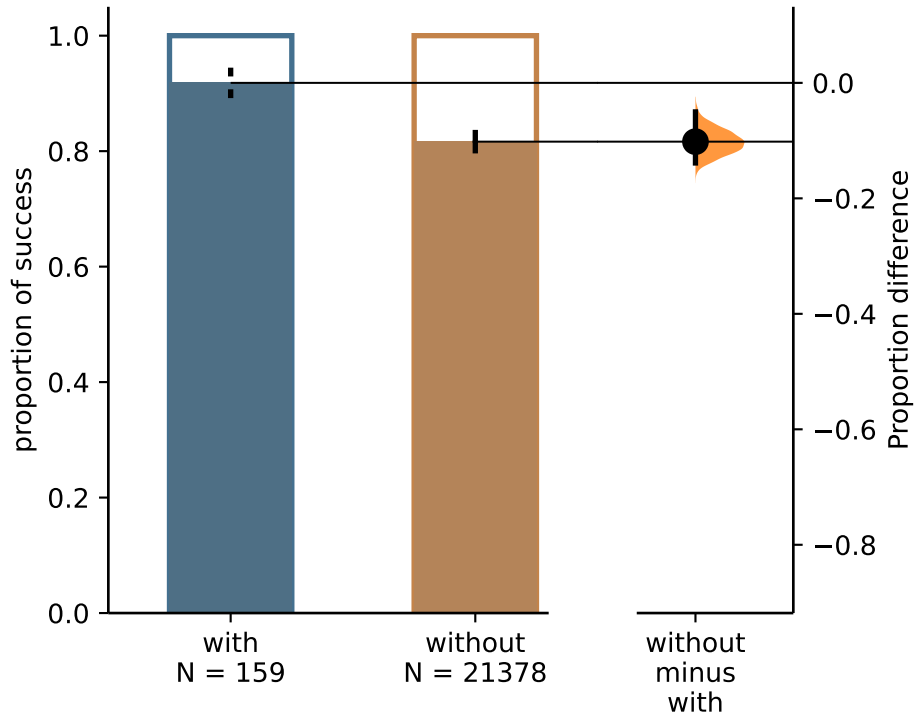

Supplement: SC-016-D5SC01100K-s001 [file SC-016-D5SC01100K-s001.zip › ESI/si_images/structural_analysis/ovlp_moft_tp20_mean_diff.pdf]

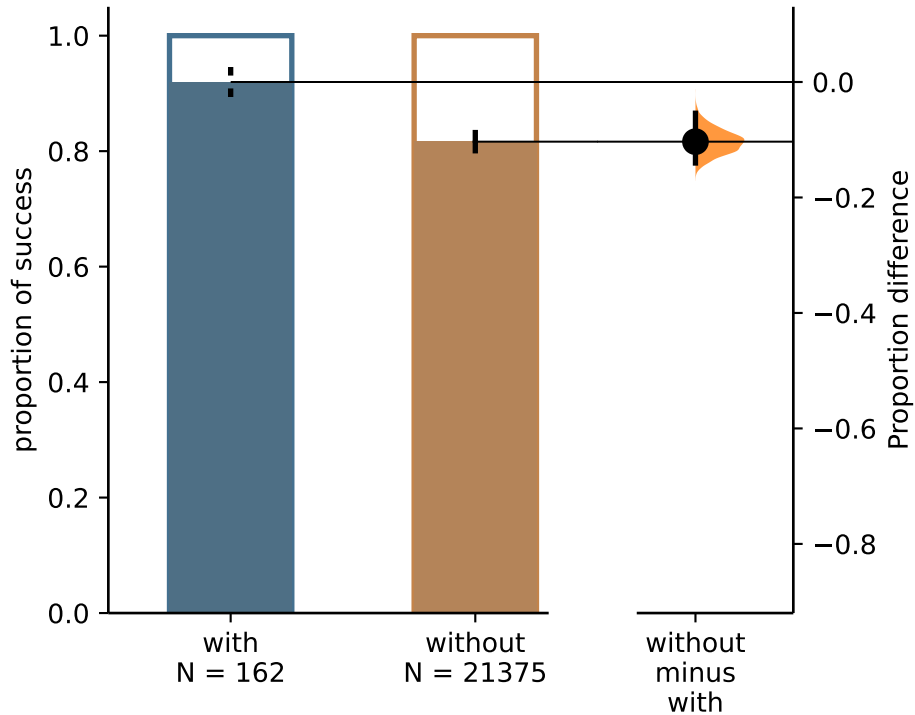

Supplement: SC-016-D5SC01100K-s001 [file SC-016-D5SC01100K-s001.zip › ESI/si_images/structural_analysis/ovlp_moft_tp8_mean_diff.pdf]

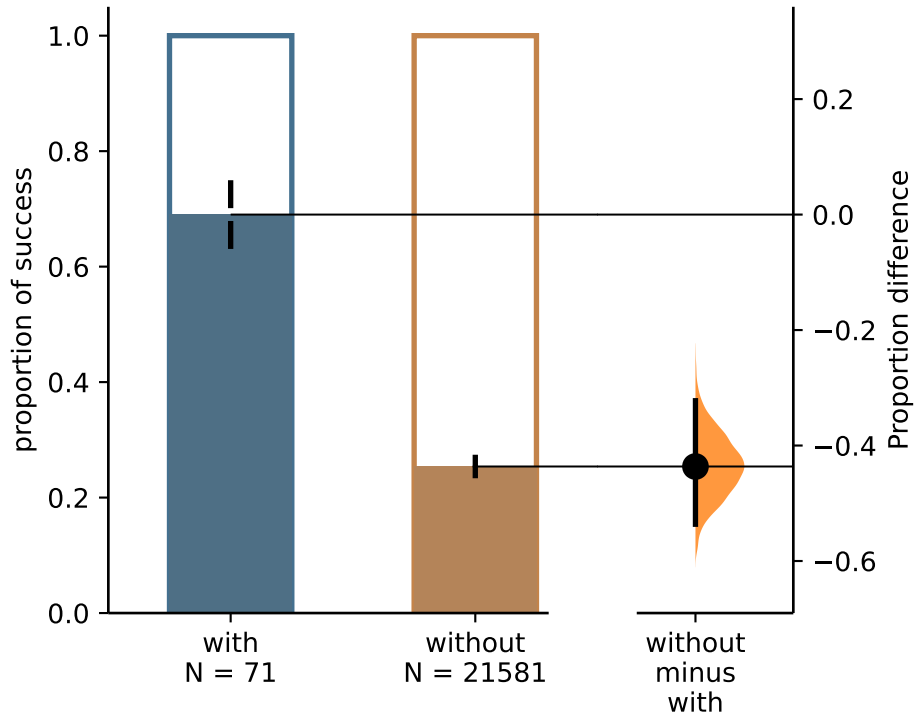

Supplement: SC-016-D5SC01100K-s001 [file SC-016-D5SC01100K-s001.zip › ESI/si_images/structural_analysis/vis_gpt_ol1_mean_diff.pdf]

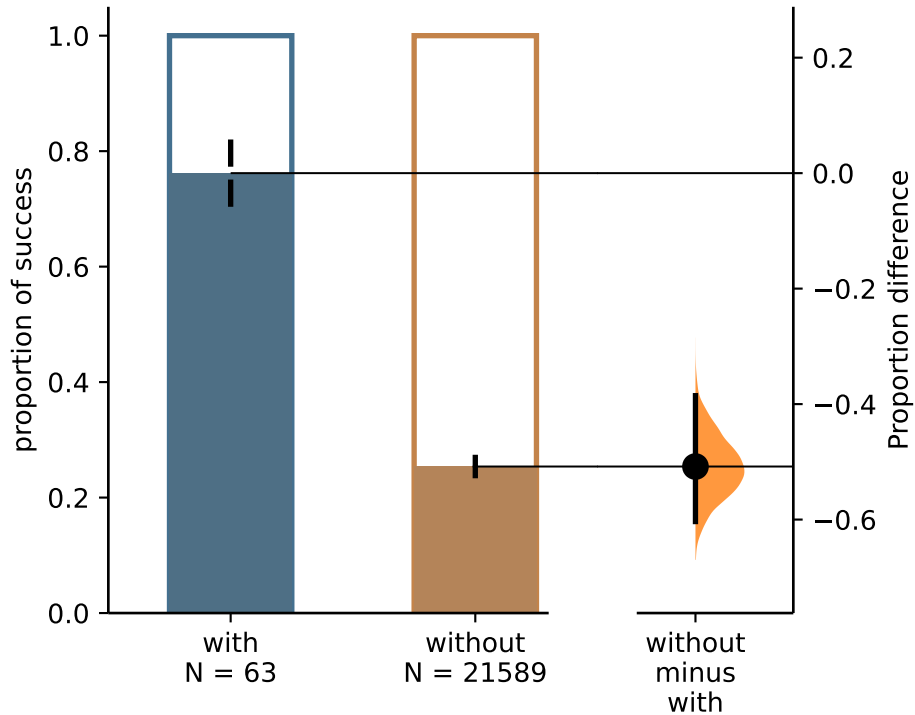

Supplement: SC-016-D5SC01100K-s001 [file SC-016-D5SC01100K-s001.zip › ESI/si_images/structural_analysis/vis_gpt_ol30_mean_diff.pdf]

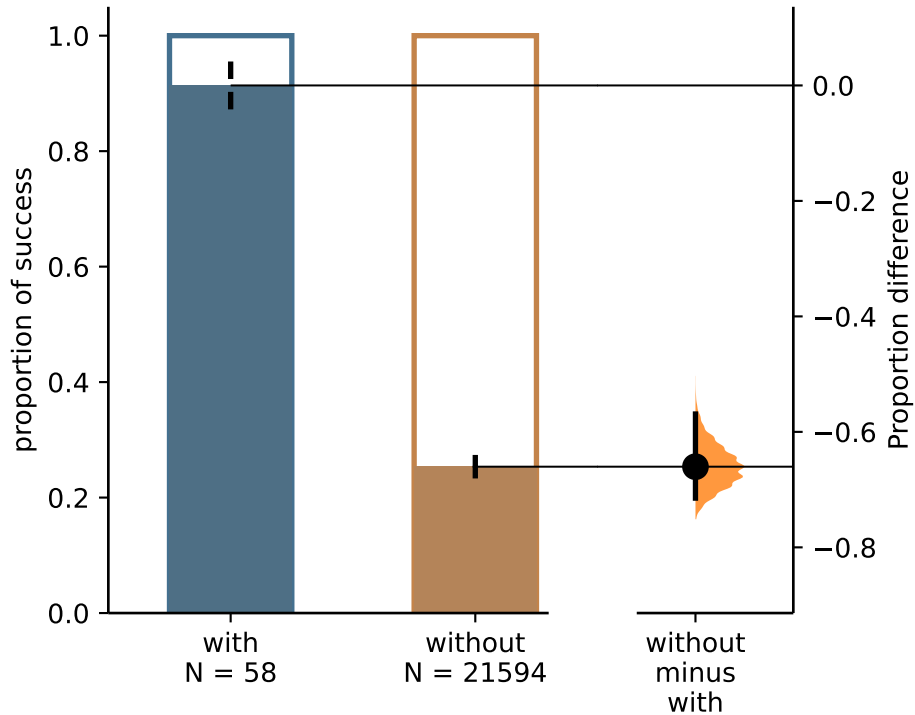

Supplement: SC-016-D5SC01100K-s001 [file SC-016-D5SC01100K-s001.zip › ESI/si_images/structural_analysis/vis_gpt_ol40_mean_diff.pdf]

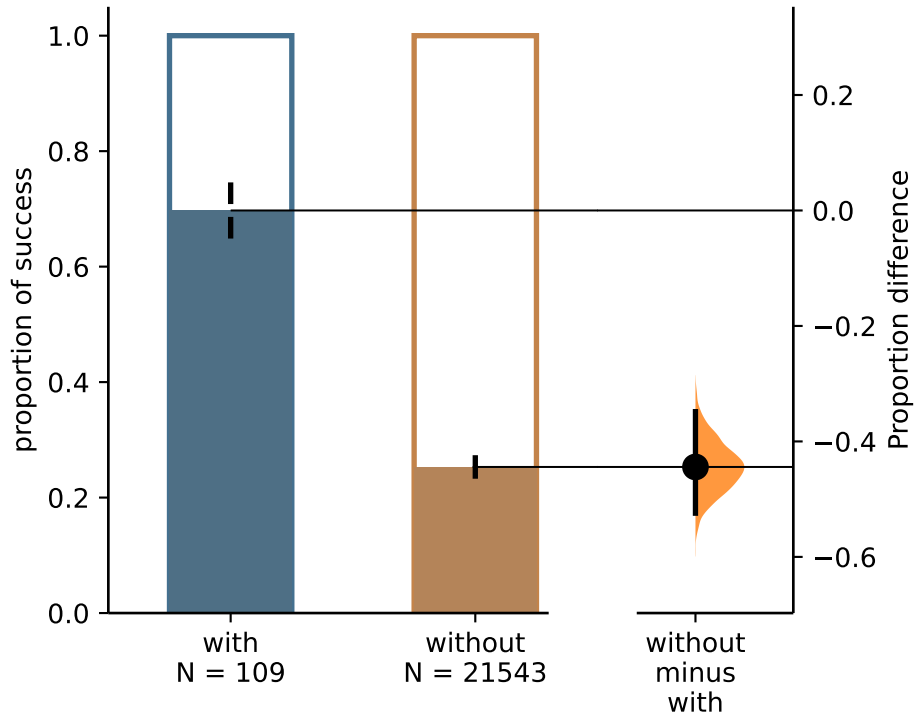

Supplement: SC-016-D5SC01100K-s001 [file SC-016-D5SC01100K-s001.zip › ESI/si_images/structural_analysis/vis_gpt_ol5_mean_diff.pdf]

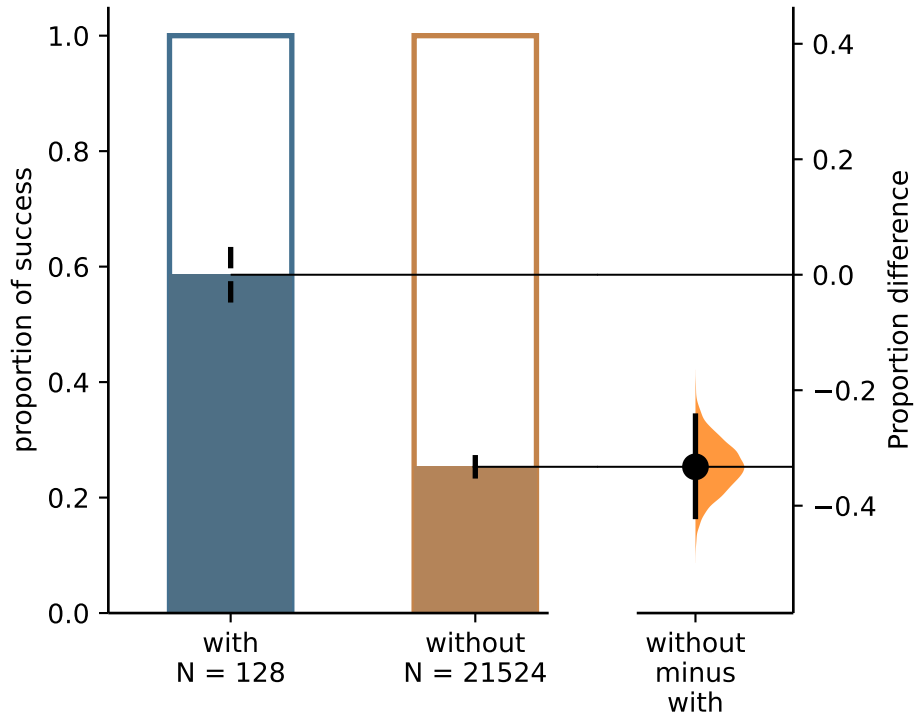

Supplement: SC-016-D5SC01100K-s001 [file SC-016-D5SC01100K-s001.zip › ESI/si_images/structural_analysis/vis_gpt_ol68_mean_diff.pdf]

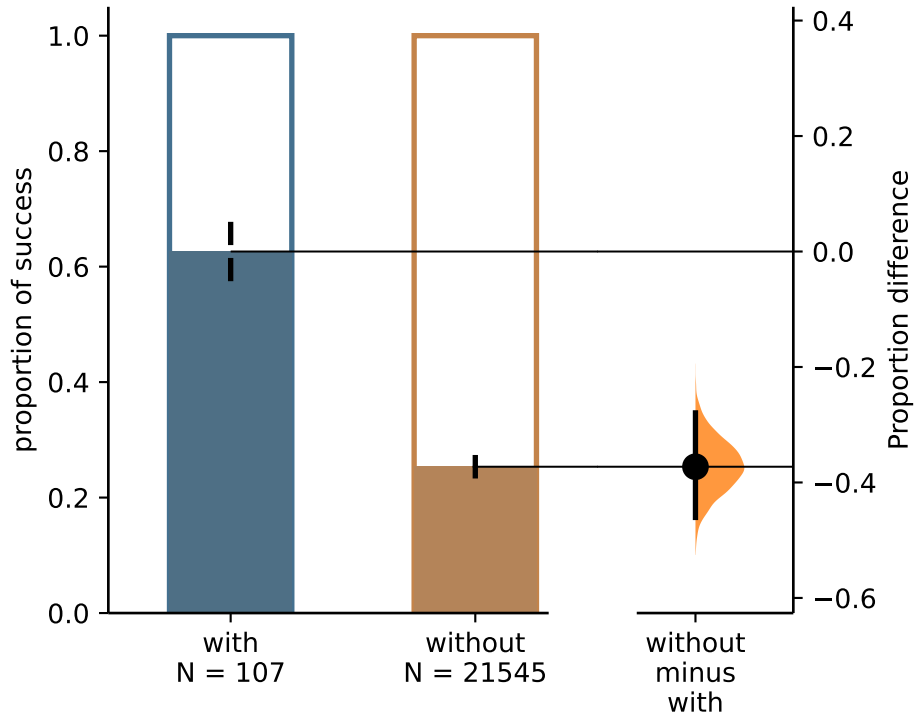

Supplement: SC-016-D5SC01100K-s001 [file SC-016-D5SC01100K-s001.zip › ESI/si_images/structural_analysis/vis_gpt_ol72_mean_diff.pdf]

proportion of success

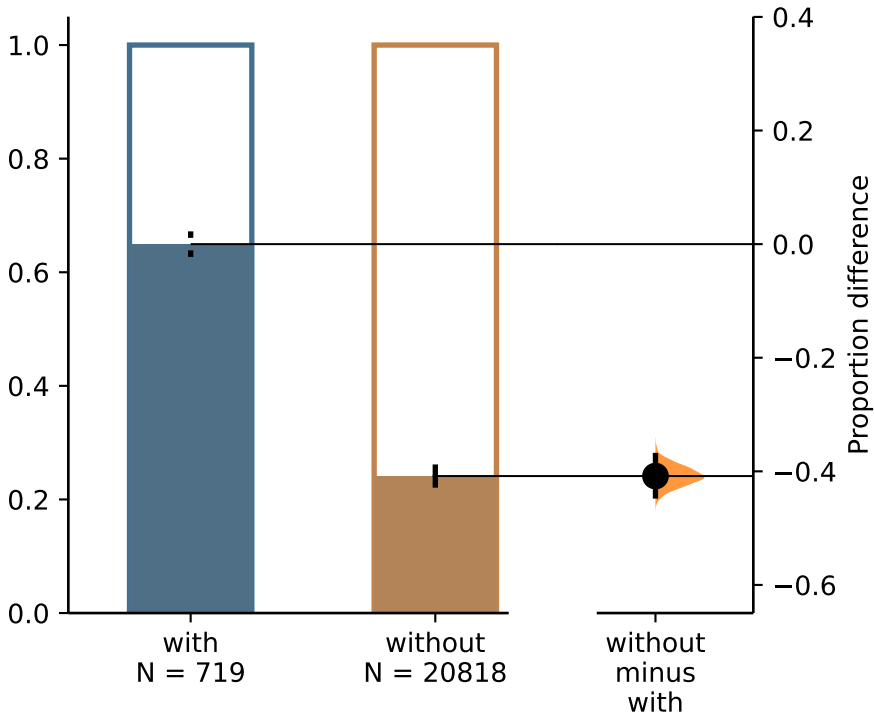

Supplement: SC-016-D5SC01100K-s001 [file SC-016-D5SC01100K-s001.zip › ESI/si_images/structural_analysis/vis_gpt_tp20_mean_diff.pdf]

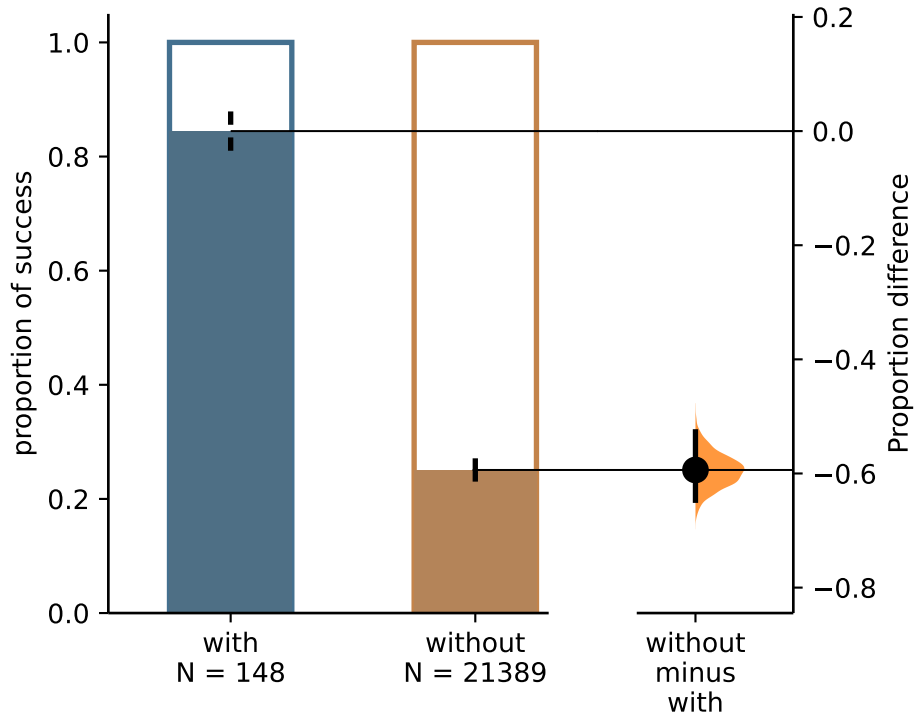

Supplement: SC-016-D5SC01100K-s001 [file SC-016-D5SC01100K-s001.zip › ESI/si_images/structural_analysis/vis_gpt_tp24_mean_diff.pdf]

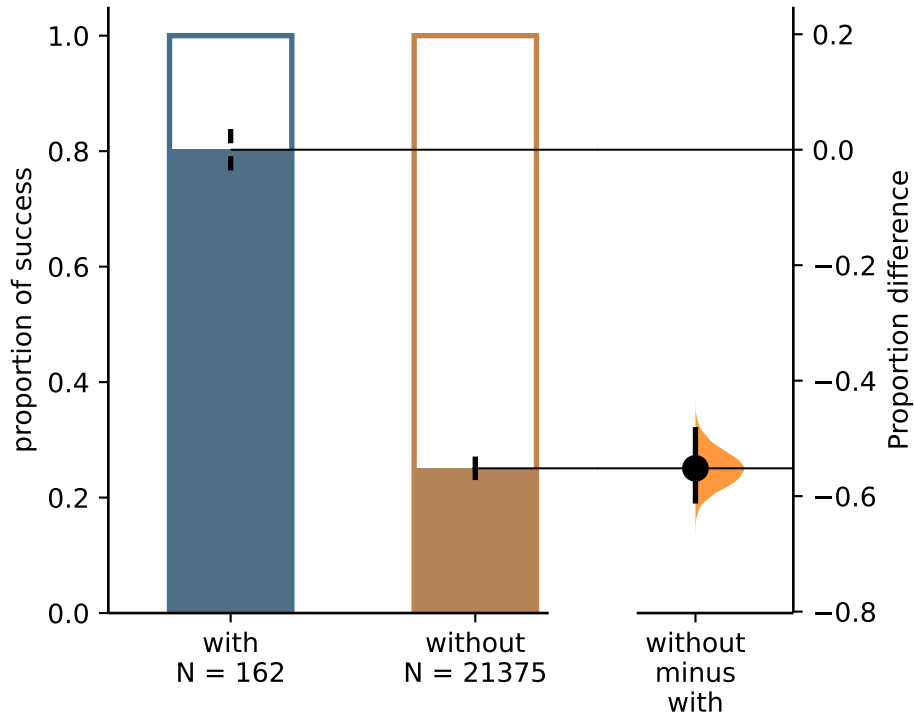

Supplement: SC-016-D5SC01100K-s001 [file SC-016-D5SC01100K-s001.zip › ESI/si_images/structural_analysis/vis_gpt_tp8_mean_diff.pdf]

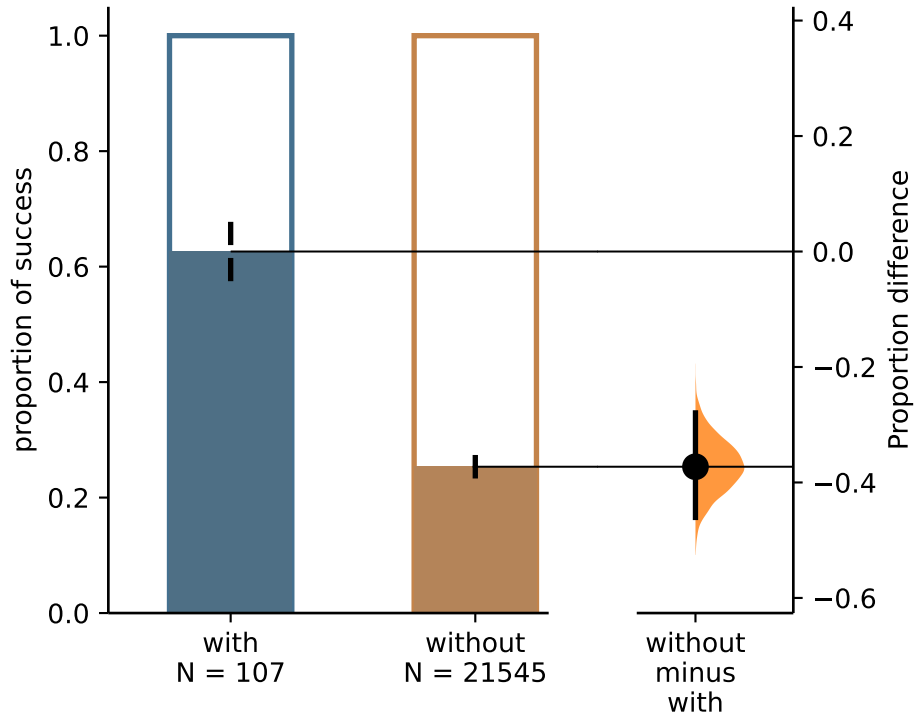

Supplement: SC-016-D5SC01100K-s001 [file SC-016-D5SC01100K-s001.zip › ESI/si_images/structural_analysis/vis_moft_ol1_mean_diff.pdf]

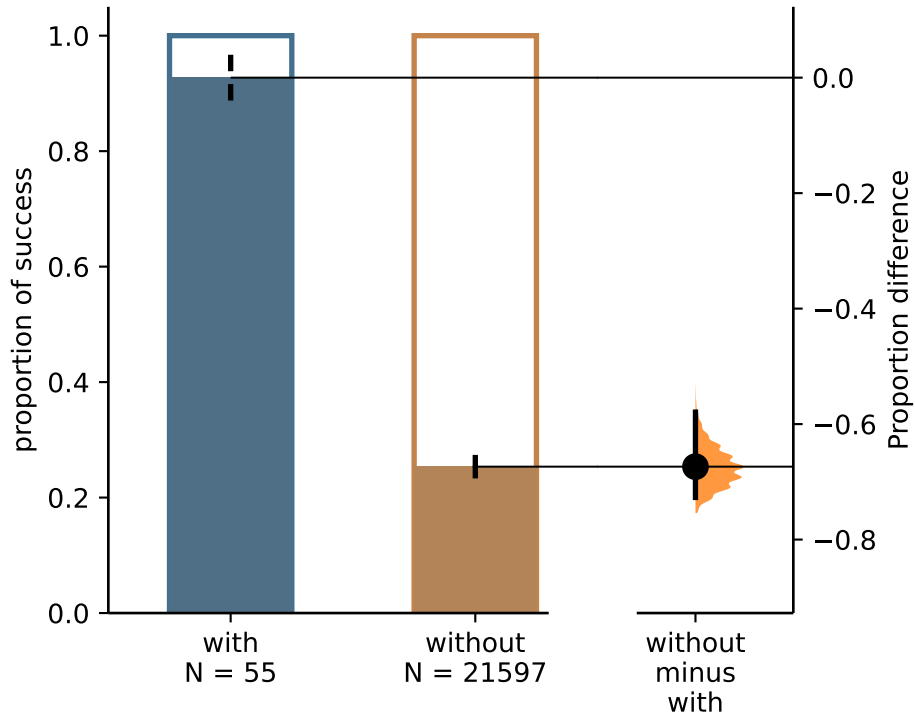

Supplement: SC-016-D5SC01100K-s001 [file SC-016-D5SC01100K-s001.zip › ESI/si_images/structural_analysis/vis_moft_ol30_mean_diff.pdf]

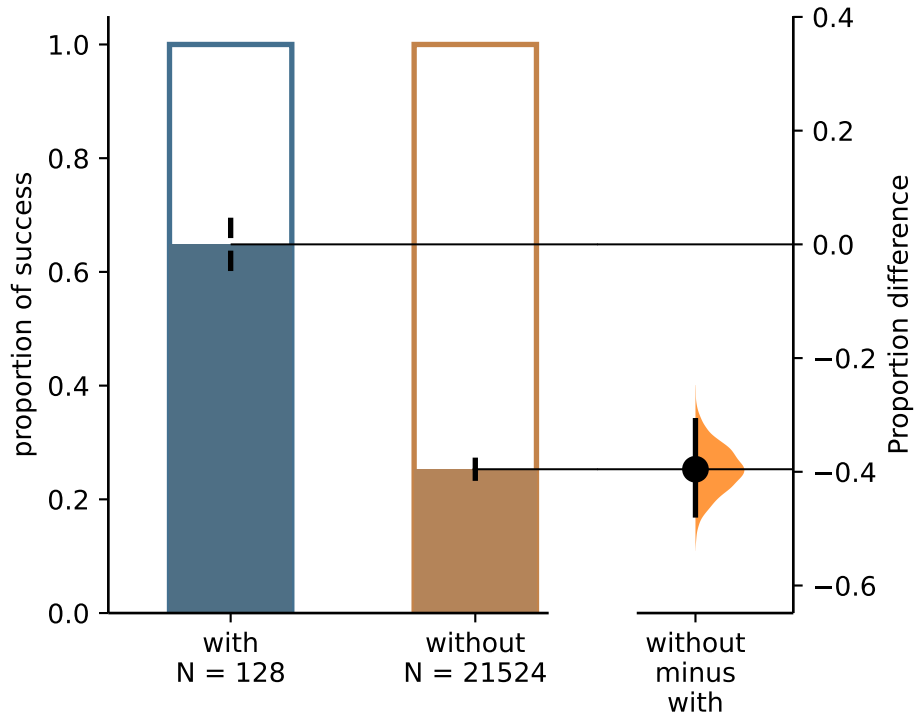

Supplement: SC-016-D5SC01100K-s001 [file SC-016-D5SC01100K-s001.zip › ESI/si_images/structural_analysis/vis_moft_ol40_mean_diff.pdf]

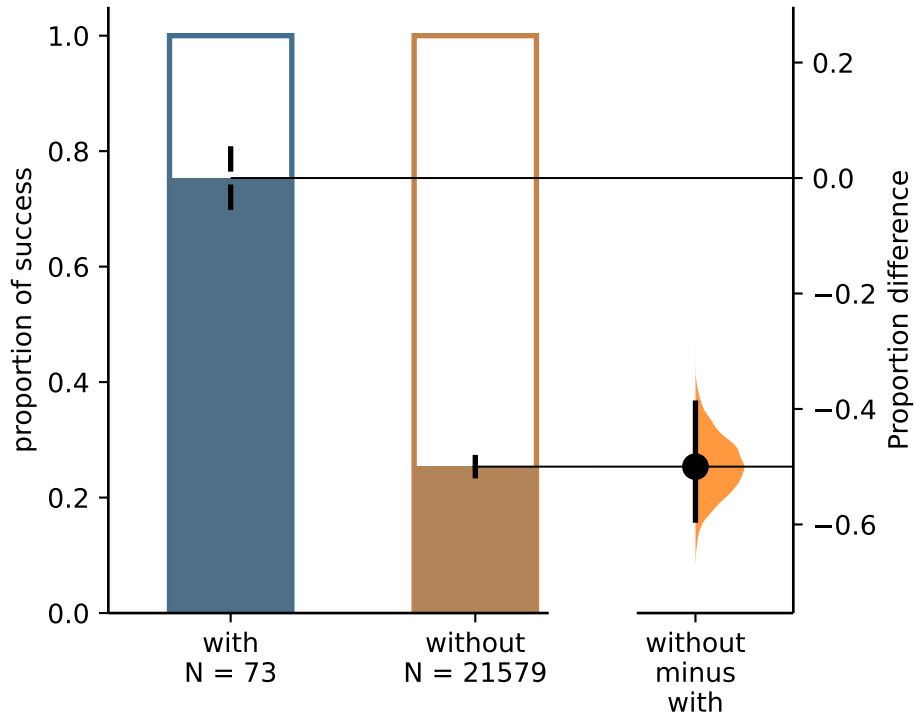

Supplement: SC-016-D5SC01100K-s001 [file SC-016-D5SC01100K-s001.zip › ESI/si_images/structural_analysis/vis_moft_ol5_mean_diff.pdf]

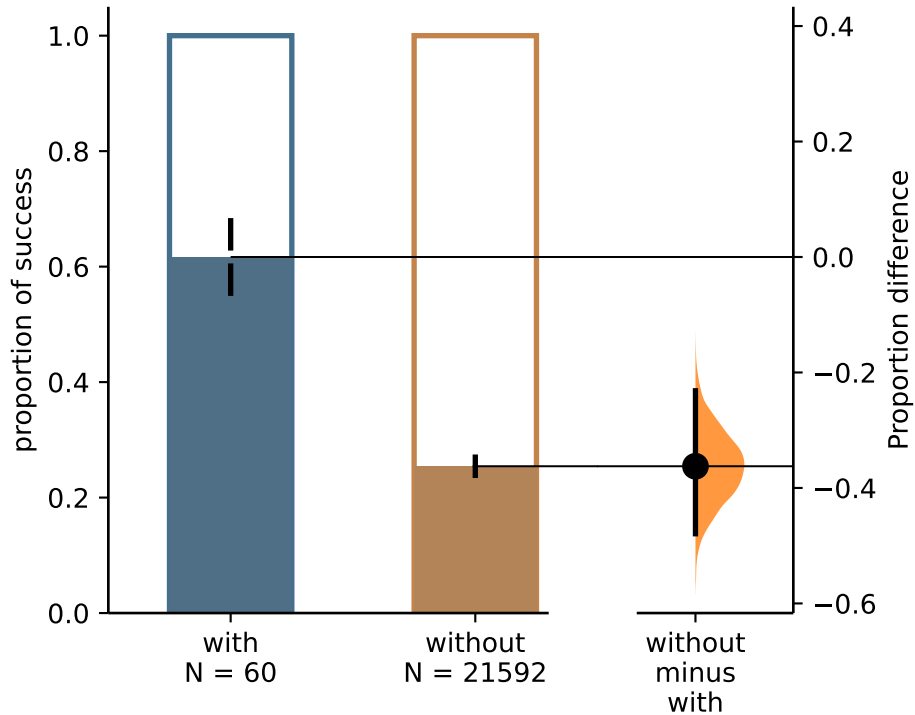

Supplement: SC-016-D5SC01100K-s001 [file SC-016-D5SC01100K-s001.zip › ESI/si_images/structural_analysis/vis_moft_ol68_mean_diff.pdf]

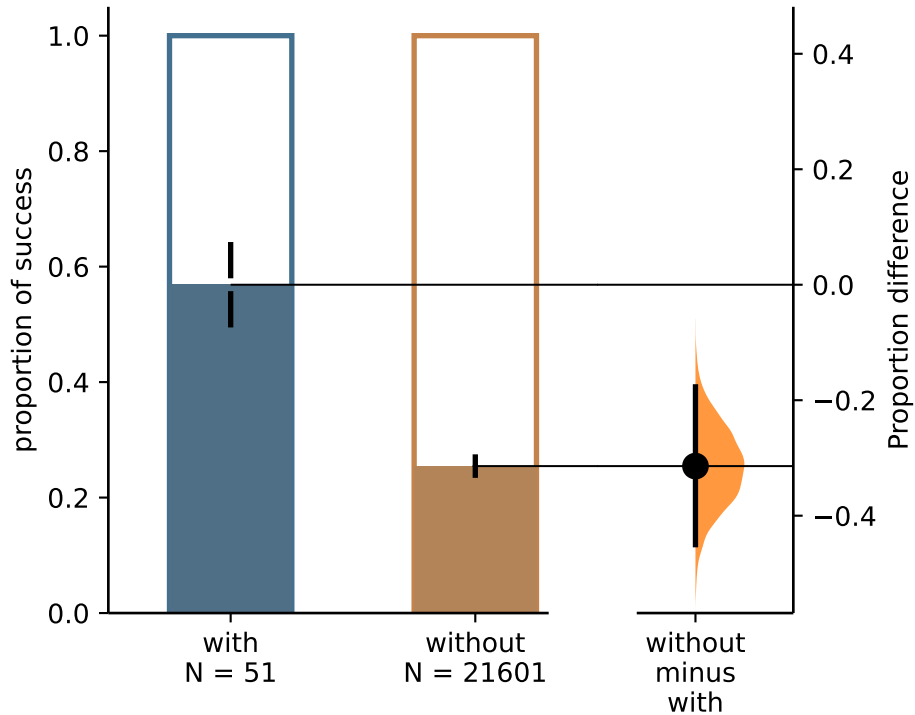

Supplement: SC-016-D5SC01100K-s001 [file SC-016-D5SC01100K-s001.zip › ESI/si_images/structural_analysis/vis_moft_ol72_mean_diff.pdf]

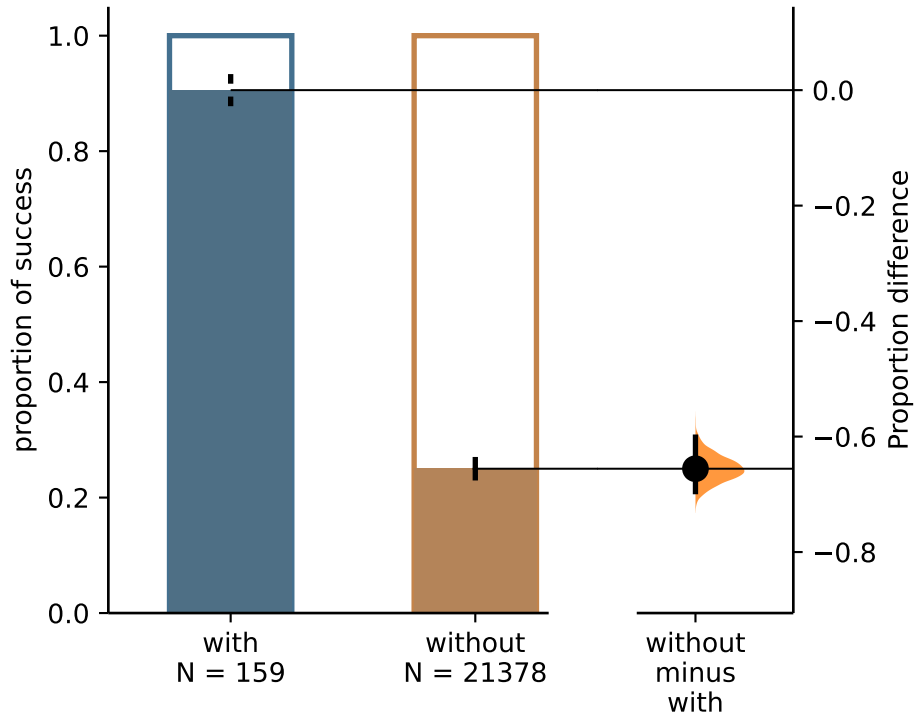

Supplement: SC-016-D5SC01100K-s001 [file SC-016-D5SC01100K-s001.zip › ESI/si_images/structural_analysis/vis_moft_tp20_mean_diff.pdf]

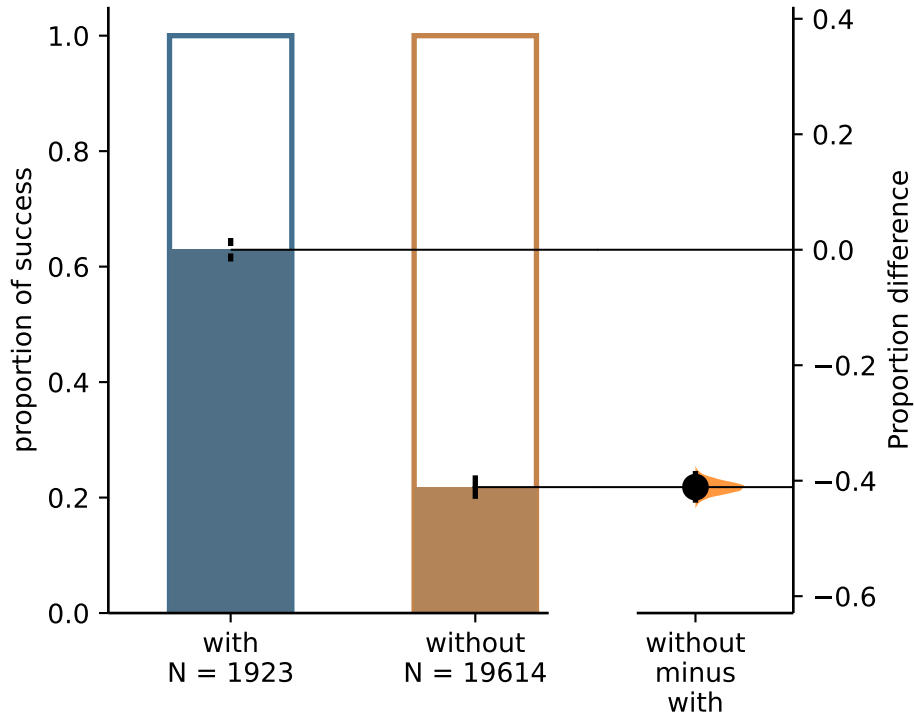

Supplement: SC-016-D5SC01100K-s001 [file SC-016-D5SC01100K-s001.zip › ESI/si_images/structural_analysis/vis_moft_tp24_mean_diff.pdf]

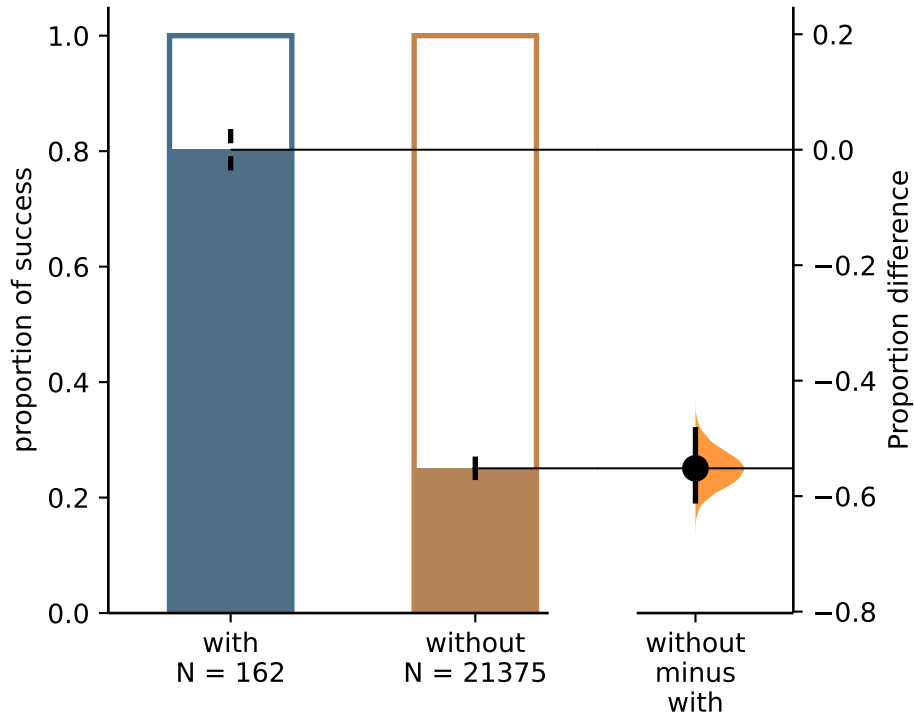

Supplement: SC-016-D5SC01100K-s001 [file SC-016-D5SC01100K-s001.zip › ESI/si_images/structural_analysis/vis_moft_tp8_mean_diff.pdf]

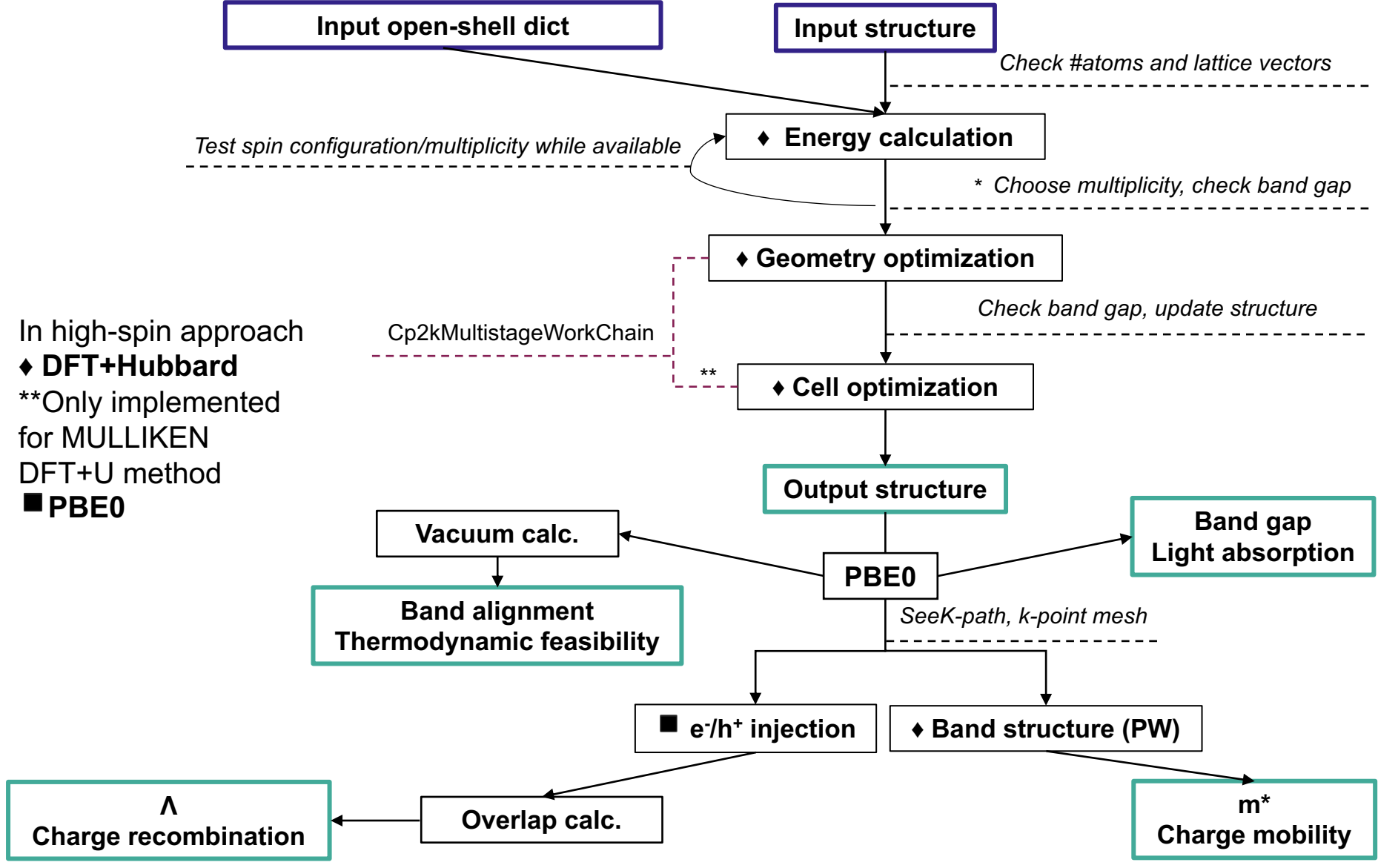

Supplement: SC-016-D5SC01100K-s001 [file SC-016-D5SC01100K-s001.zip › ESI/si_images/temp_wf.pdf]

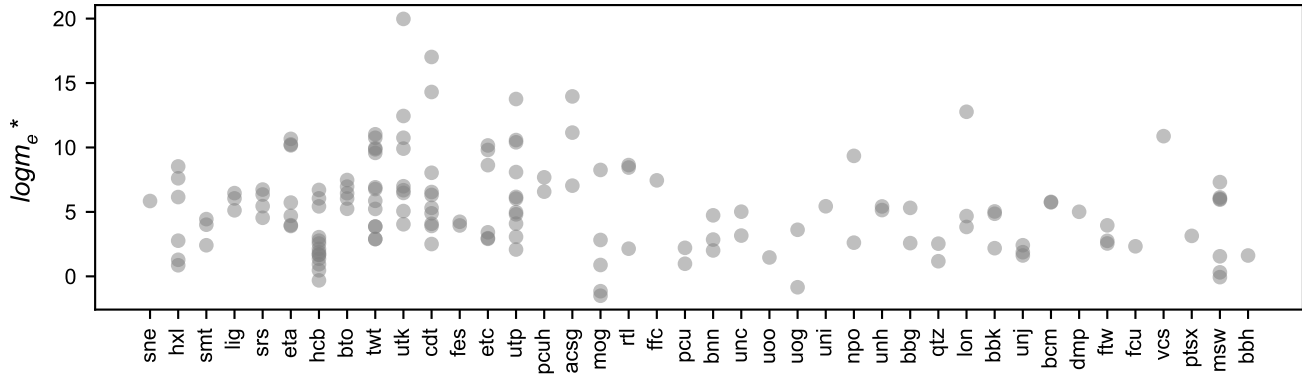

Supplement: SC-016-D5SC01100K-s001 [file SC-016-D5SC01100K-s001.zip › ESI/si_images/topologies_def_me.pdf]

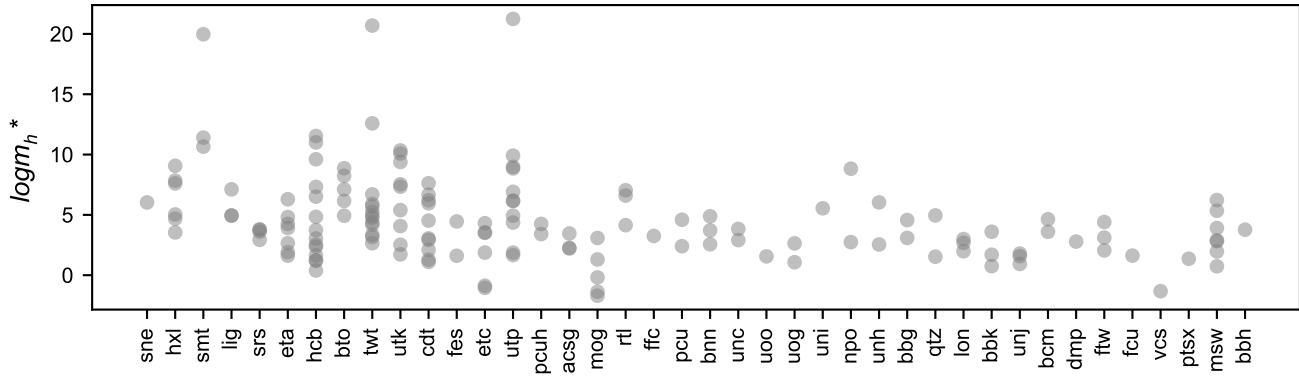

Supplement: SC-016-D5SC01100K-s001 [file SC-016-D5SC01100K-s001.zip › ESI/si_images/topologies_def_mh.pdf]

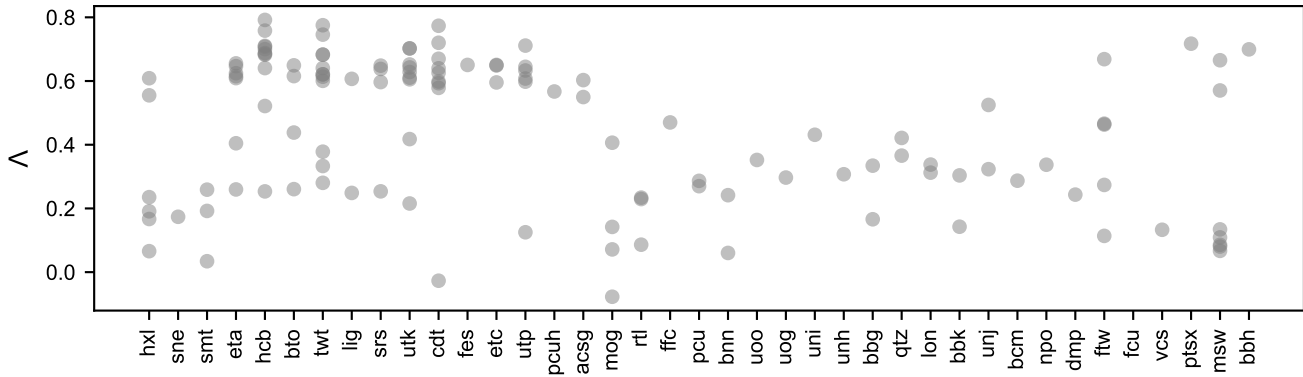

Supplement: SC-016-D5SC01100K-s001 [file SC-016-D5SC01100K-s001.zip › ESI/si_images/topologies_def_ovlp.pdf]

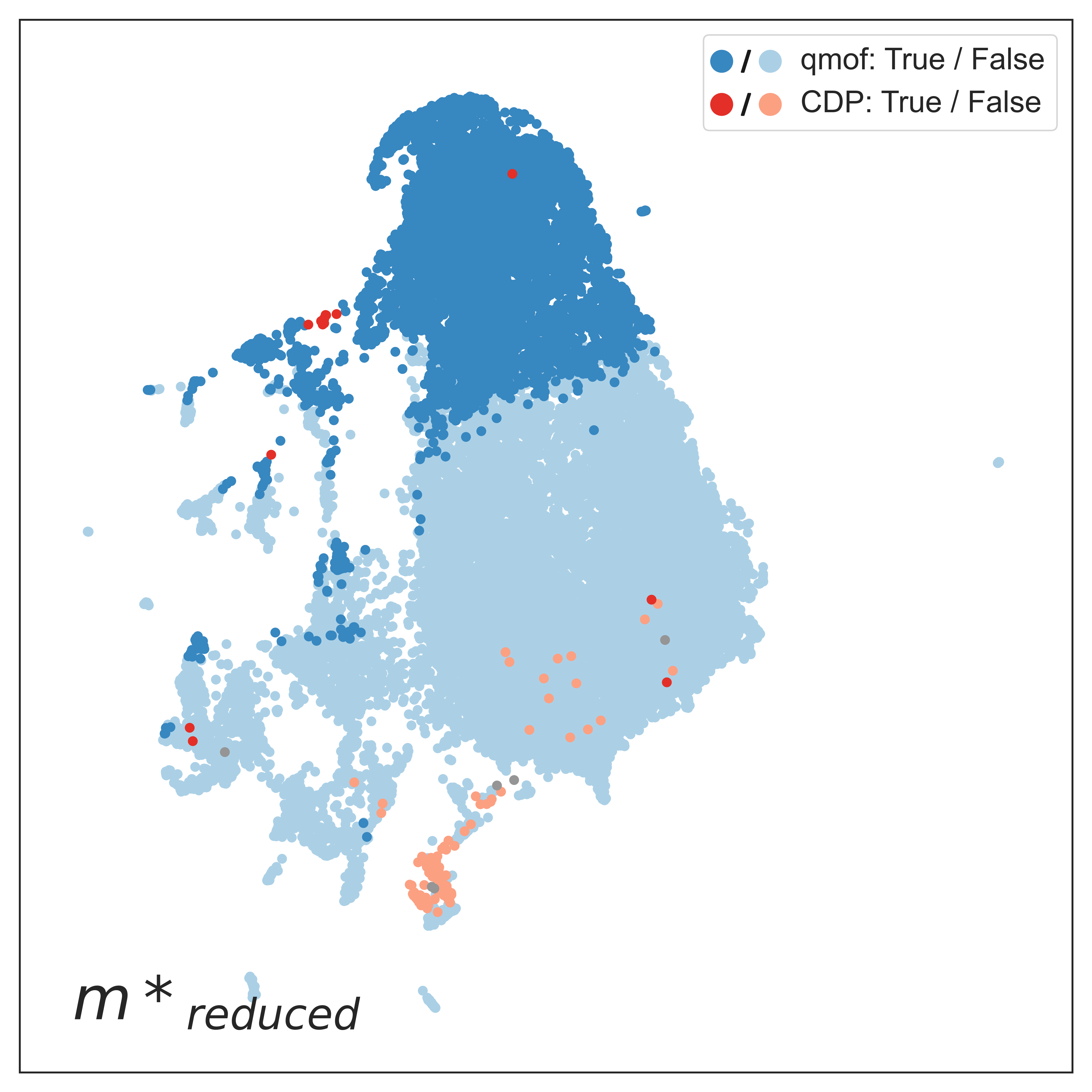

Supplement: SC-016-D5SC01100K-s001 [file SC-016-D5SC01100K-s001.zip › ESI/si_images/umap/eff1_qmof_CDP_cls_feat.png]

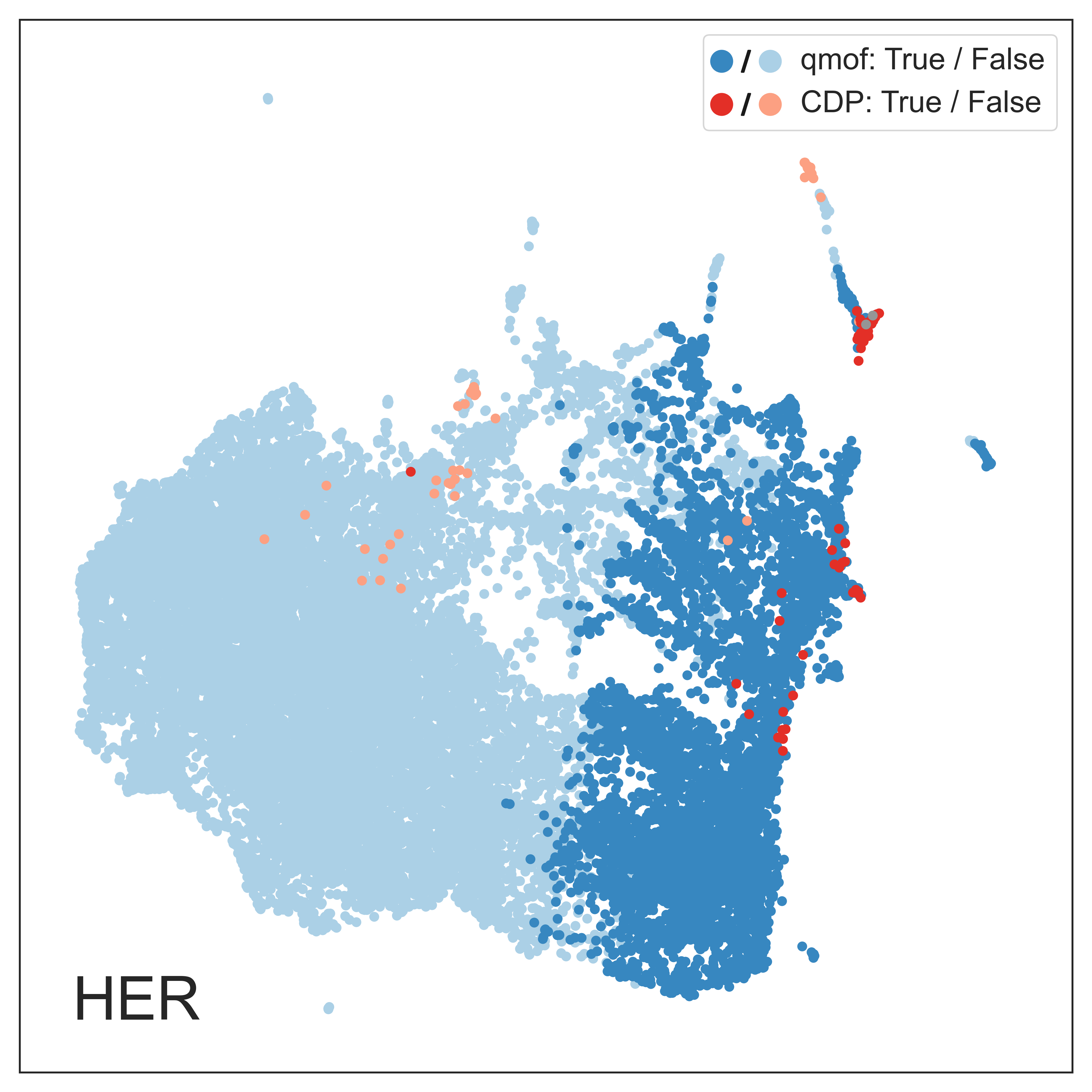

Supplement: SC-016-D5SC01100K-s001 [file SC-016-D5SC01100K-s001.zip › ESI/si_images/umap/HER_qmof_CDP_cls_feat.png]

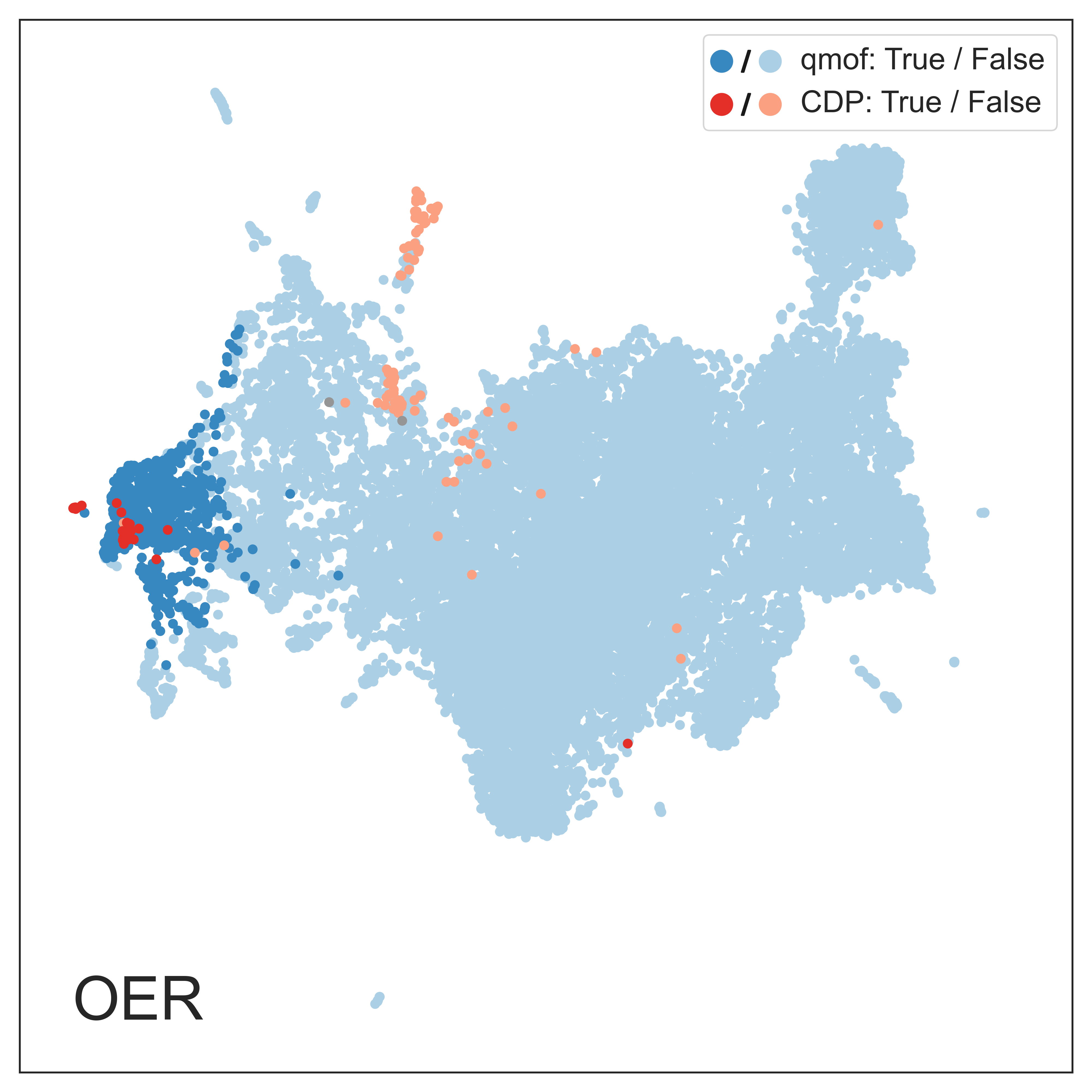

Supplement: SC-016-D5SC01100K-s001 [file SC-016-D5SC01100K-s001.zip › ESI/si_images/umap/OER_qmof_CDP_cls_feat.png]

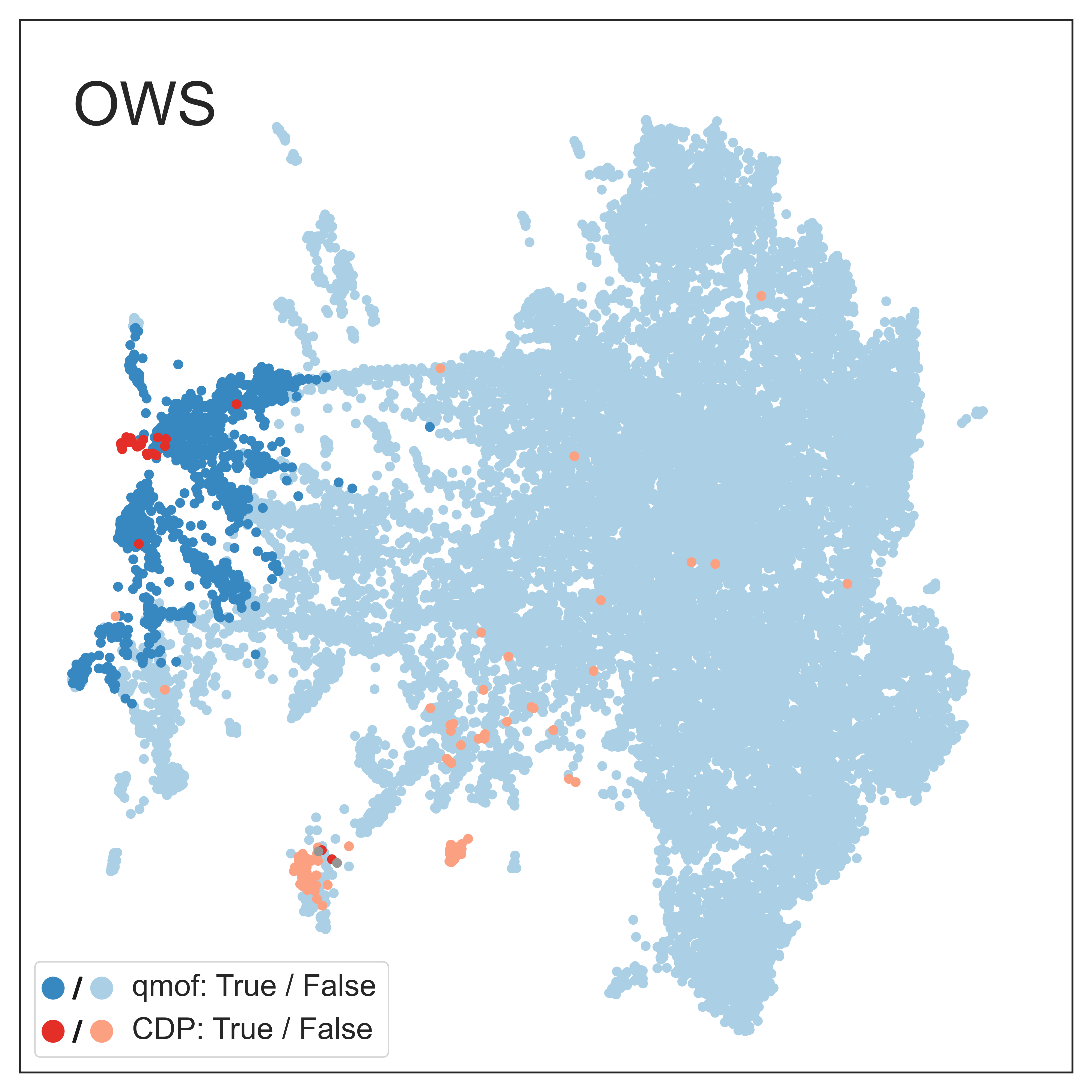

Supplement: SC-016-D5SC01100K-s001 [file SC-016-D5SC01100K-s001.zip › ESI/si_images/umap/OWS_qmof_CDP_cls_feat.png]

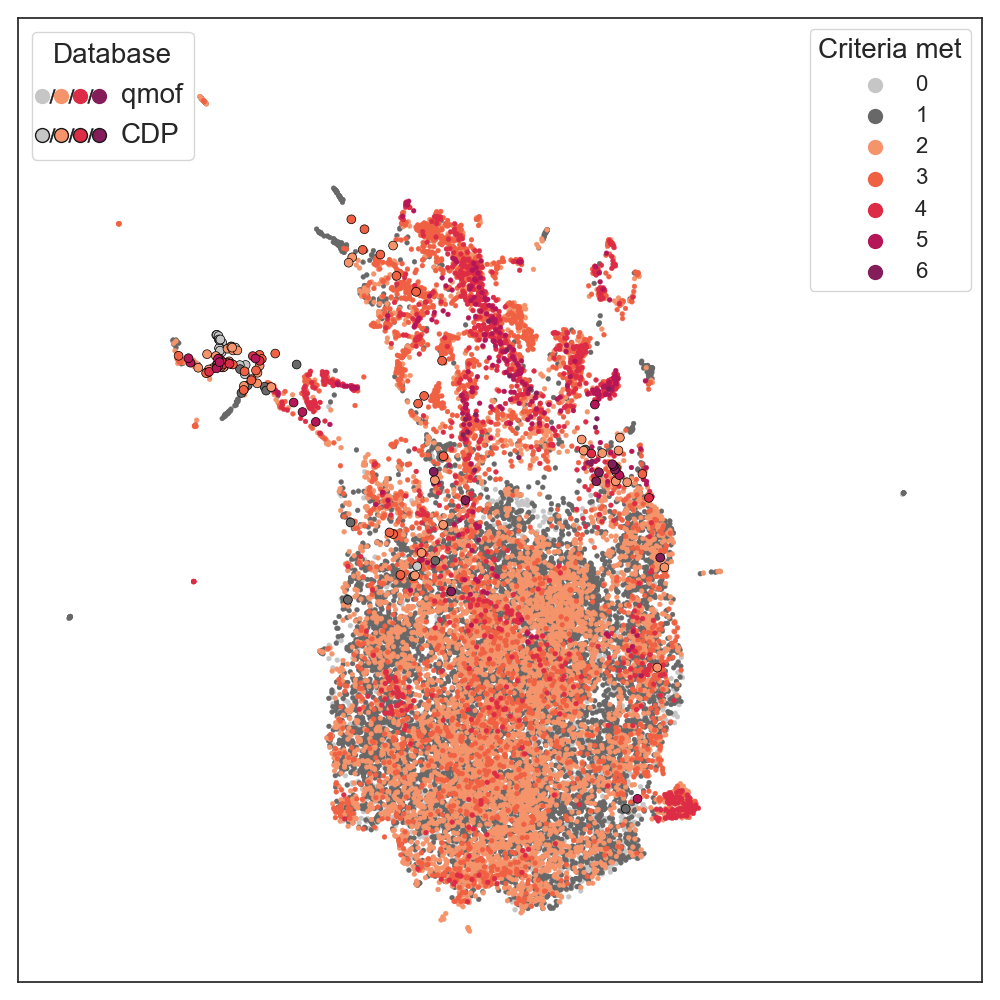

Supplement: SC-016-D5SC01100K-s001 [file SC-016-D5SC01100K-s001.zip › ESI/si_images/umap/pretrained_criteria_met_qmof_CDP_highlighted_cls_feat_alt.png]

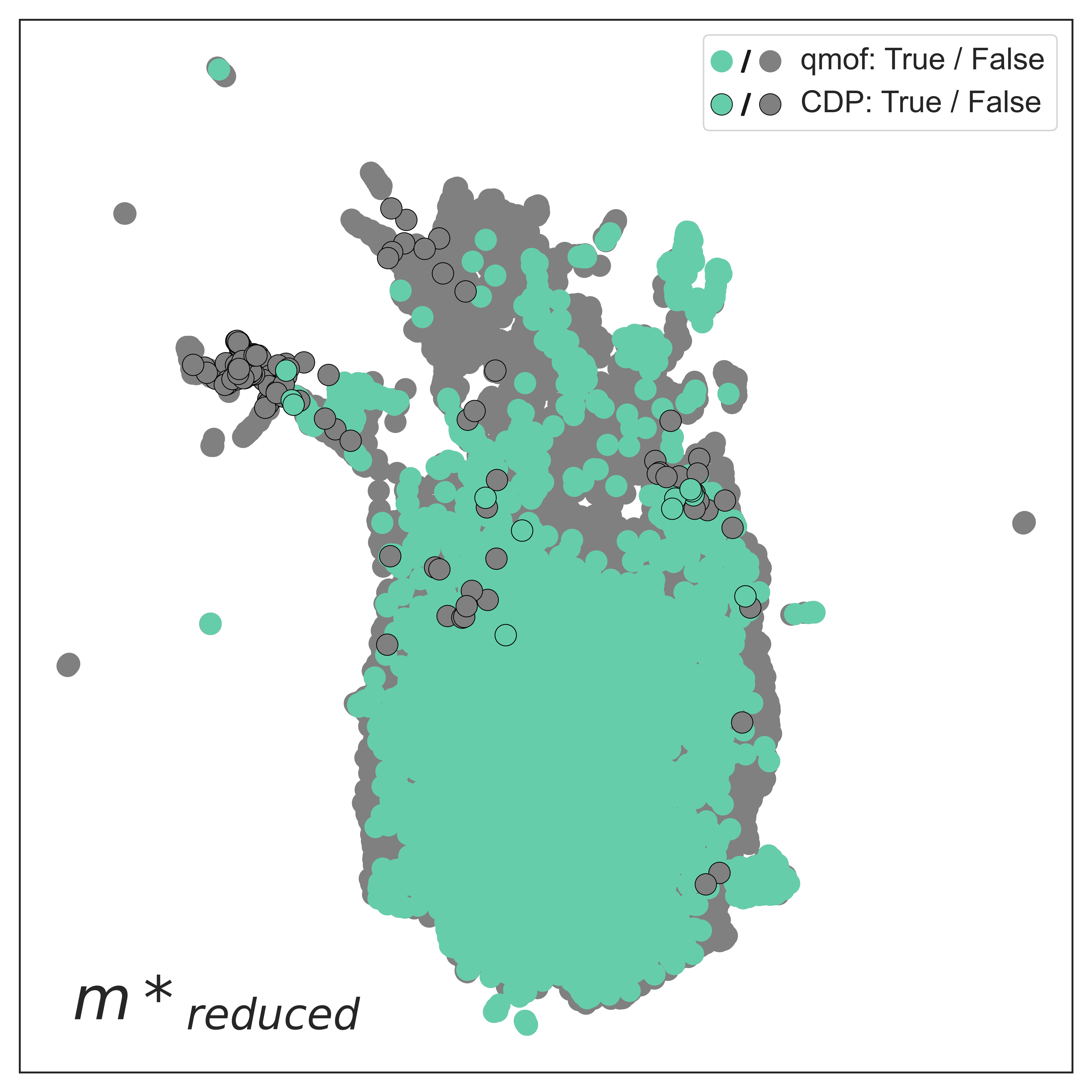

Supplement: SC-016-D5SC01100K-s001 [file SC-016-D5SC01100K-s001.zip › ESI/si_images/umap/pretrained_eff1_qmof_CDP_cls_feat.png]

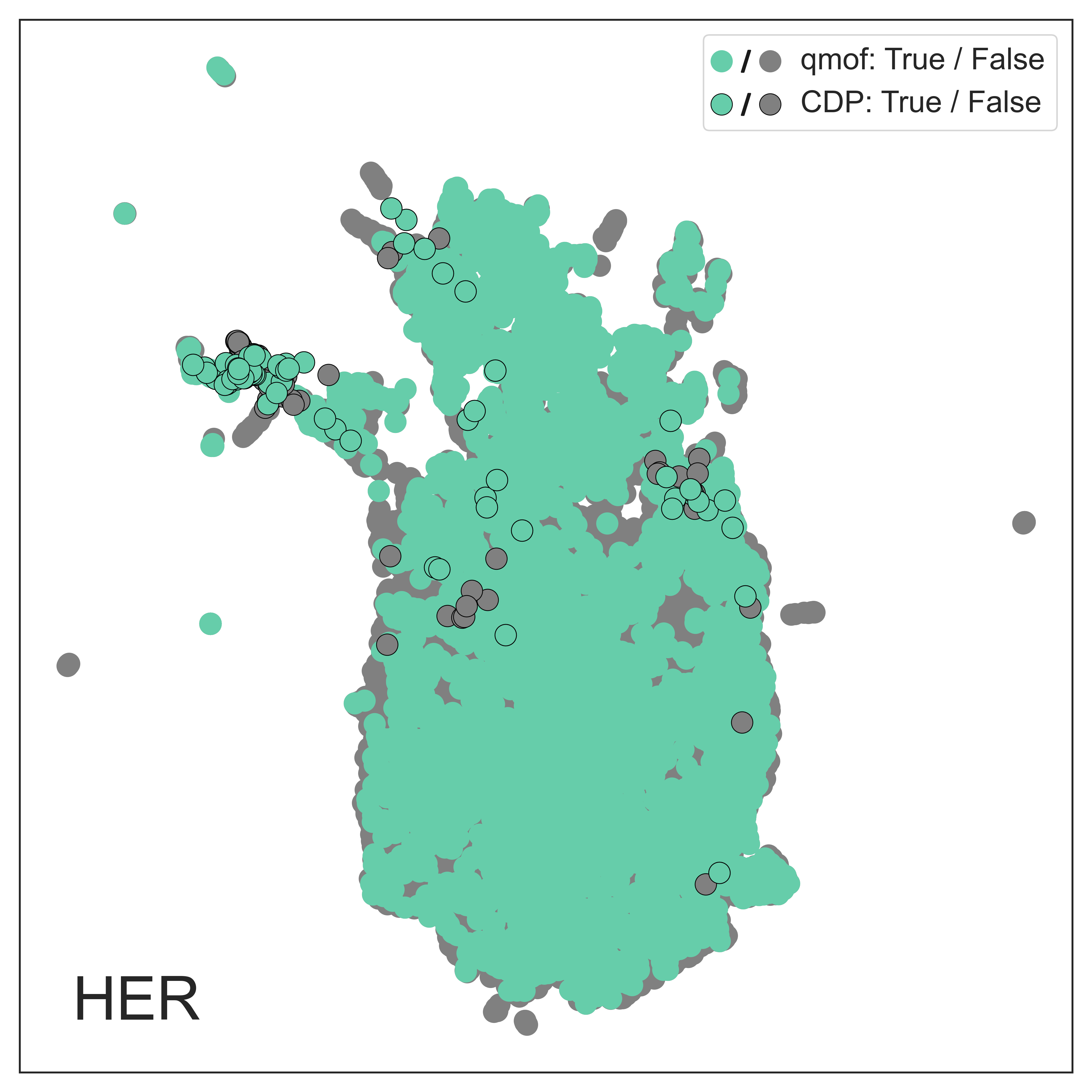

Supplement: SC-016-D5SC01100K-s001 [file SC-016-D5SC01100K-s001.zip › ESI/si_images/umap/pretrained_HER_qmof_CDP_cls_feat.png]

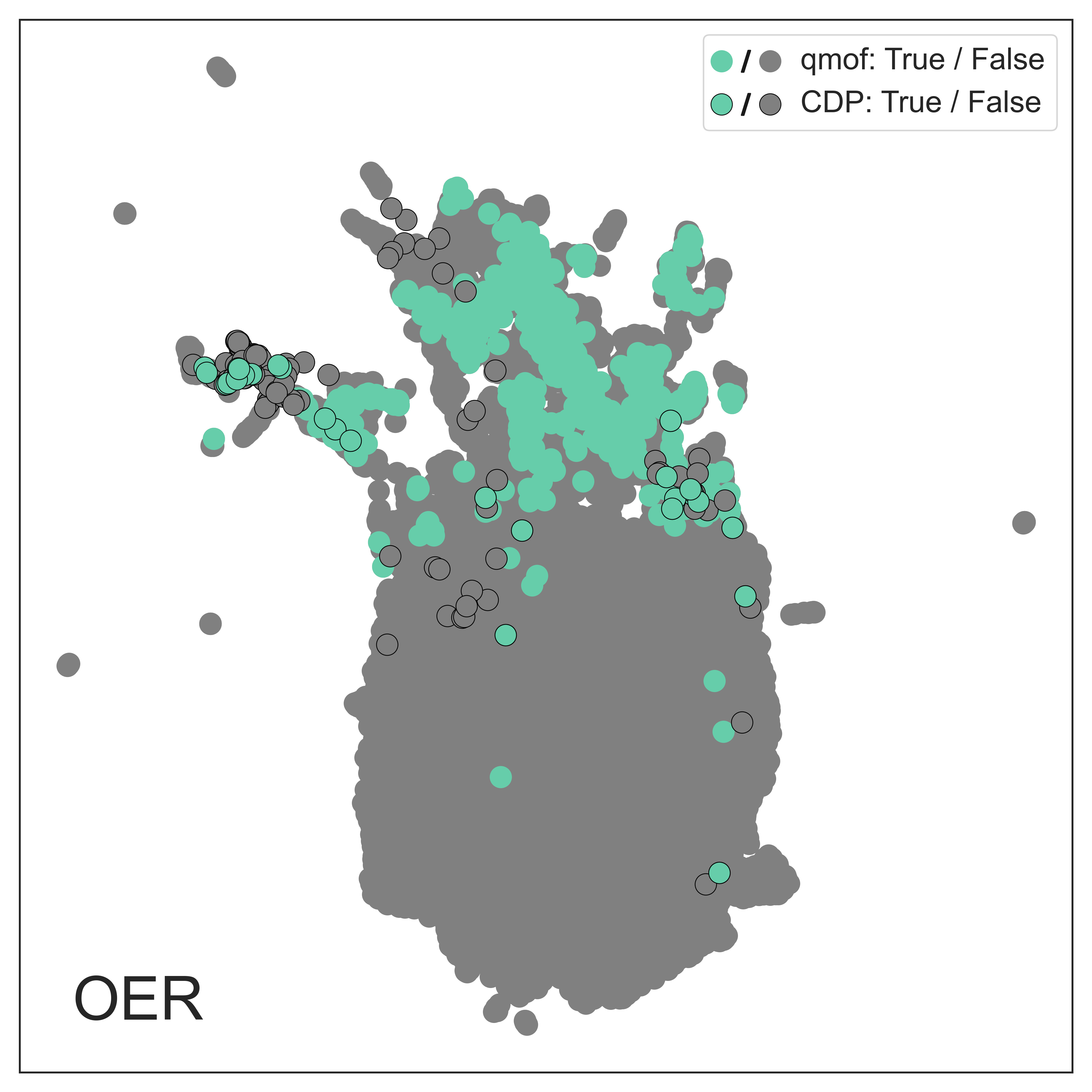

Supplement: SC-016-D5SC01100K-s001 [file SC-016-D5SC01100K-s001.zip › ESI/si_images/umap/pretrained_OER_qmof_CDP_cls_feat.png]

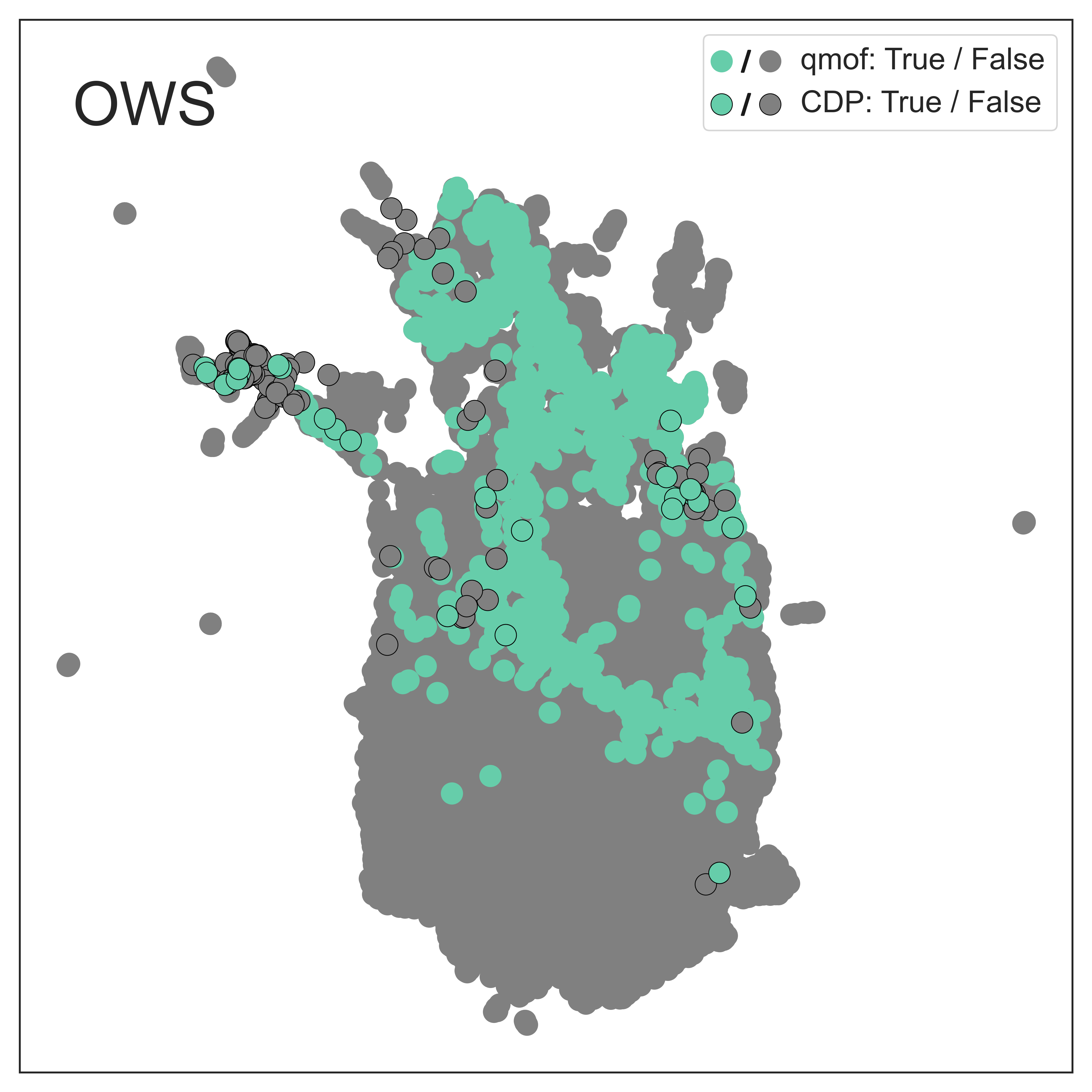

Supplement: SC-016-D5SC01100K-s001 [file SC-016-D5SC01100K-s001.zip › ESI/si_images/umap/pretrained_OWS_qmof_CDP_cls_feat.png]

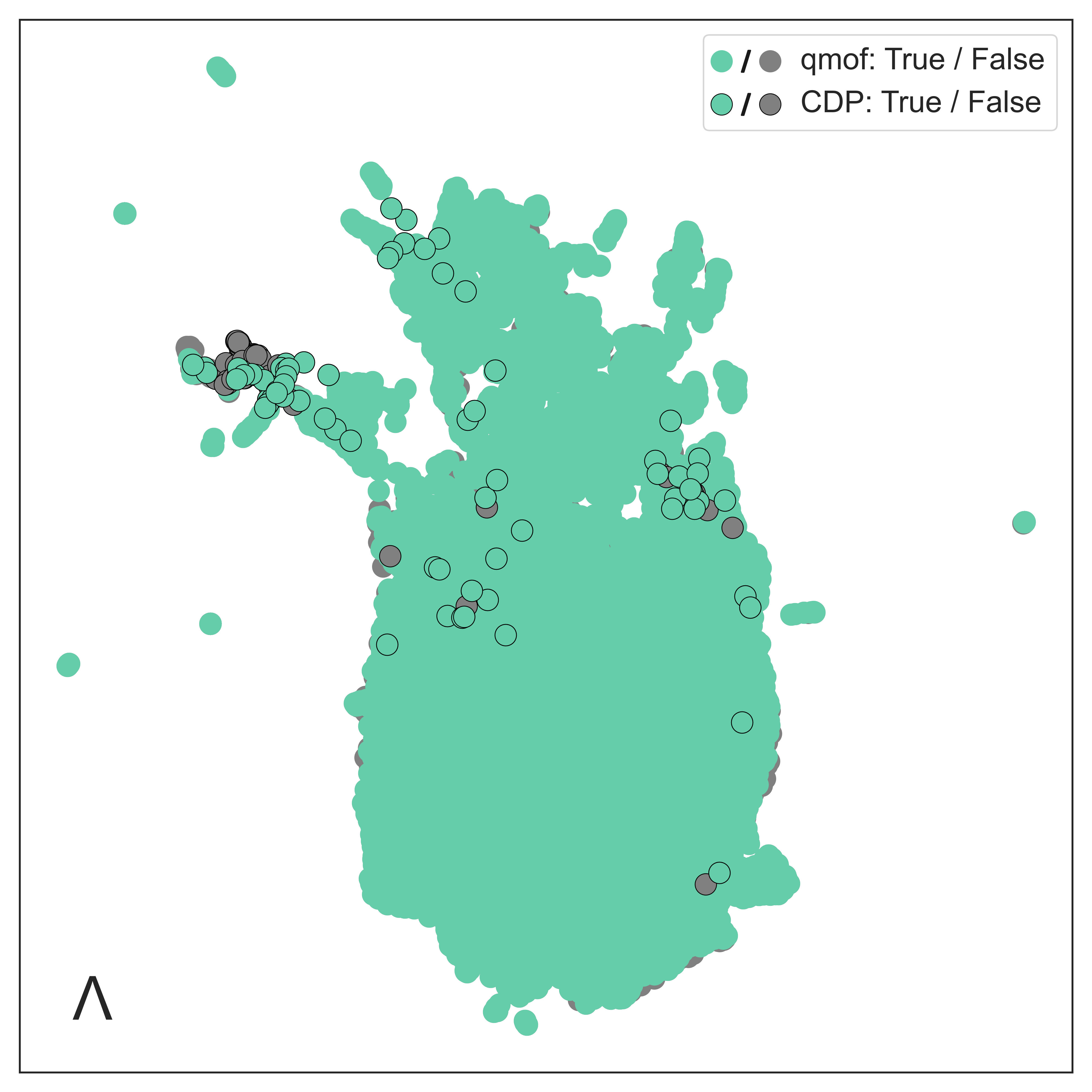

Supplement: SC-016-D5SC01100K-s001 [file SC-016-D5SC01100K-s001.zip › ESI/si_images/umap/pretrained_spo_qmof_CDP_cls_feat.png]

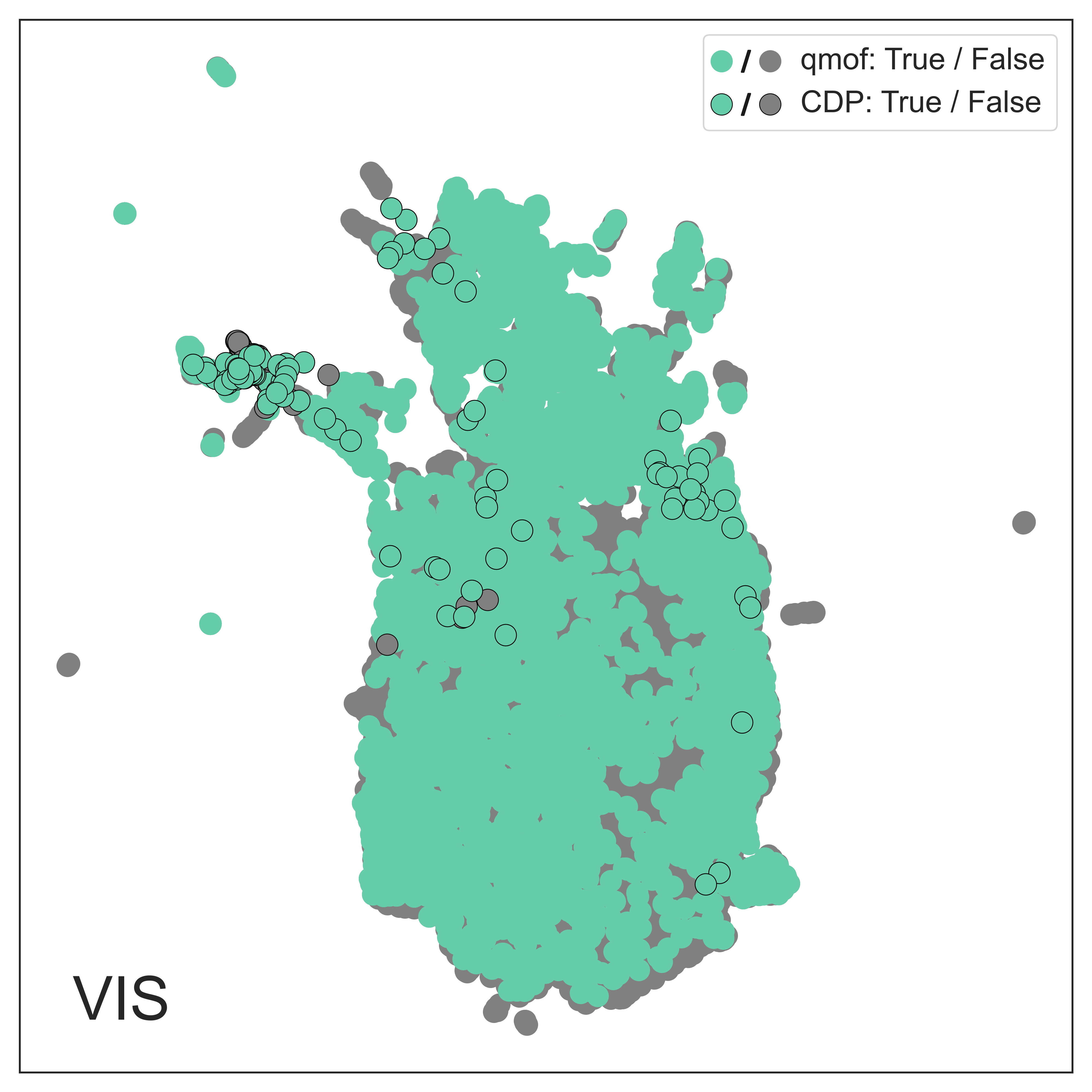

Supplement: SC-016-D5SC01100K-s001 [file SC-016-D5SC01100K-s001.zip › ESI/si_images/umap/pretrained_VIS_qmof_CDP_cls_feat.png]

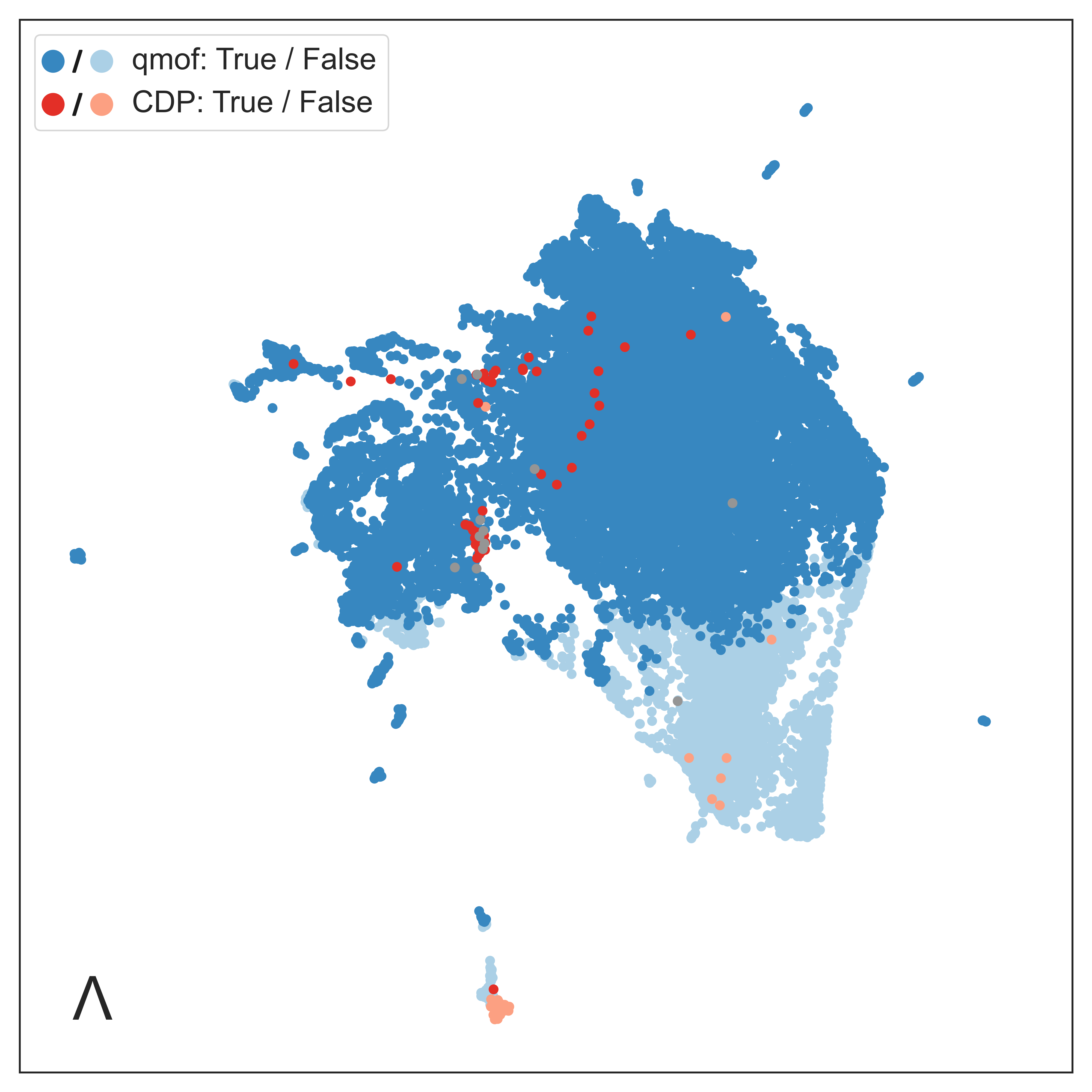

Supplement: SC-016-D5SC01100K-s001 [file SC-016-D5SC01100K-s001.zip › ESI/si_images/umap/spo_qmof_CDP_cls_feat.png]

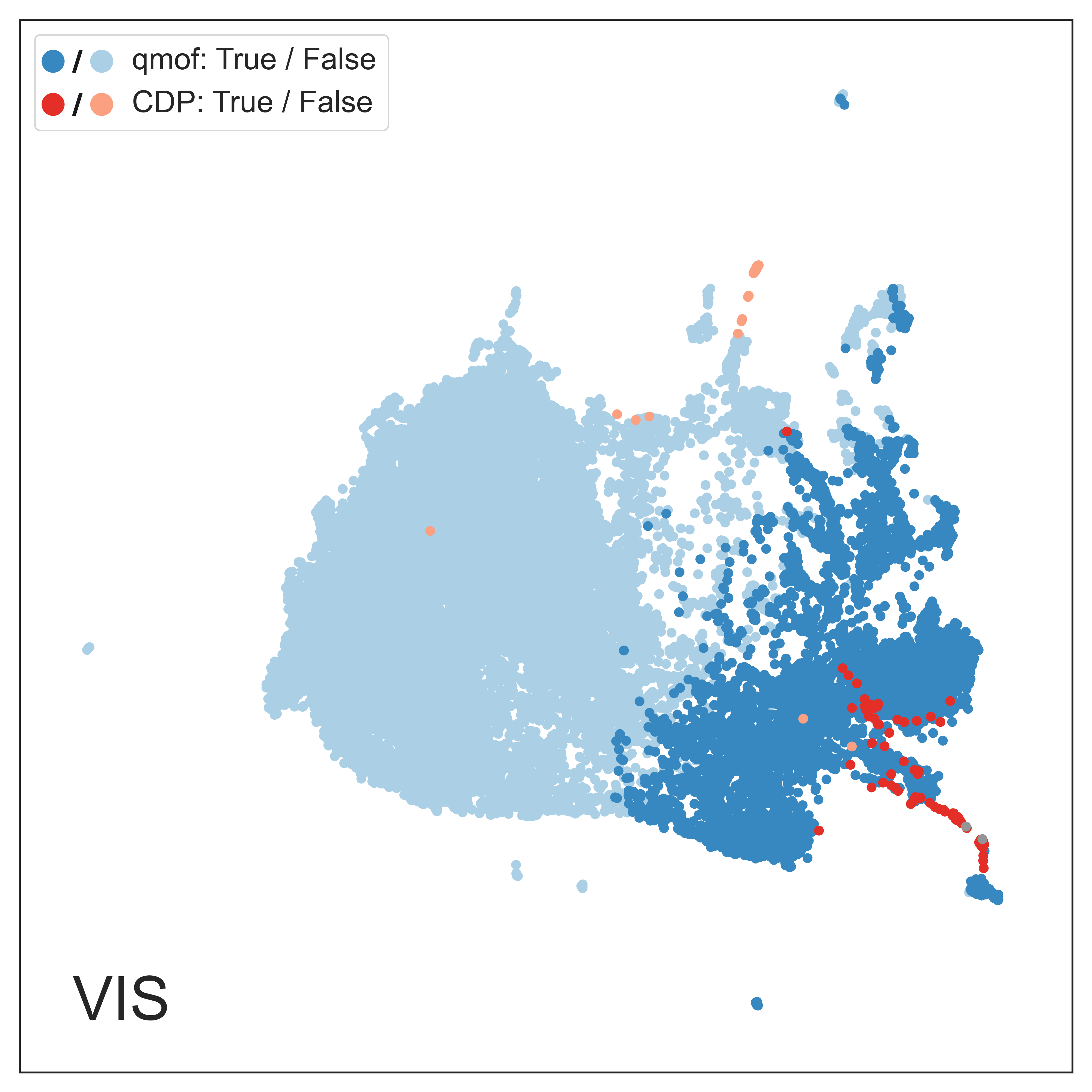

Supplement: SC-016-D5SC01100K-s001 [file SC-016-D5SC01100K-s001.zip › ESI/si_images/umap/VIS_qmof_CDP_cls_feat.png]

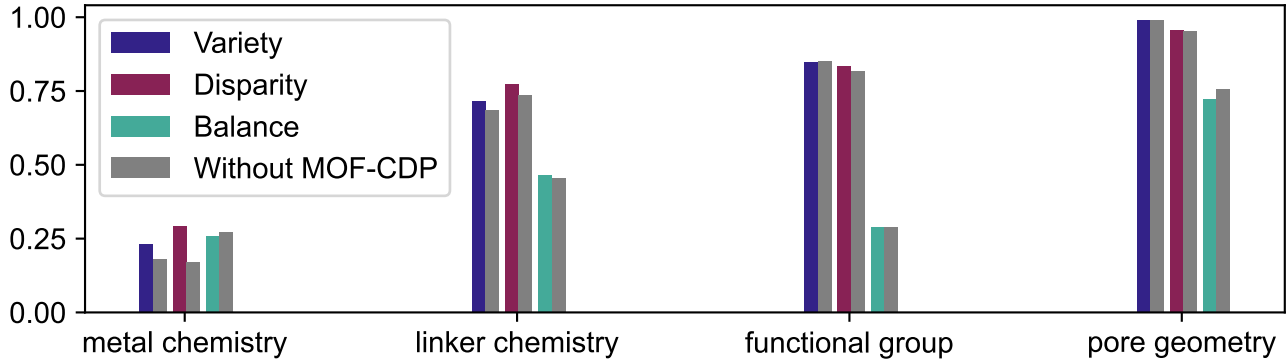

Supplement: SC-016-D5SC01100K-s001 [file SC-016-D5SC01100K-s001.zip › ESI/si_images/vbd_all_plots_2.pdf]

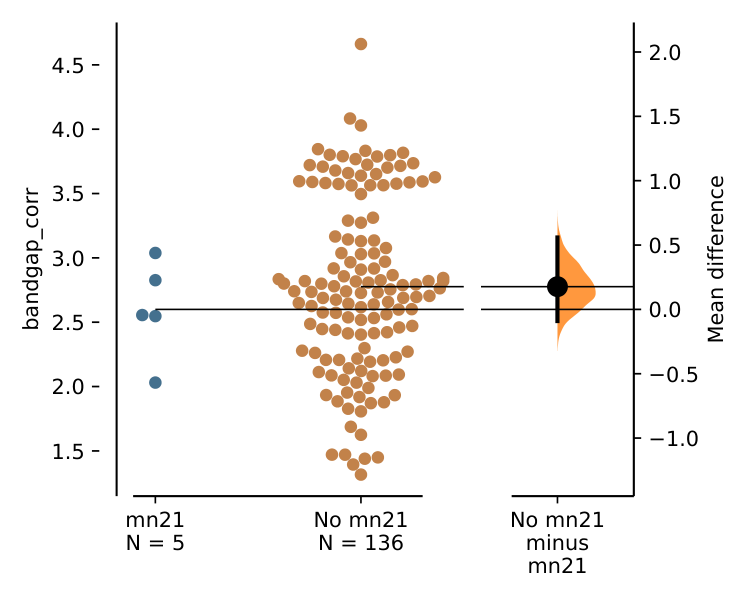

Supplement: SC-016-D5SC01100K-s001 [file SC-016-D5SC01100K-s001.zip › ESI/si_images_lowres/bg_mn21.png]

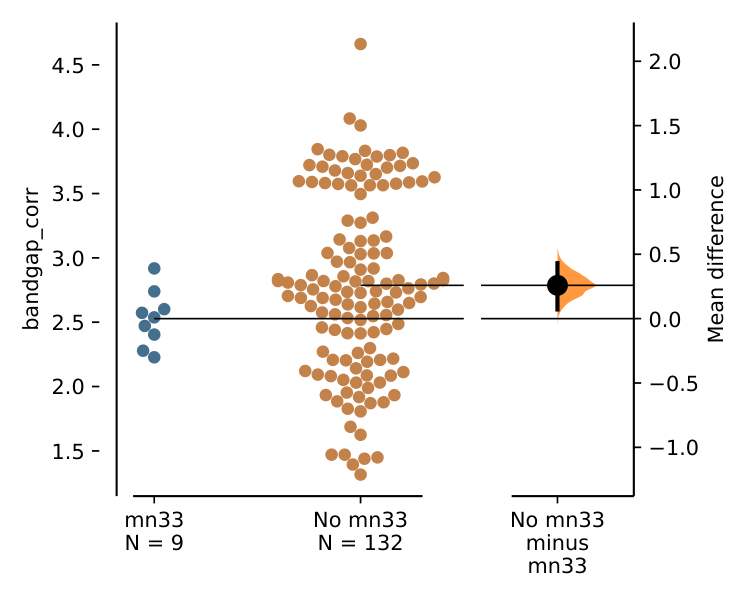

Supplement: SC-016-D5SC01100K-s001 [file SC-016-D5SC01100K-s001.zip › ESI/si_images_lowres/bg_mn33.png]

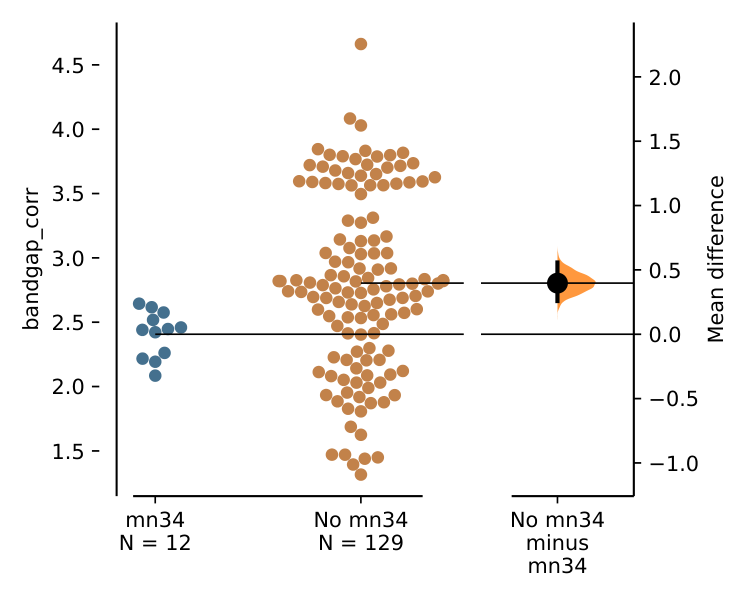

Supplement: SC-016-D5SC01100K-s001 [file SC-016-D5SC01100K-s001.zip › ESI/si_images_lowres/bg_mn34.png]

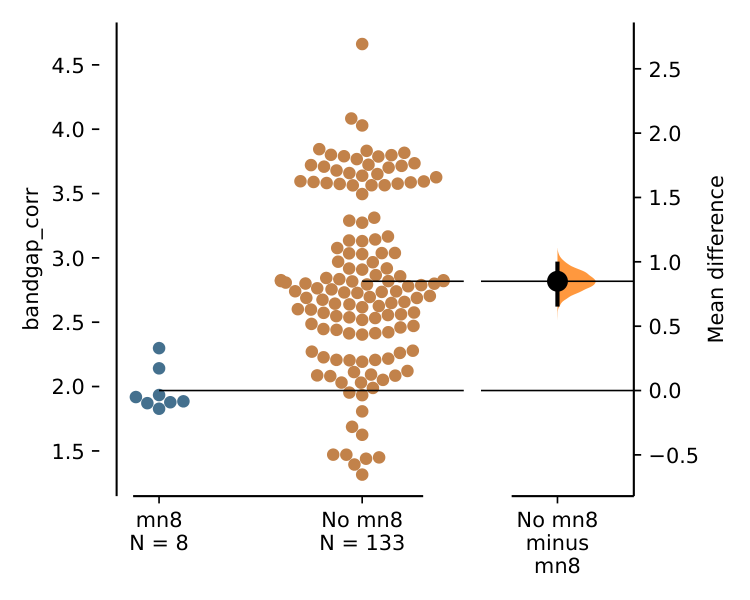

Supplement: SC-016-D5SC01100K-s001 [file SC-016-D5SC01100K-s001.zip › ESI/si_images_lowres/bg_mn8.png]

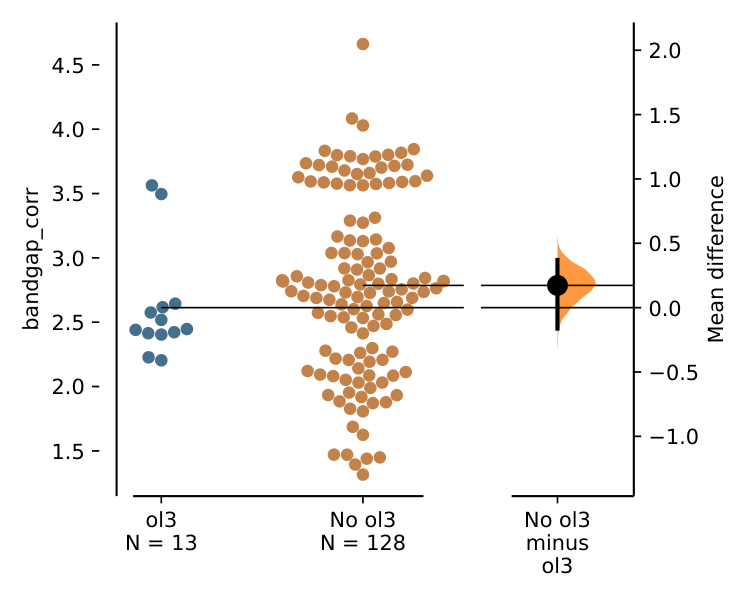

Supplement: SC-016-D5SC01100K-s001 [file SC-016-D5SC01100K-s001.zip › ESI/si_images_lowres/bg_ol3.png]

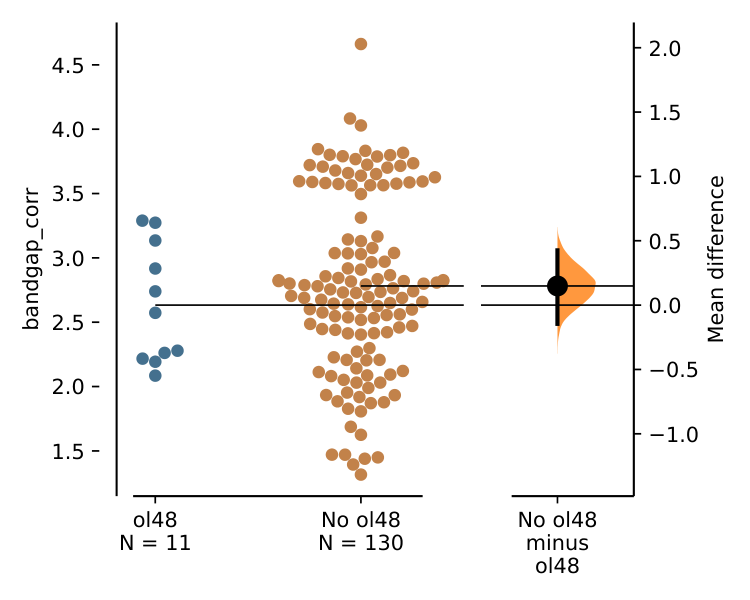

Supplement: SC-016-D5SC01100K-s001 [file SC-016-D5SC01100K-s001.zip › ESI/si_images_lowres/bg_ol48.png]

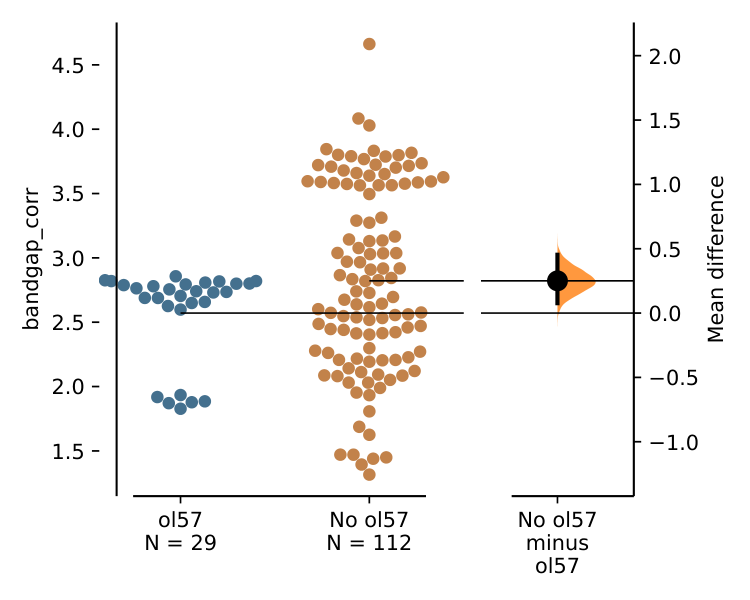

Supplement: SC-016-D5SC01100K-s001 [file SC-016-D5SC01100K-s001.zip › ESI/si_images_lowres/bg_ol57.png]

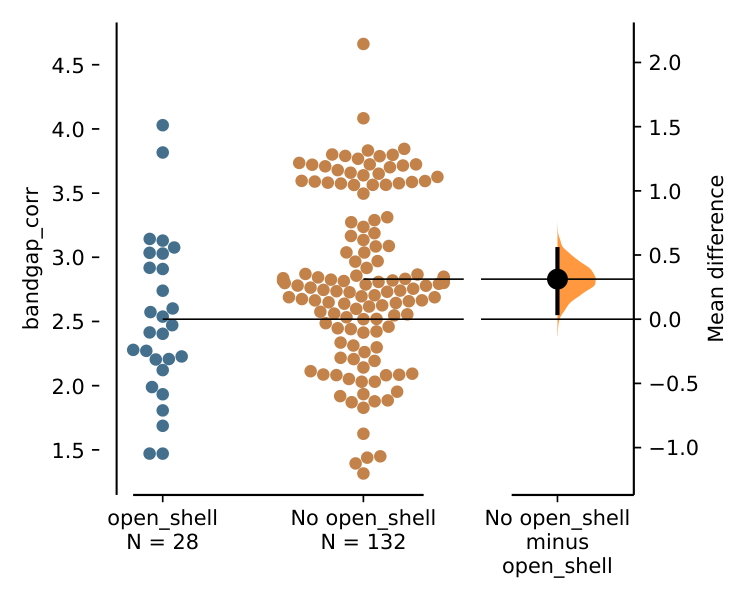

Supplement: SC-016-D5SC01100K-s001 [file SC-016-D5SC01100K-s001.zip › ESI/si_images_lowres/bg_open_shell.png]

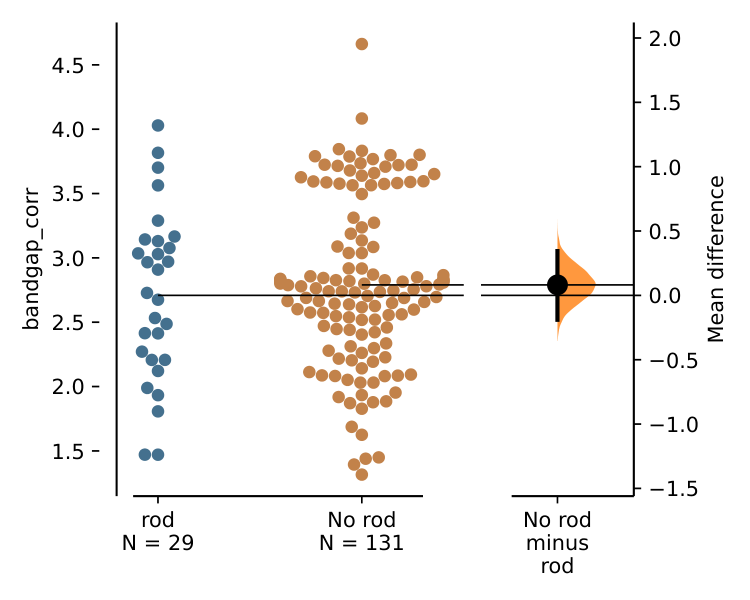

Supplement: SC-016-D5SC01100K-s001 [file SC-016-D5SC01100K-s001.zip › ESI/si_images_lowres/bg_rod.png]

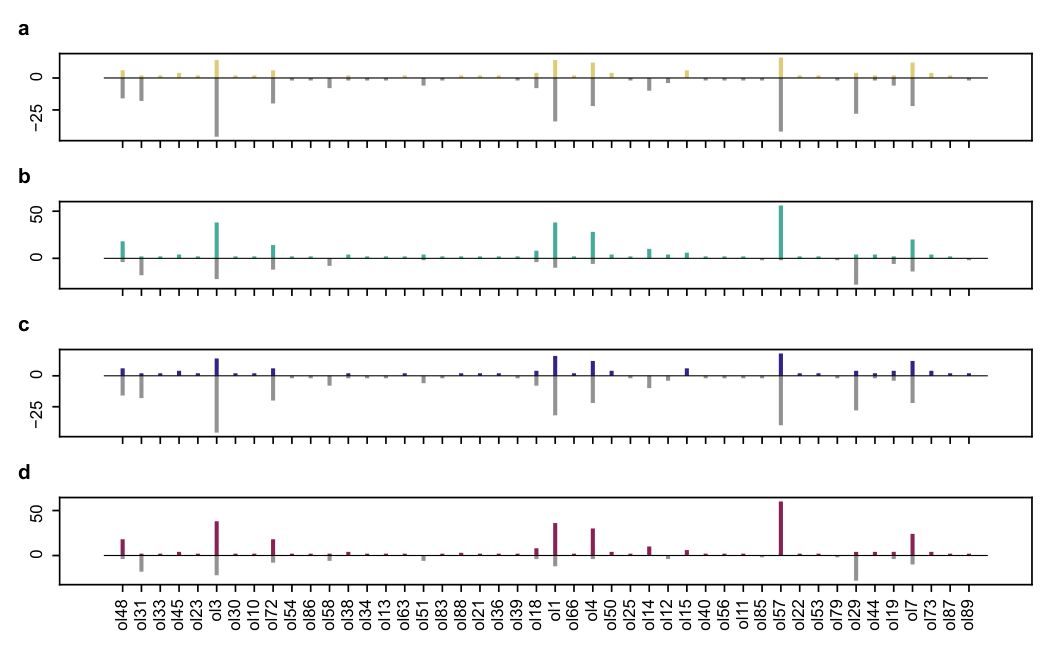

Supplement: SC-016-D5SC01100K-s001 [file SC-016-D5SC01100K-s001.zip › ESI/si_images_lowres/classification_ol.png]

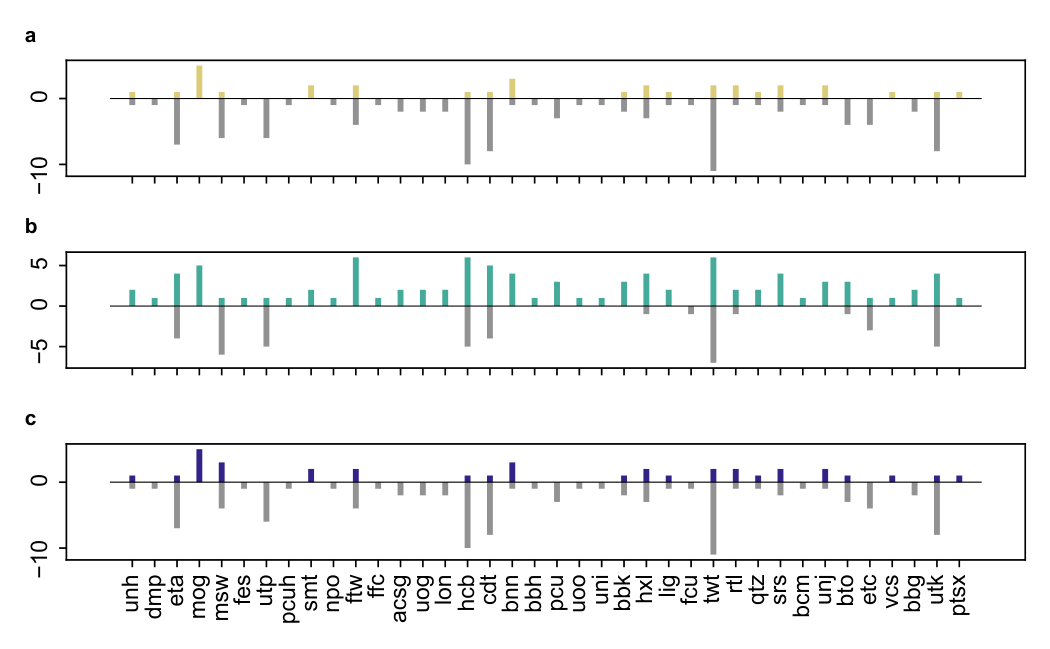

Supplement: SC-016-D5SC01100K-s001 [file SC-016-D5SC01100K-s001.zip › ESI/si_images_lowres/classification_topo.png]

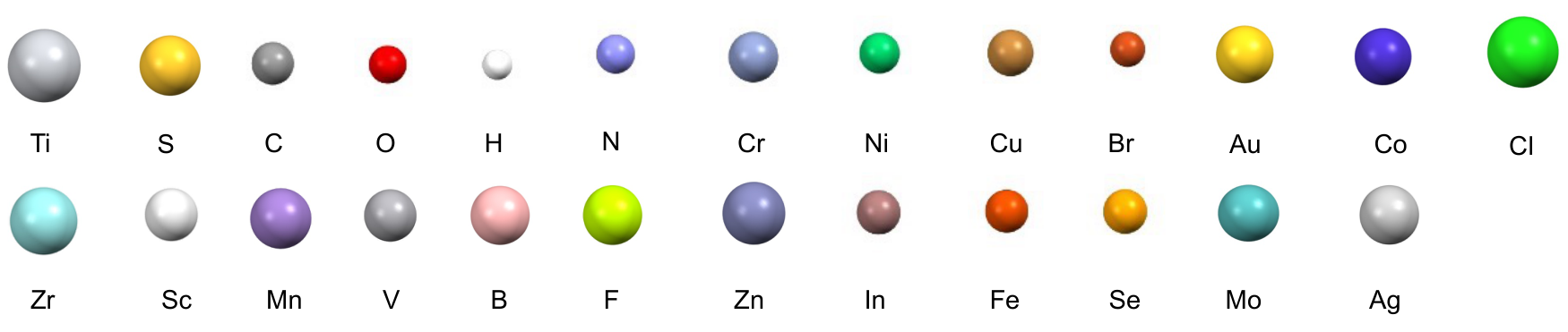

Supplement: SC-016-D5SC01100K-s001 [file SC-016-D5SC01100K-s001.zip › ESI/si_images_lowres/color_code.png]

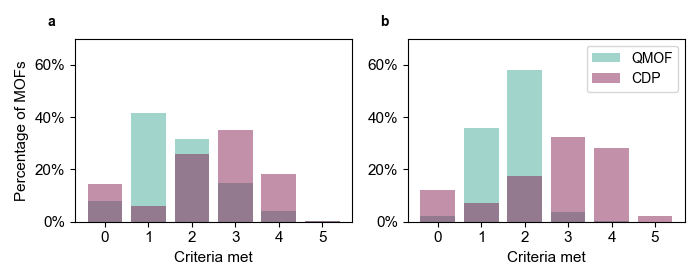

Supplement: SC-016-D5SC01100K-s001 [file SC-016-D5SC01100K-s001.zip › ESI/si_images_lowres/criteria_met.png]

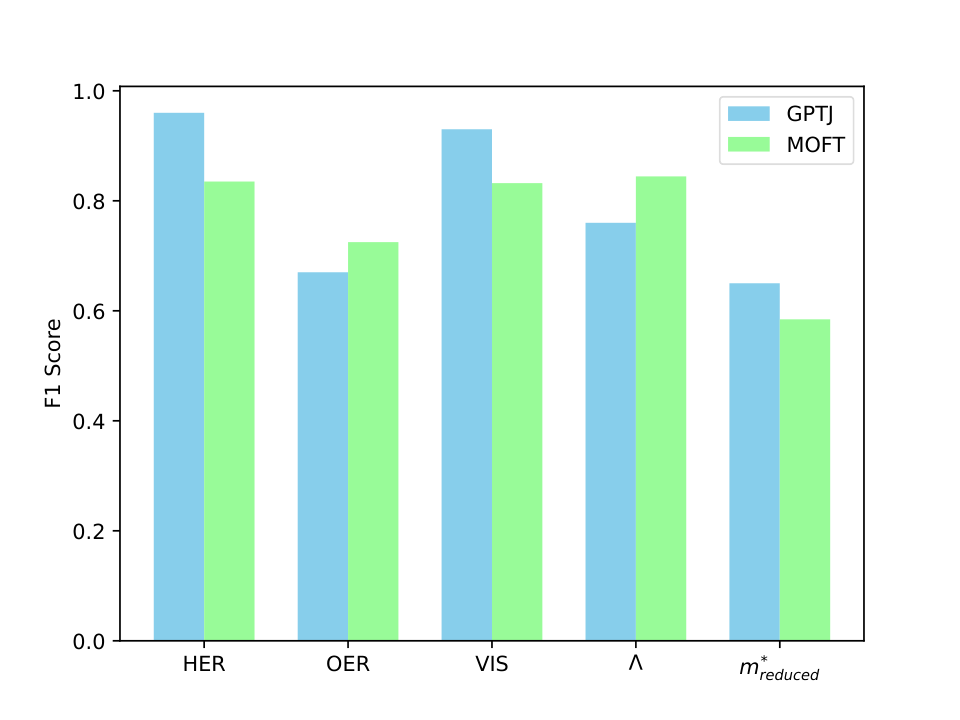

Supplement: SC-016-D5SC01100K-s001 [file SC-016-D5SC01100K-s001.zip › ESI/si_images_lowres/GPTJvsMOFT_F1Score.png]

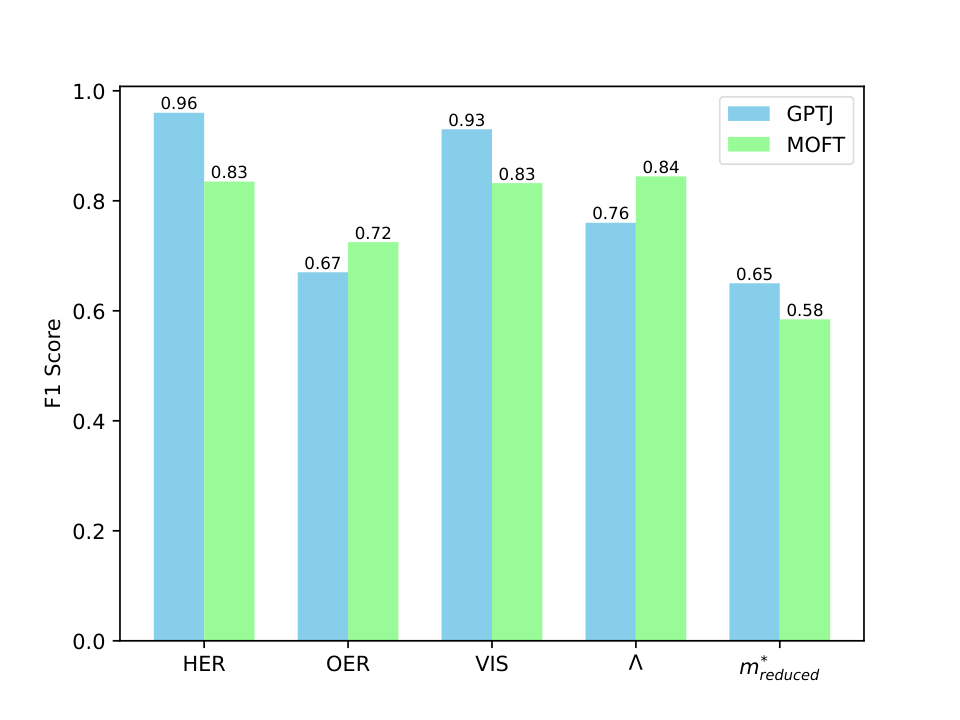

Supplement: SC-016-D5SC01100K-s001 [file SC-016-D5SC01100K-s001.zip › ESI/si_images_lowres/GPTJvsMOFT_F1Score_withValues.png]

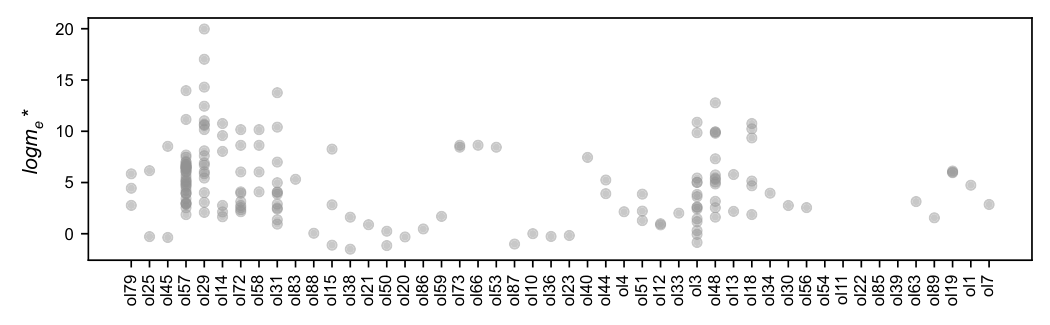

Supplement: SC-016-D5SC01100K-s001 [file SC-016-D5SC01100K-s001.zip › ESI/si_images_lowres/linkers_def_me.png]

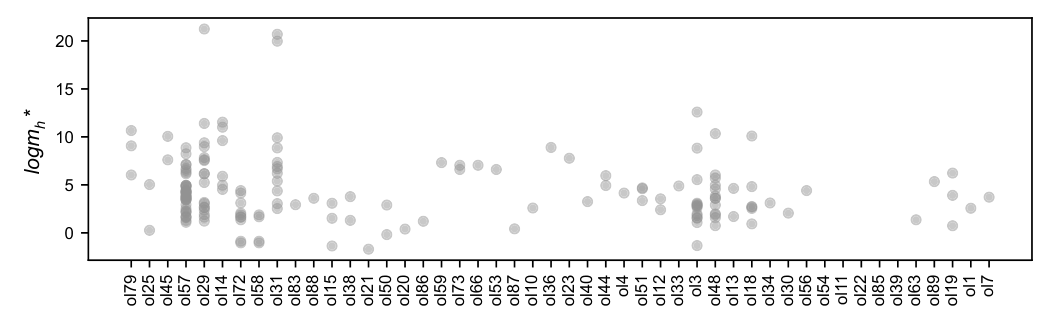

Supplement: SC-016-D5SC01100K-s001 [file SC-016-D5SC01100K-s001.zip › ESI/si_images_lowres/linkers_def_mh.png]
