# Supplementary figures and images for: Exploring the chemical design space of metal–organic frameworks for photocatalysis (part 1 of 2)
Source: Chem Sci. 2025 May 13;16(25):11434–46. doi: 10.1039/d5sc01100k (PMC12107286; doi:10.1039/d5sc01100k)

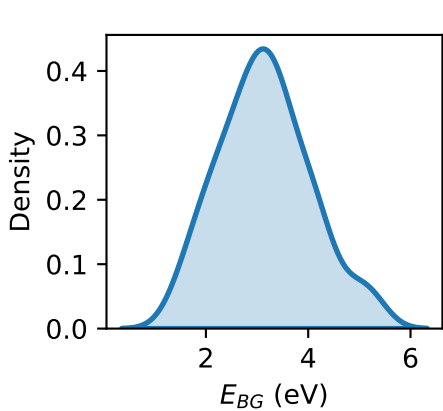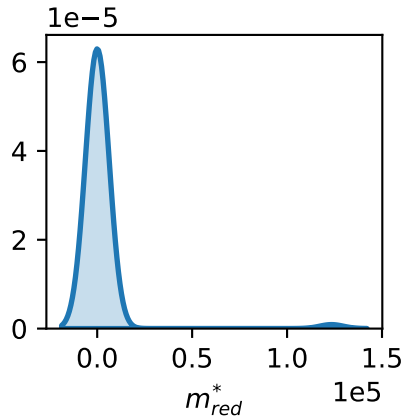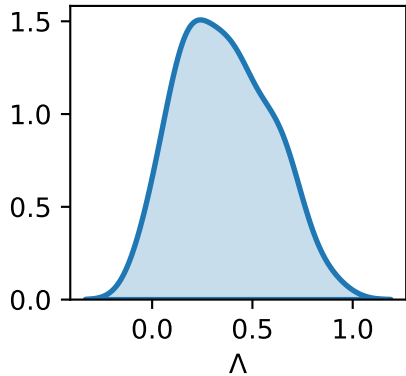

Supplement: SC-016-D5SC01100K-s001 [file SC-016-D5SC01100K-s001.zip › ESI/si_images/all_cs_kde.pdf]

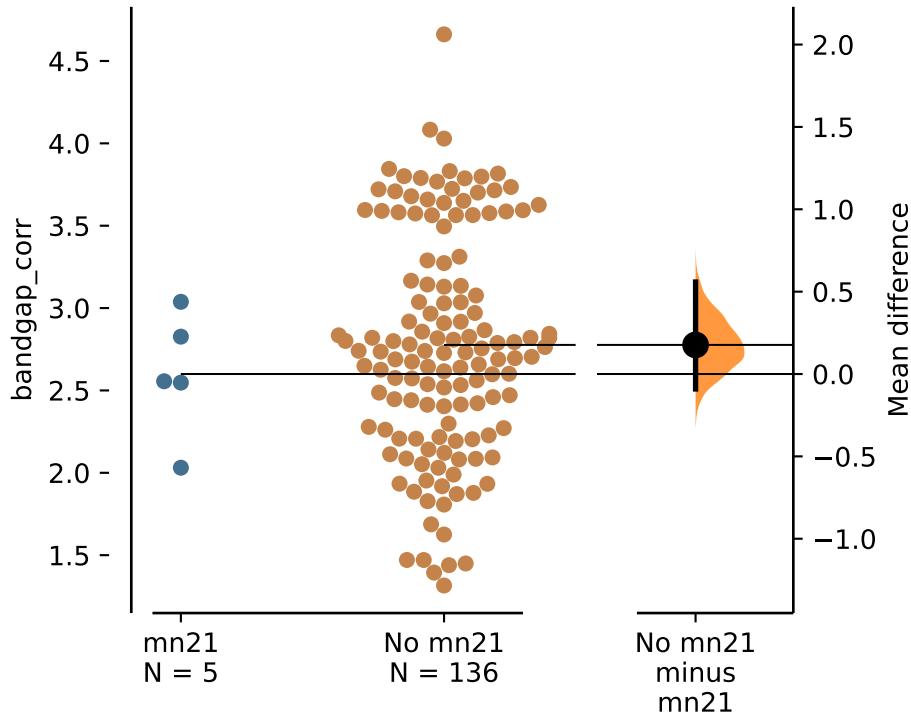

Supplement: SC-016-D5SC01100K-s001 [file SC-016-D5SC01100K-s001.zip › ESI/si_images/bg_mn21.pdf]

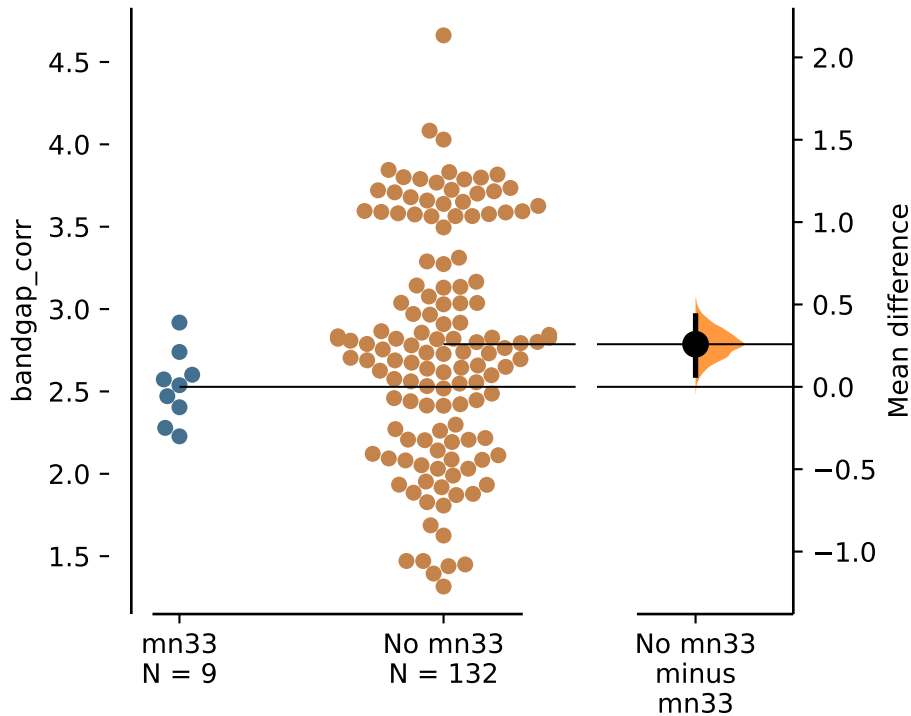

Supplement: SC-016-D5SC01100K-s001 [file SC-016-D5SC01100K-s001.zip › ESI/si_images/bg_mn33.pdf]

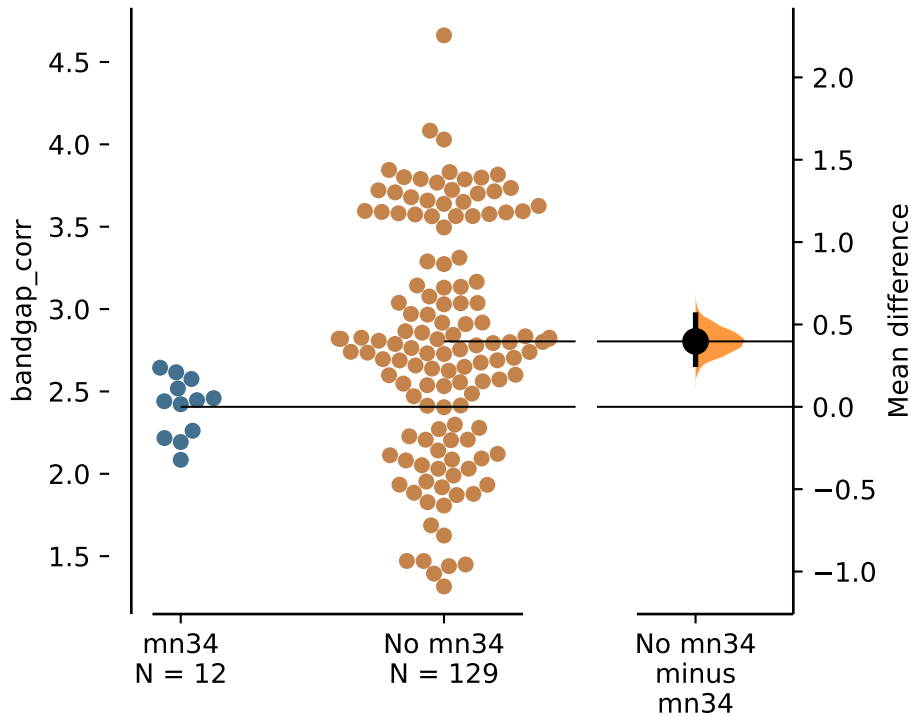

Supplement: SC-016-D5SC01100K-s001 [file SC-016-D5SC01100K-s001.zip › ESI/si_images/bg_mn34.pdf]

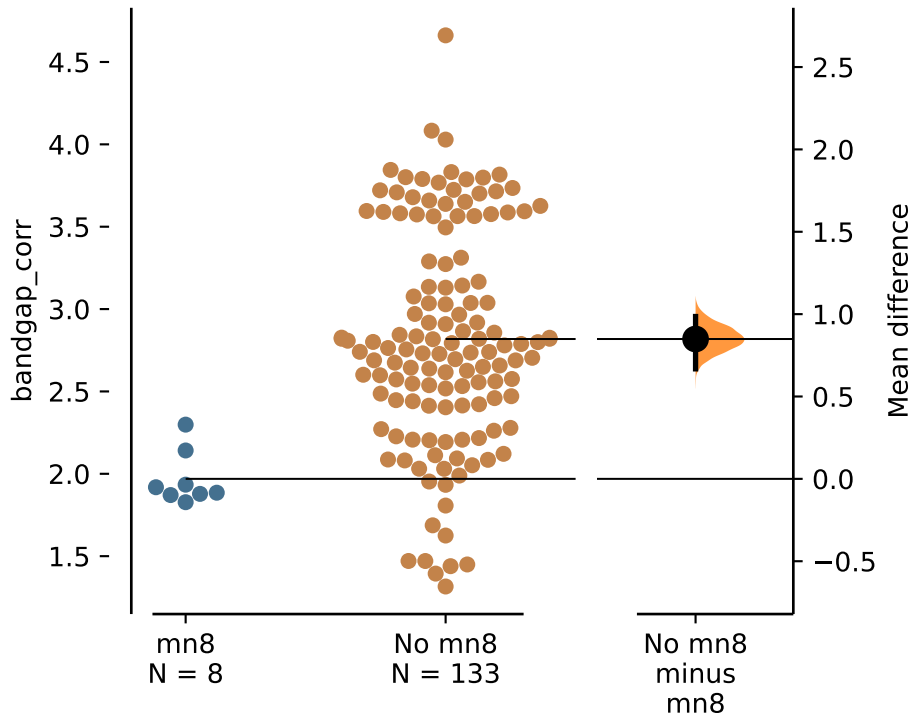

Supplement: SC-016-D5SC01100K-s001 [file SC-016-D5SC01100K-s001.zip › ESI/si_images/bg_mn8.pdf]

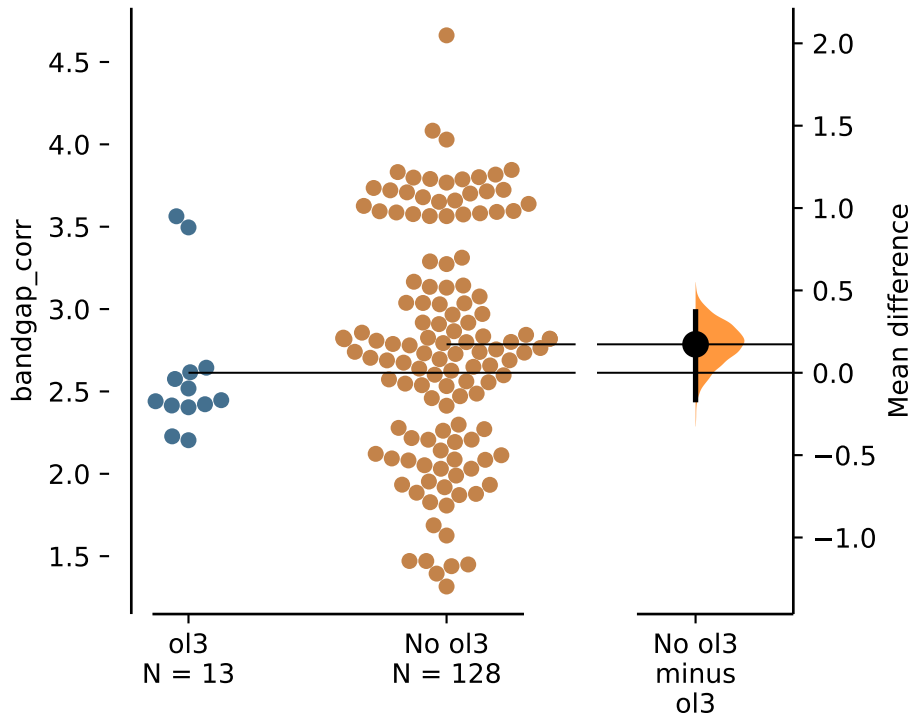

Supplement: SC-016-D5SC01100K-s001 [file SC-016-D5SC01100K-s001.zip › ESI/si_images/bg_ol3.pdf]

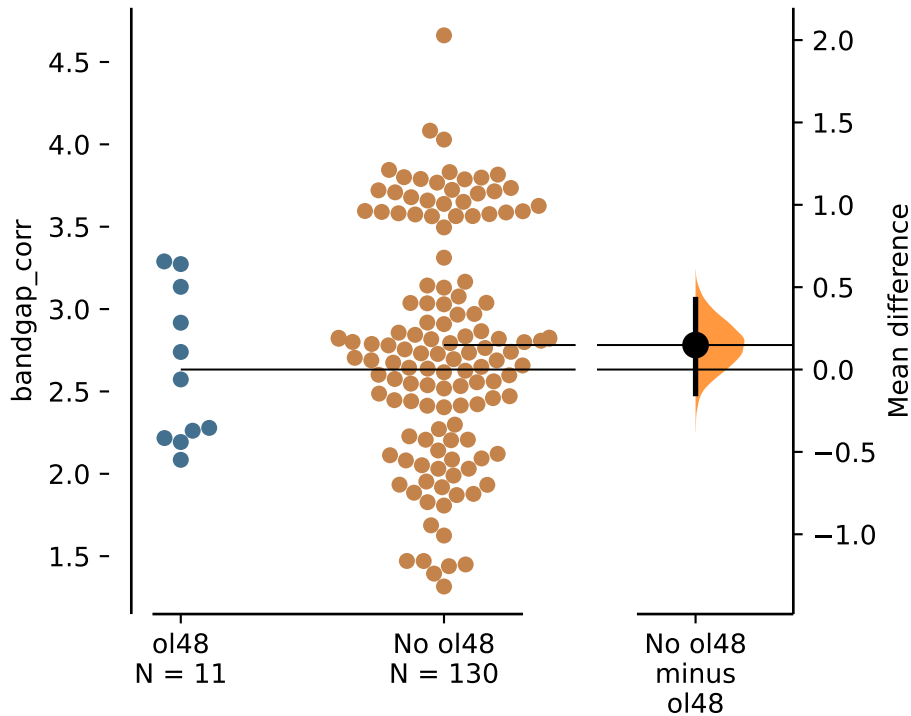

Supplement: SC-016-D5SC01100K-s001 [file SC-016-D5SC01100K-s001.zip › ESI/si_images/bg_ol48.pdf]

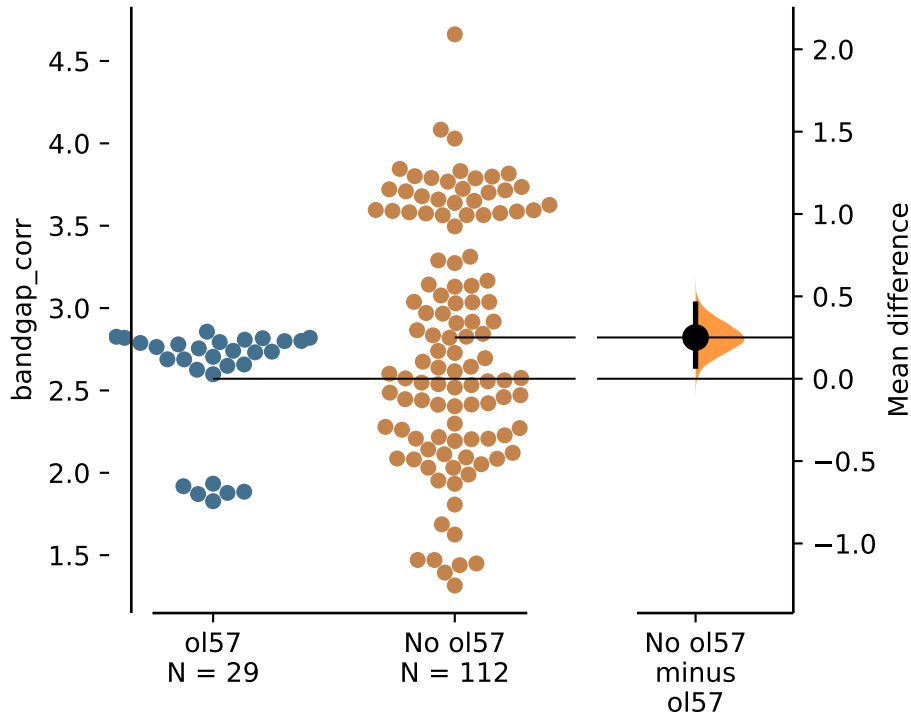

Supplement: SC-016-D5SC01100K-s001 [file SC-016-D5SC01100K-s001.zip › ESI/si_images/bg_ol57.pdf]

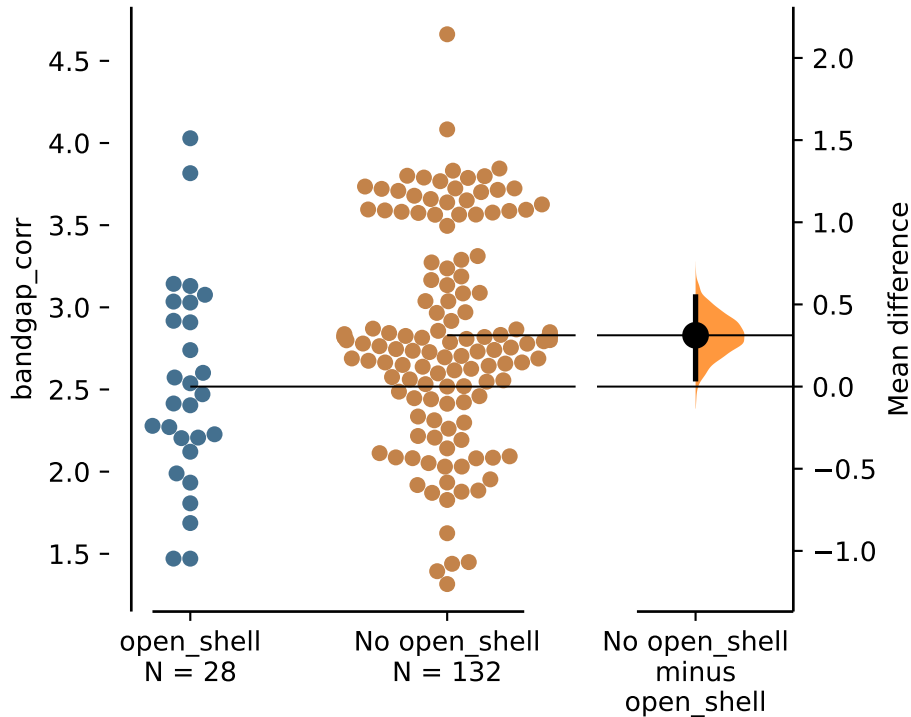

Supplement: SC-016-D5SC01100K-s001 [file SC-016-D5SC01100K-s001.zip › ESI/si_images/bg_open_shell.pdf]

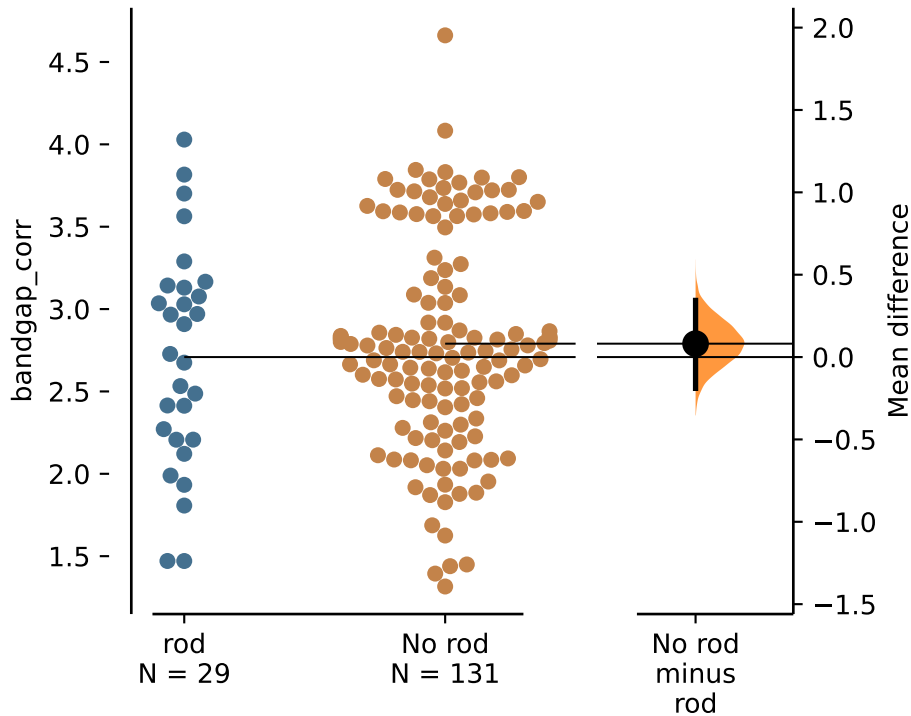

Supplement: SC-016-D5SC01100K-s001 [file SC-016-D5SC01100K-s001.zip › ESI/si_images/bg_rod.pdf]

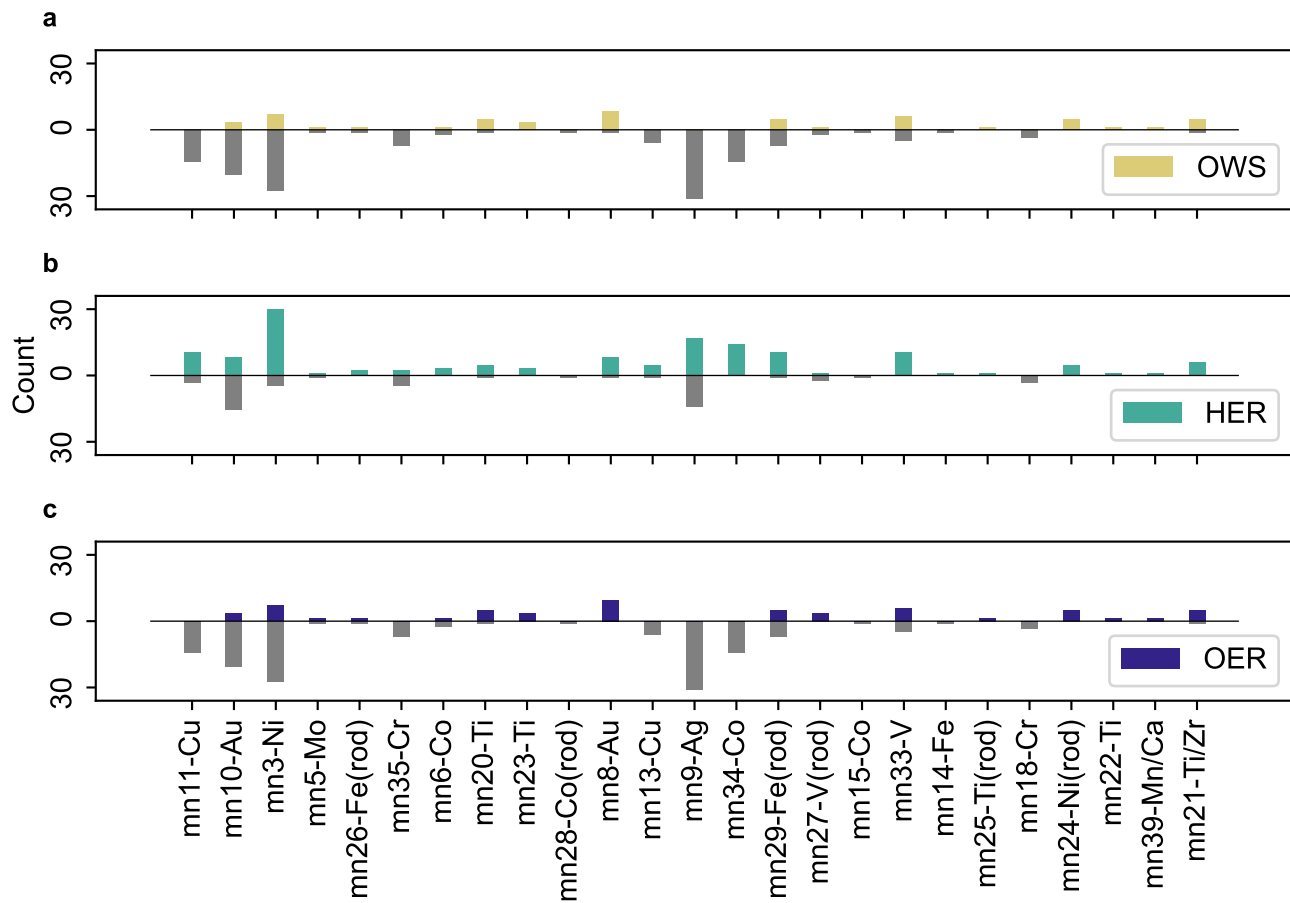

Supplement: SC-016-D5SC01100K-s001 [file SC-016-D5SC01100K-s001.zip › ESI/si_images/classification_mn.pdf]

**a**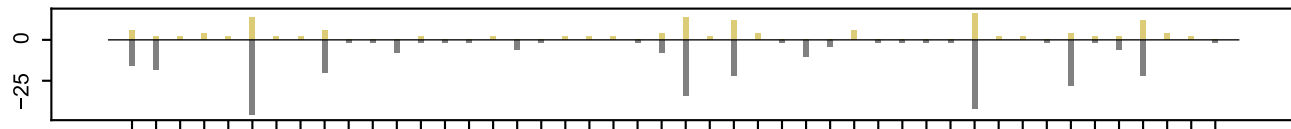**b**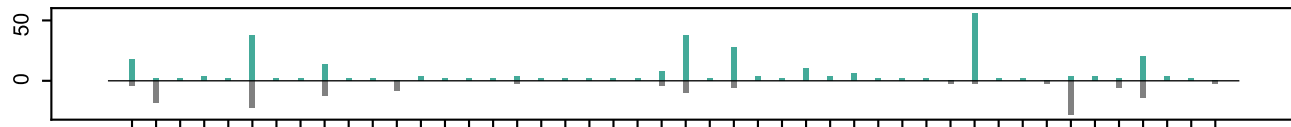**c**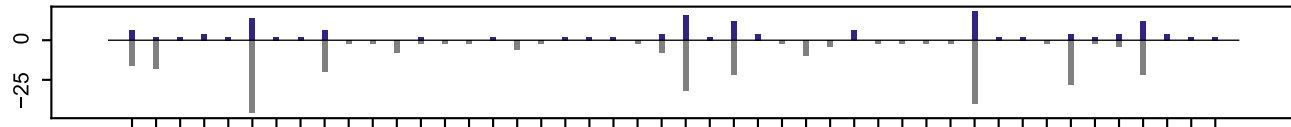**d**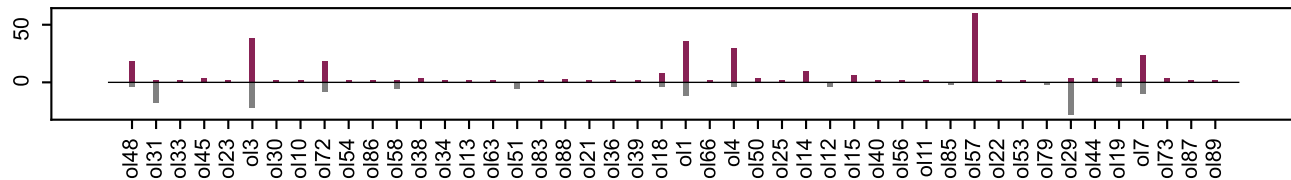

Supplement: SC-016-D5SC01100K-s001 [file SC-016-D5SC01100K-s001.zip › ESI/si_images/classification_ol.pdf]

**a**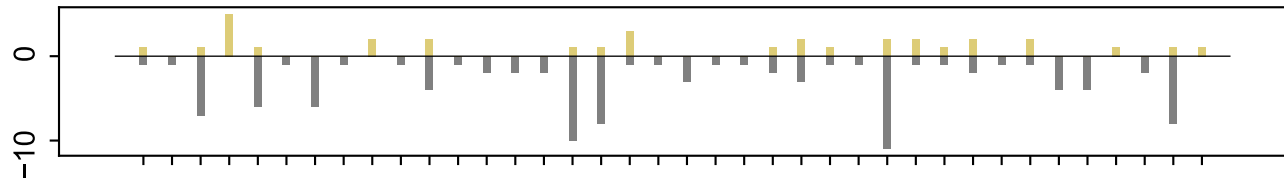**b**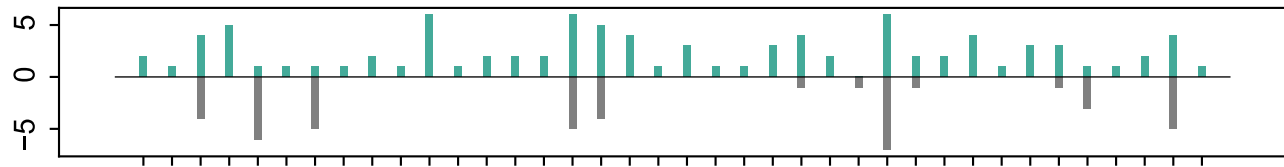**c**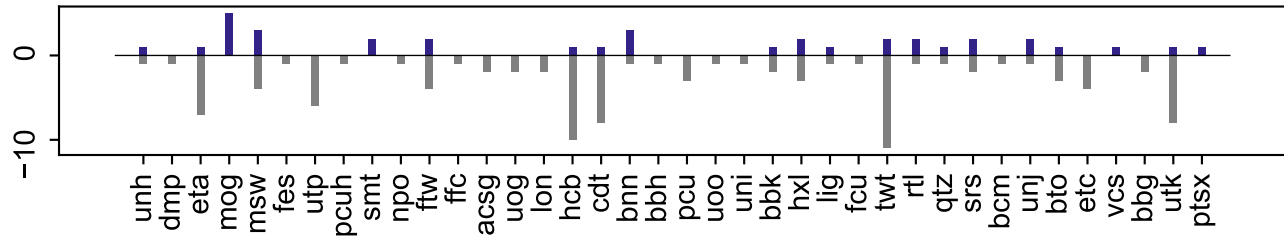

Supplement: SC-016-D5SC01100K-s001 [file SC-016-D5SC01100K-s001.zip › ESI/si_images/classification_topo.pdf]

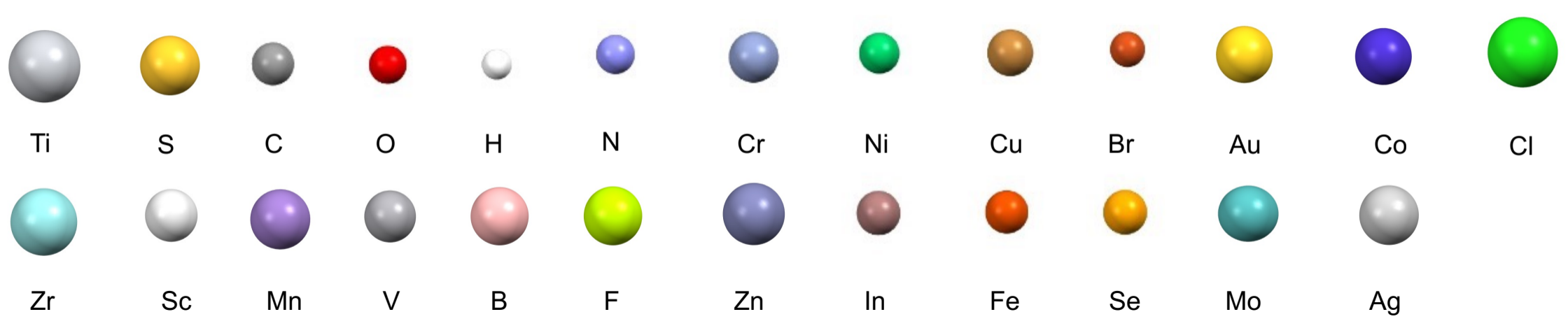

Supplement: SC-016-D5SC01100K-s001 [file SC-016-D5SC01100K-s001.zip › ESI/si_images/color_code.pdf]

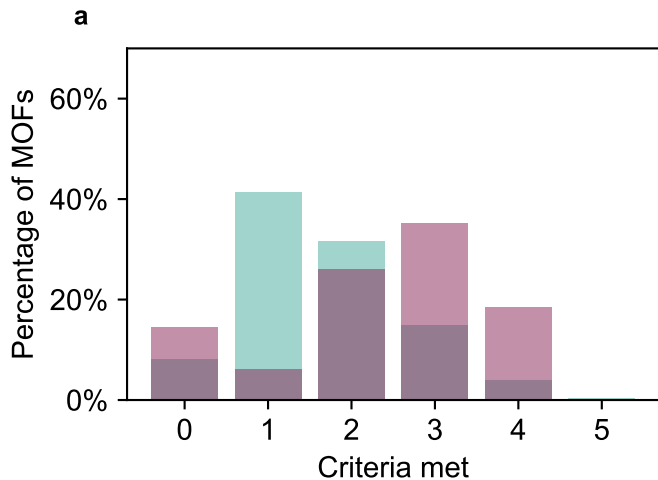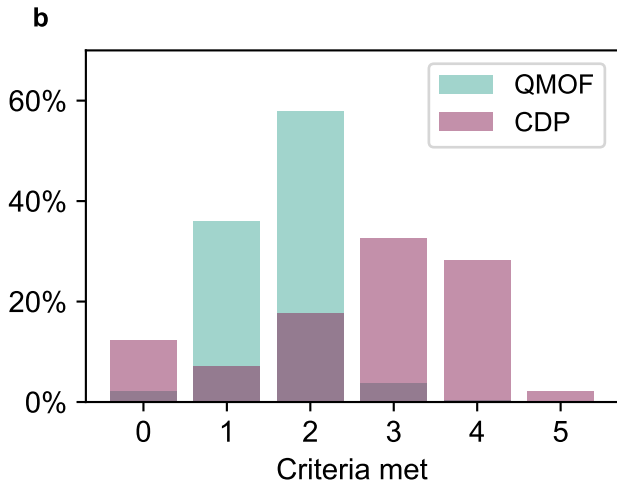

Supplement: SC-016-D5SC01100K-s001 [file SC-016-D5SC01100K-s001.zip › ESI/si_images/criteria_met.pdf]

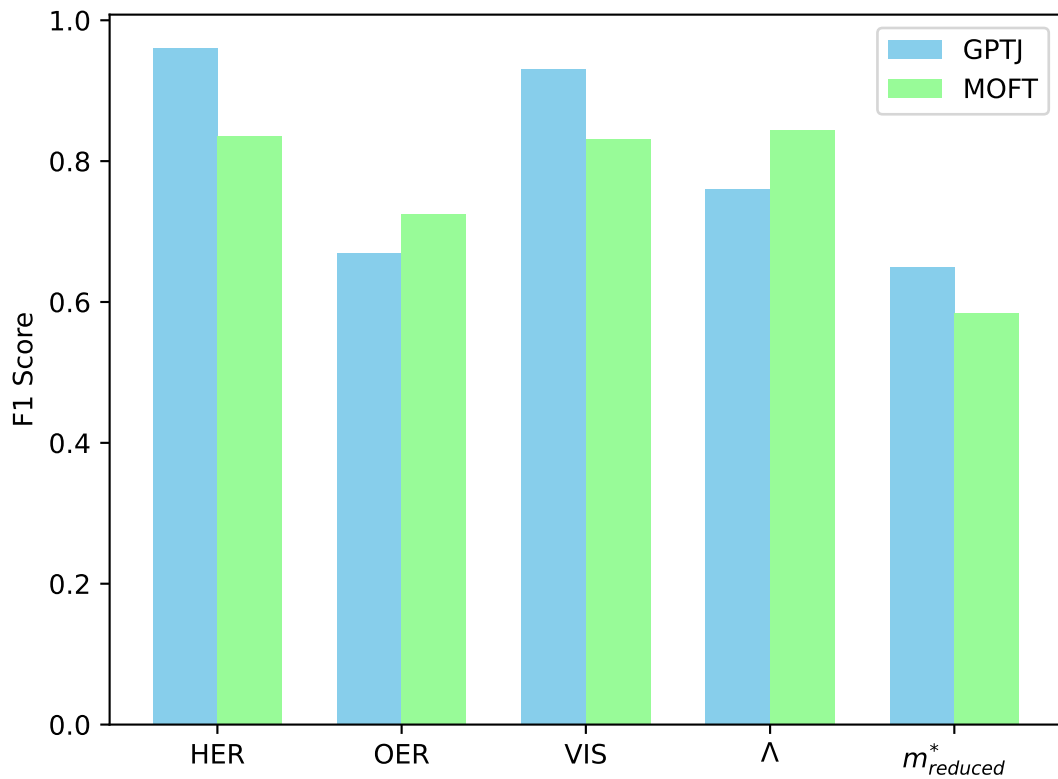

Supplement: SC-016-D5SC01100K-s001 [file SC-016-D5SC01100K-s001.zip › ESI/si_images/GPTJvsMOFT_F1Score.pdf]

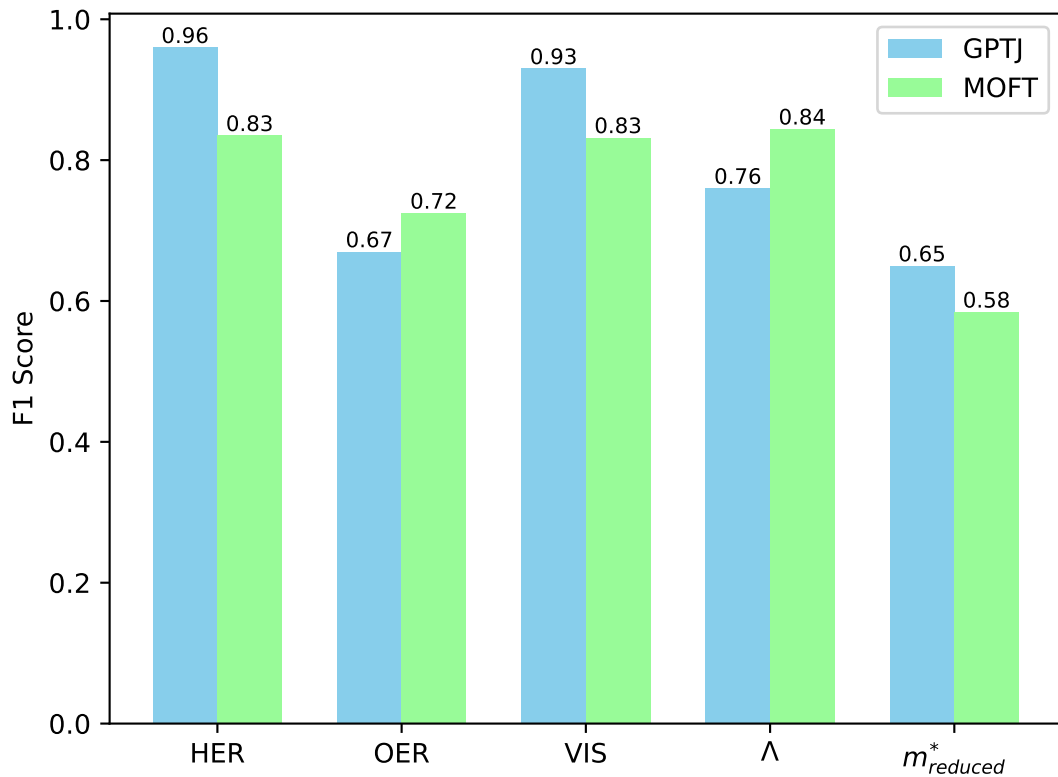

Supplement: SC-016-D5SC01100K-s001 [file SC-016-D5SC01100K-s001.zip › ESI/si_images/GPTJvsMOFT_F1Score_withValues.pdf]

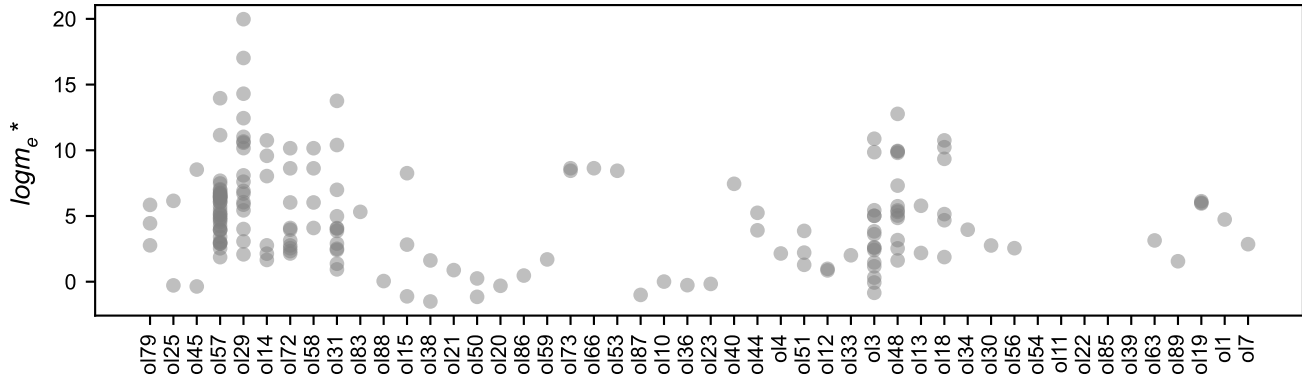

Supplement: SC-016-D5SC01100K-s001 [file SC-016-D5SC01100K-s001.zip › ESI/si_images/linkers_def_me.pdf]

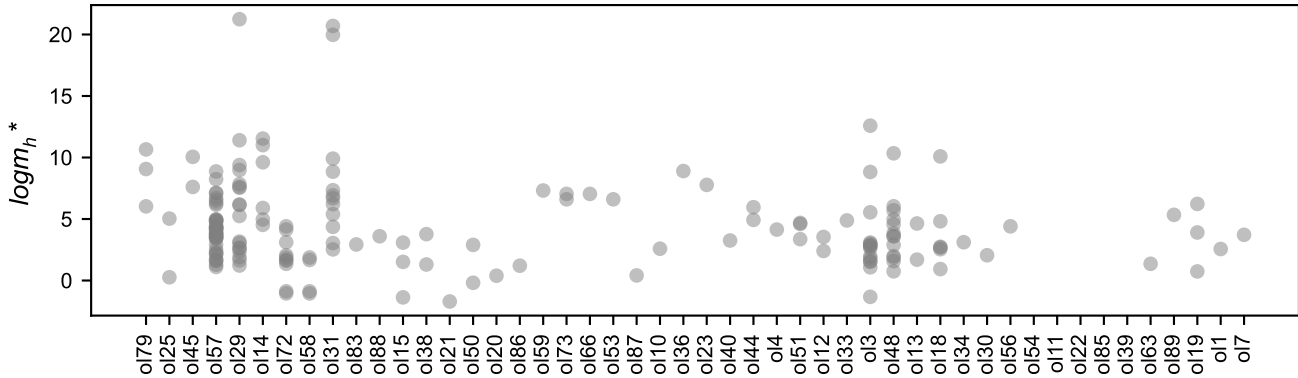

Supplement: SC-016-D5SC01100K-s001 [file SC-016-D5SC01100K-s001.zip › ESI/si_images/linkers_def_mh.pdf]

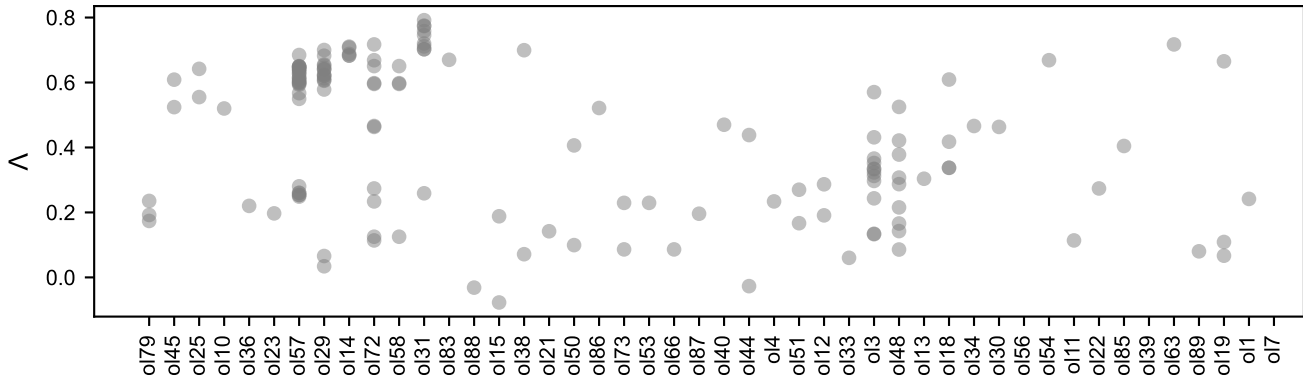

Supplement: SC-016-D5SC01100K-s001 [file SC-016-D5SC01100K-s001.zip › ESI/si_images/linkers_def_ovlp.pdf]

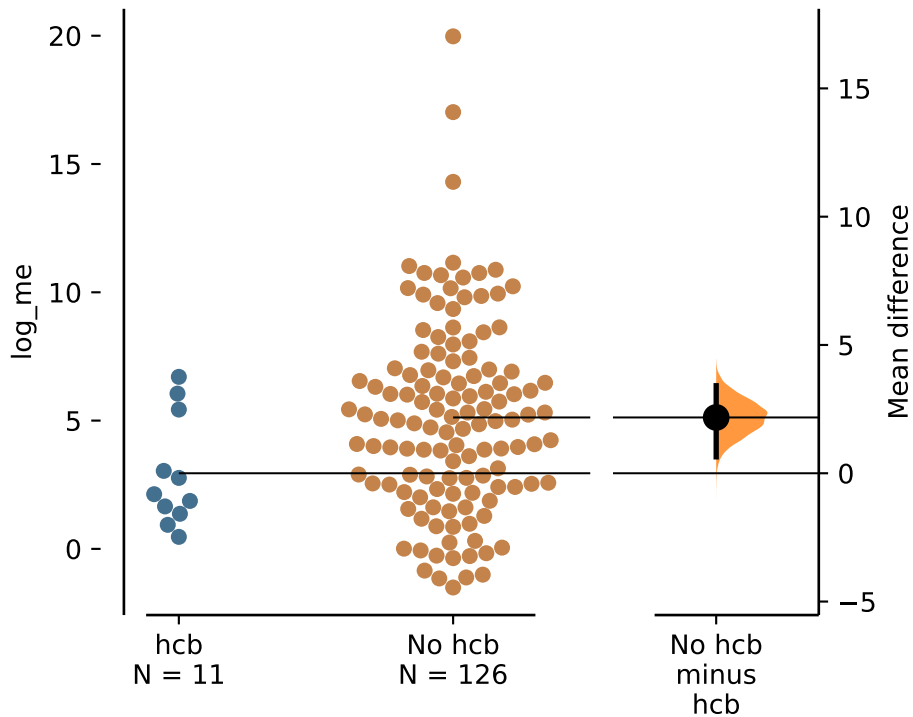

Supplement: SC-016-D5SC01100K-s001 [file SC-016-D5SC01100K-s001.zip › ESI/si_images/me_hcb.pdf]

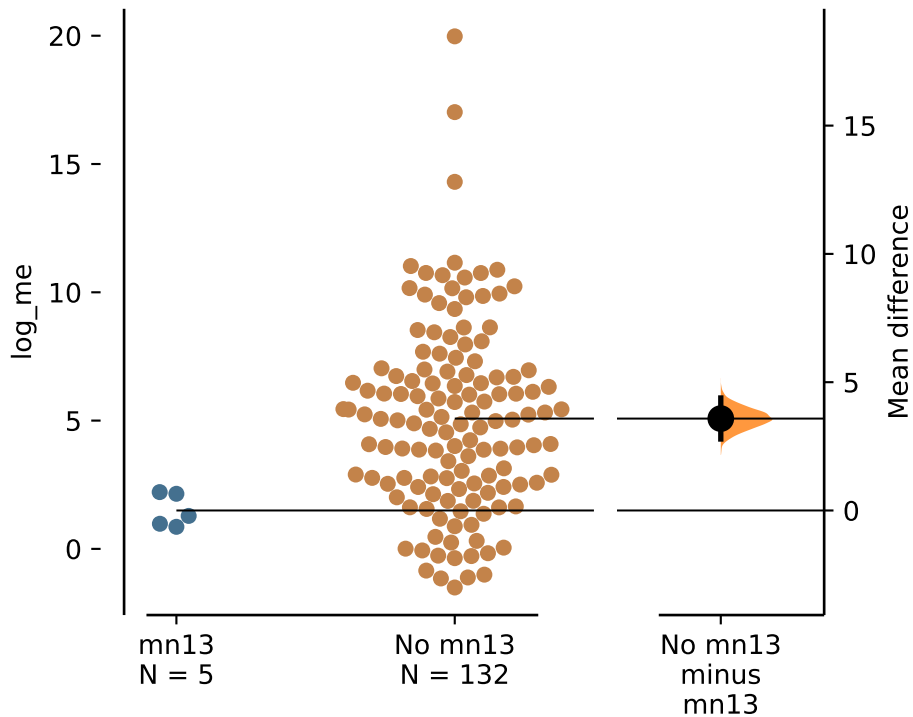

Supplement: SC-016-D5SC01100K-s001 [file SC-016-D5SC01100K-s001.zip › ESI/si_images/me_mn13.pdf]

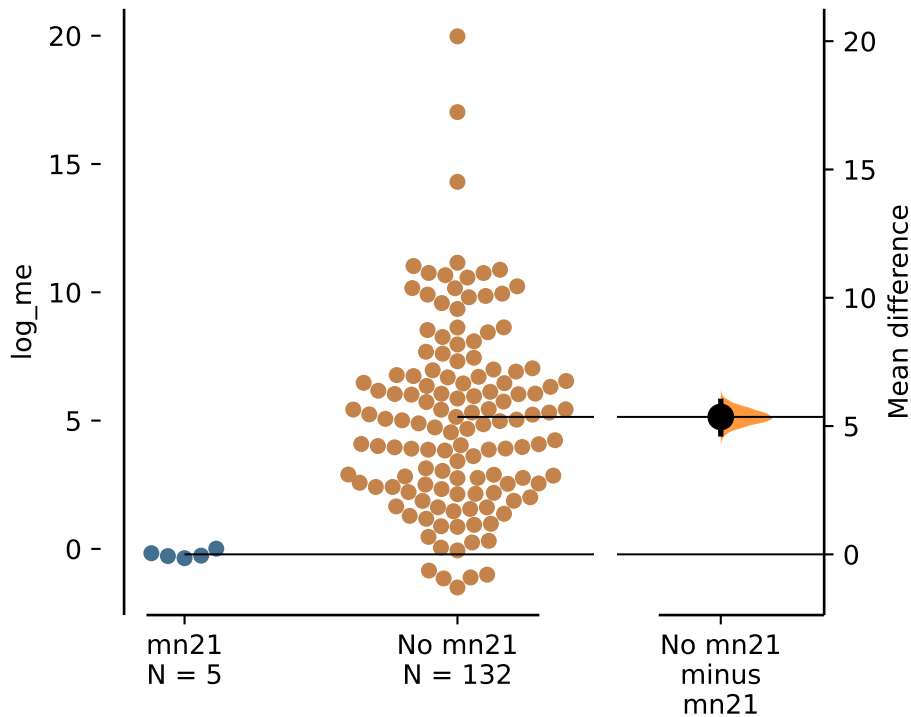

Supplement: SC-016-D5SC01100K-s001 [file SC-016-D5SC01100K-s001.zip › ESI/si_images/me_mn21.pdf]

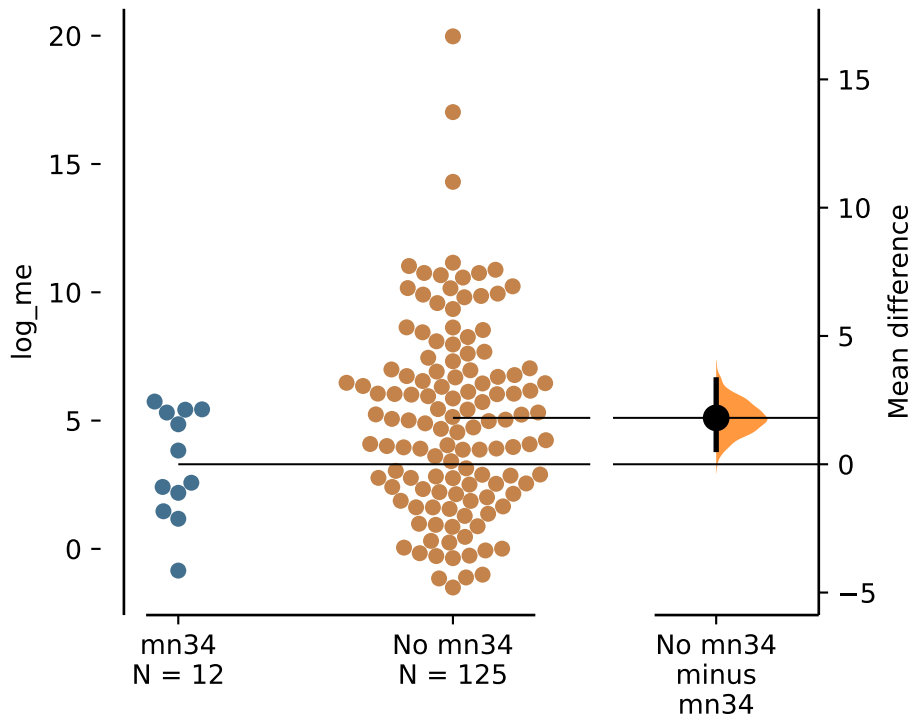

Supplement: SC-016-D5SC01100K-s001 [file SC-016-D5SC01100K-s001.zip › ESI/si_images/me_mn34.pdf]

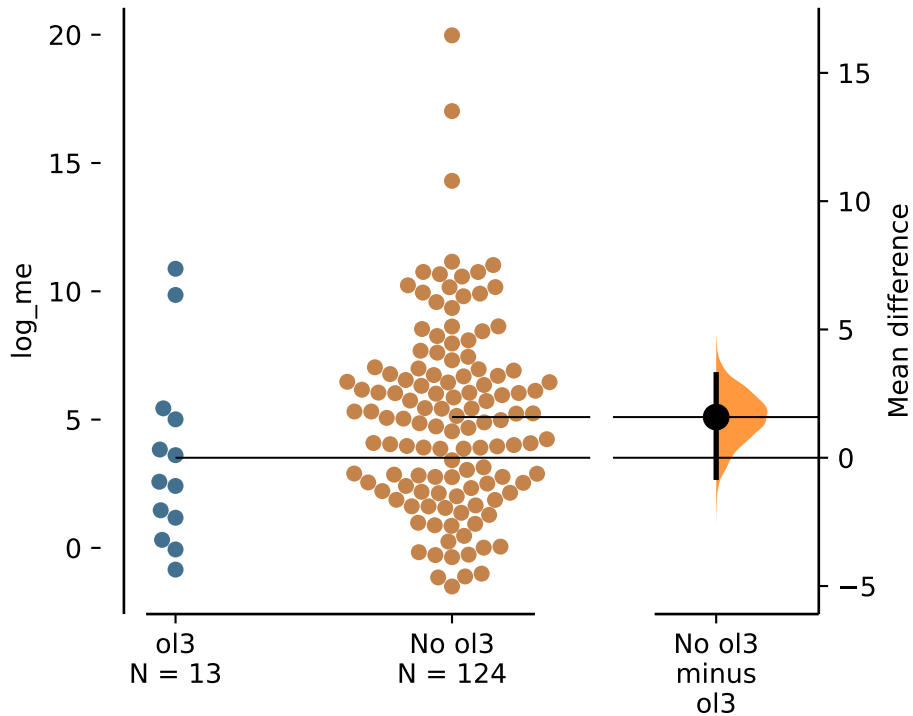

Supplement: SC-016-D5SC01100K-s001 [file SC-016-D5SC01100K-s001.zip › ESI/si_images/me_ol3.pdf]

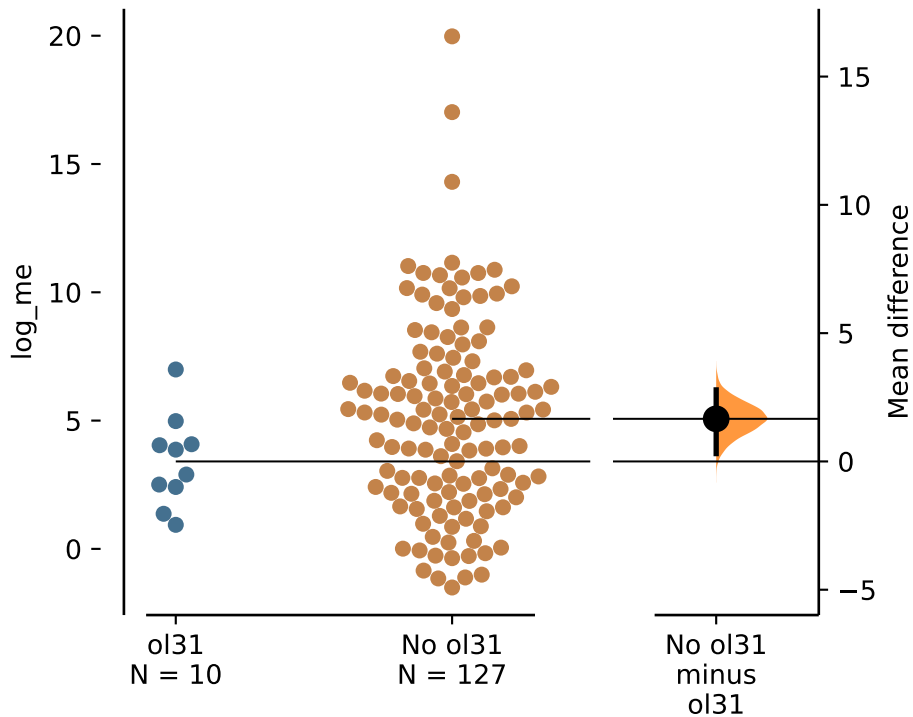

Supplement: SC-016-D5SC01100K-s001 [file SC-016-D5SC01100K-s001.zip › ESI/si_images/me_ol31.pdf]

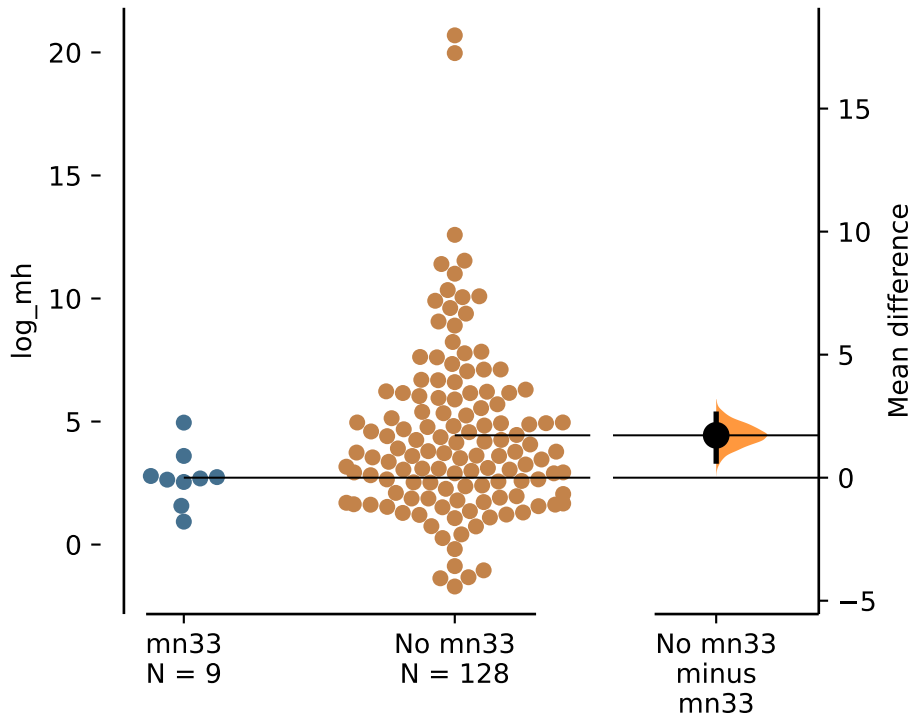

Supplement: SC-016-D5SC01100K-s001 [file SC-016-D5SC01100K-s001.zip › ESI/si_images/mh_mn33.pdf]

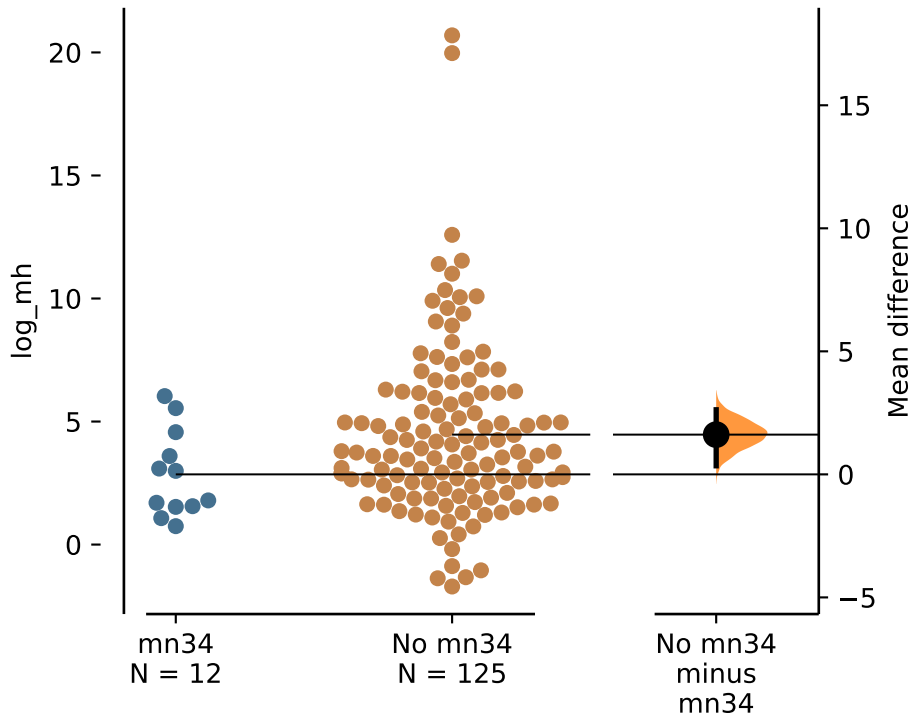

Supplement: SC-016-D5SC01100K-s001 [file SC-016-D5SC01100K-s001.zip › ESI/si_images/mh_mn34.pdf]

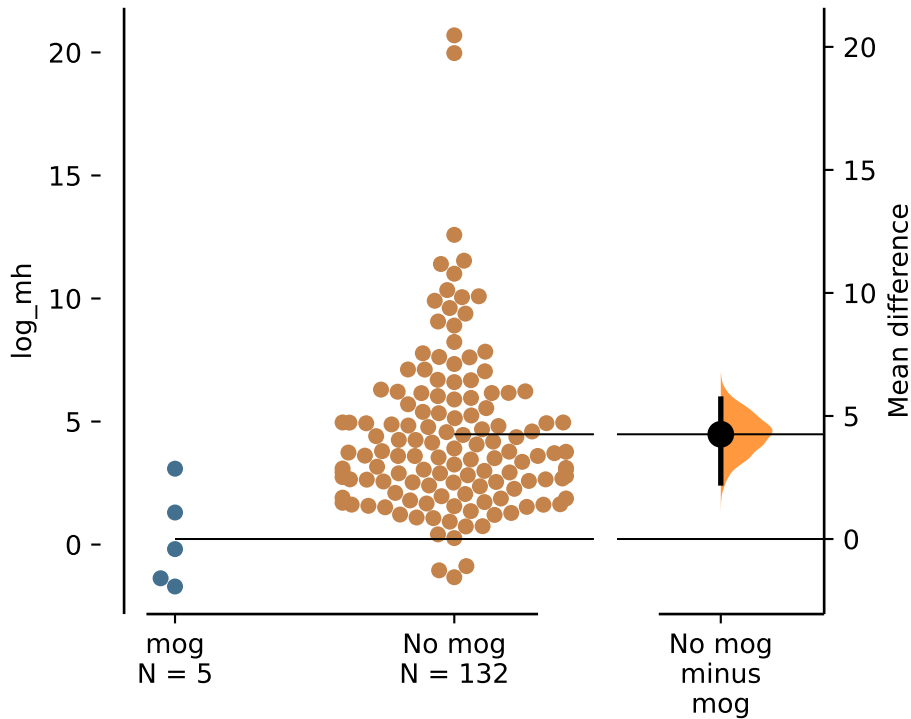

Supplement: SC-016-D5SC01100K-s001 [file SC-016-D5SC01100K-s001.zip › ESI/si_images/mh_mog.pdf]

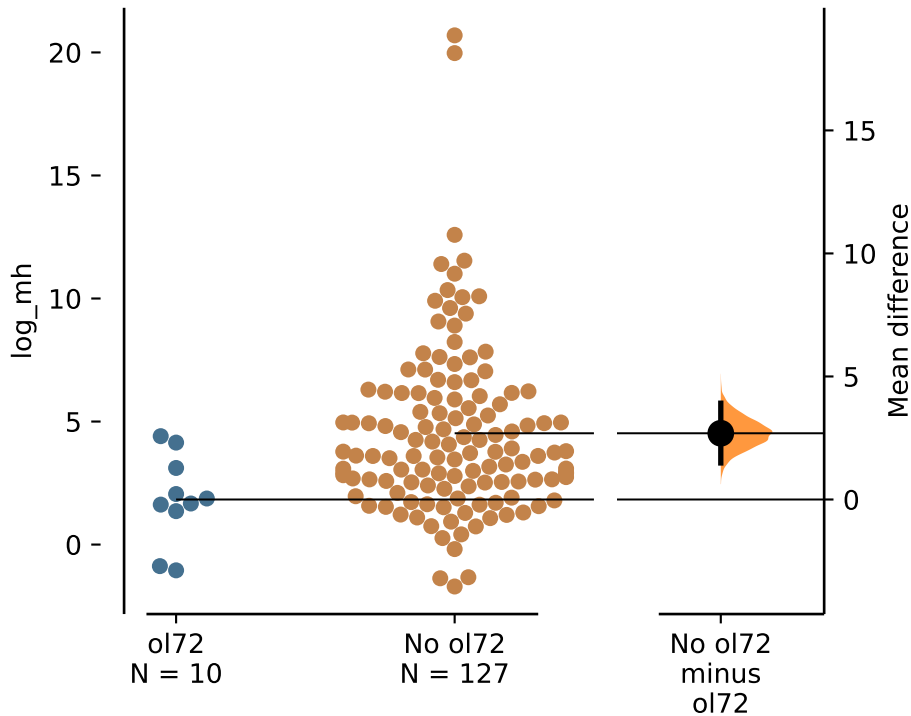

Supplement: SC-016-D5SC01100K-s001 [file SC-016-D5SC01100K-s001.zip › ESI/si_images/mh_ol72.pdf]

GPT-J

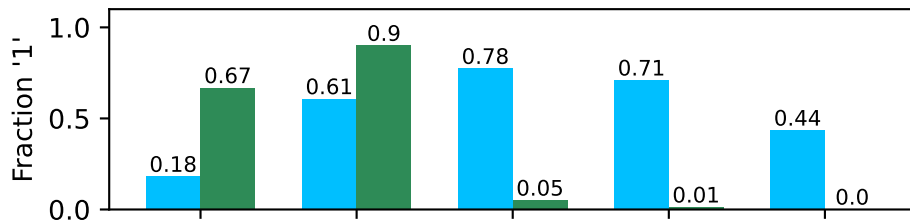

MOFTransformer

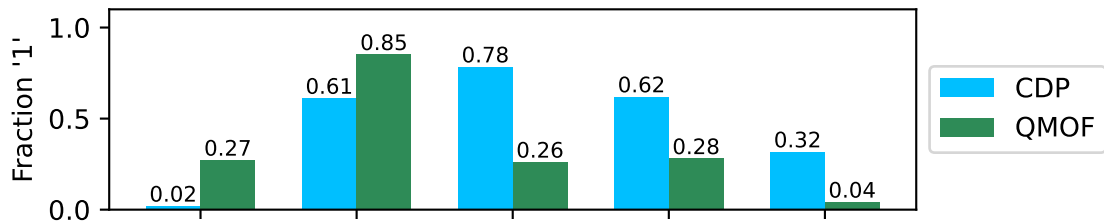

Agreement

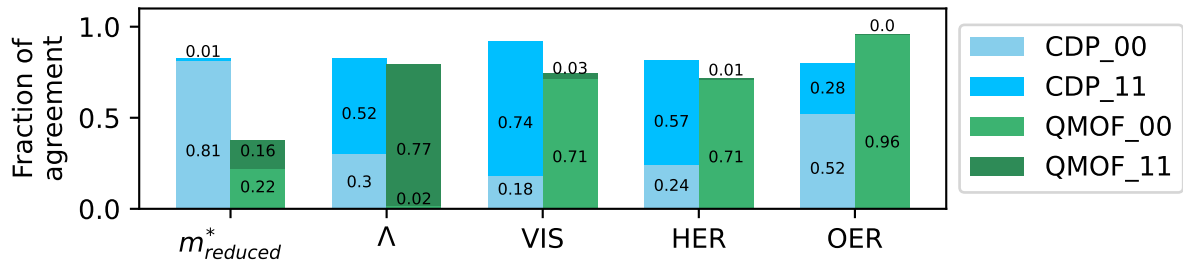

Supplement: SC-016-D5SC01100K-s001 [file SC-016-D5SC01100K-s001.zip › ESI/si_images/ml.pdf]

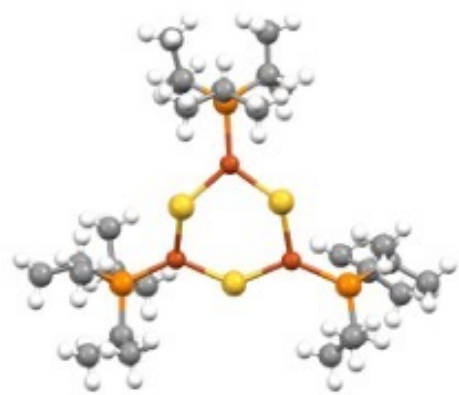

mn1

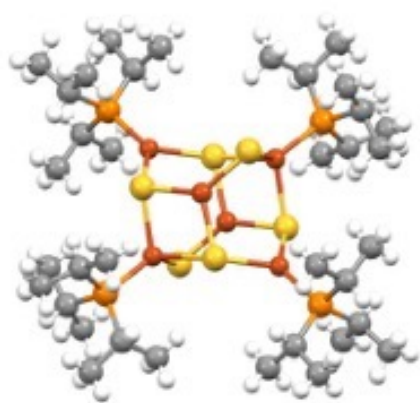

mn2

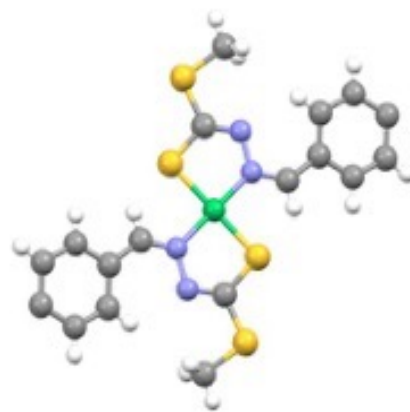

mn3

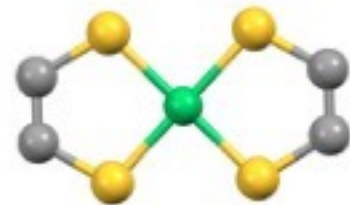

mn4

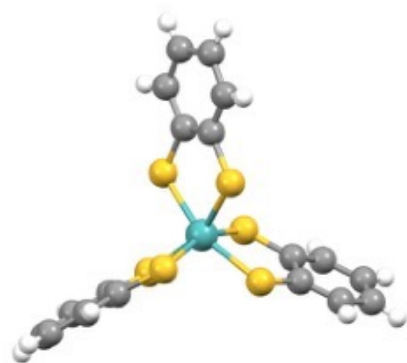

mn5

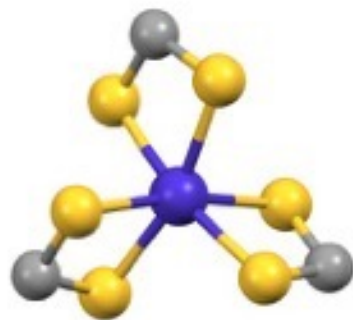

mn6

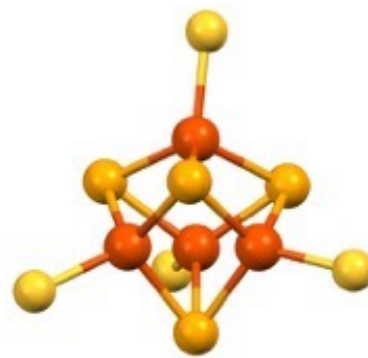

mn7

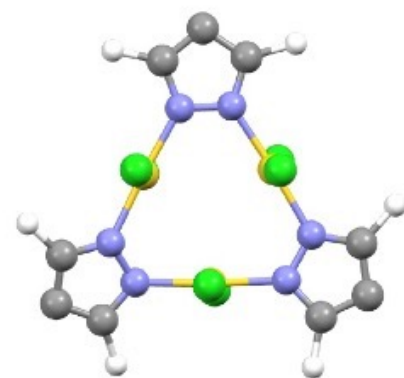

mn8

Supplement: SC-016-D5SC01100K-s001 [file SC-016-D5SC01100K-s001.zip › ESI/si_images/mn1-8.pdf]

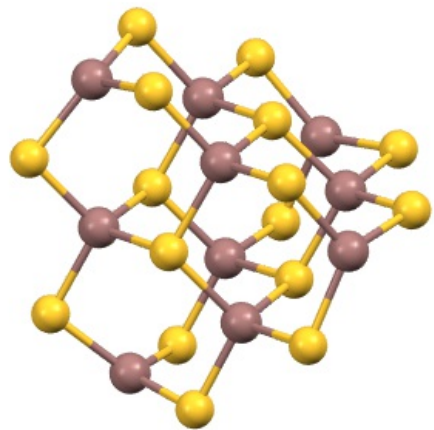

mn17

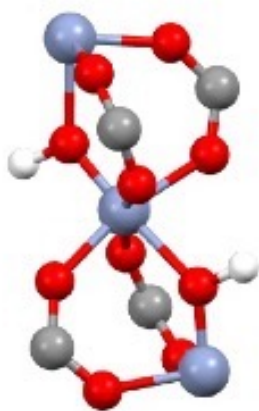

mn18

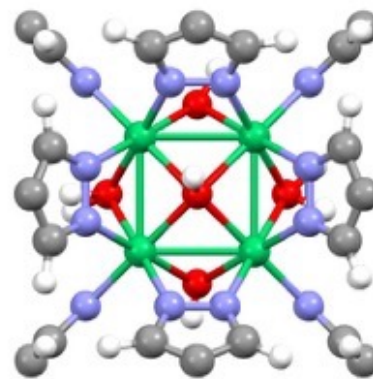

mn19

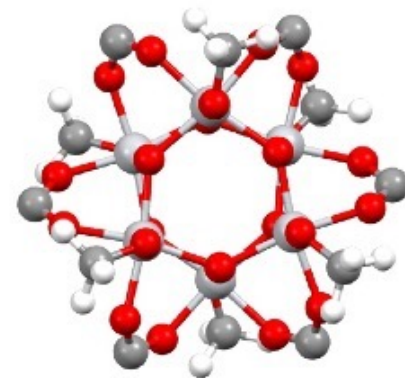

mn20

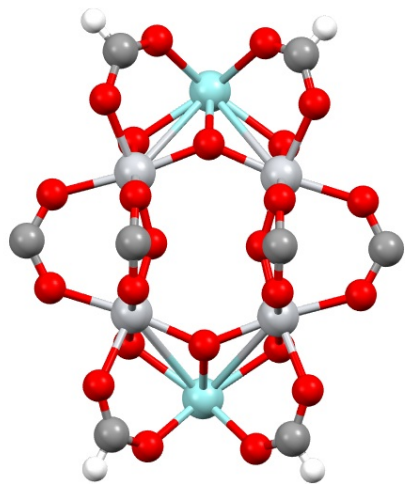

mn21

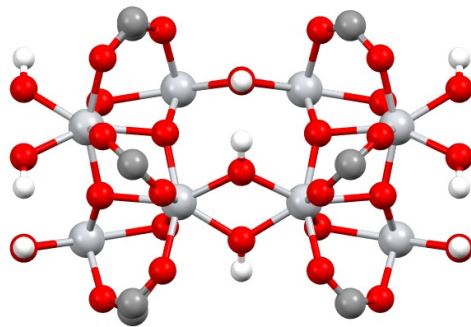

mn22

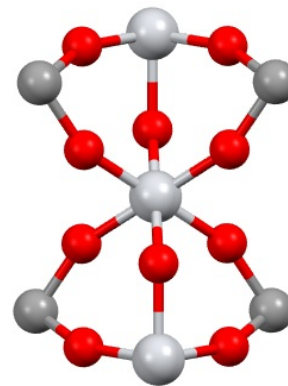

mn23

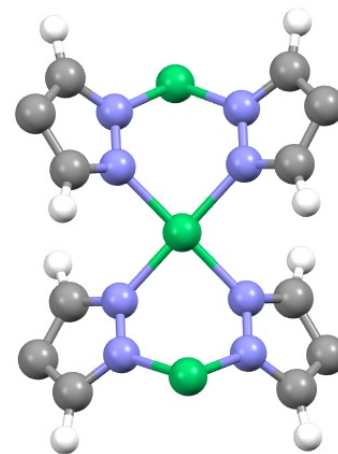

mn24

Supplement: SC-016-D5SC01100K-s001 [file SC-016-D5SC01100K-s001.zip › ESI/si_images/mn17-24.pdf]

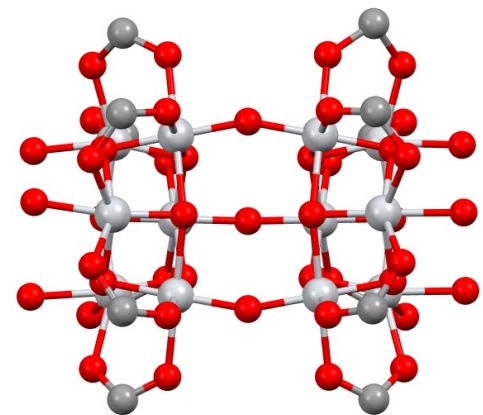

mn25

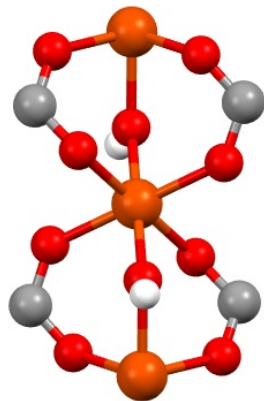

mn26

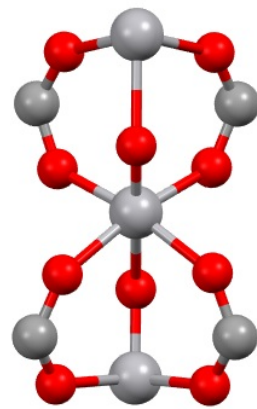

mn27

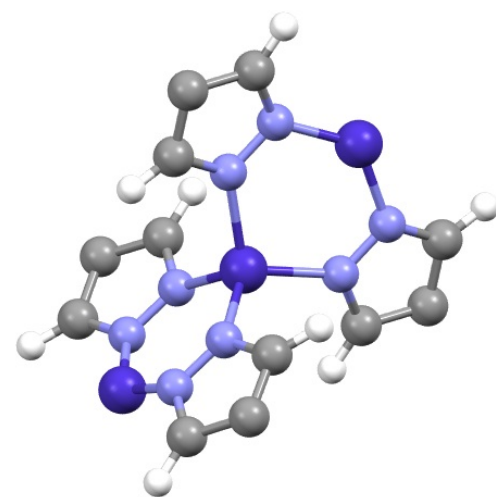

mn28

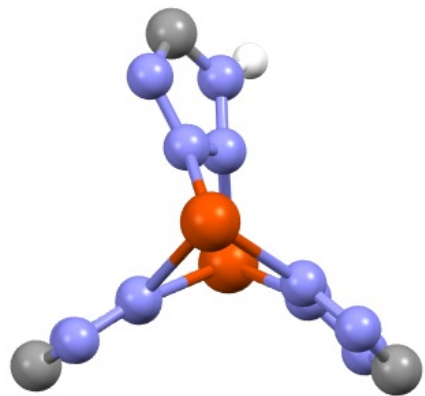

mn29

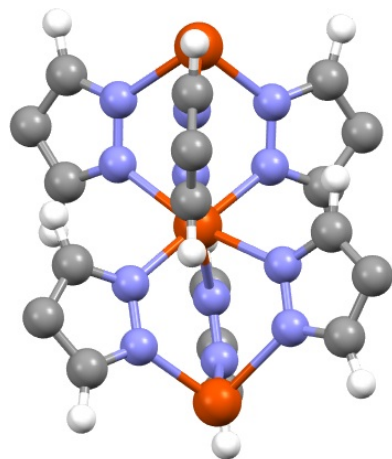

mn30

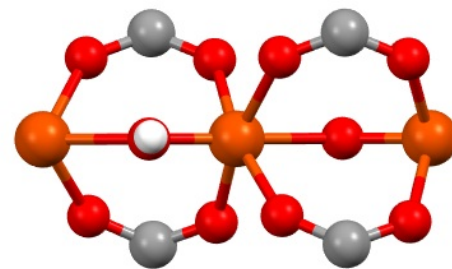

mn31

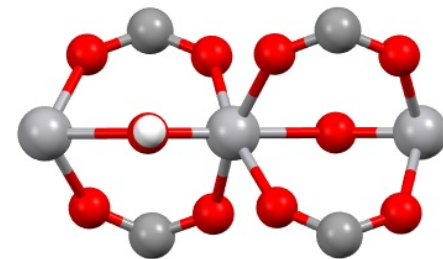

mn32

Supplement: SC-016-D5SC01100K-s001 [file SC-016-D5SC01100K-s001.zip › ESI/si_images/mn25-32.pdf]

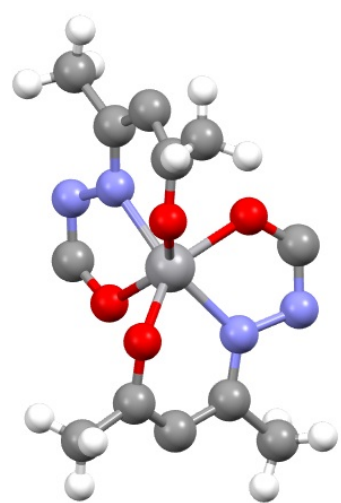

mn33

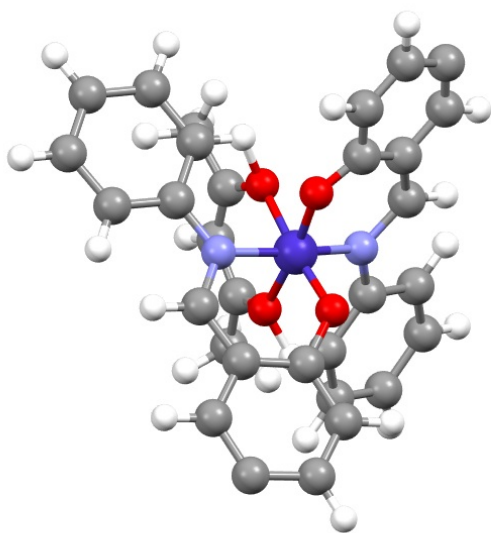

mn34

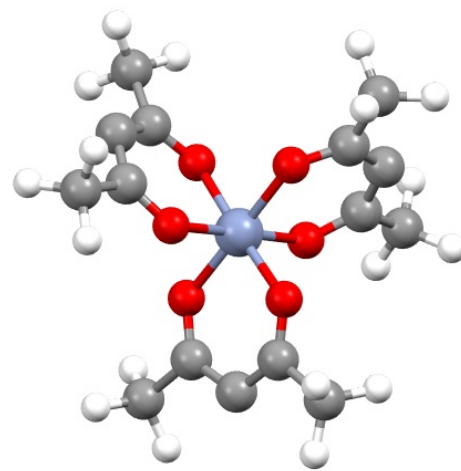

mn35

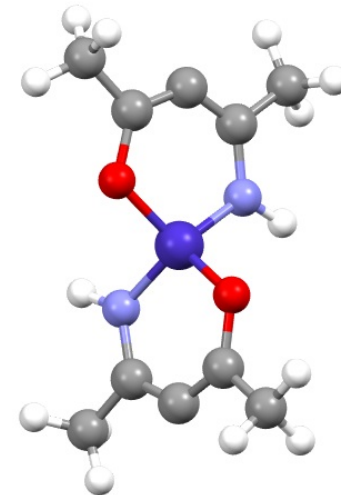

mn36

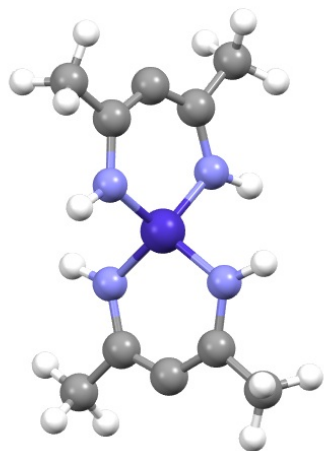

mn37

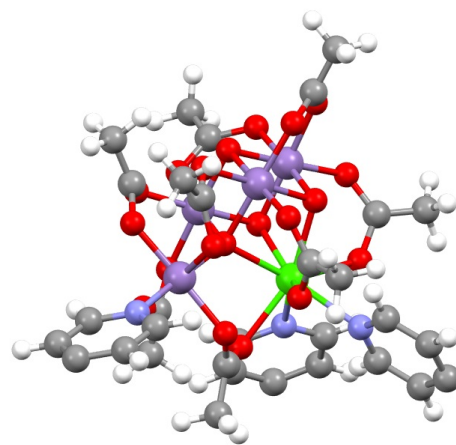

mn38

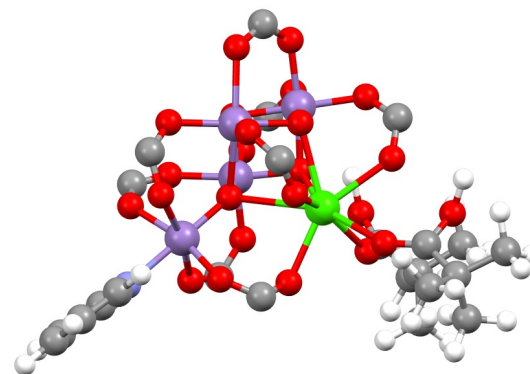

mn39

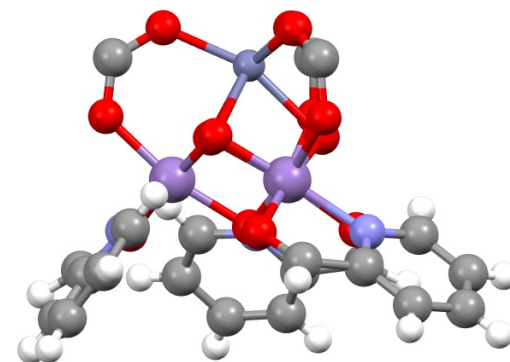

mn40

Supplement: SC-016-D5SC01100K-s001 [file SC-016-D5SC01100K-s001.zip › ESI/si_images/mn33-mn40.pdf]

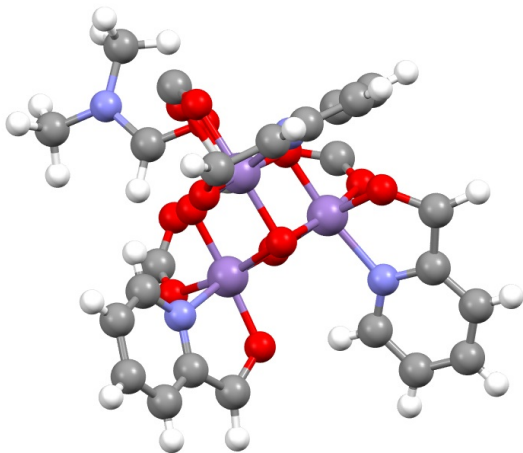

mn41

Supplement: SC-016-D5SC01100K-s001 [file SC-016-D5SC01100K-s001.zip › ESI/si_images/mn41-41.pdf]

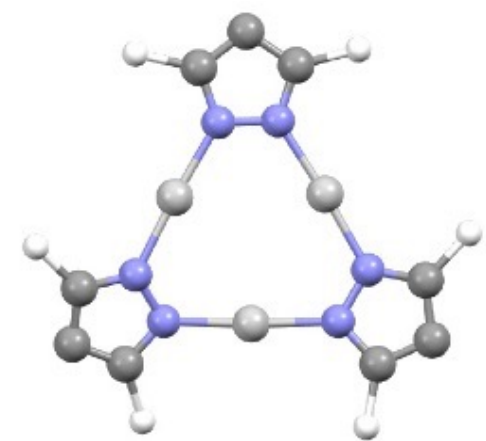

mn9

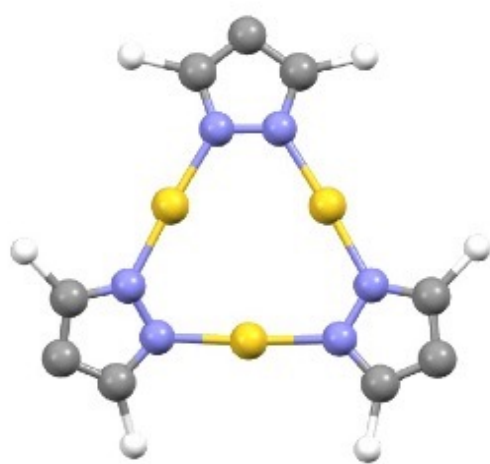

mn10

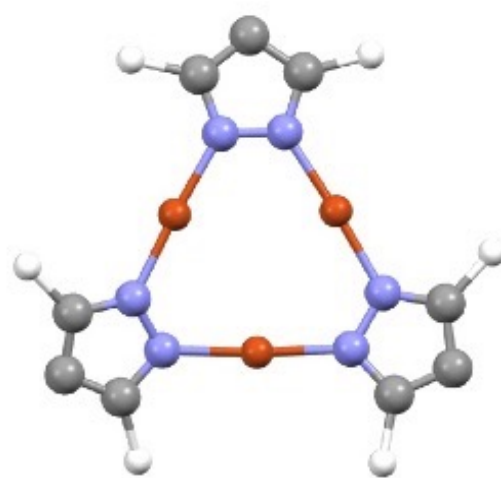

mn11

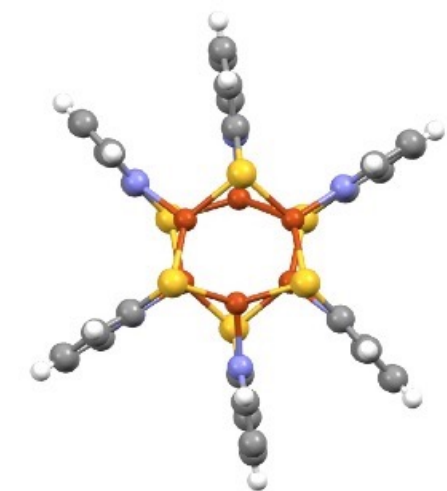

mn13

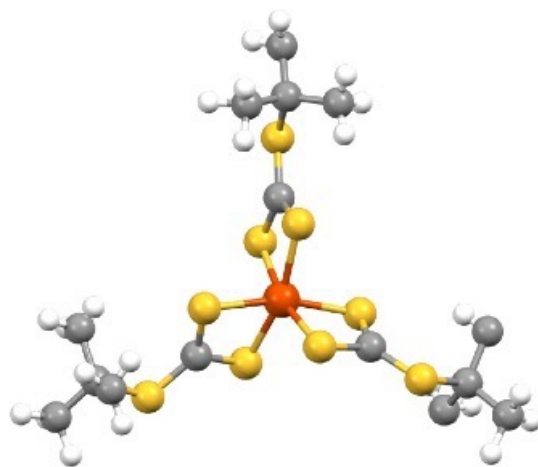

mn14

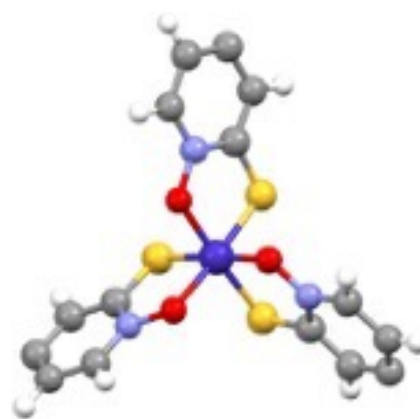

mn15

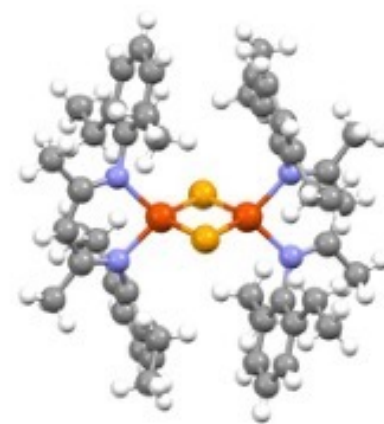

mn16

Supplement: SC-016-D5SC01100K-s001 [file SC-016-D5SC01100K-s001.zip › ESI/si_images/mn9-11_13-16.pdf]

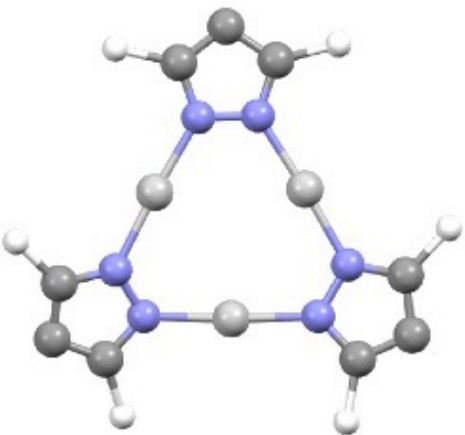

mn9

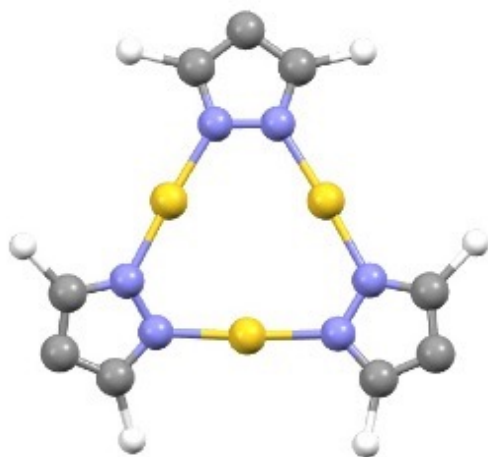

mn10

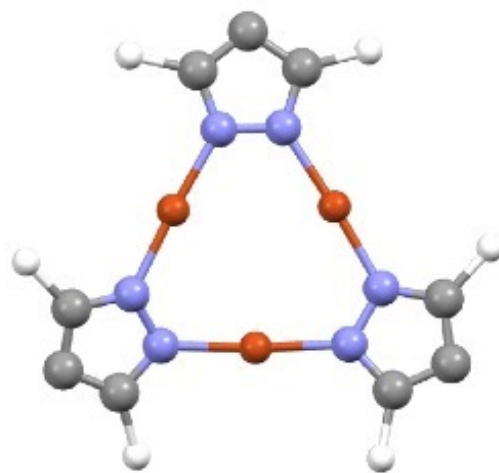

mn11

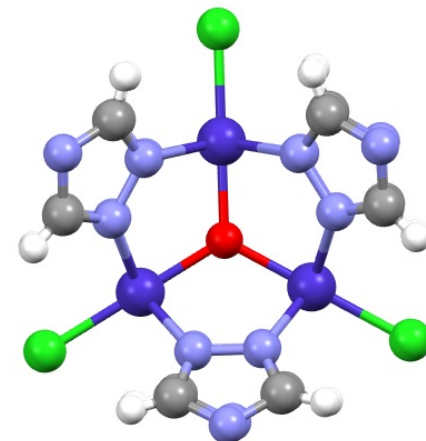

mn12

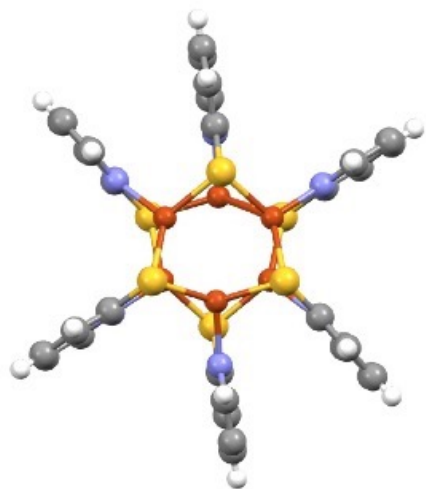

mn13

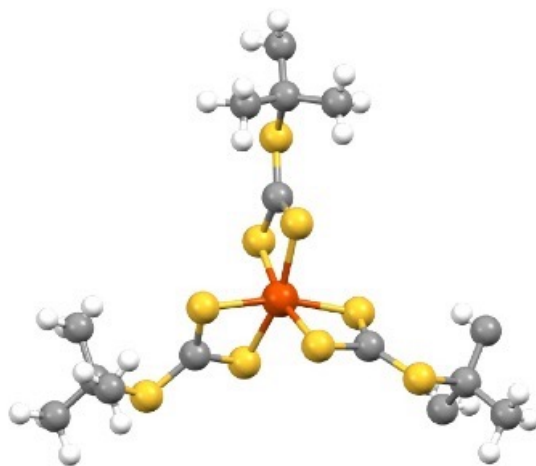

mn14

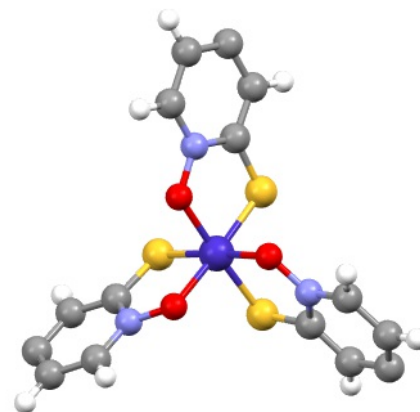

mn15

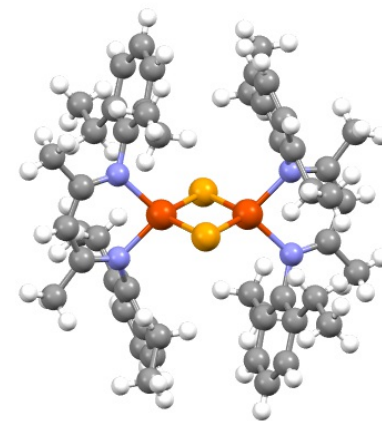

mn16

Supplement: SC-016-D5SC01100K-s001 [file SC-016-D5SC01100K-s001.zip › ESI/si_images/mn9-16.pdf]

pcu-v1-mn13-1-ol12.cif

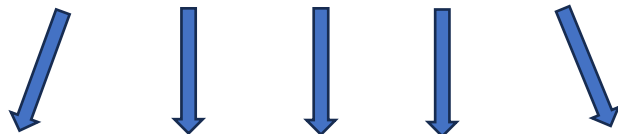

topology   vertex1   metal   edge1   organic  
                         node                           linker

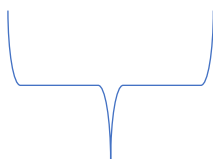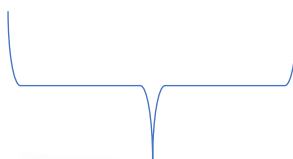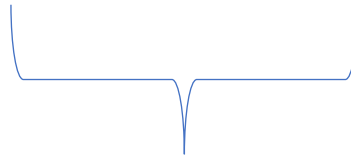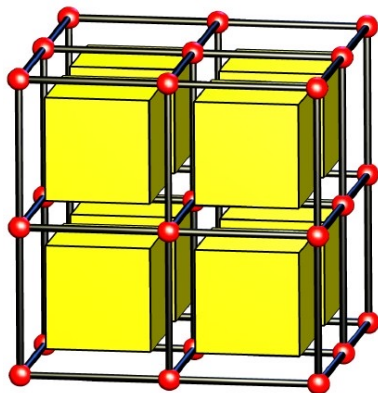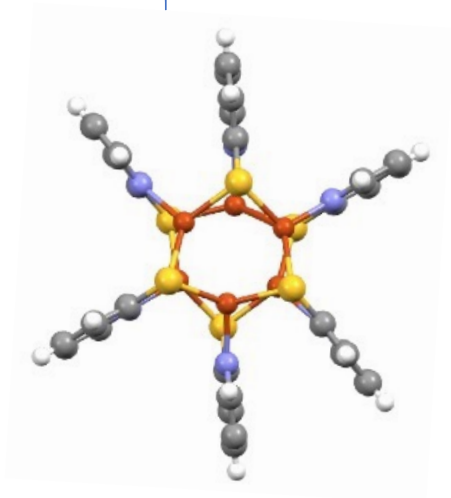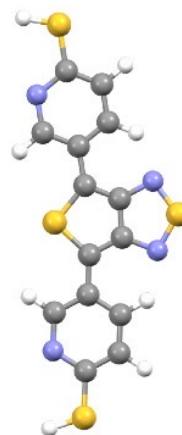

Supplement: SC-016-D5SC01100K-s001 [file SC-016-D5SC01100K-s001.zip › ESI/si_images/mof_naming_final.pdf]

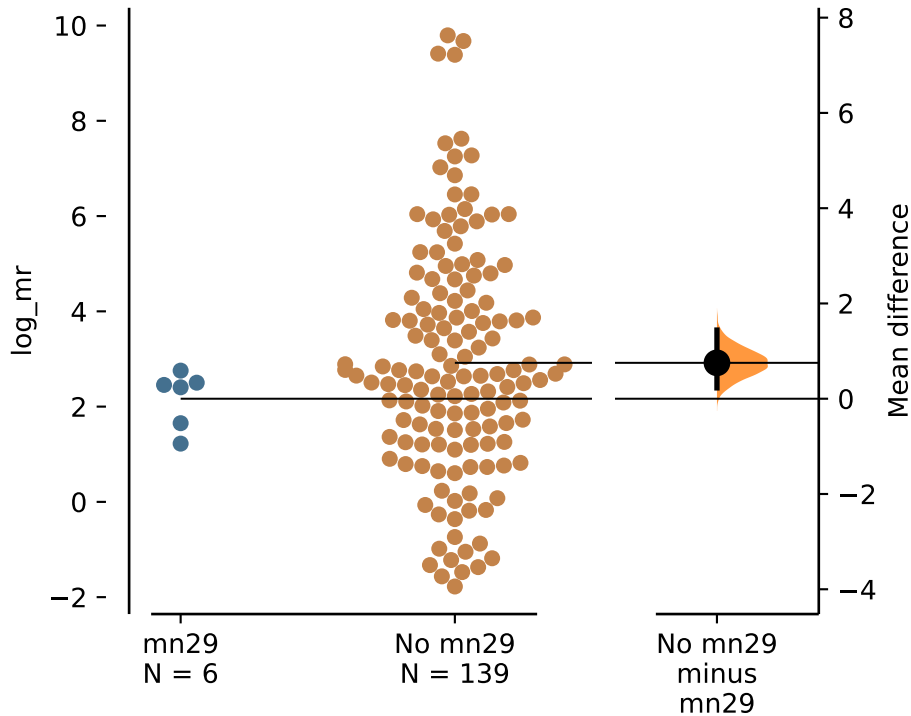

Supplement: SC-016-D5SC01100K-s001 [file SC-016-D5SC01100K-s001.zip › ESI/si_images/mr_mn29.pdf]

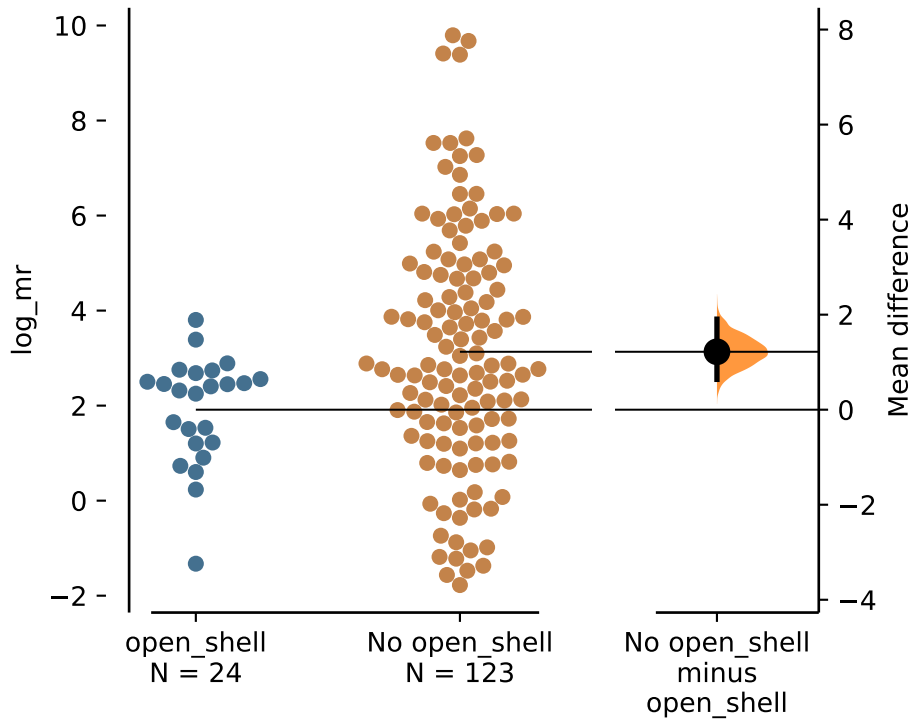

Supplement: SC-016-D5SC01100K-s001 [file SC-016-D5SC01100K-s001.zip › ESI/si_images/mr_open_shell.pdf]

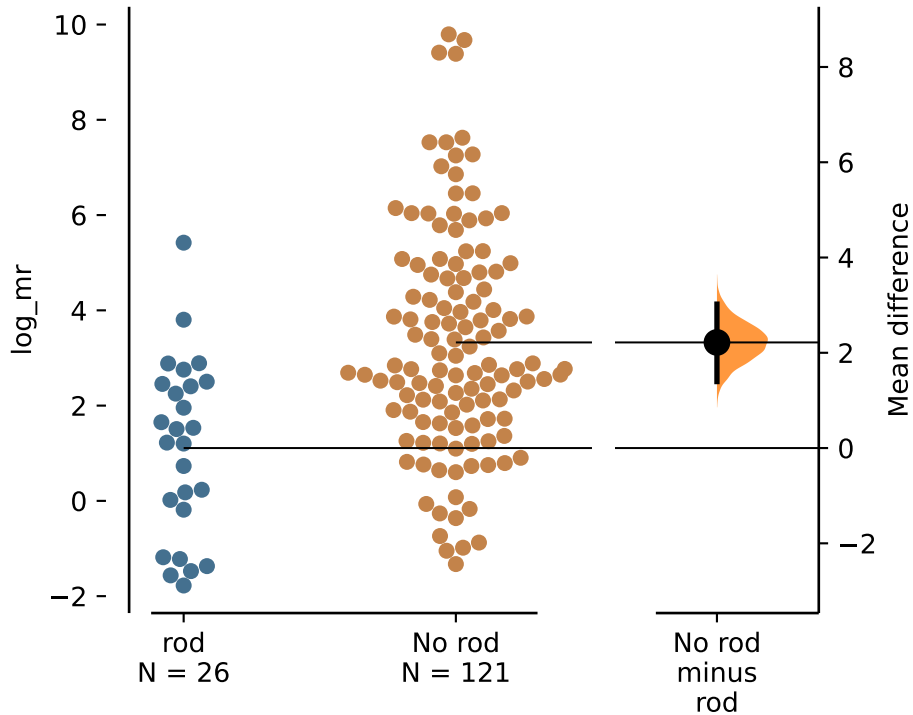

Supplement: SC-016-D5SC01100K-s001 [file SC-016-D5SC01100K-s001.zip › ESI/si_images/mr_rod.pdf]

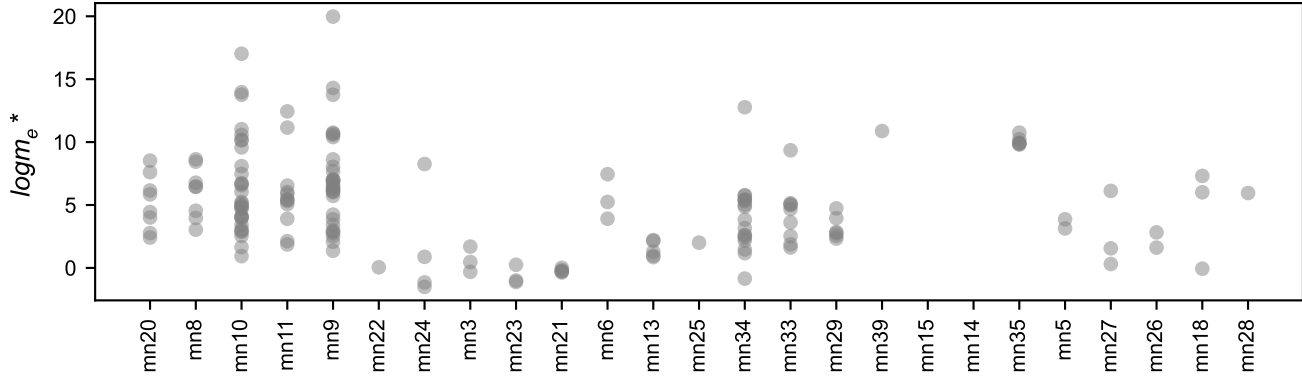

Supplement: SC-016-D5SC01100K-s001 [file SC-016-D5SC01100K-s001.zip › ESI/si_images/nodes_def_me.pdf]

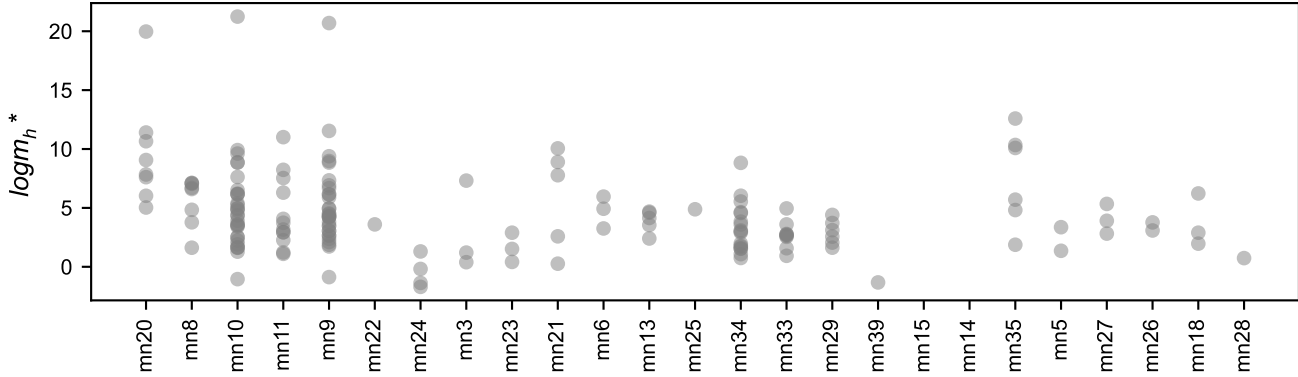

Supplement: SC-016-D5SC01100K-s001 [file SC-016-D5SC01100K-s001.zip › ESI/si_images/nodes_def_mh.pdf]

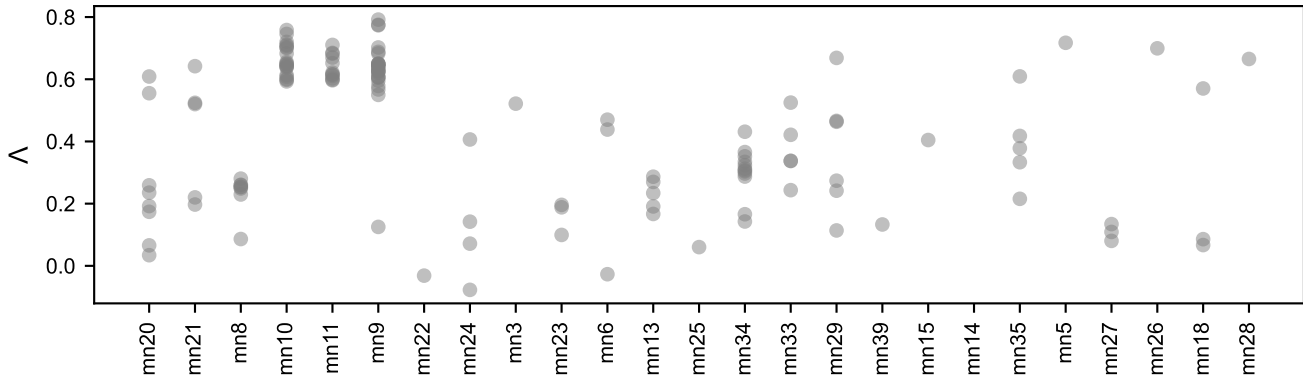

Supplement: SC-016-D5SC01100K-s001 [file SC-016-D5SC01100K-s001.zip › ESI/si_images/nodes_def_ovlp.pdf]

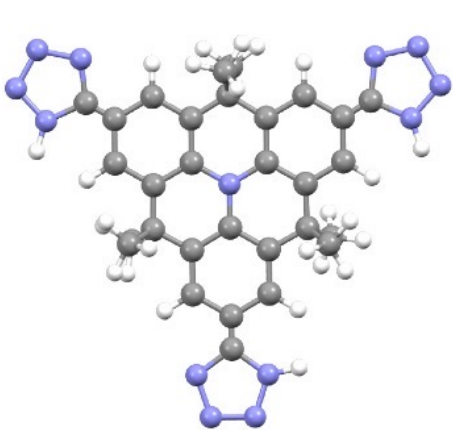

ol1

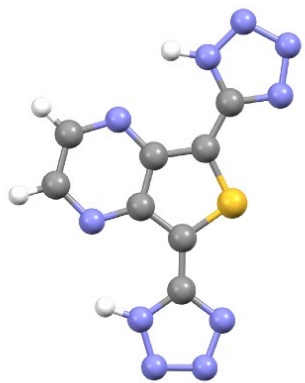

ol2

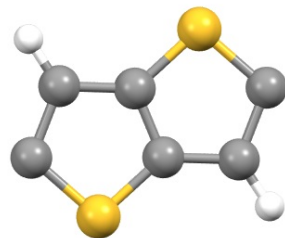

ol3

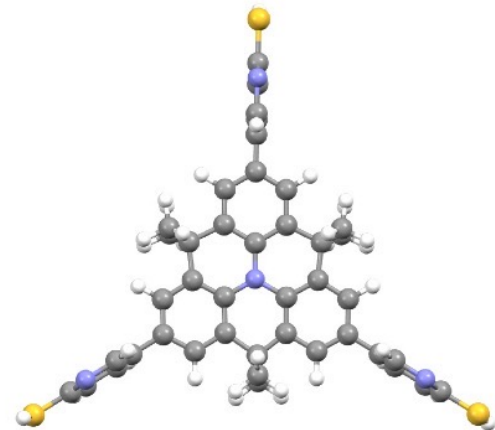

ol4

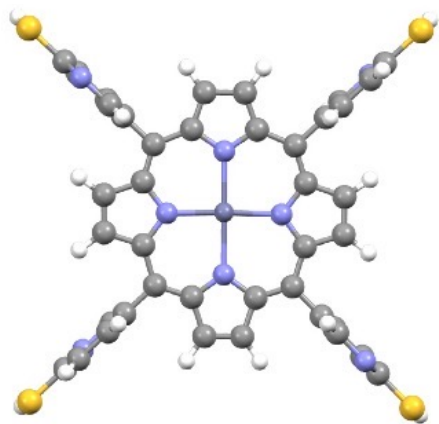

ol5

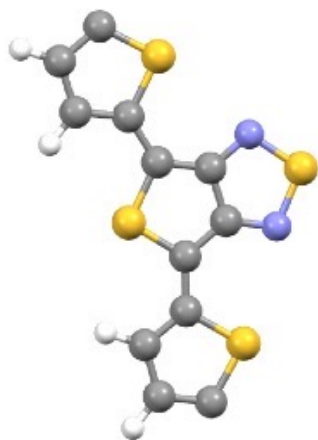

ol6

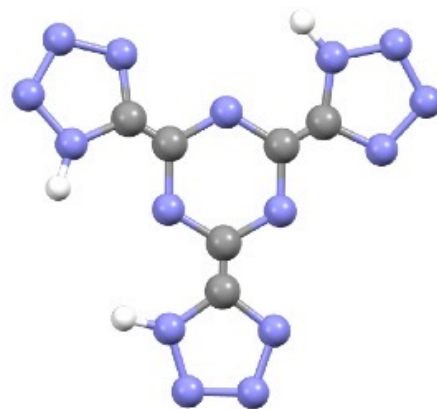

ol7

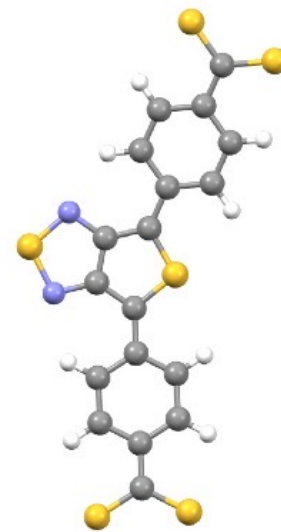

ol8

Supplement: SC-016-D5SC01100K-s001 [file SC-016-D5SC01100K-s001.zip › ESI/si_images/ol1-8.pdf]

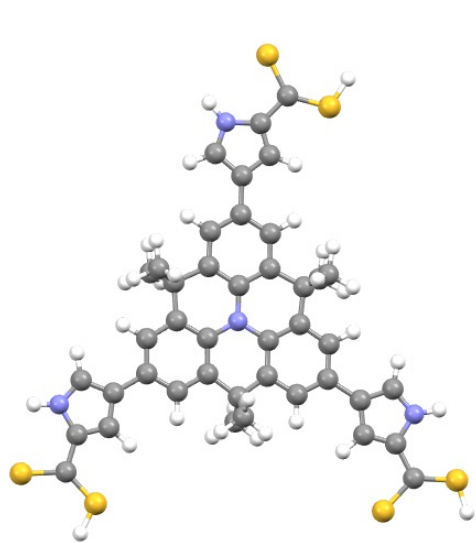

ol17

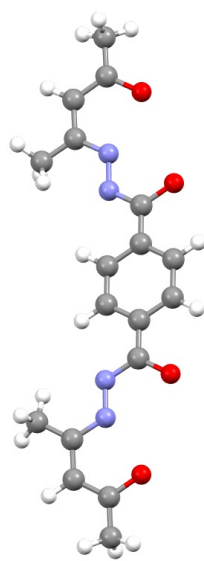

ol18

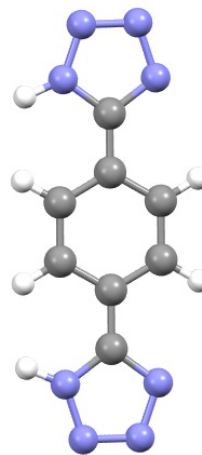

ol19

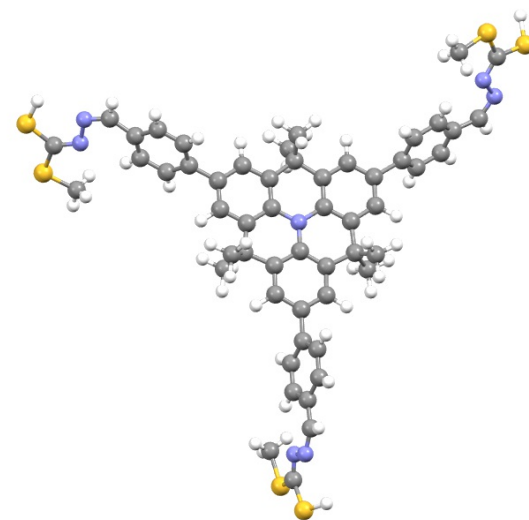

ol20

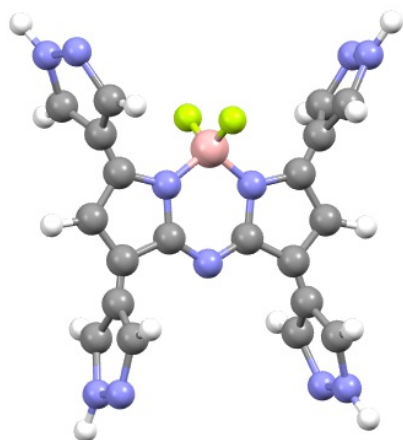

ol21

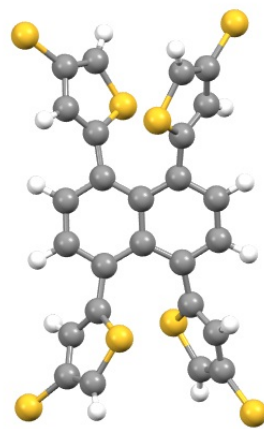

ol22

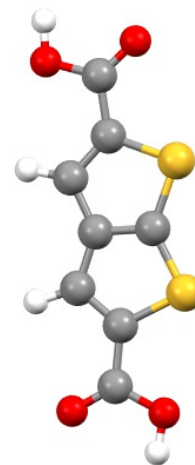

ol23

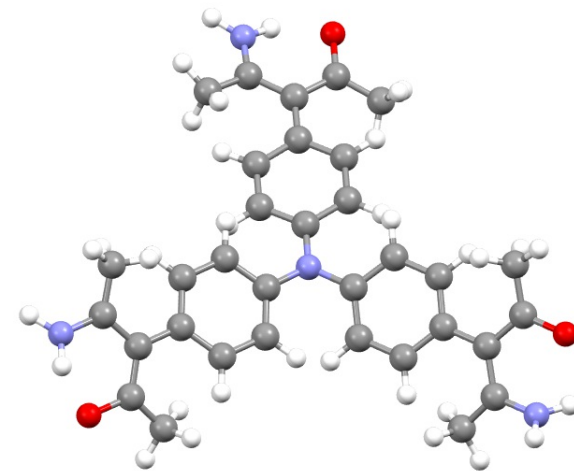

ol24

Supplement: SC-016-D5SC01100K-s001 [file SC-016-D5SC01100K-s001.zip › ESI/si_images/ol17-24.pdf]

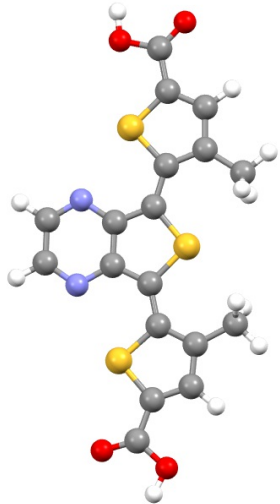

ol25

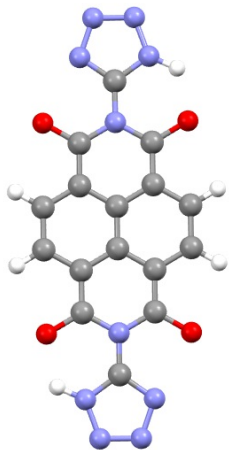

ol26

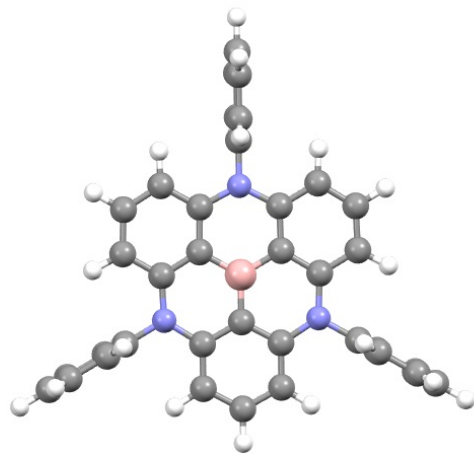

ol27

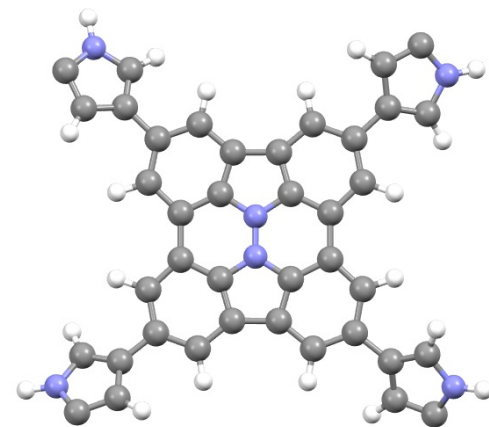

ol28

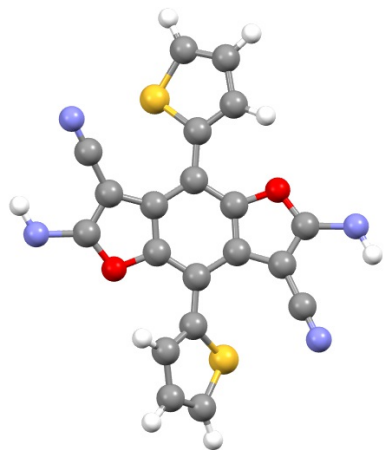

ol29

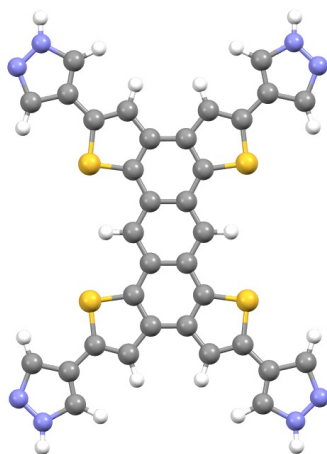

ol30

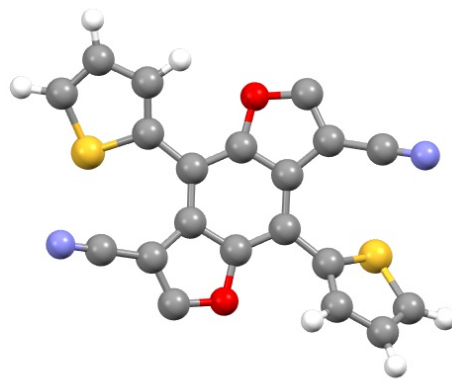

ol31

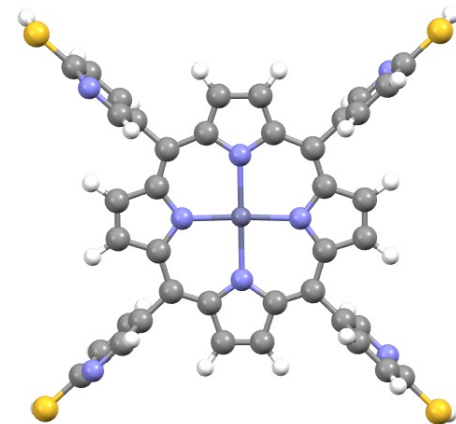

ol32

Supplement: SC-016-D5SC01100K-s001 [file SC-016-D5SC01100K-s001.zip › ESI/si_images/ol25-32.pdf]

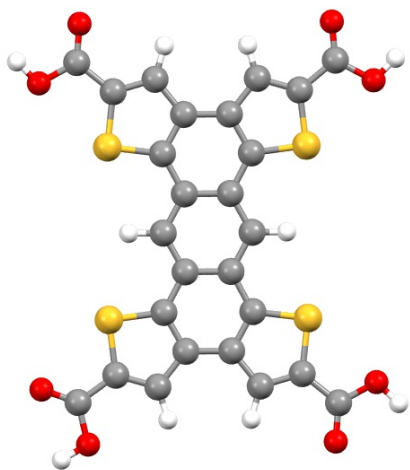

ol33

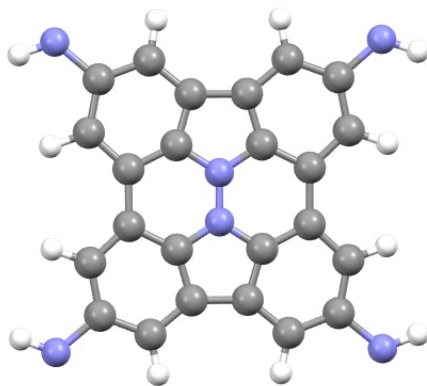

ol34

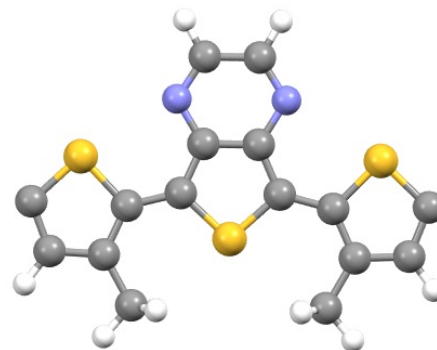

ol35

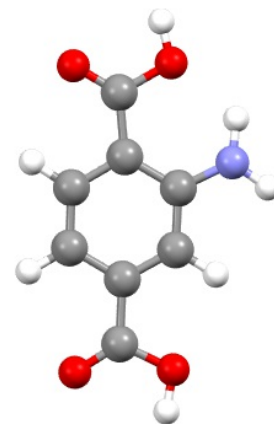

ol36

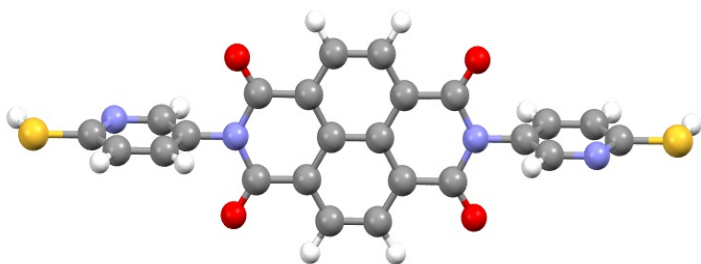

ol37

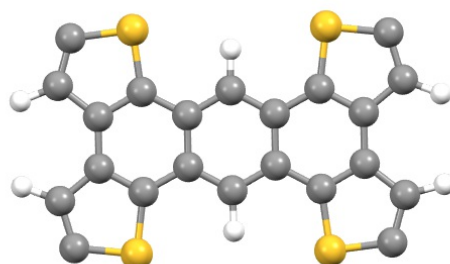

ol38

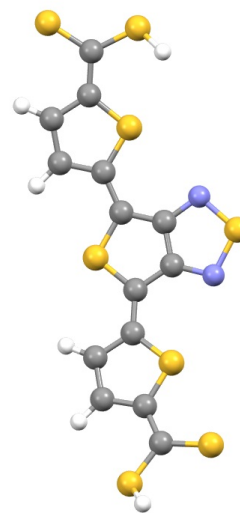

ol39

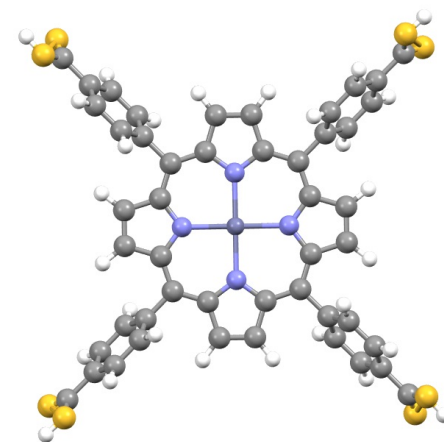

ol40

Supplement: SC-016-D5SC01100K-s001 [file SC-016-D5SC01100K-s001.zip › ESI/si_images/ol33-40.pdf]

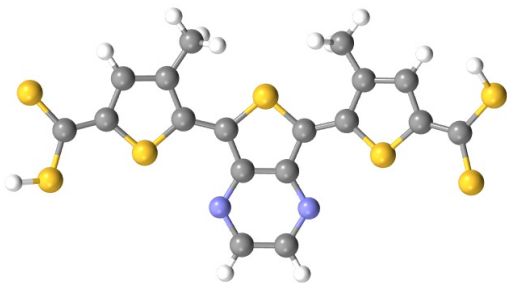

ol41

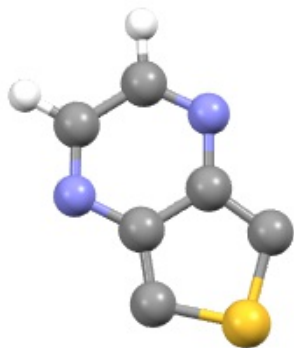

ol42

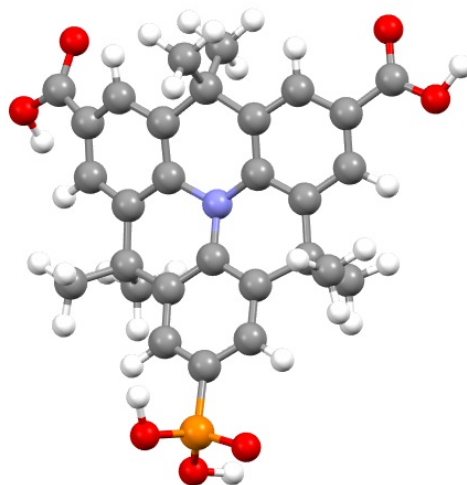

ol43

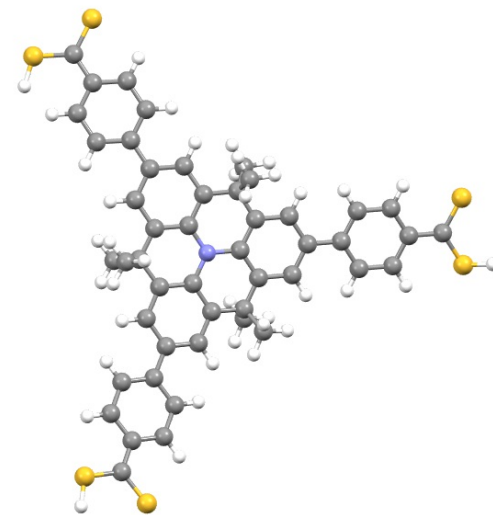

ol44

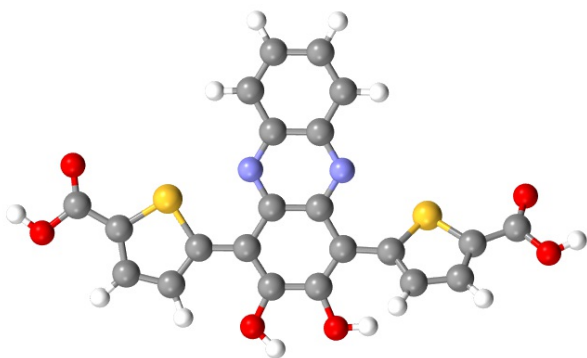

ol45

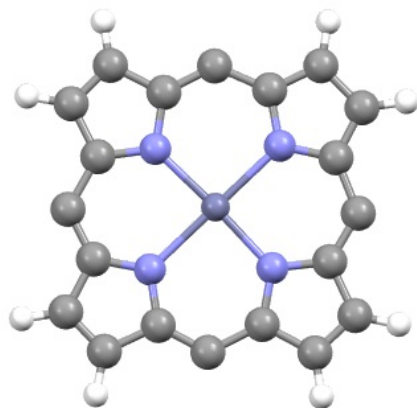

ol46

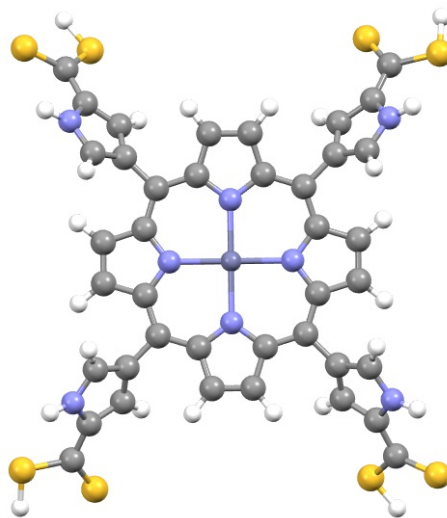

ol47

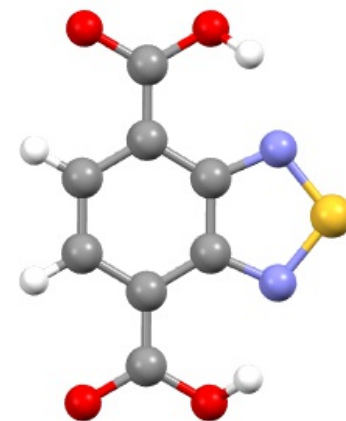

ol48

Supplement: SC-016-D5SC01100K-s001 [file SC-016-D5SC01100K-s001.zip › ESI/si_images/ol41-48.pdf]

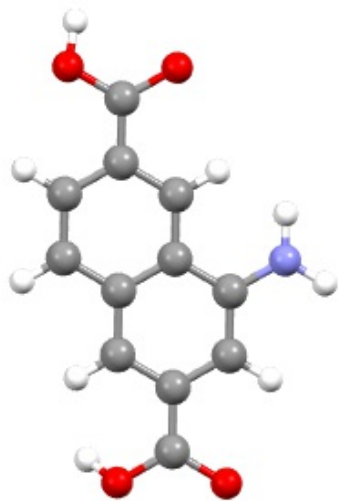

ol49

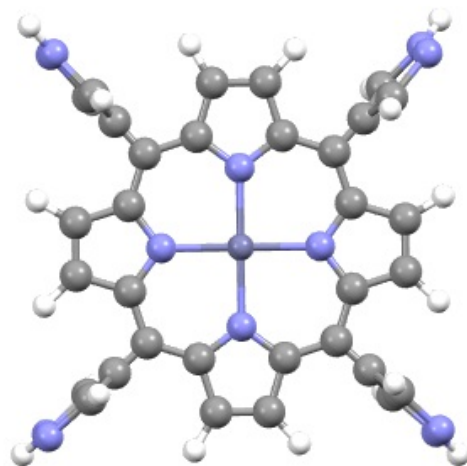

ol50

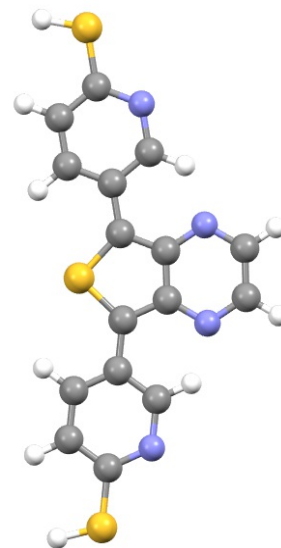

ol51

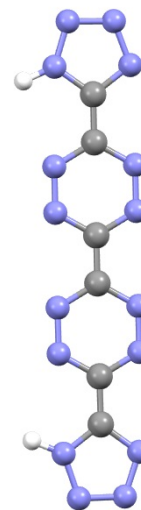

ol52

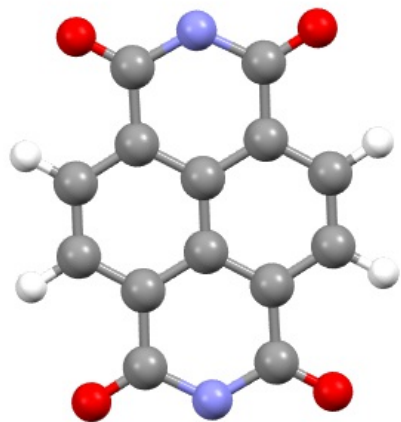

ol53

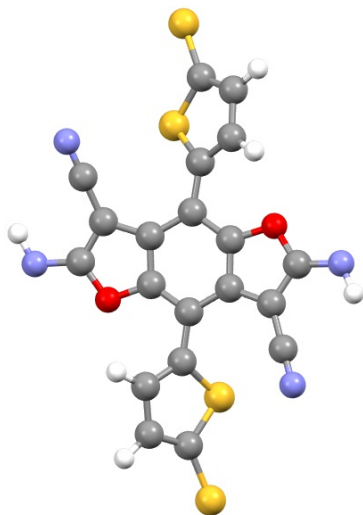

ol54

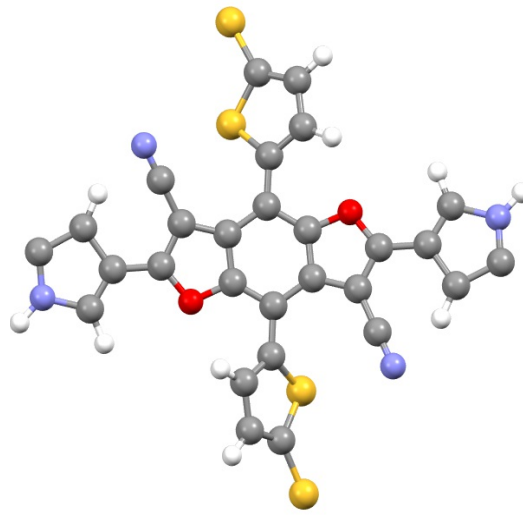

ol55

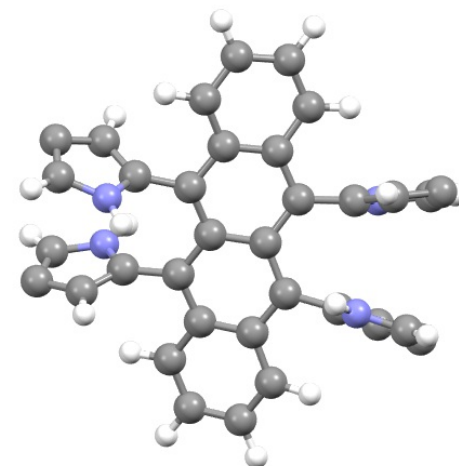

ol56

Supplement: SC-016-D5SC01100K-s001 [file SC-016-D5SC01100K-s001.zip › ESI/si_images/ol49-56.pdf]

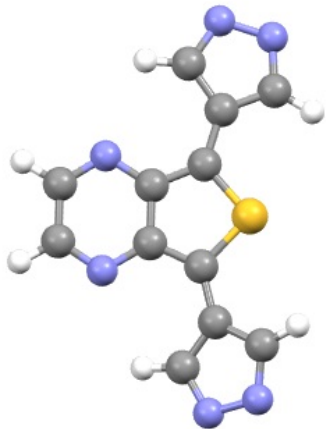

ol57

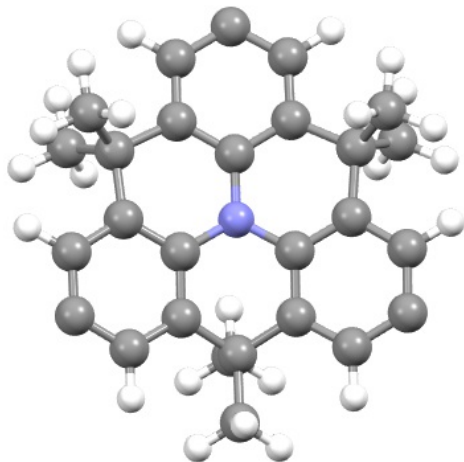

ol58

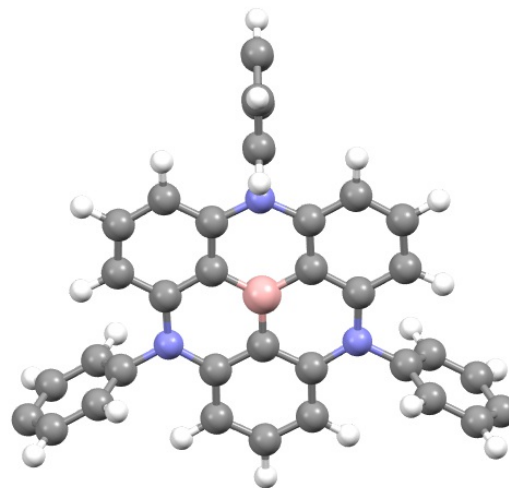

ol59

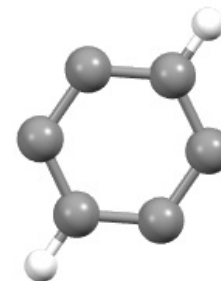

ol60

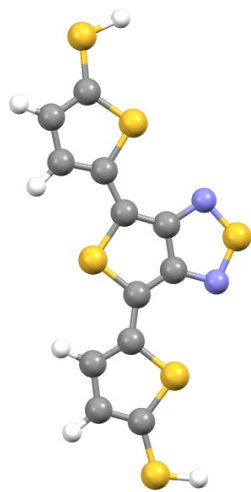

ol61

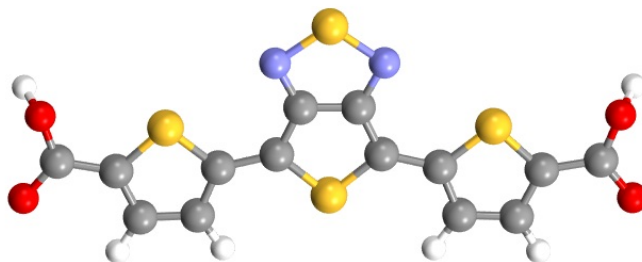

ol62

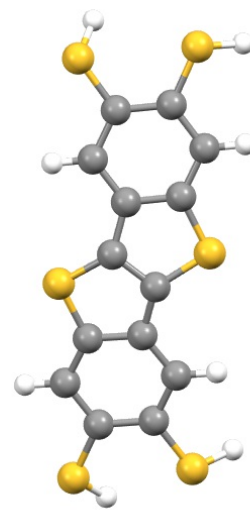

ol63

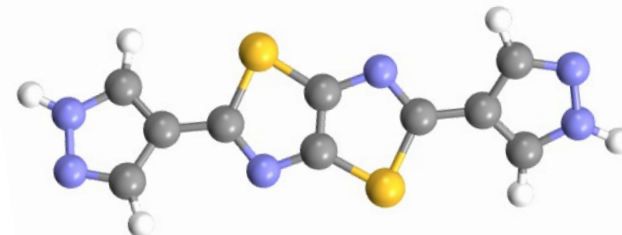

ol64

Supplement: SC-016-D5SC01100K-s001 [file SC-016-D5SC01100K-s001.zip › ESI/si_images/ol57-64.pdf]

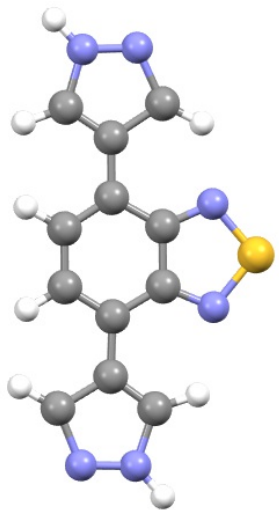

ol65

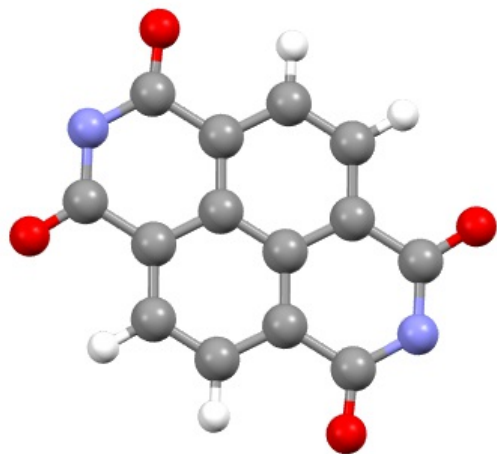

ol66

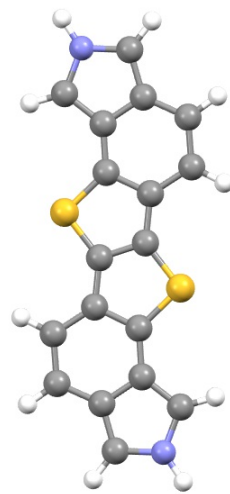

ol67

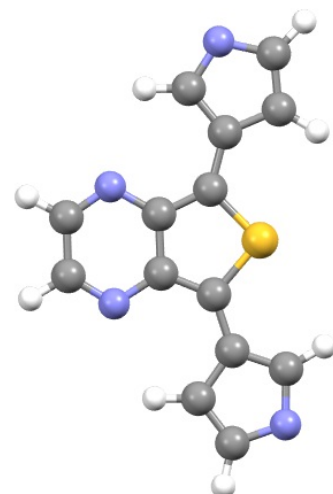

ol68

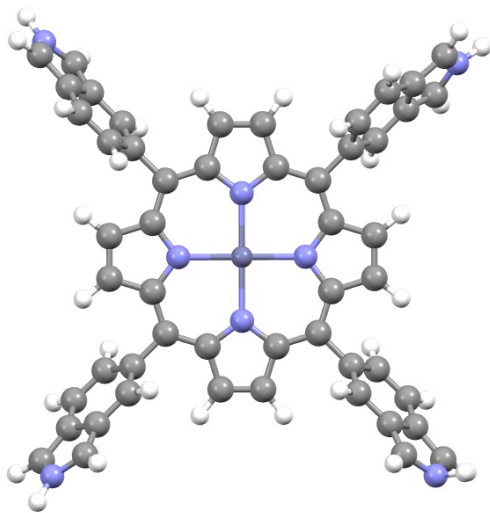

ol69

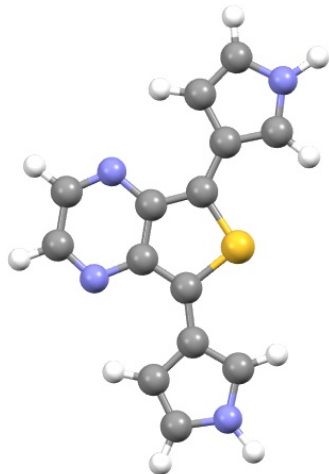

ol70

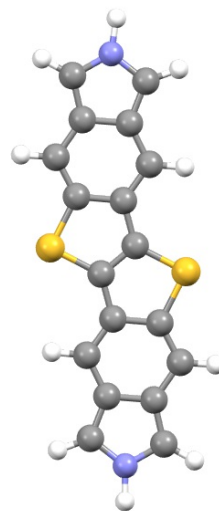

ol71

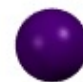

ol72

Supplement: SC-016-D5SC01100K-s001 [file SC-016-D5SC01100K-s001.zip › ESI/si_images/ol65-72.pdf]

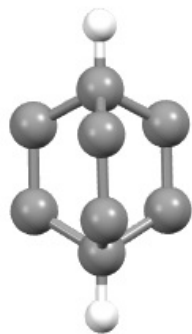

ol73

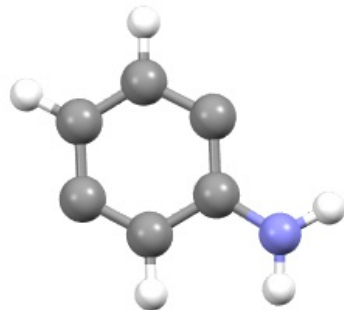

ol79

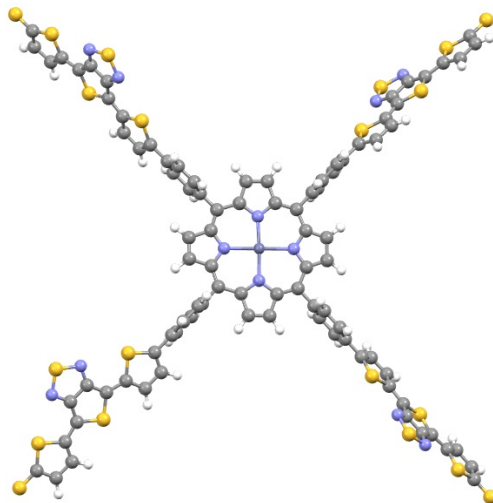

ol81

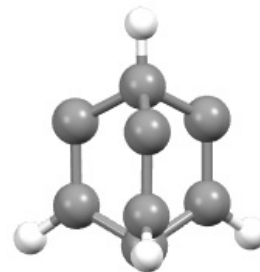

ol82

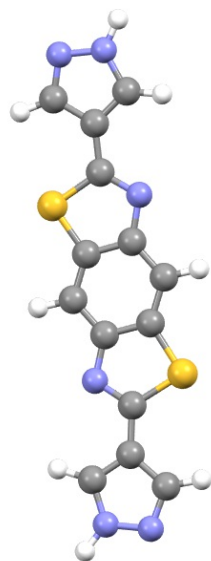

ol83

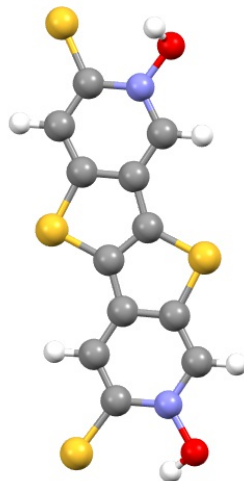

ol84

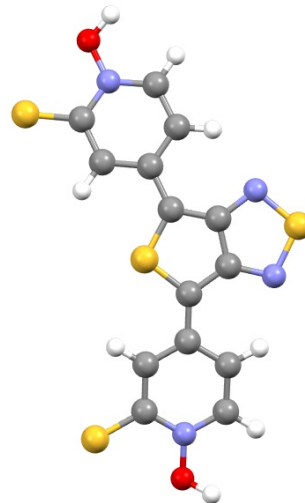

ol85

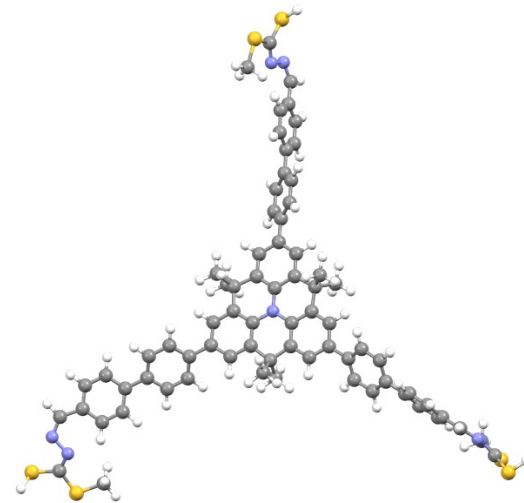

ol86

Supplement: SC-016-D5SC01100K-s001 [file SC-016-D5SC01100K-s001.zip › ESI/si_images/ol73-86.pdf]

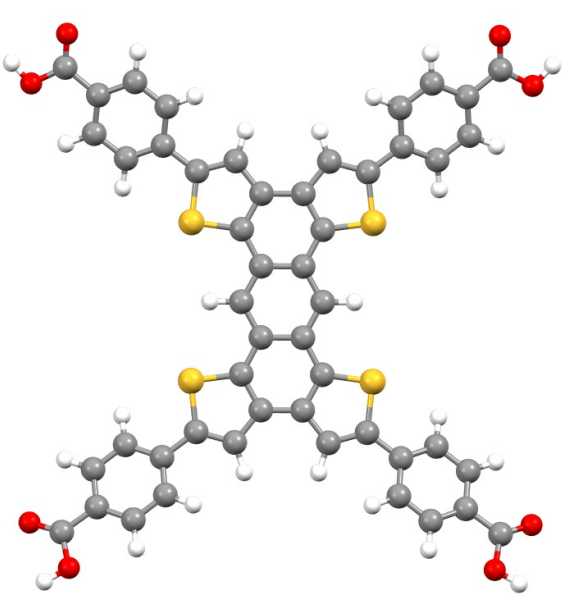

ol87

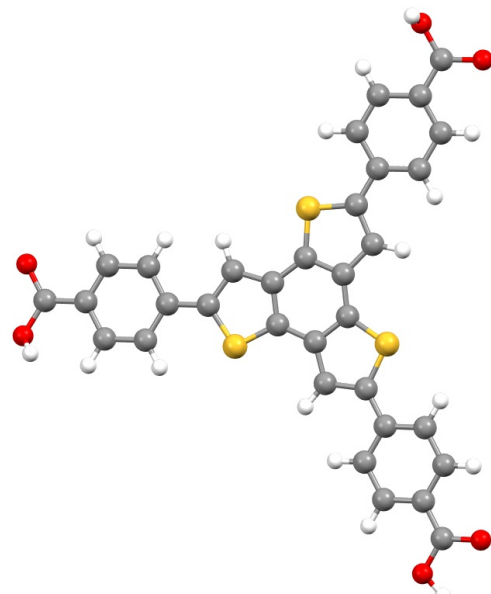

ol88

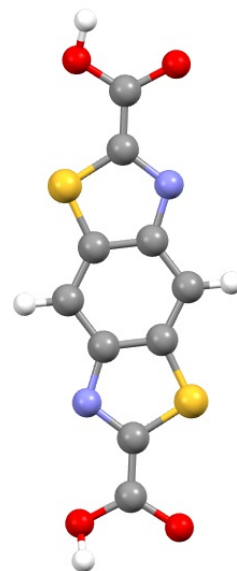

ol89

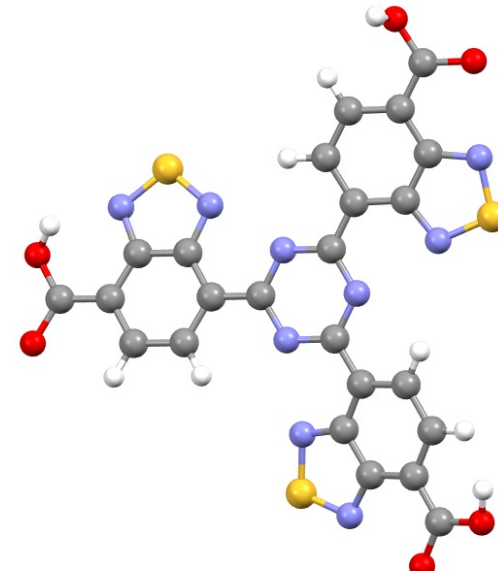

ol90

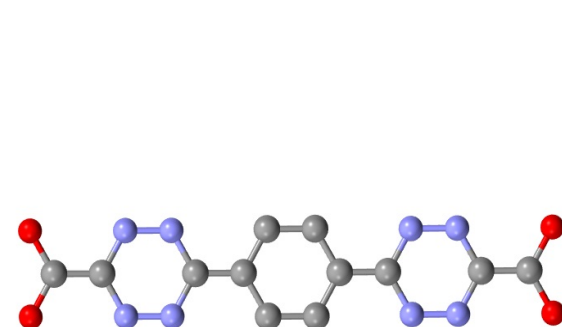

ol91

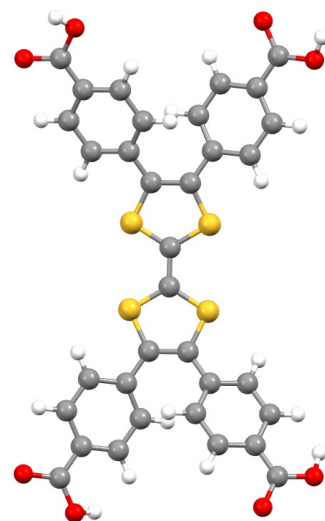

ol92

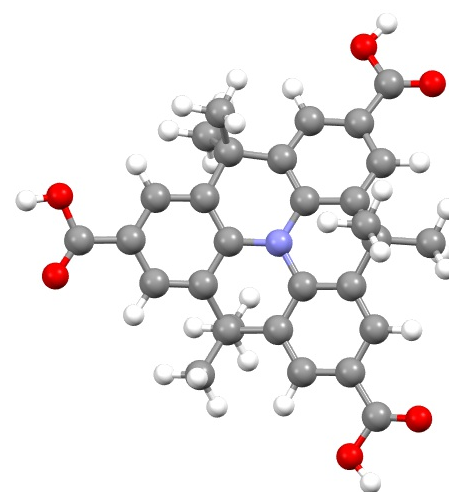

ol93

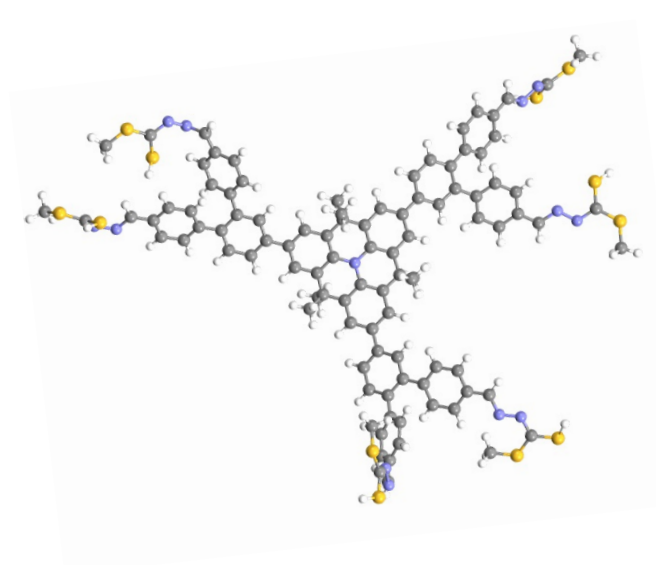

ol94

Supplement: SC-016-D5SC01100K-s001 [file SC-016-D5SC01100K-s001.zip › ESI/si_images/ol87-ol94.pdf]

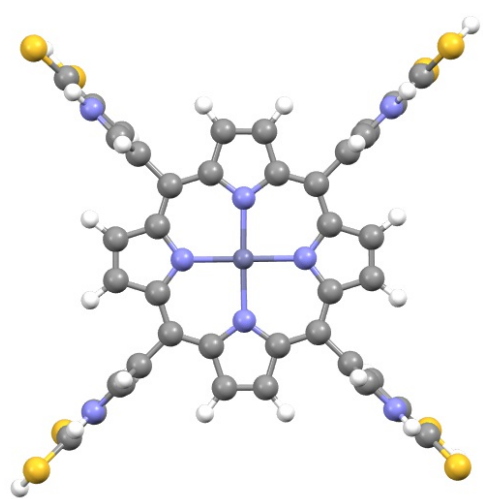

ol9

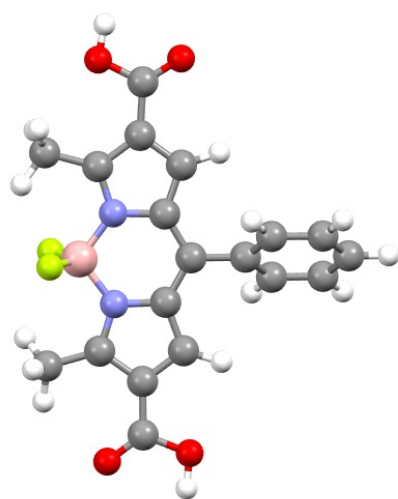

ol10

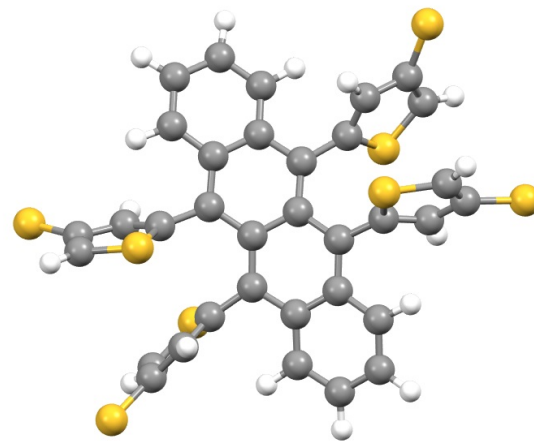

ol11

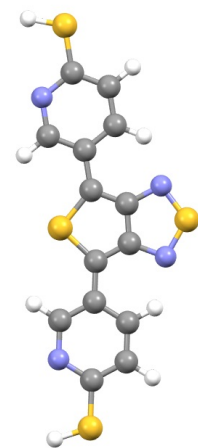

ol12

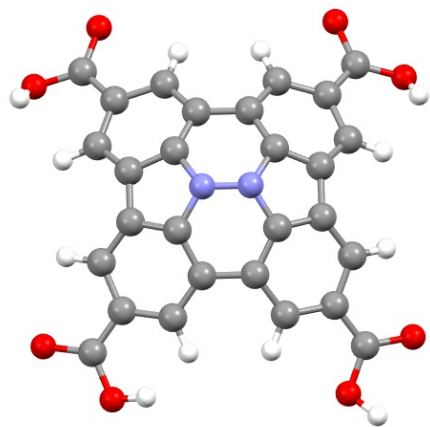

ol13

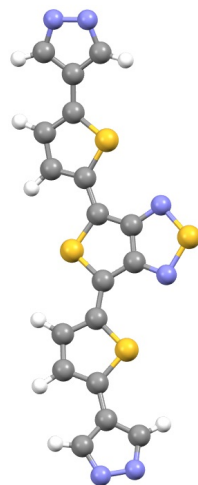

ol14

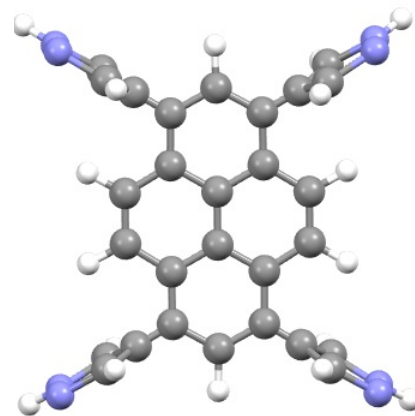

ol15

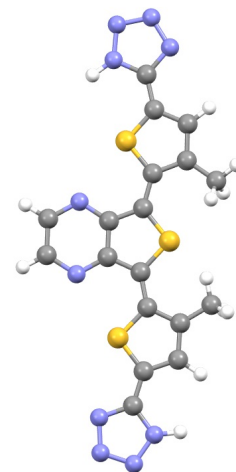

ol16

Supplement: SC-016-D5SC01100K-s001 [file SC-016-D5SC01100K-s001.zip › ESI/si_images/ol9-16.pdf]

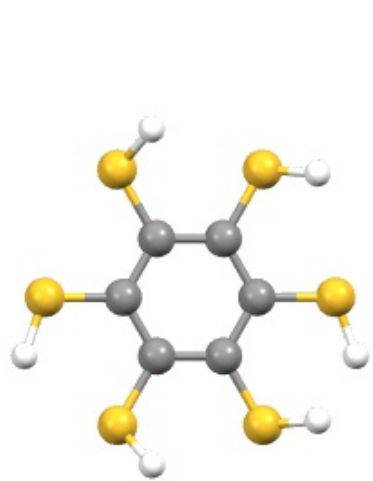

ol95

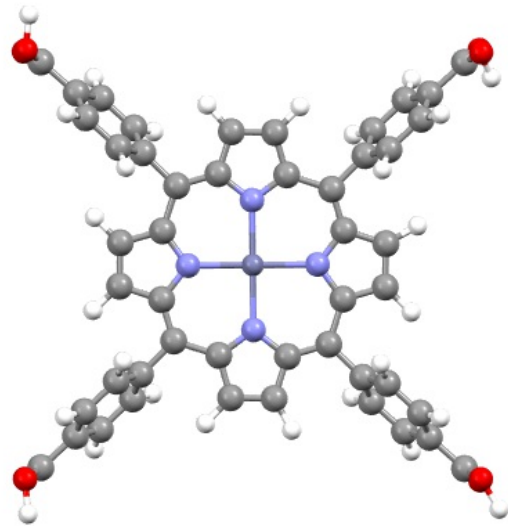

ol96

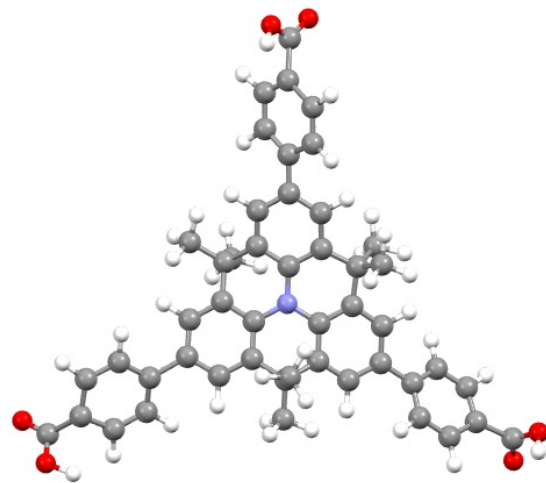

ol97

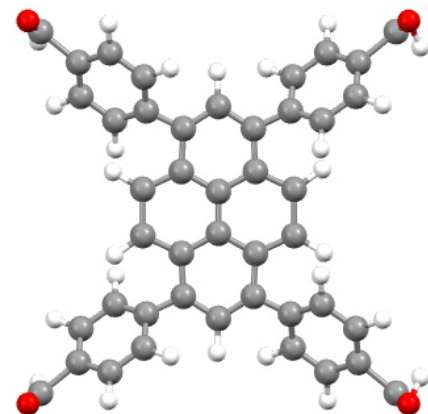

ol98

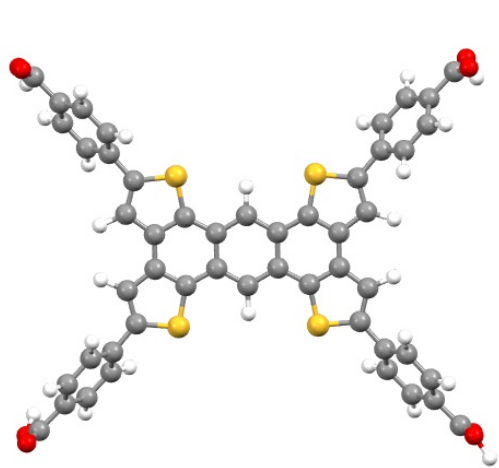

ol99

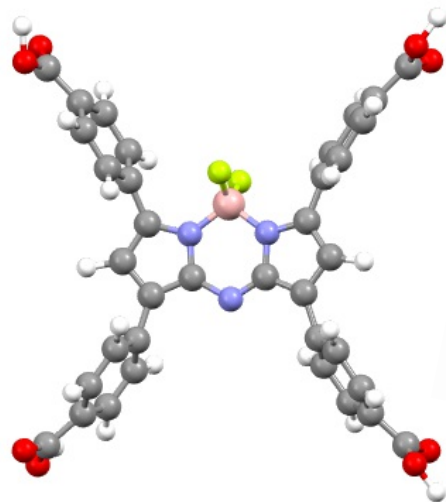

ol100

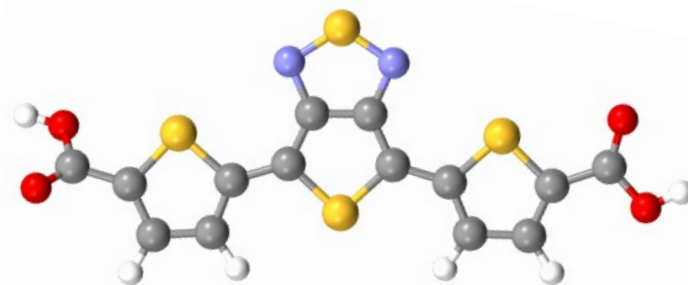

ol101

Supplement: SC-016-D5SC01100K-s001 [file SC-016-D5SC01100K-s001.zip › ESI/si_images/ol95-101.pdf]

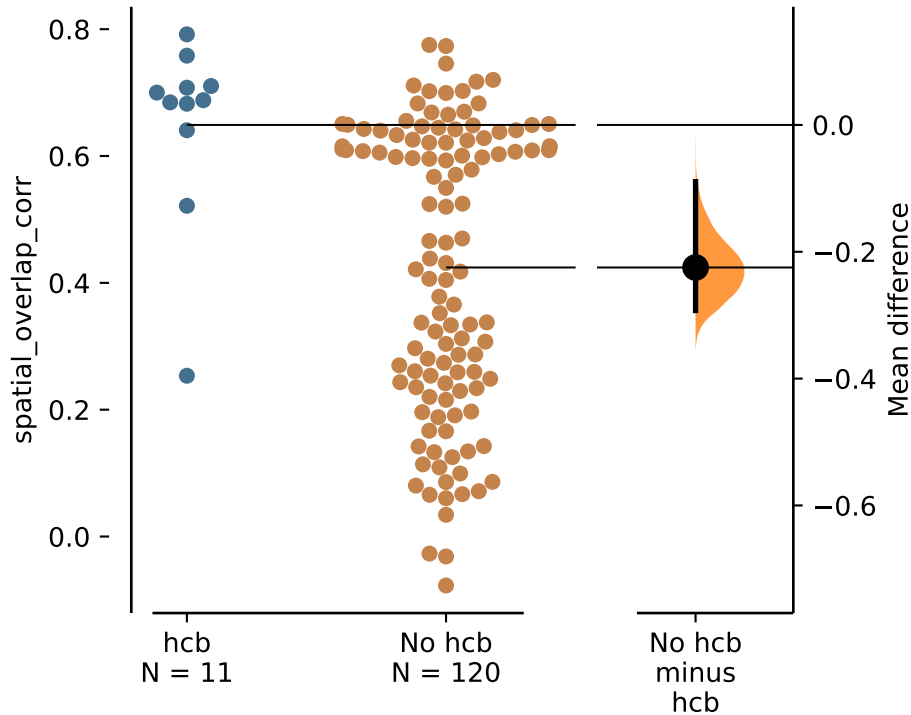

Supplement: SC-016-D5SC01100K-s001 [file SC-016-D5SC01100K-s001.zip › ESI/si_images/ovlp_hcb.pdf]

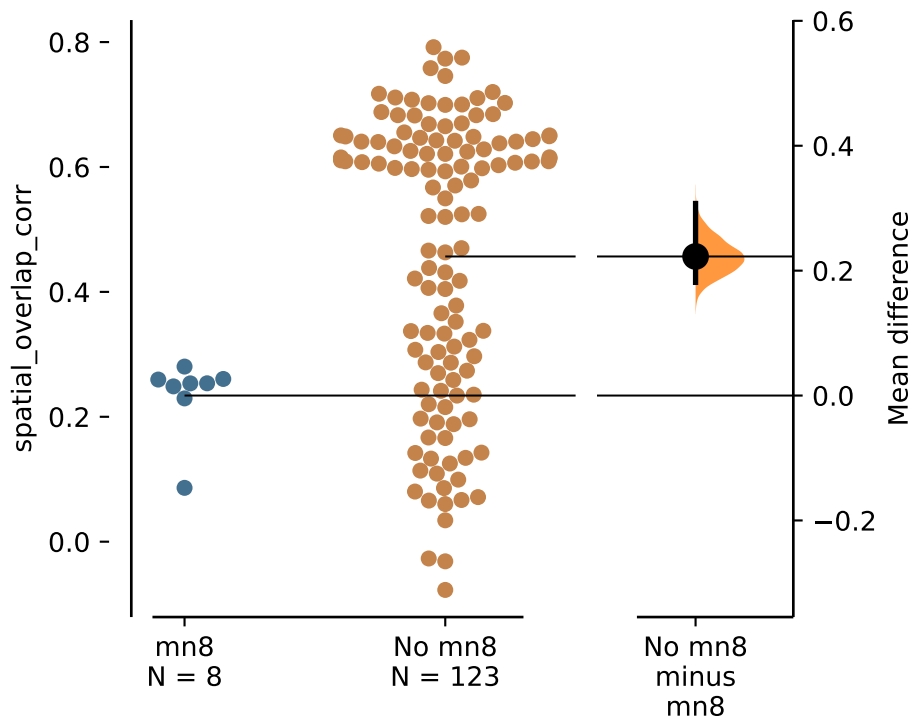

Supplement: SC-016-D5SC01100K-s001 [file SC-016-D5SC01100K-s001.zip › ESI/si_images/ovlp_mn8.pdf]

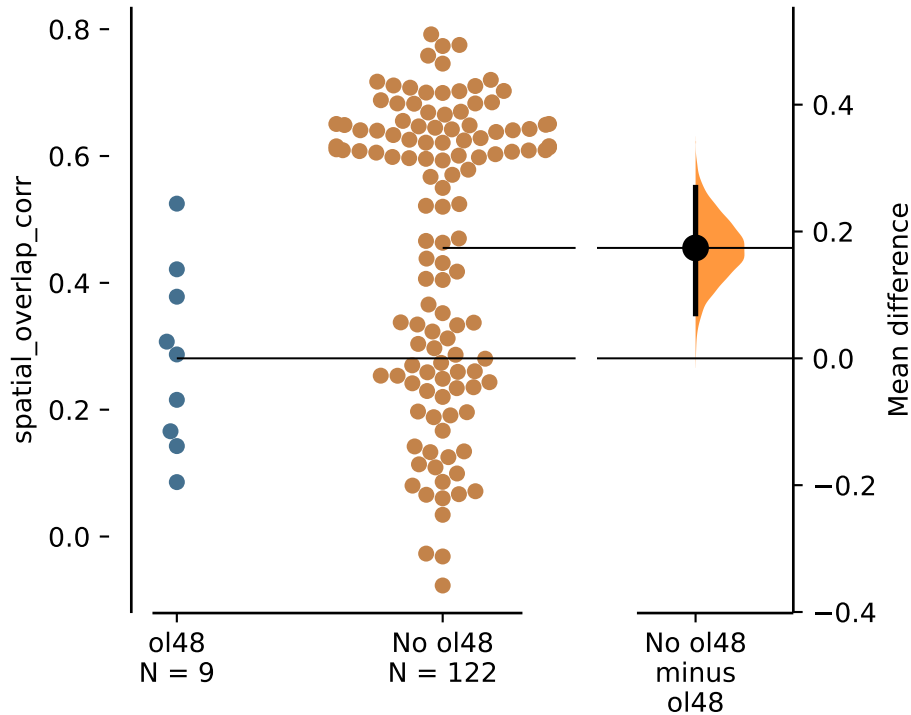

Supplement: SC-016-D5SC01100K-s001 [file SC-016-D5SC01100K-s001.zip › ESI/si_images/ovlp_ol48.pdf]

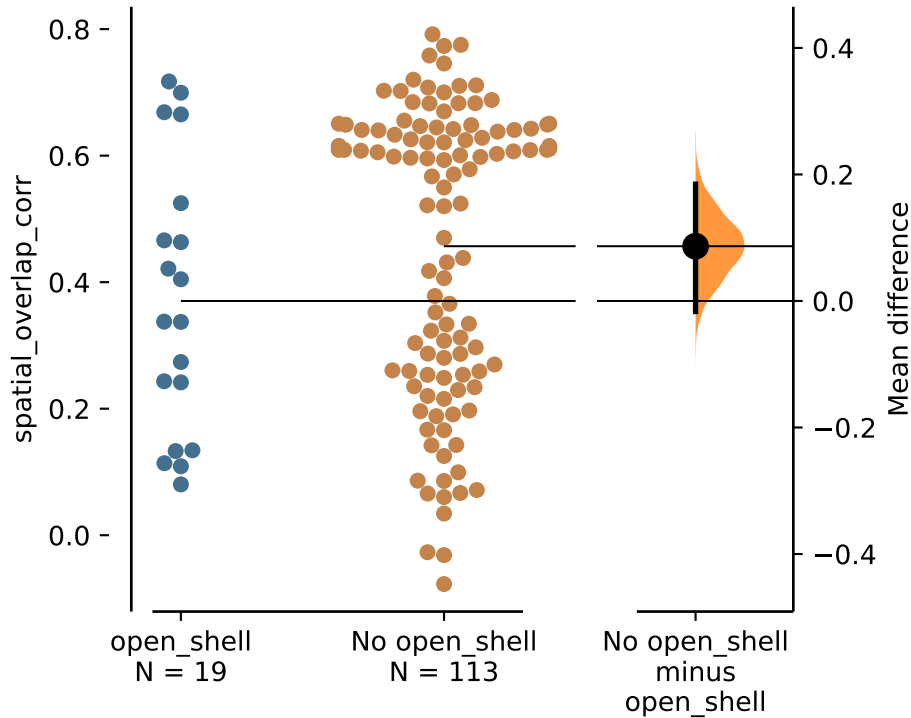

Supplement: SC-016-D5SC01100K-s001 [file SC-016-D5SC01100K-s001.zip › ESI/si_images/ovlp_open_shell.pdf]

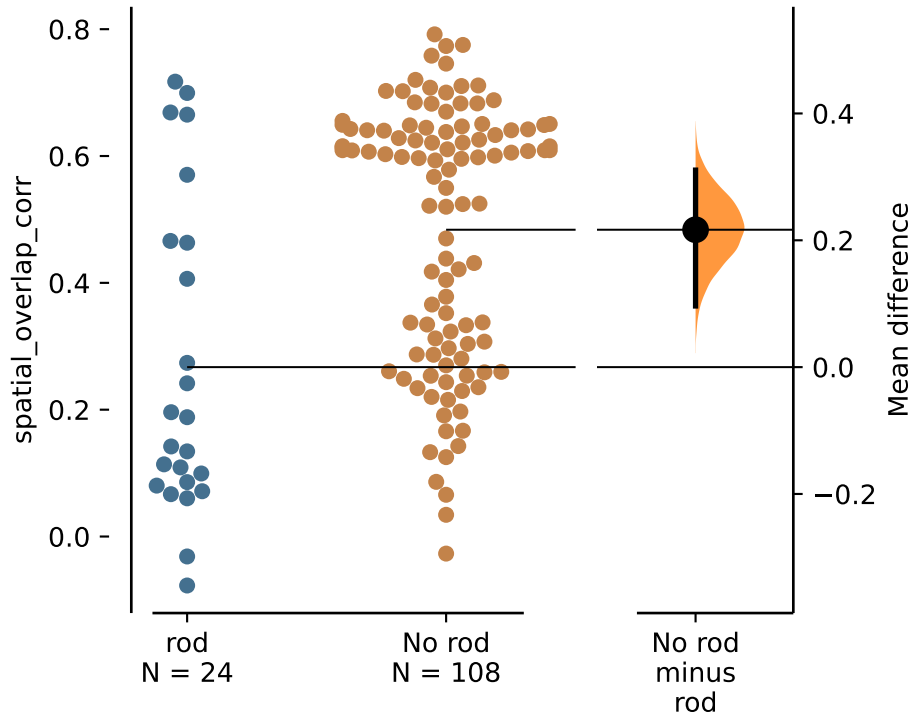

Supplement: SC-016-D5SC01100K-s001 [file SC-016-D5SC01100K-s001.zip › ESI/si_images/ovlp_rod.pdf]

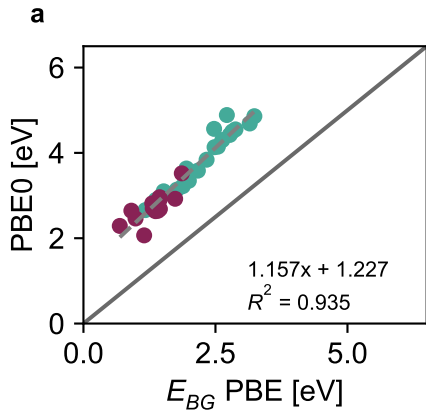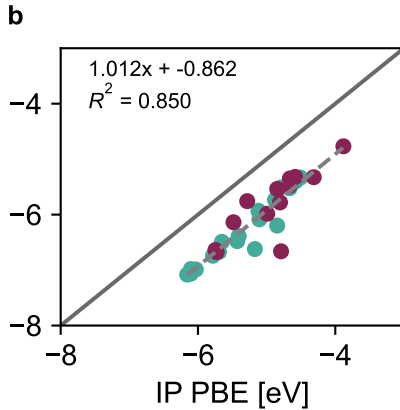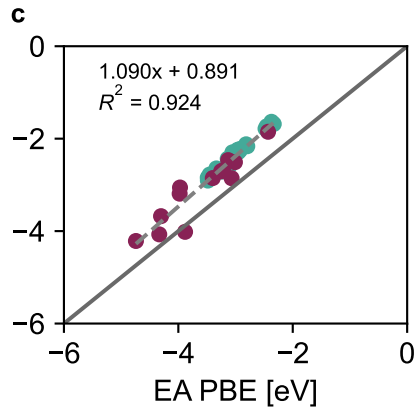

Supplement: SC-016-D5SC01100K-s001 [file SC-016-D5SC01100K-s001.zip › ESI/si_images/pbe-pbe0.pdf]

GPT-J and MOFT predictions on CDP and QMOF

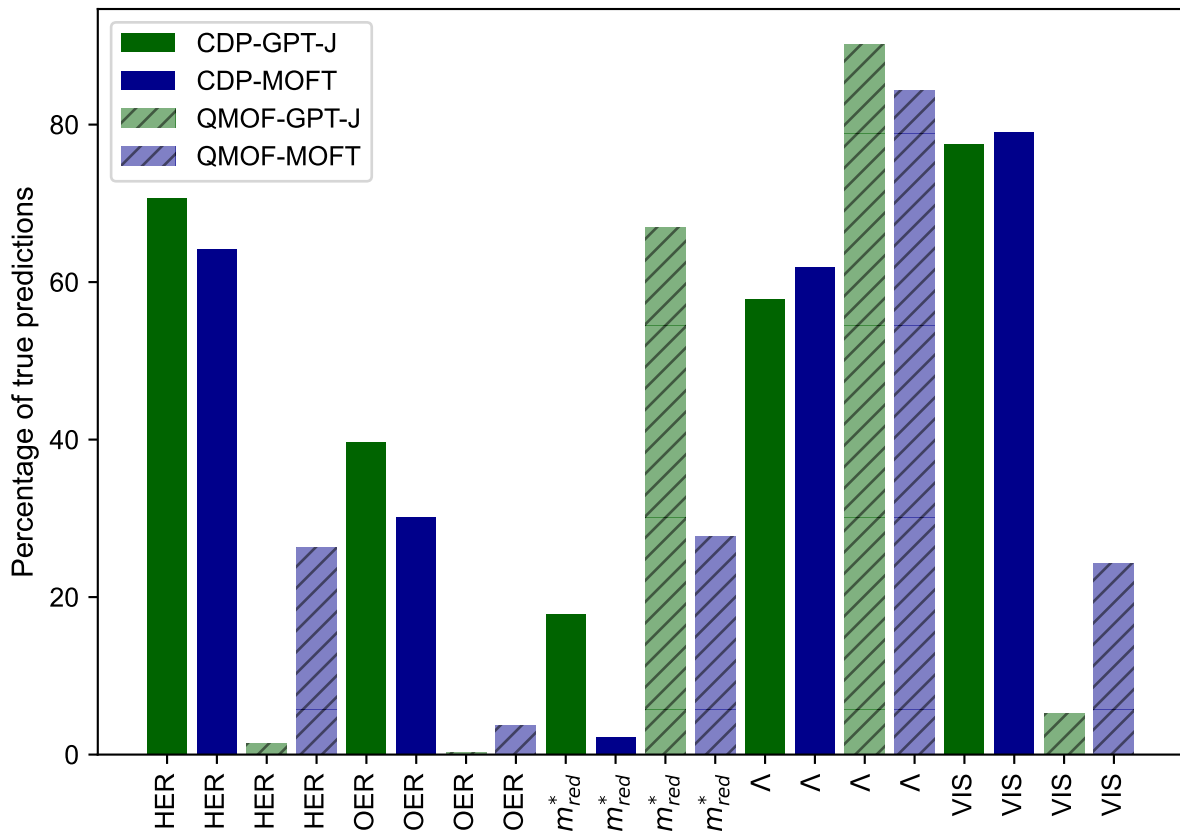

Supplement: SC-016-D5SC01100K-s001 [file SC-016-D5SC01100K-s001.zip › ESI/si_images/preds.pdf]

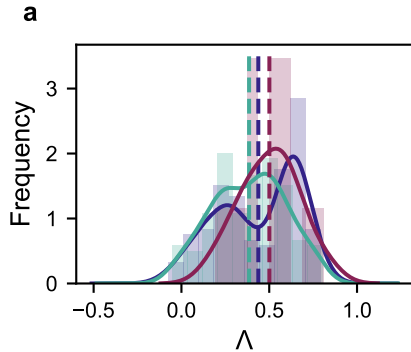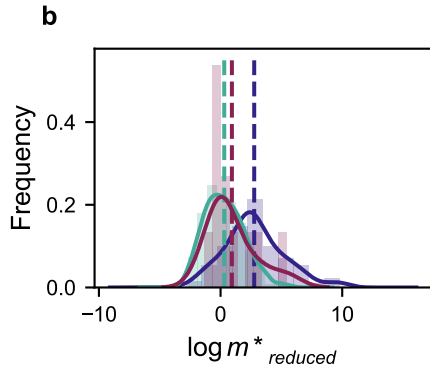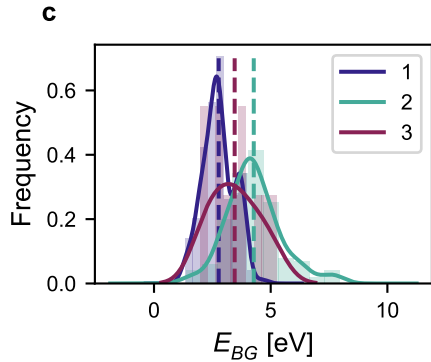

Supplement: SC-016-D5SC01100K-s001 [file SC-016-D5SC01100K-s001.zip › ESI/si_images/refs_compare.pdf]

**a**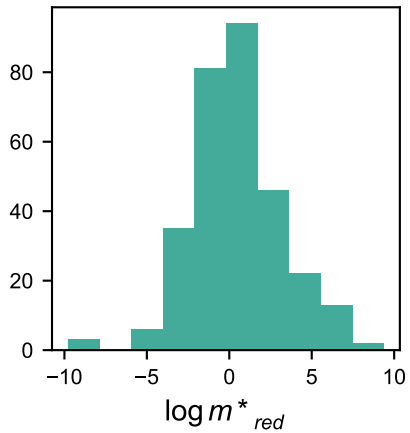**b**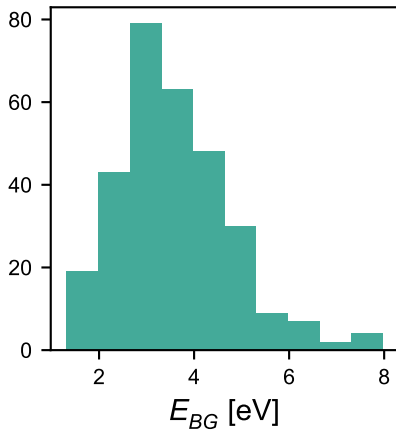**c**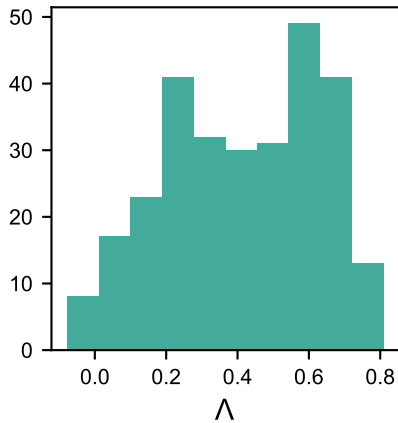

Supplement: SC-016-D5SC01100K-s001 [file SC-016-D5SC01100K-s001.zip › ESI/si_images/stats_314.pdf]

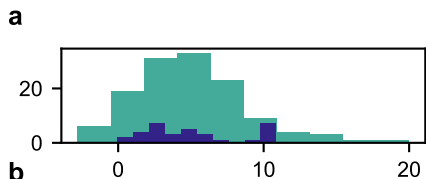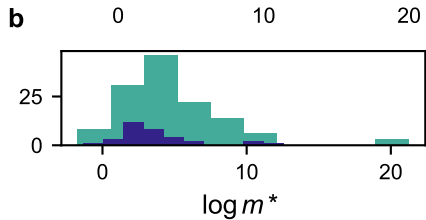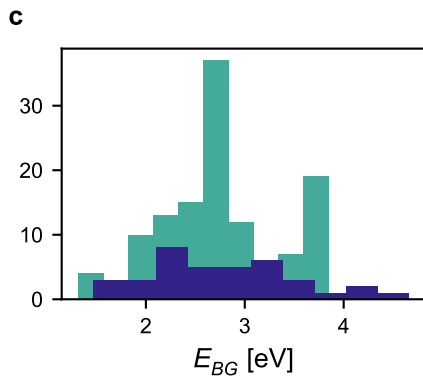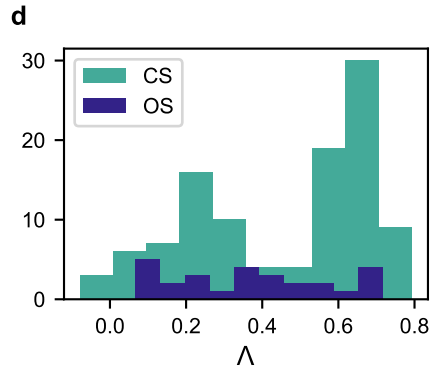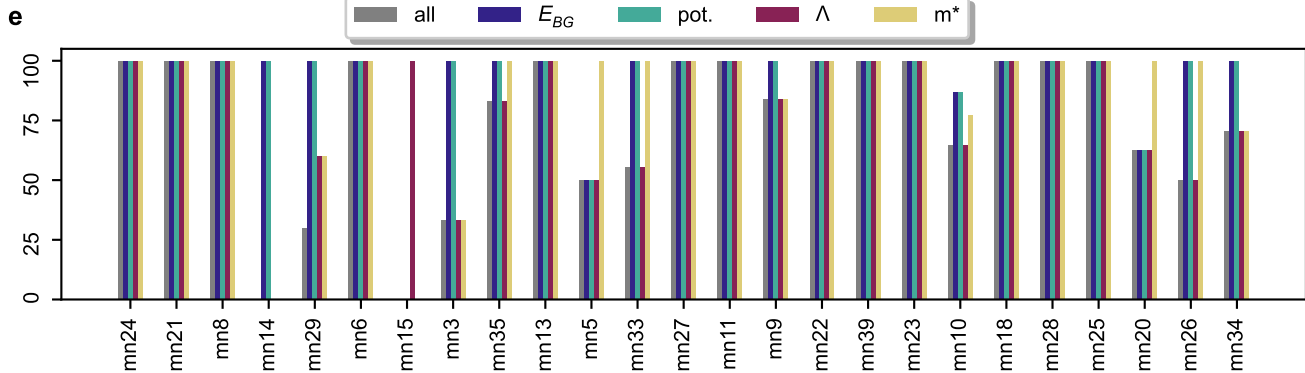

Supplement: SC-016-D5SC01100K-s001 [file SC-016-D5SC01100K-s001.zip › ESI/si_images/stats_all_log.pdf]

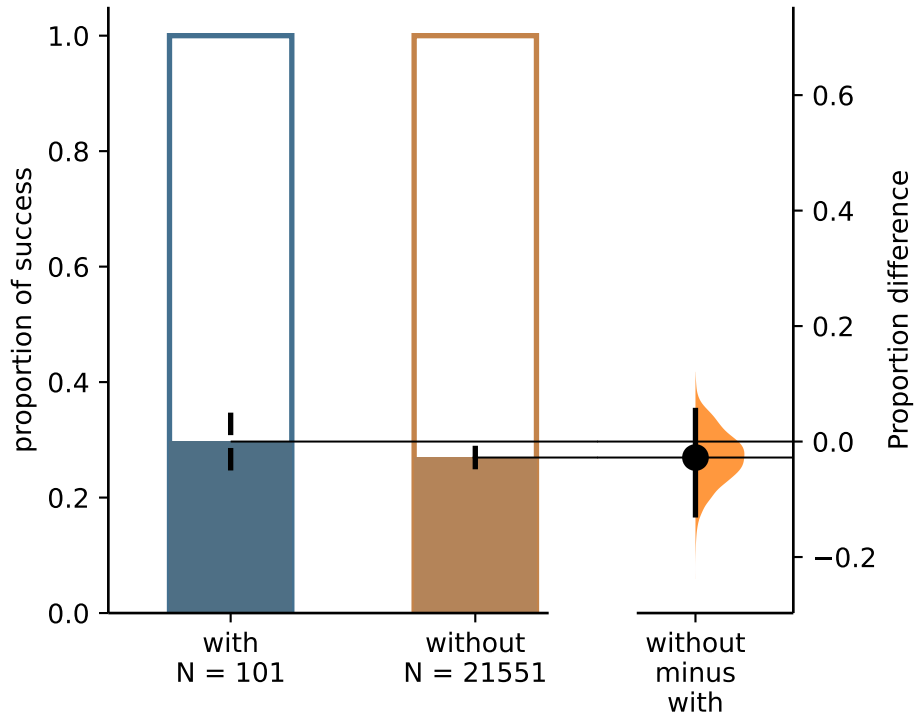

Supplement: SC-016-D5SC01100K-s001 [file SC-016-D5SC01100K-s001.zip › ESI/si_images/structural_analysis/her_gpt_mn14_mean_diff.pdf]

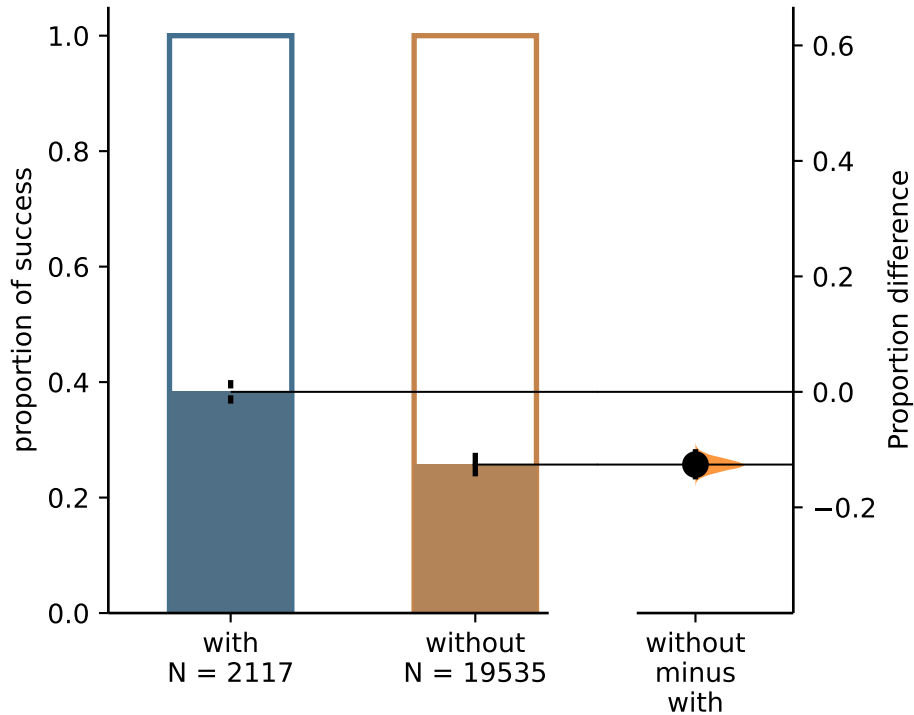

Supplement: SC-016-D5SC01100K-s001 [file SC-016-D5SC01100K-s001.zip › ESI/si_images/structural_analysis/her_gpt_mn44_mean_diff.pdf]

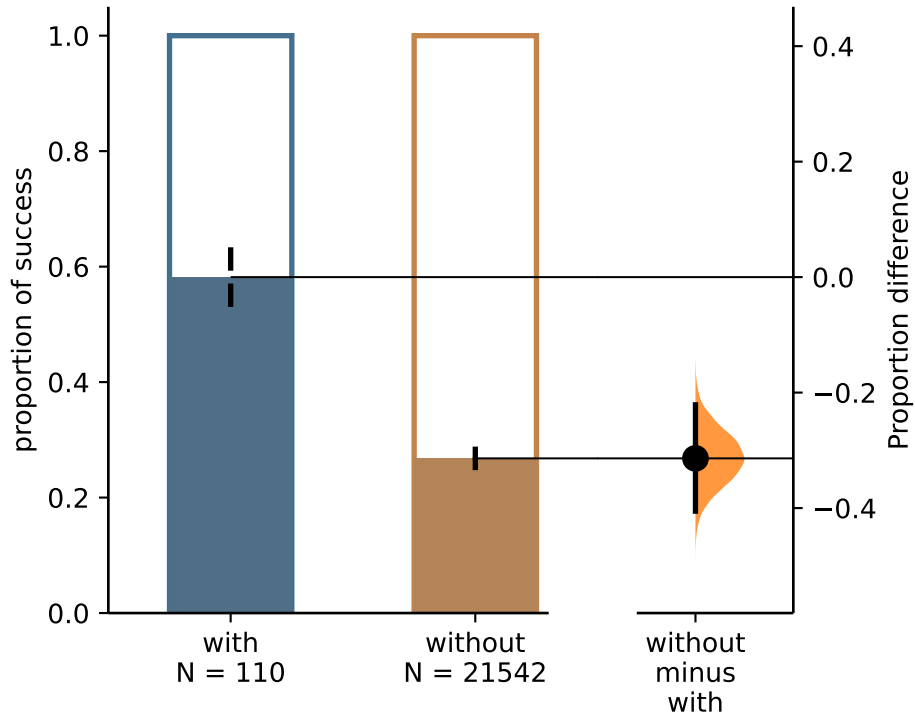

Supplement: SC-016-D5SC01100K-s001 [file SC-016-D5SC01100K-s001.zip › ESI/si_images/structural_analysis/her_gpt_mn53_mean_diff.pdf]

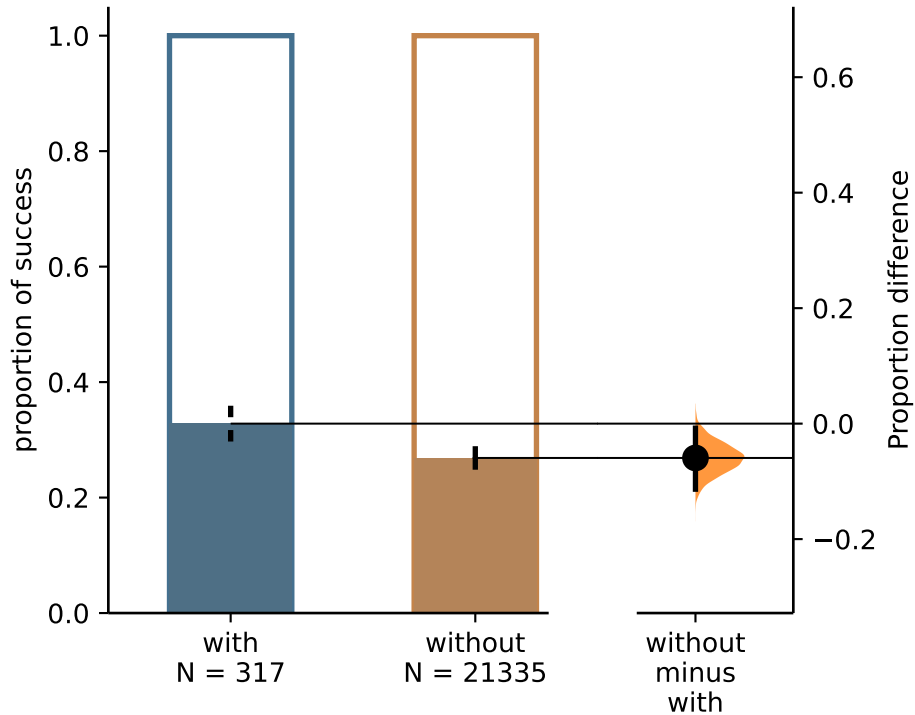

Supplement: SC-016-D5SC01100K-s001 [file SC-016-D5SC01100K-s001.zip › ESI/si_images/structural_analysis/her_gpt_mn60_mean_diff.pdf]

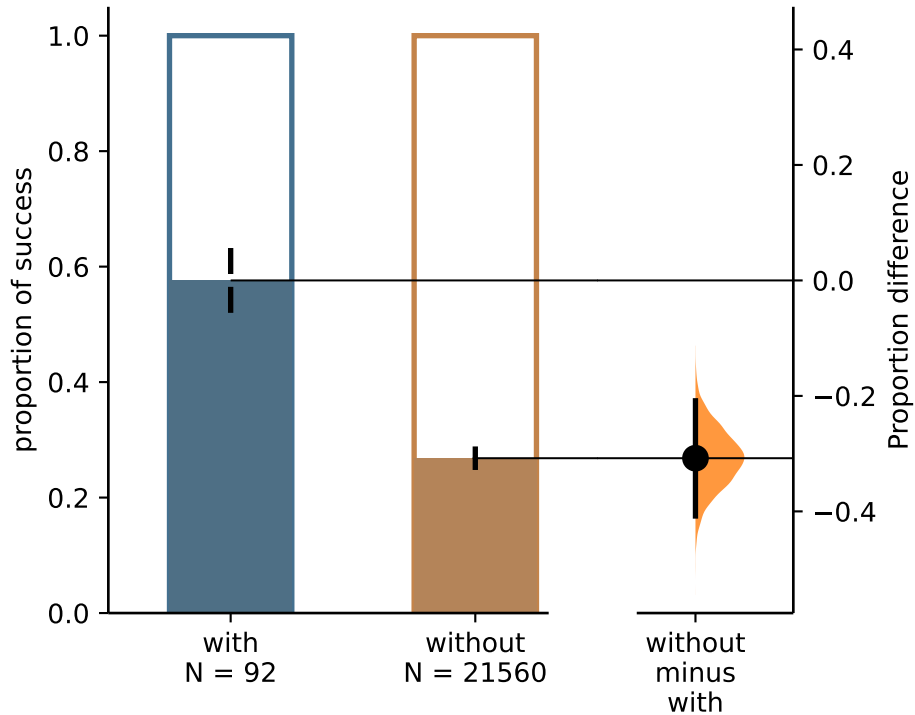

Supplement: SC-016-D5SC01100K-s001 [file SC-016-D5SC01100K-s001.zip › ESI/si_images/structural_analysis/her_gpt_ol17_mean_diff.pdf]

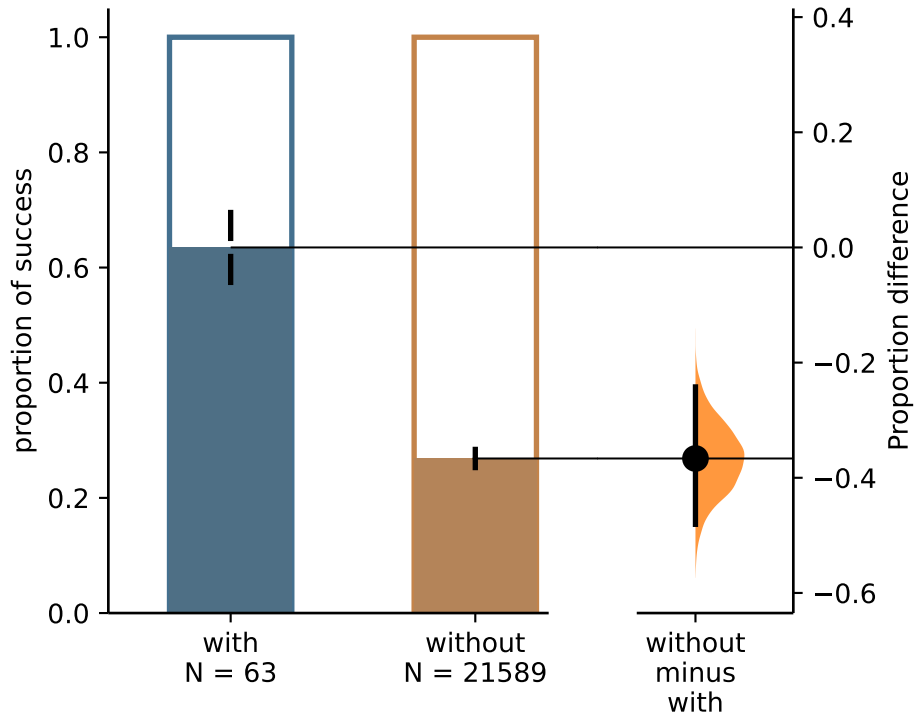

Supplement: SC-016-D5SC01100K-s001 [file SC-016-D5SC01100K-s001.zip › ESI/si_images/structural_analysis/her_gpt_ol30_mean_diff.pdf]

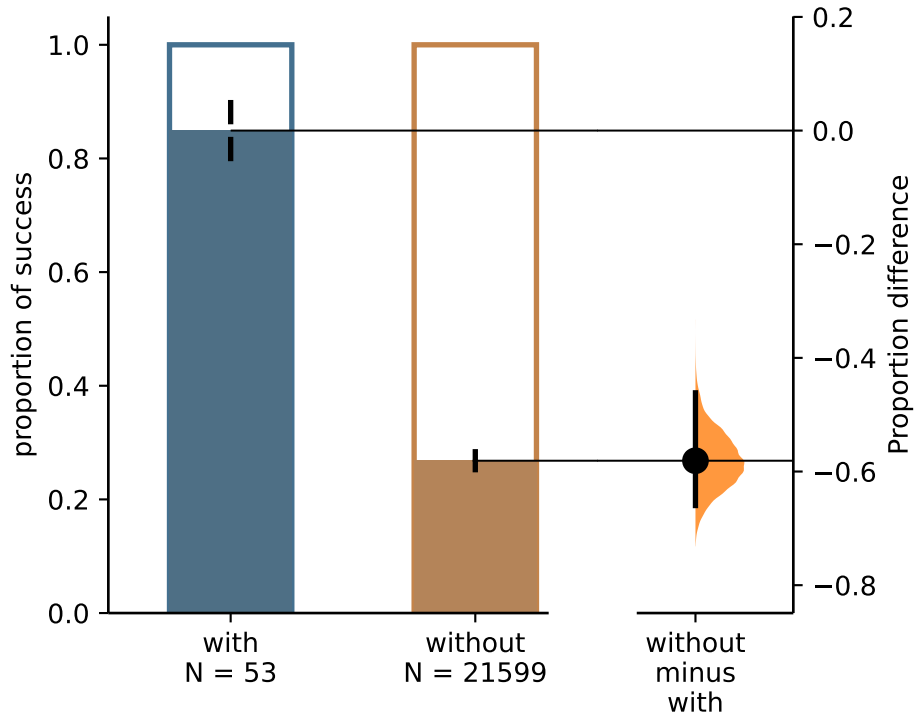

Supplement: SC-016-D5SC01100K-s001 [file SC-016-D5SC01100K-s001.zip › ESI/si_images/structural_analysis/her_gpt_ol45_mean_diff.pdf]

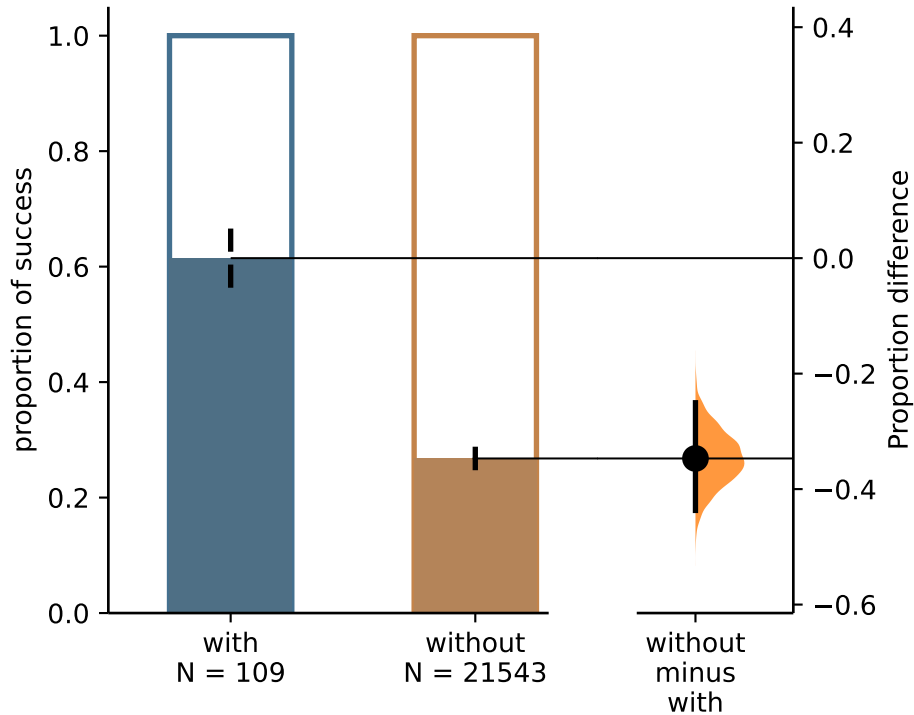

Supplement: SC-016-D5SC01100K-s001 [file SC-016-D5SC01100K-s001.zip › ESI/si_images/structural_analysis/her_gpt_ol5_mean_diff.pdf]

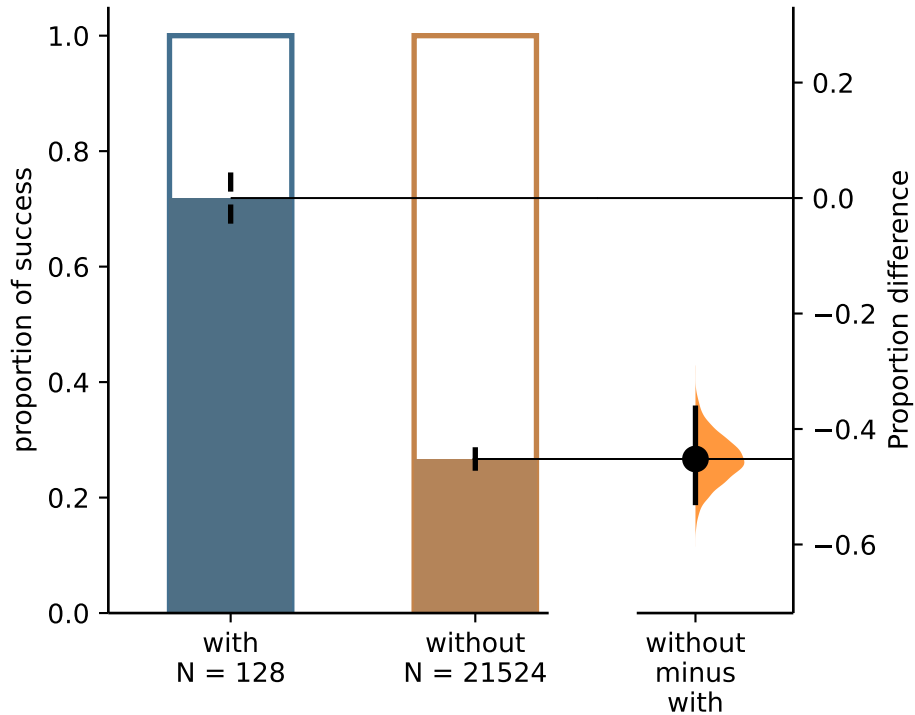

Supplement: SC-016-D5SC01100K-s001 [file SC-016-D5SC01100K-s001.zip › ESI/si_images/structural_analysis/her_gpt_ol68_mean_diff.pdf]

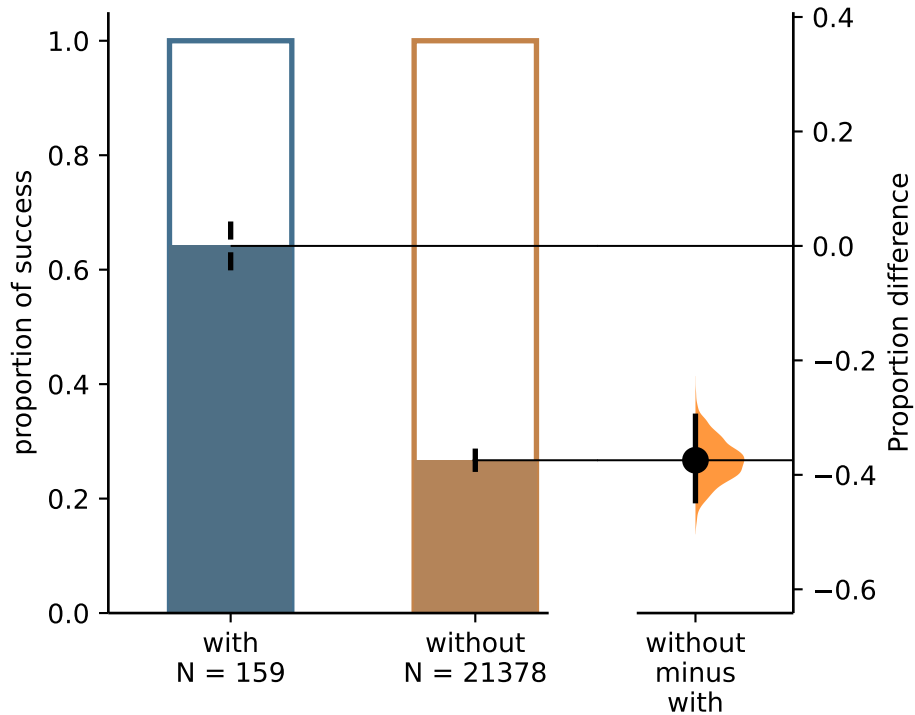

Supplement: SC-016-D5SC01100K-s001 [file SC-016-D5SC01100K-s001.zip › ESI/si_images/structural_analysis/her_gpt_tp15_mean_diff.pdf]

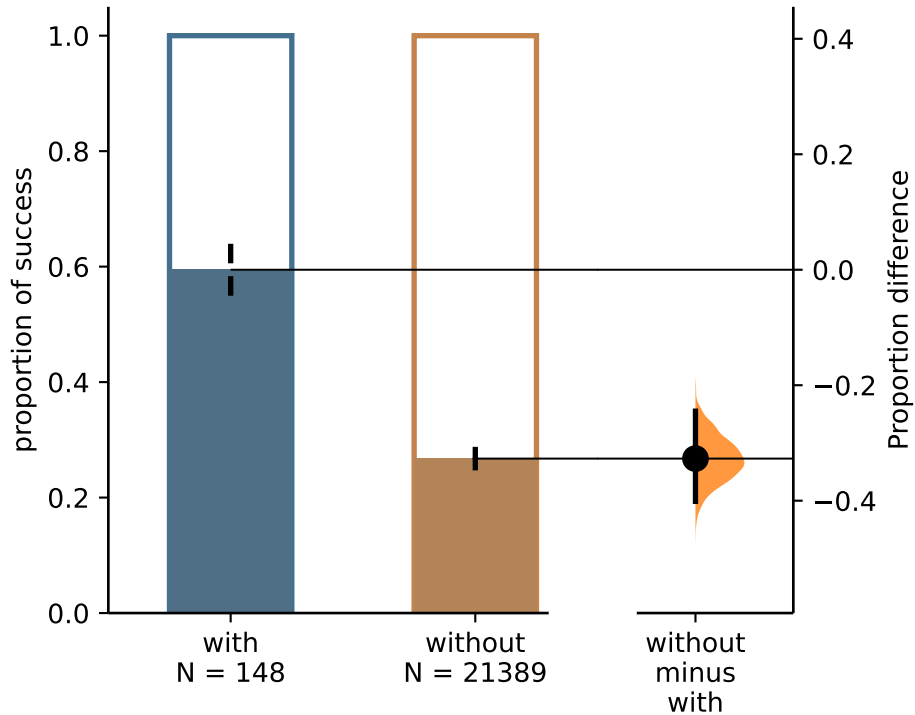

Supplement: SC-016-D5SC01100K-s001 [file SC-016-D5SC01100K-s001.zip › ESI/si_images/structural_analysis/her_gpt_tp24_mean_diff.pdf]

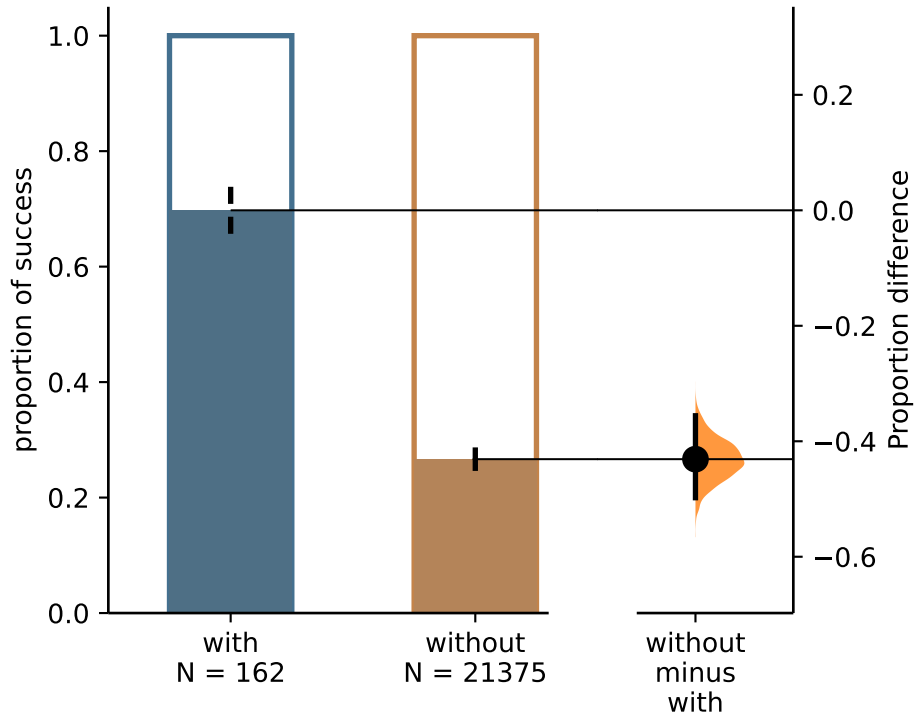

Supplement: SC-016-D5SC01100K-s001 [file SC-016-D5SC01100K-s001.zip › ESI/si_images/structural_analysis/her_gpt_tp8_mean_diff.pdf]

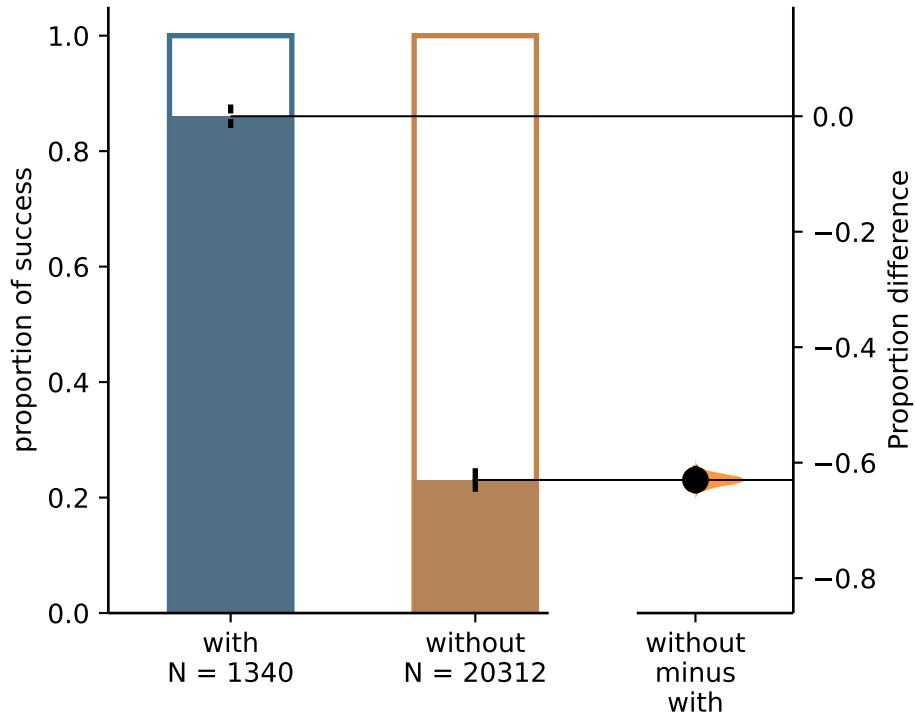

Supplement: SC-016-D5SC01100K-s001 [file SC-016-D5SC01100K-s001.zip › ESI/si_images/structural_analysis/her_moft_mn14_mean_diff.pdf]

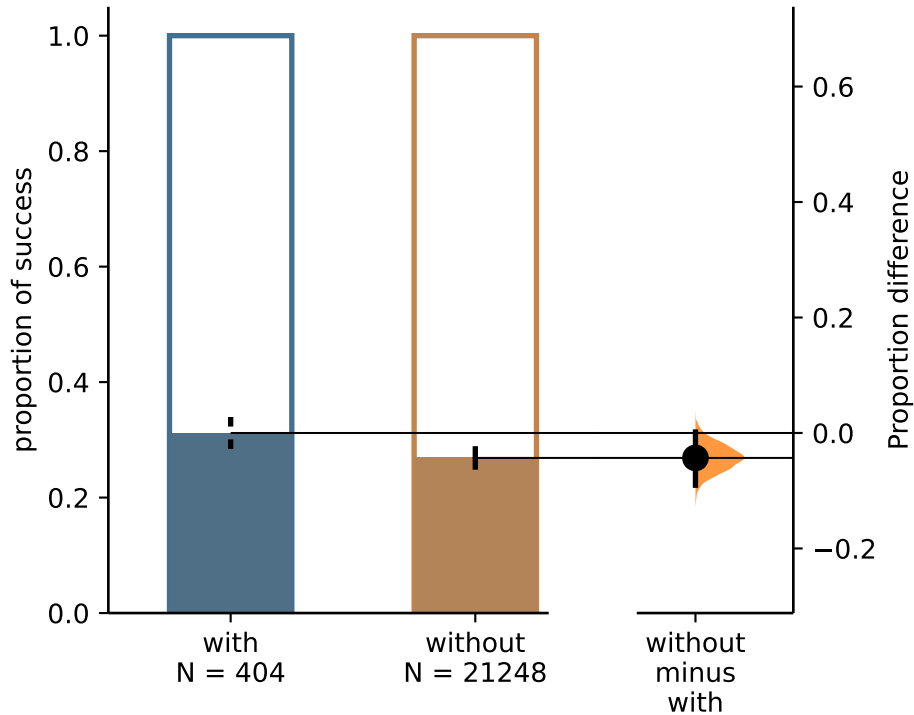

Supplement: SC-016-D5SC01100K-s001 [file SC-016-D5SC01100K-s001.zip › ESI/si_images/structural_analysis/her_moft_mn44_mean_diff.pdf]

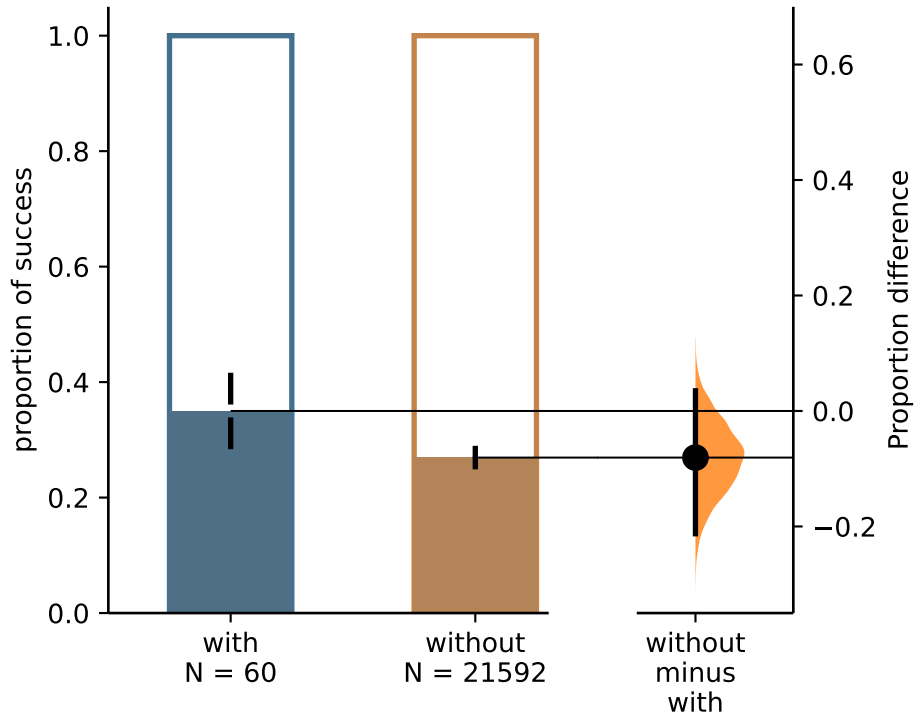

Supplement: SC-016-D5SC01100K-s001 [file SC-016-D5SC01100K-s001.zip › ESI/si_images/structural_analysis/her_moft_mn53_mean_diff.pdf]

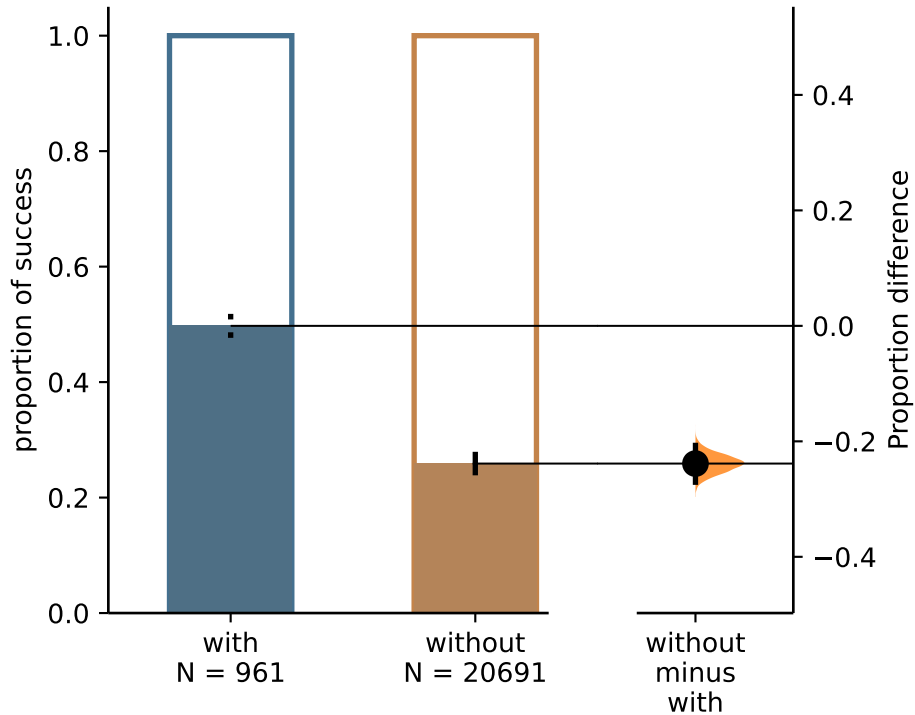

Supplement: SC-016-D5SC01100K-s001 [file SC-016-D5SC01100K-s001.zip › ESI/si_images/structural_analysis/her_moft_mn60_mean_diff.pdf]

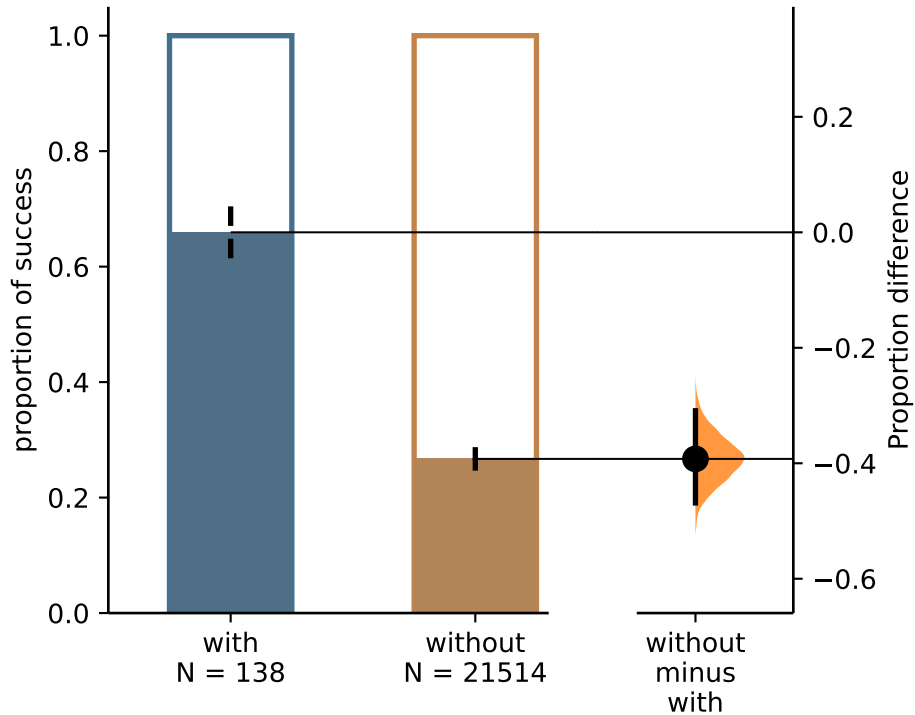

Supplement: SC-016-D5SC01100K-s001 [file SC-016-D5SC01100K-s001.zip › ESI/si_images/structural_analysis/her_moft_ol17_mean_diff.pdf]

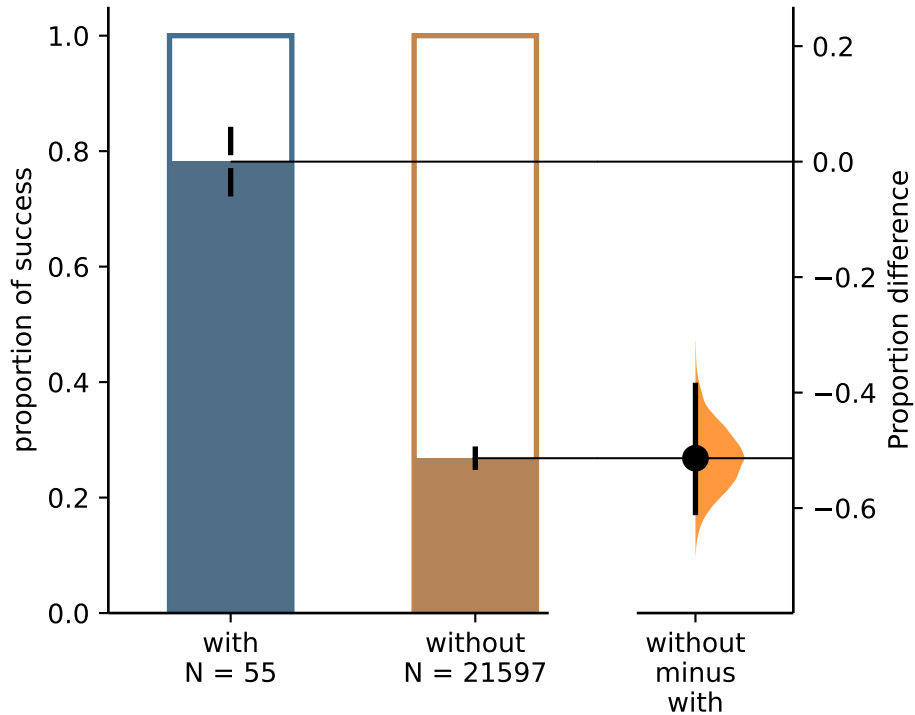

Supplement: SC-016-D5SC01100K-s001 [file SC-016-D5SC01100K-s001.zip › ESI/si_images/structural_analysis/her_moft_ol30_mean_diff.pdf]

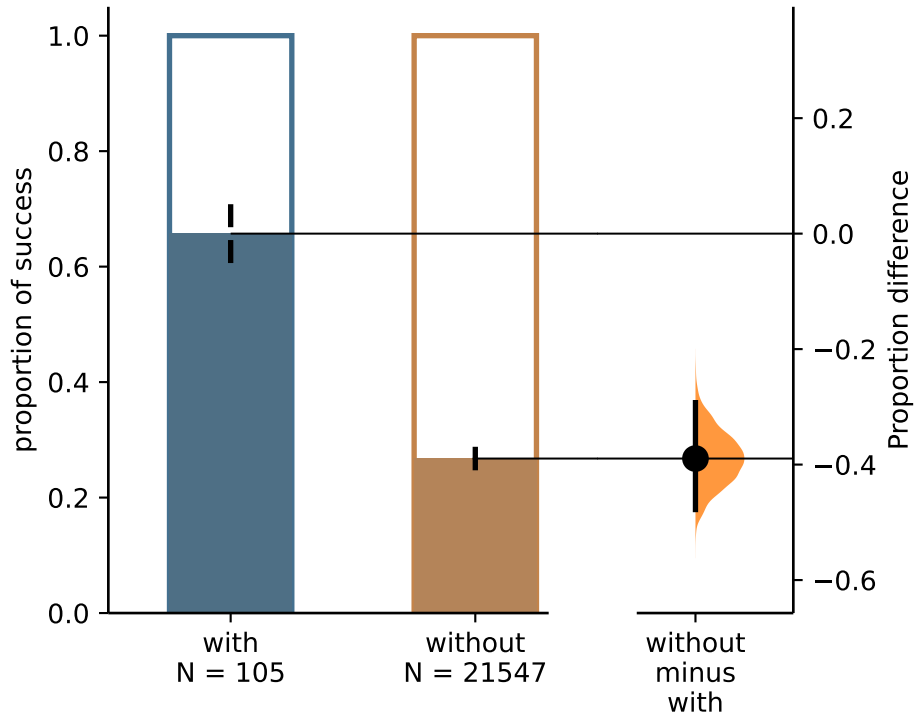

Supplement: SC-016-D5SC01100K-s001 [file SC-016-D5SC01100K-s001.zip › ESI/si_images/structural_analysis/her_moft_ol45_mean_diff.pdf]

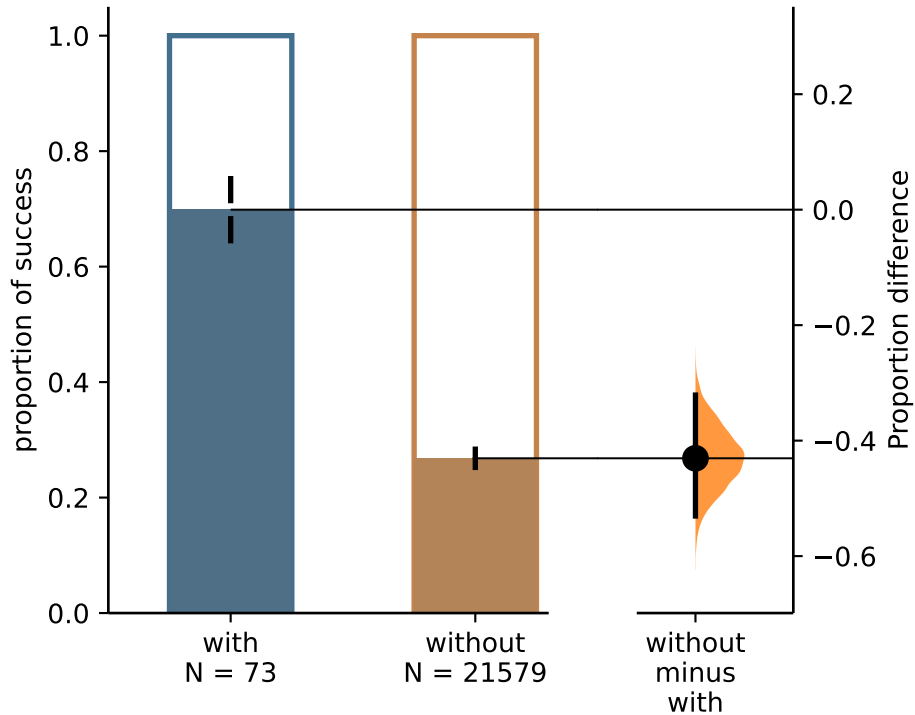

Supplement: SC-016-D5SC01100K-s001 [file SC-016-D5SC01100K-s001.zip › ESI/si_images/structural_analysis/her_moft_ol5_mean_diff.pdf]

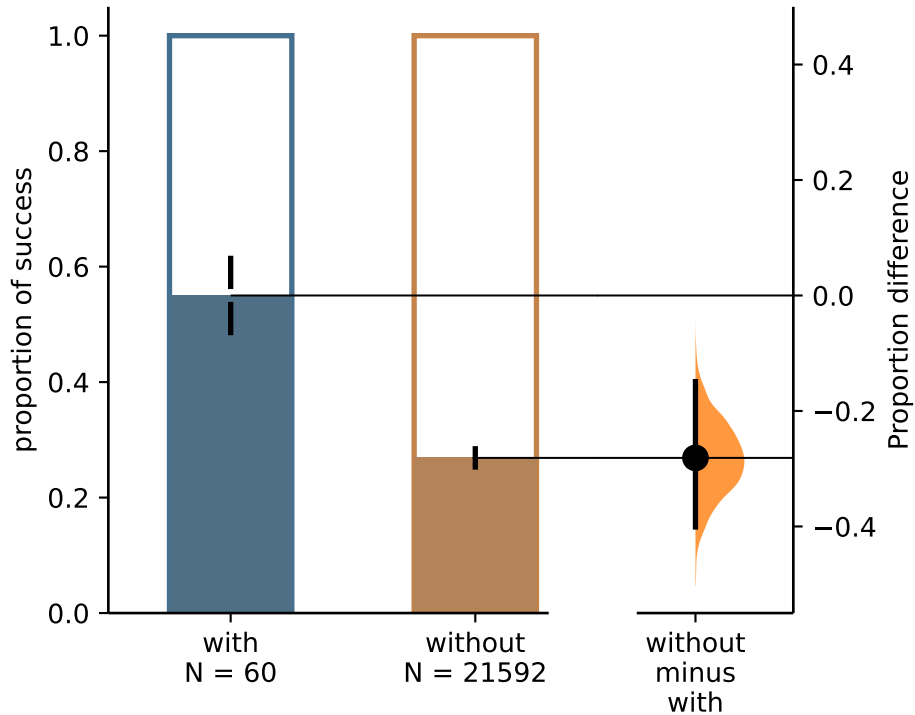

Supplement: SC-016-D5SC01100K-s001 [file SC-016-D5SC01100K-s001.zip › ESI/si_images/structural_analysis/her_moft_ol68_mean_diff.pdf]

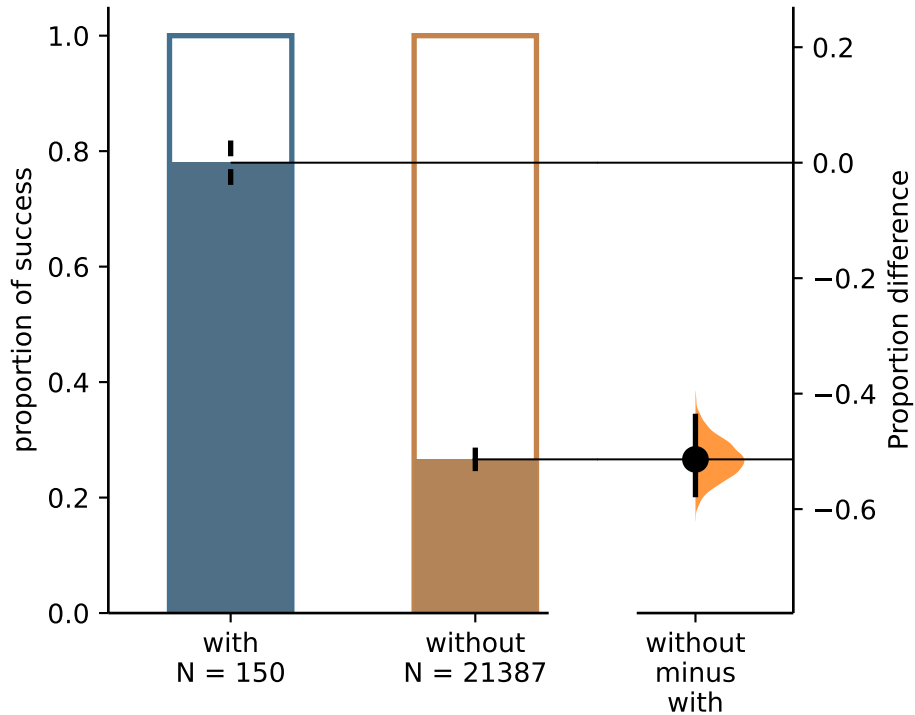

Supplement: SC-016-D5SC01100K-s001 [file SC-016-D5SC01100K-s001.zip › ESI/si_images/structural_analysis/her_moft_tp15_mean_diff.pdf]

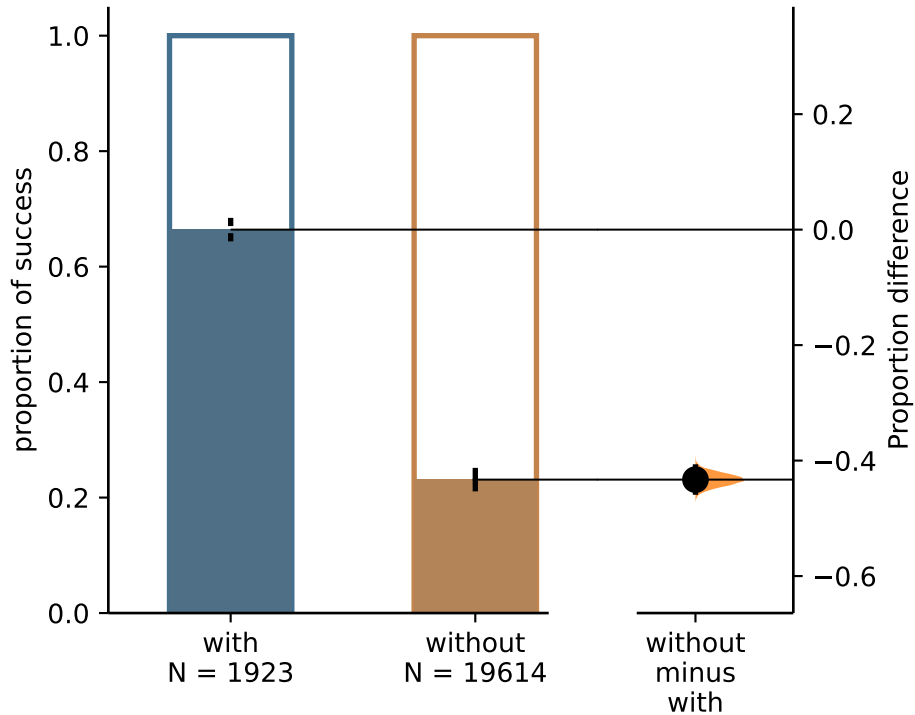

Supplement: SC-016-D5SC01100K-s001 [file SC-016-D5SC01100K-s001.zip › ESI/si_images/structural_analysis/her_moft_tp24_mean_diff.pdf]

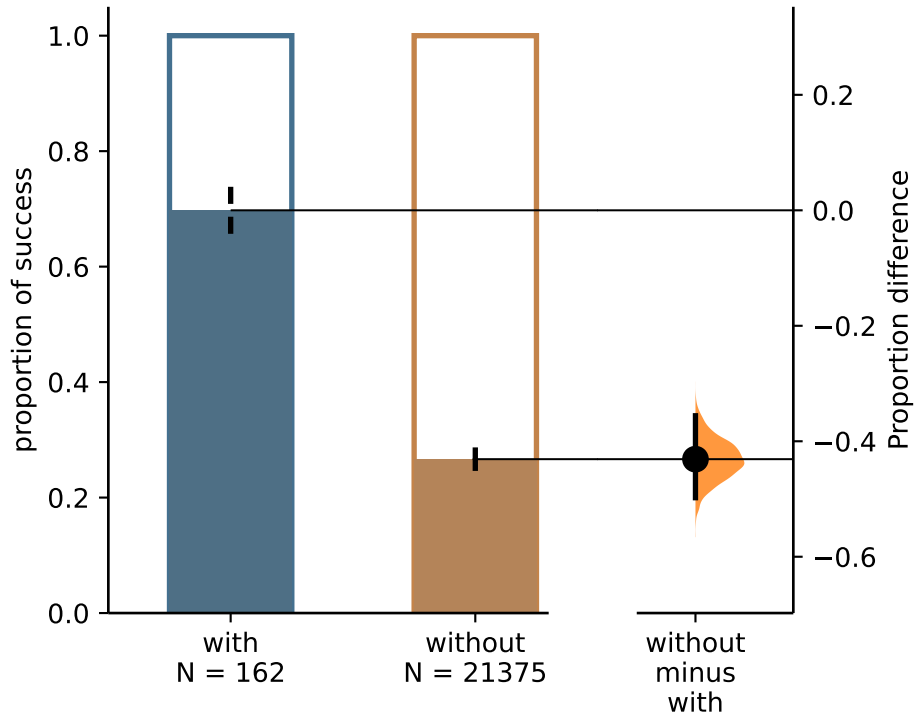

Supplement: SC-016-D5SC01100K-s001 [file SC-016-D5SC01100K-s001.zip › ESI/si_images/structural_analysis/her_moft_tp8_mean_diff.pdf]

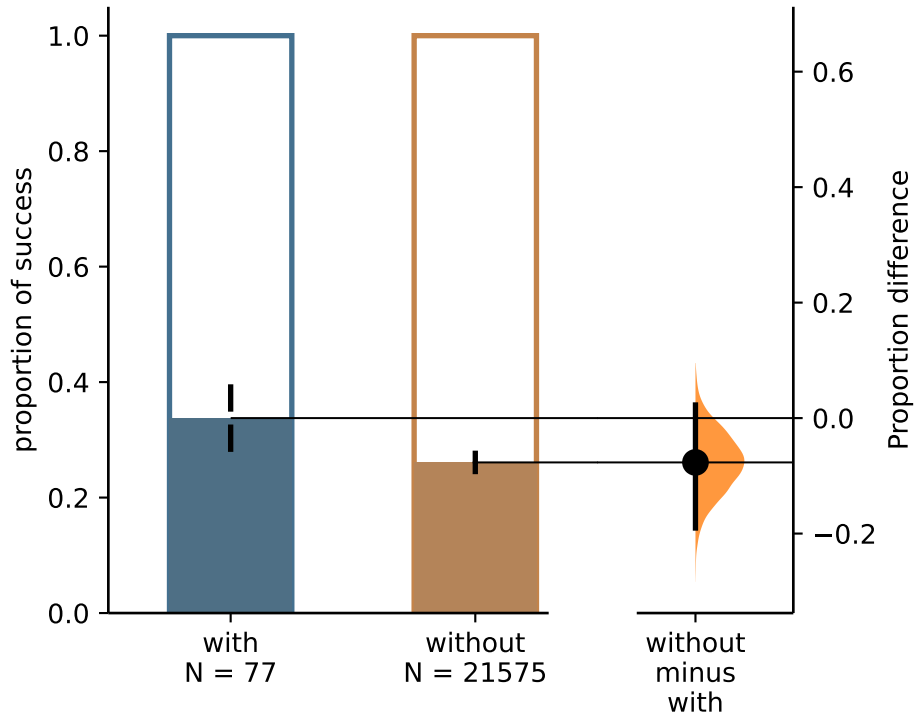

Supplement: SC-016-D5SC01100K-s001 [file SC-016-D5SC01100K-s001.zip › ESI/si_images/structural_analysis/mred_gpt_mn15_mean_diff.pdf]

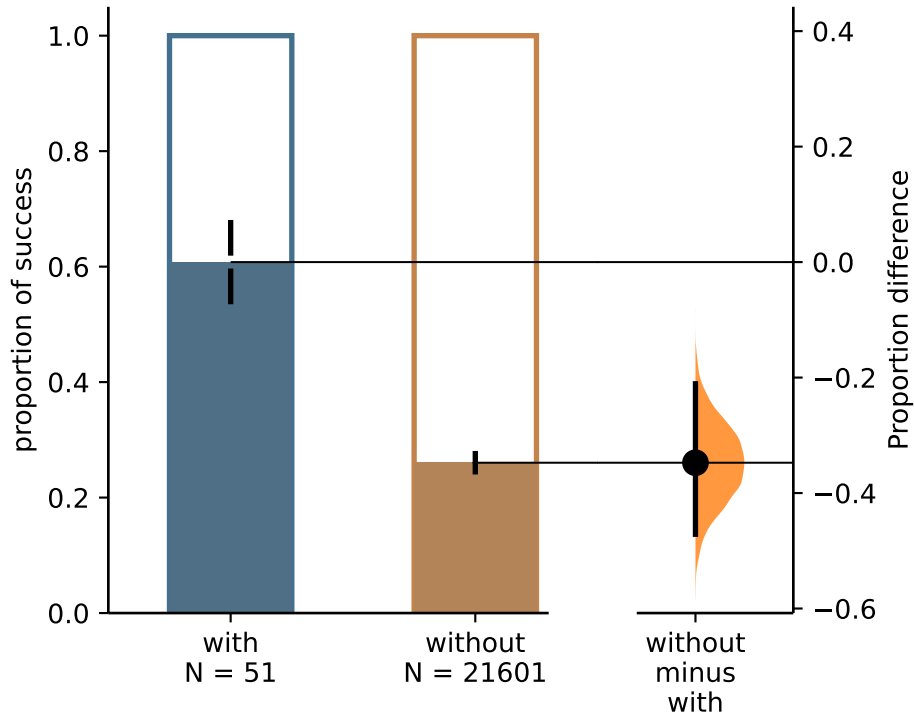

Supplement: SC-016-D5SC01100K-s001 [file SC-016-D5SC01100K-s001.zip › ESI/si_images/structural_analysis/mred_gpt_mn7_mean_diff.pdf]

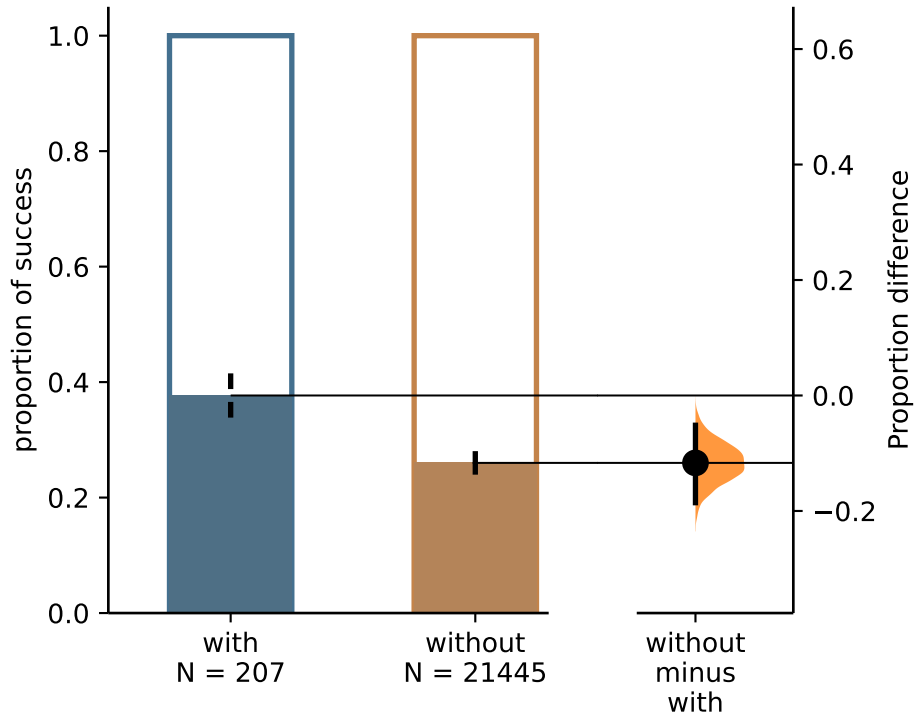

Supplement: SC-016-D5SC01100K-s001 [file SC-016-D5SC01100K-s001.zip › ESI/si_images/structural_analysis/mred_gpt_ol18_mean_diff.pdf]

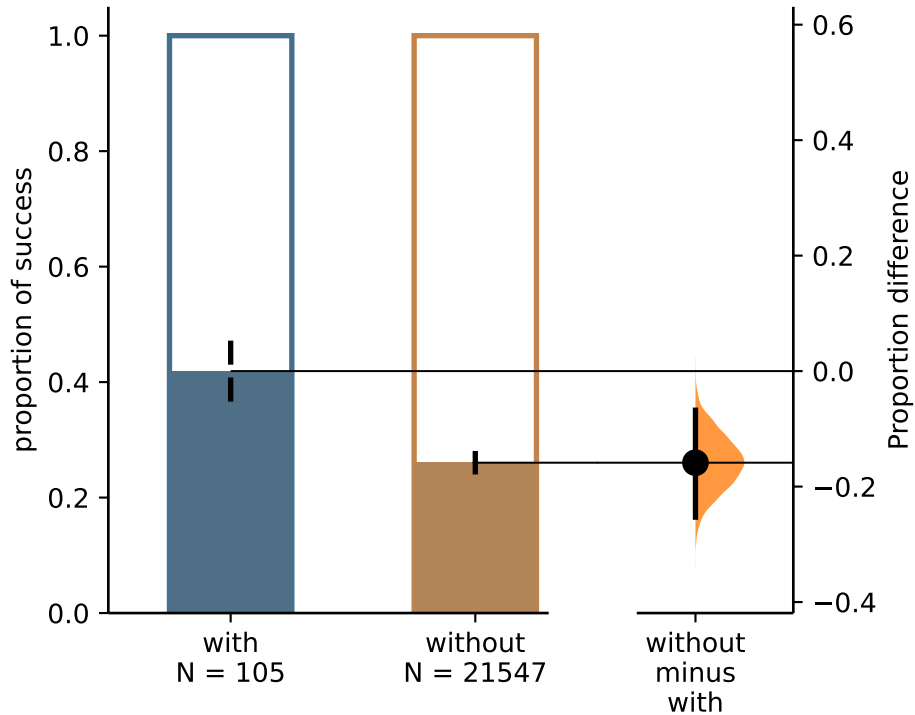

Supplement: SC-016-D5SC01100K-s001 [file SC-016-D5SC01100K-s001.zip › ESI/si_images/structural_analysis/mred_gpt_ol48_mean_diff.pdf]

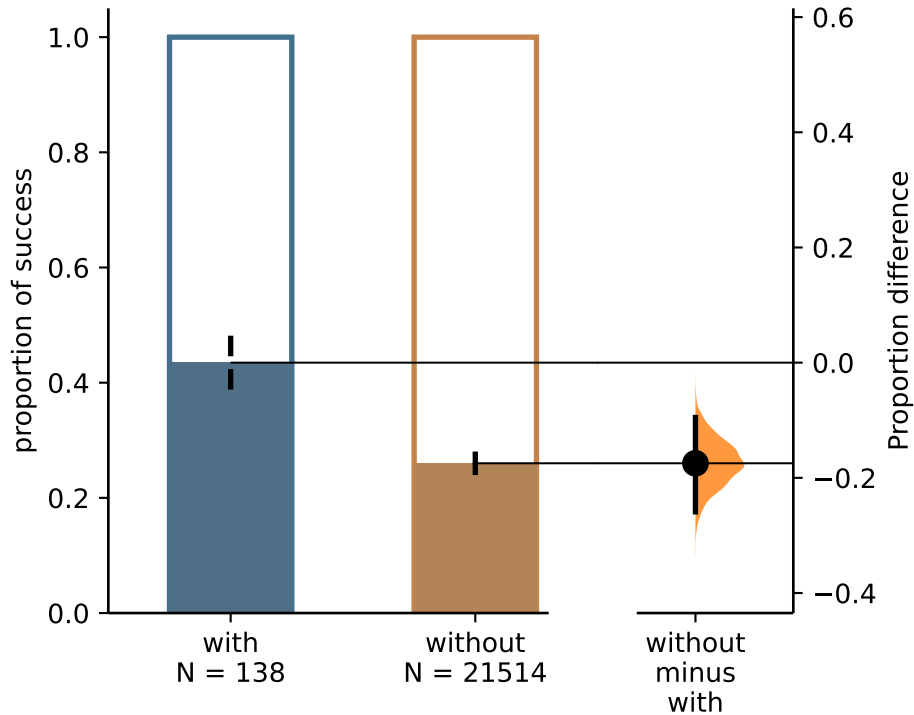

Supplement: SC-016-D5SC01100K-s001 [file SC-016-D5SC01100K-s001.zip › ESI/si_images/structural_analysis/mred_gpt_ol58_mean_diff.pdf]

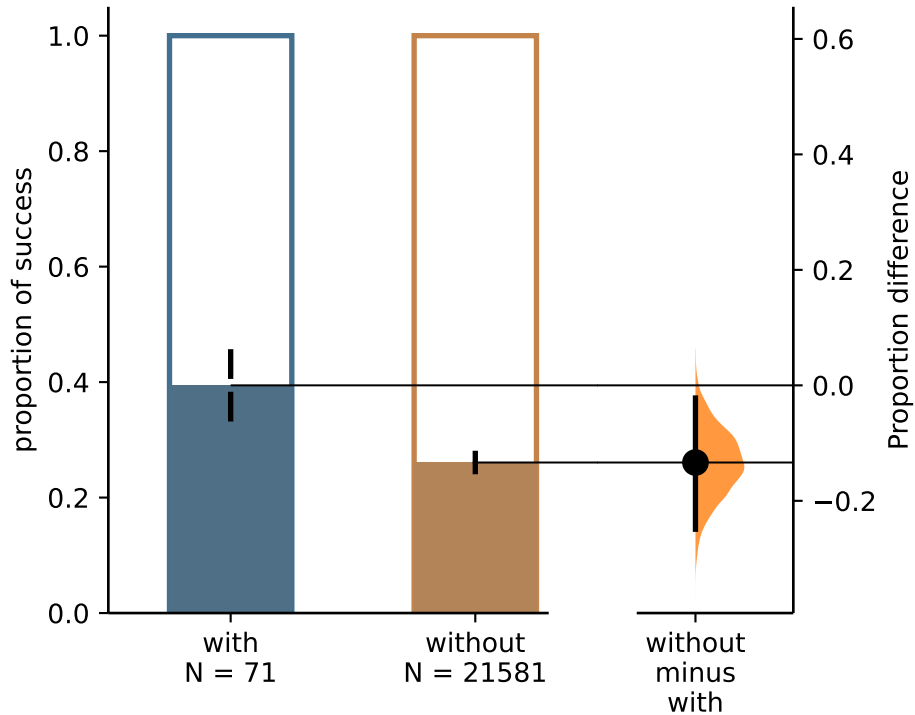

Supplement: SC-016-D5SC01100K-s001 [file SC-016-D5SC01100K-s001.zip › ESI/si_images/structural_analysis/mred_gpt_ol70_mean_diff.pdf]

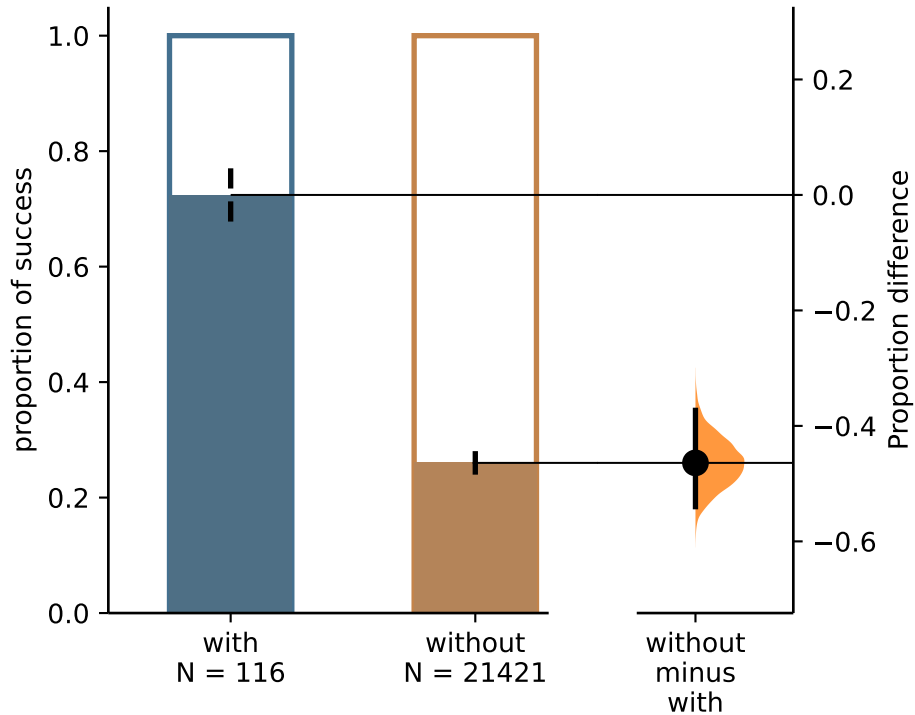

Supplement: SC-016-D5SC01100K-s001 [file SC-016-D5SC01100K-s001.zip › ESI/si_images/structural_analysis/mred_gpt_tp17_mean_diff.pdf]

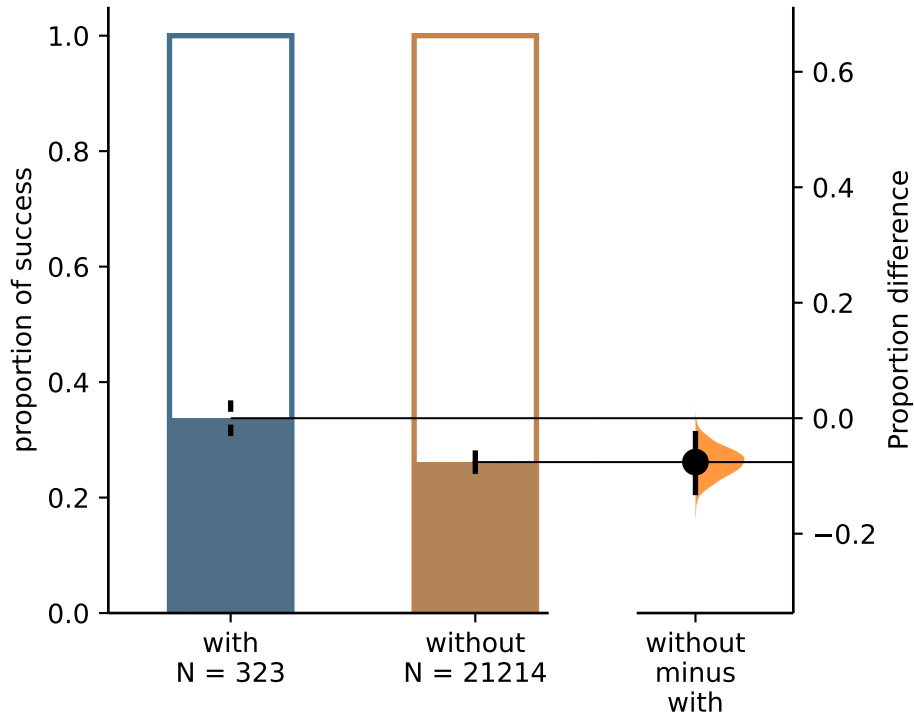

Supplement: SC-016-D5SC01100K-s001 [file SC-016-D5SC01100K-s001.zip › ESI/si_images/structural_analysis/mred_gpt_tp28_mean_diff.pdf]

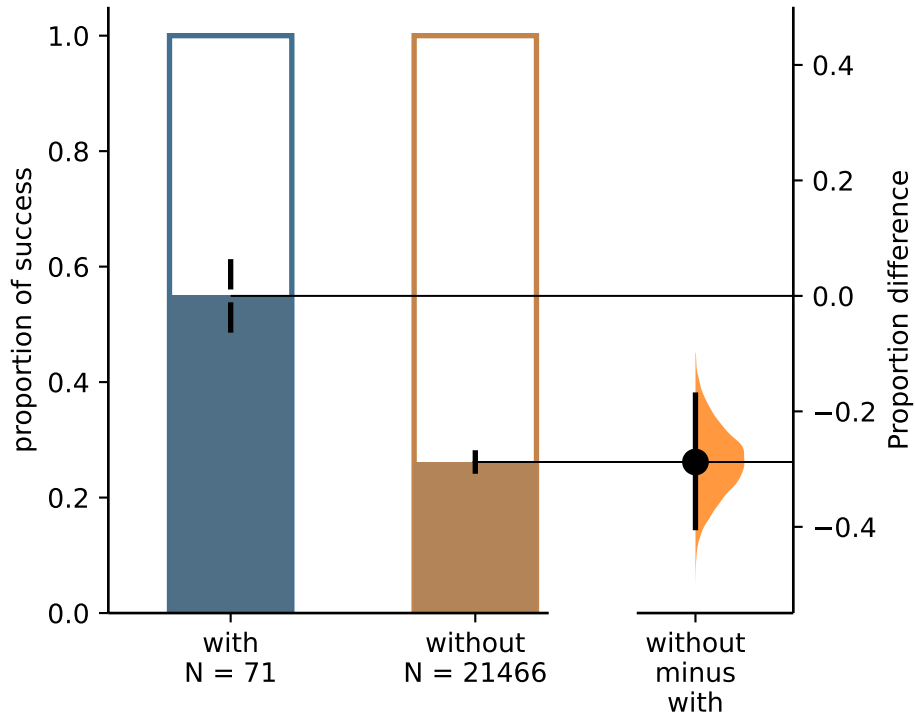

Supplement: SC-016-D5SC01100K-s001 [file SC-016-D5SC01100K-s001.zip › ESI/si_images/structural_analysis/mred_gpt_tp2_mean_diff.pdf]
