# Supplementary material for: Exploring the chemical design space of metal–organic frameworks for photocatalysis
Source: Chem Sci. 2025 May 13;16(25):11434–46. doi: 10.1039/d5sc01100k (PMC12107286; doi:10.1039/d5sc01100k)
Supplement: SC-016-D5SC01100K-s002 [file SC-016-D5SC01100K-s002.pdf]

Supplementary Information (SI) for Chemical Science.
This journal is © The Royal Society of Chemistry 2025

Supporting Information:

Supporting Information: Exploring the Chemical Design Space of Metal-Organic Frameworks for Photocatalysis

Beatriz Mourino,[†,§] Sauradeep Majumdar,[†,§] Xin Jin,[†] Fergus McIlwaine,[‡] Joren Van Herck,[†] Andres Ortega-Guerrero,[†,¶] Susana Garcia,[‡] and Berend Smit[*,†]

†*Laboratory of Molecular Simulation (LSMO), Institut des Sciences et Ingénierie Chimiques, Valais Ecole Polytechnique Fédérale de Lausanne (EPFL), Rue de l'Industrie 17, CH-1951 Sion, Valais, Switzerland*

‡*The Research Centre for Carbon Solutions(RCCS), School of Engineering and Physical Sciences, Heriot-Watt University, EH144AS Edinburgh, United Kingdom*

¶*Nanotech@surfaces Laboratory, Empa - Swiss Federal Laboratories for Materials Science and Technology, 8600 Dübendorf, Switzerland*

§*These authors contributed equally to this work*

E-mail: berend.smit@epfl.ch

Contents

1 Database details **S4**

1.1 Metal nodes S4

1.2 Linkers S9

1.3 Topologies S16

1.4 Structure generation S17

1.5 Diversity analysis S19

1.6 Experimental MOFs S20

2 Photocatalysis assessment **S21**

2.1 Evaluation of photocatalytic descriptors S21

2.1.1 A comment on KS band gaps S21

2.2 DFT calculations S22

2.3 Open shell workflow S24

2.4 Further DFT details on photocatalytic descriptors S26

2.5 Experimental information on promising building blocks S29

2.6 Comparison with experimental and QMOF subsets S30

3 Supervised machine learning **S31**

3.1 GPT-J S32

3.2 MOFTransformer S33

3.3 Embeddings S34

3.4 Model comparison S36

3.5 DFT evaluation of MOFs predicted to meet all criteria S37

4 Structural analysis **S39**

4.1 Effect of CDP–MOF building blocks on the photocatalytic descriptors S39

4.1.1 Metal nodes S42

4.1.2 Organic linkers S44
4.1.3 Topologies S46
4.1.4 Bootstrapped effect sizes S48
4.2 Structure-property relationship analysis on all predictions S56
4.2.1 Organic linkers S57
4.2.2 Metal nodes S67
4.2.3 MOFs that met all criteria S68

5 Packages **S73**

References **S74**

1 Database details

Figures S2–S12 show the different building blocks—metal nodes and organic linkers— used to design our database. These were manually checked for missing atoms, and charges were assigned to the building blocks in case they were not charge-neutral. In some cases, we optimized the metal nodes and organic linkers using DFT to obtain a more reasonable geometry. Thus, our building blocks were 'healed'.[S1] These building blocks were then used to build MOF structures using the ToBaCCo algorithm.[S2]

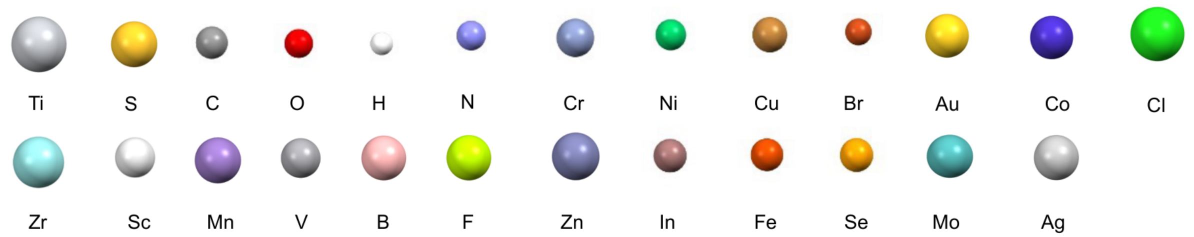

Figure S1: Color coding of atoms used in the different organic molecules and metal nodes

1.1 Metal nodes

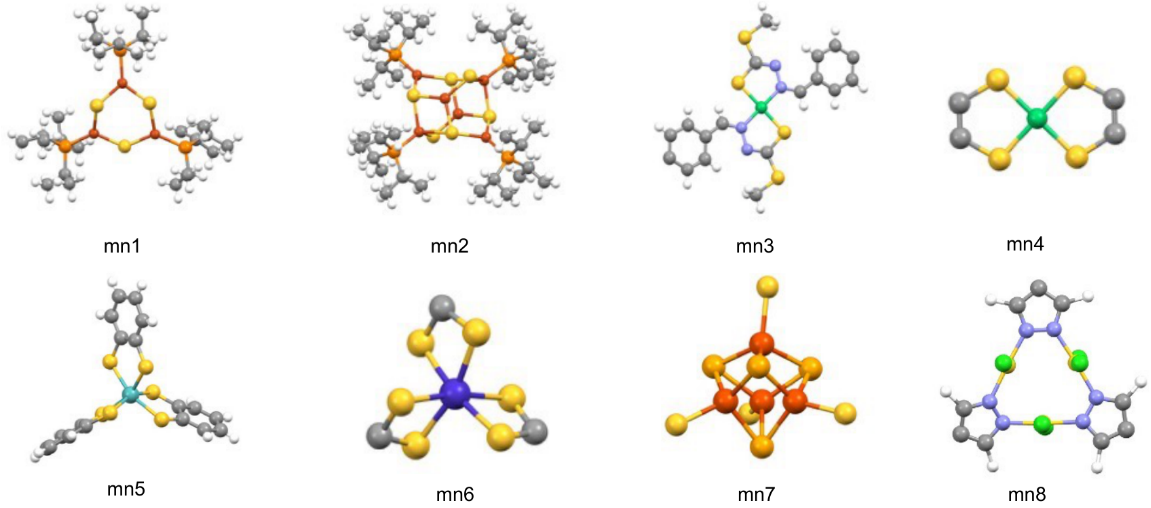

Figure S2: Structures of metal nodes used in this work.

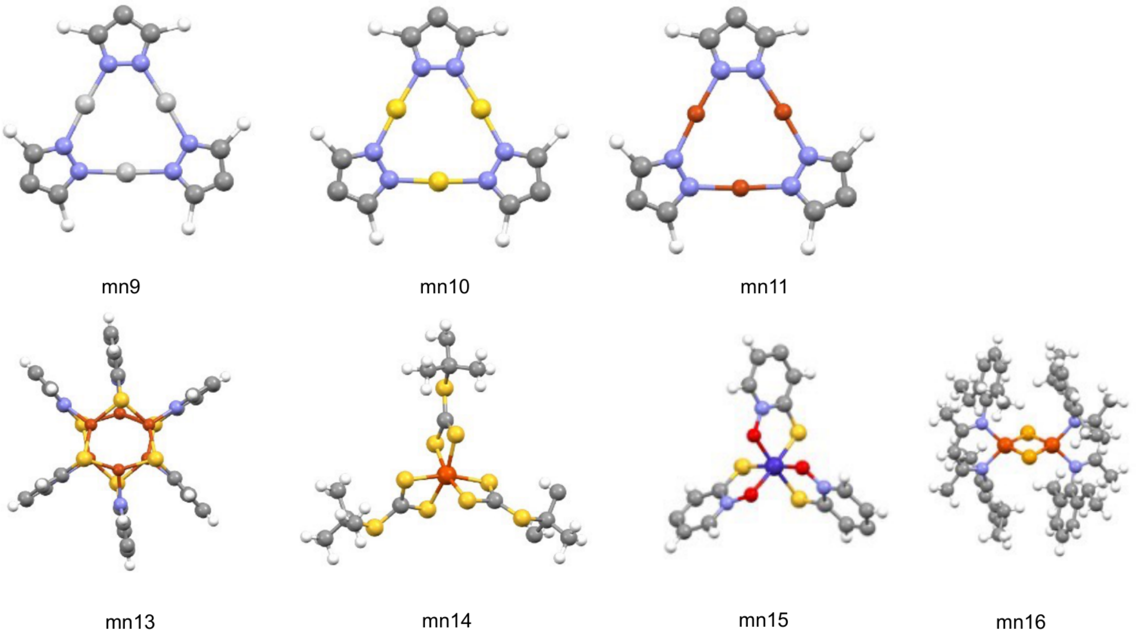

Figure S3: Structures of metal nodes used in this work (contd.).

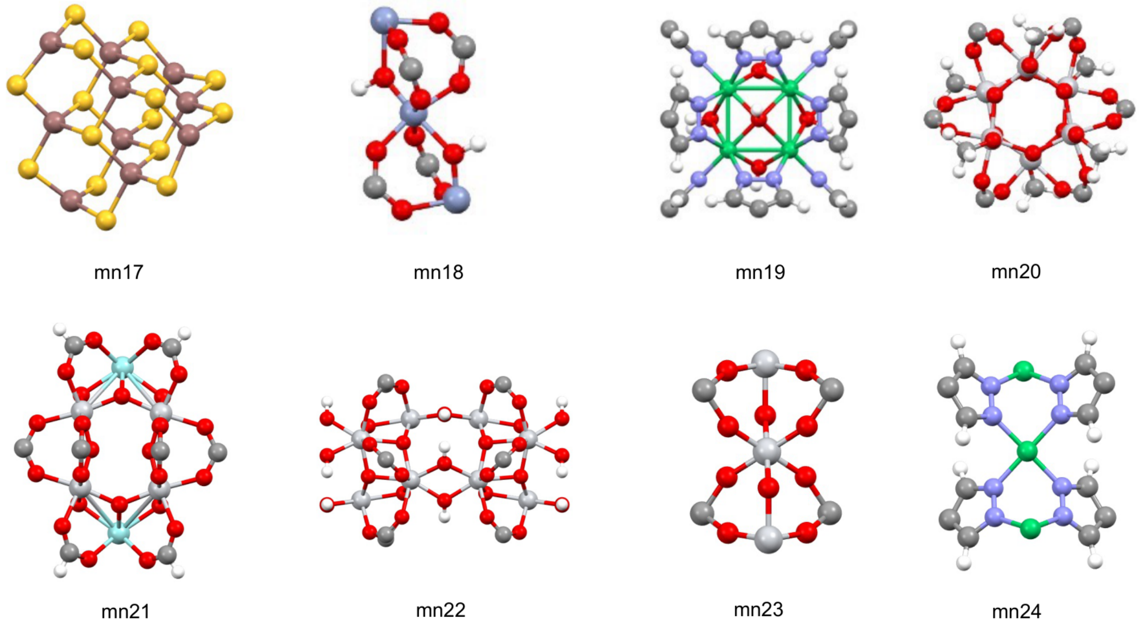

Figure S4: Structures of metal nodes used in this work (contd.).

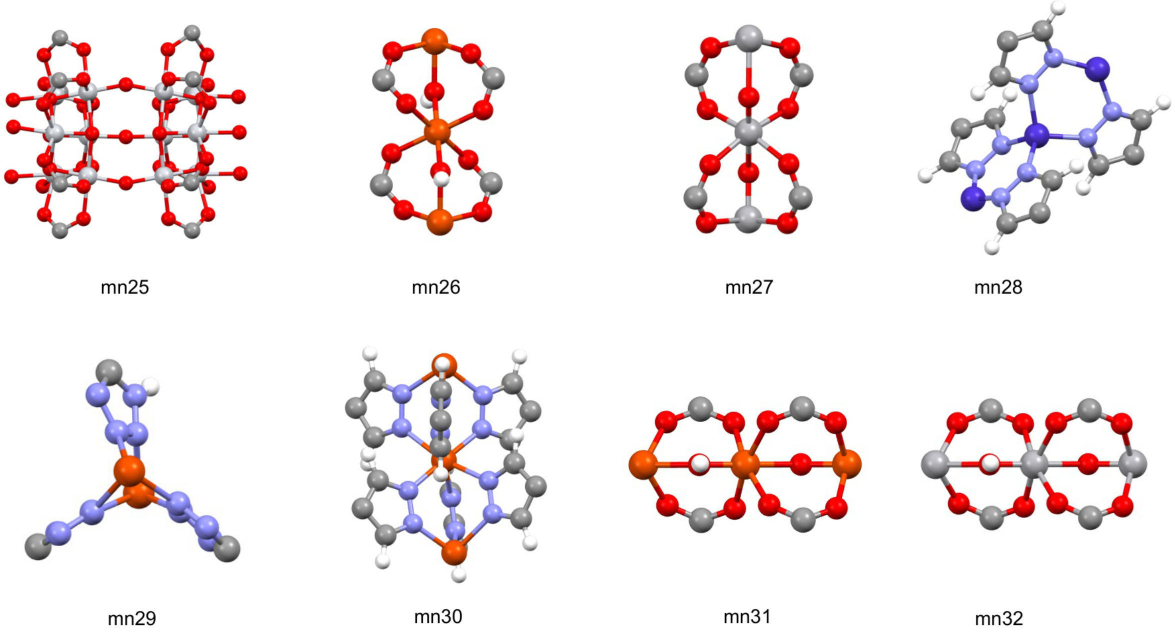

Figure S5: Structures of metal nodes used in this work (contd.).

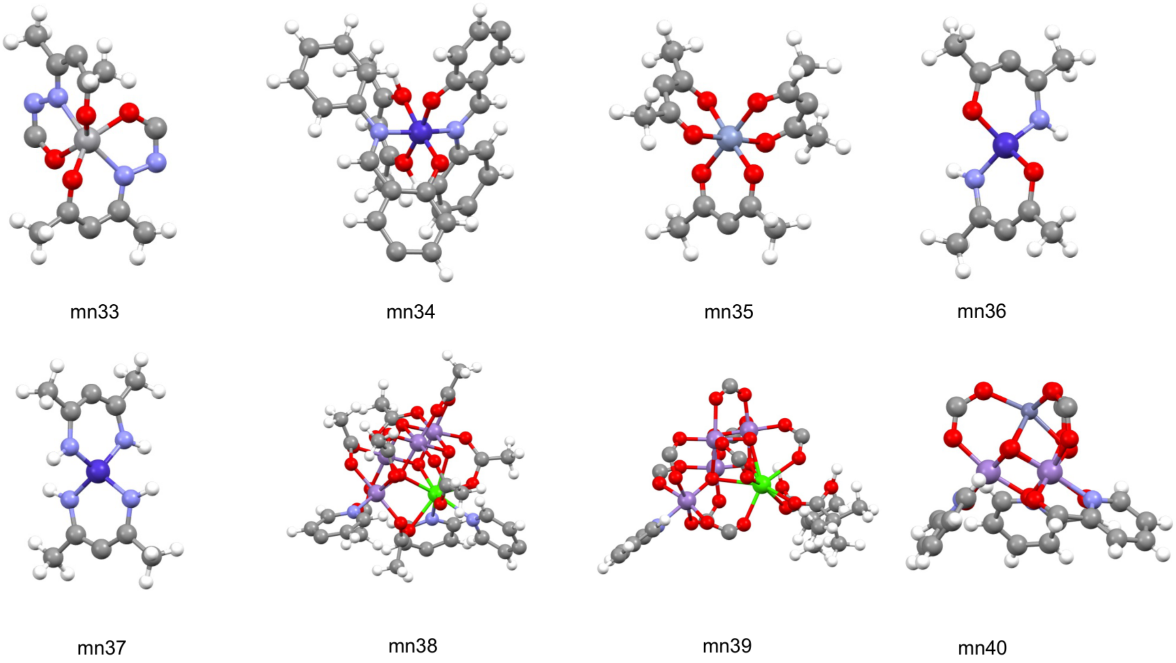

Figure S6: Structures of metal nodes used in this work (contd.).

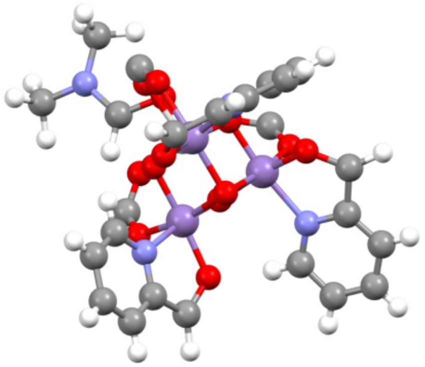

mn41

Figure S7: Structures of metal nodes used in this work (contd.).

Table S1: Naming of metal nodes

**Metal node**	**CSD Name**	**Metal** [a]	**Ox. state** [b]	**Used in MOFs?**	**Net charge**
mn1	ABOGAB	Cu	I	No	0
mn2	ABOGEF	Cu	I	No	0
mn3⋄	ACEWIO	Ni	II	No	0
mn4	ACIFID	Ni	II	Yes	0
mn5⋄	ACODAX	Mo	(V/VI)	Yes	-1/0
mn6⋄	ACTXCO10	Co	III	No	0
mn7	AFESES	Fe	(II/III)	No	-2
mn8⋄	AHAGUL	Au	III	No	0
mn9⋄	modified AHAGUL	Ag	I	Yes	0
mn10⋄	modified AHAGUL	Au	I	No	0
mn11⋄	modified AHAGUL	Cu	I	Yes	0
mn13⋄	AWUGEH	Cu	I	No	0
mn14⋄	BTXANI	Fe	III	Yes	0
mn15⋄	CAQBOL	Co	III	No	0
mn16	CEHHUV	Fe	III	No	0
mn17	EMIGOX	In	III	Yes	-4
mn18⋄, +	GUSNEN	Cr	III	Yes	0
mn19	MALMAQ	Ni	II	Yes	0
mn20⋄	YUFMEU	Ti	IV	Yes	0
mn21⋄, +	WOTTOR	Zr,Ti	IV,IV	Yes	0
mn22⋄, +	TOYHEX	Ti	IV	Yes	0
mn23⋄, +	KIHXUX	Ti	IV	Yes	0
mn24⋄, +	SUTBIT	Ni	II	Yes	0
mn25⋄, +	NOPCIH	Ti	IV	Yes	0
mn26⋄, +	POJTOY	Fe	III	Yes	0
mn27⋄, +	IDIWOH	V	IV	Yes	0
mn28⋄, +	COJHIT	Co	II	Yes	0
mn29⋄, +	ZARTAP	Fe	II	Yes	0
mn30+	VICDOC	Fe	III	Yes	0
mn31+	XADCOW	Fe	III	Yes	0
mn32+	ATOTIM	V	III	Yes	0
mn33⋄	ABHYZV	V	IV	No	0
mn34⋄	ABOQAJ	Co	III	No	0
mn35⋄	ACACCR	Cr	III	No	0
mn36	ADEQOS	Co	II	No	0
mn37	ADERAF	Co	II	No	0
mn38	IHUZUI	Mn,Ca	(III/IV),II	No	0
mn39⋄	IJABAY	Mn,Ca	(III/IV),II	No	0
mn40	FINKIY	Zn,Mn	II,IV	No	0
mn41	FINKAQ	Sc,Mn	II,(III/IV)	No	0

[a]⋄ refers to nodes with at least one structure evaluated at DFT level, and + refers to rod-shaped metal nodes.

[b]We assumed the metal oxidation state by manual inspection of the metal node and consultation with oximachine[S3] and the original article from which the node was taken.

1.2 Linkers

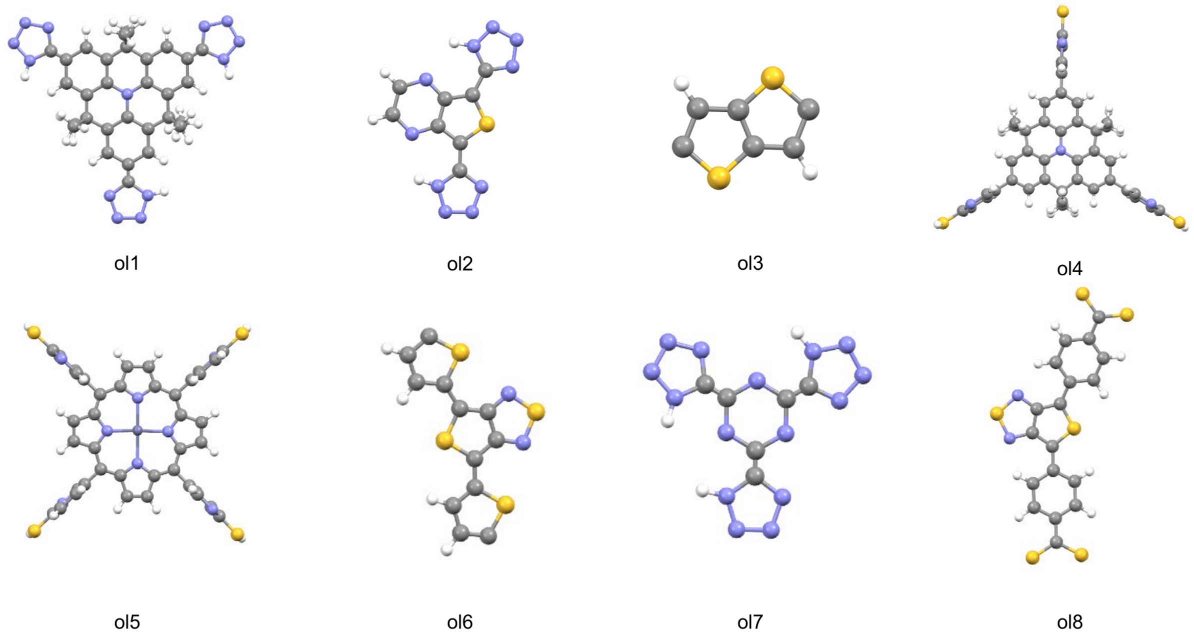

Figure S8: Structures of organic linkers used in this work. Unsaturated atoms in any linker molecule denote their connection points with other metal nodes and linkers (unless specified otherwise).

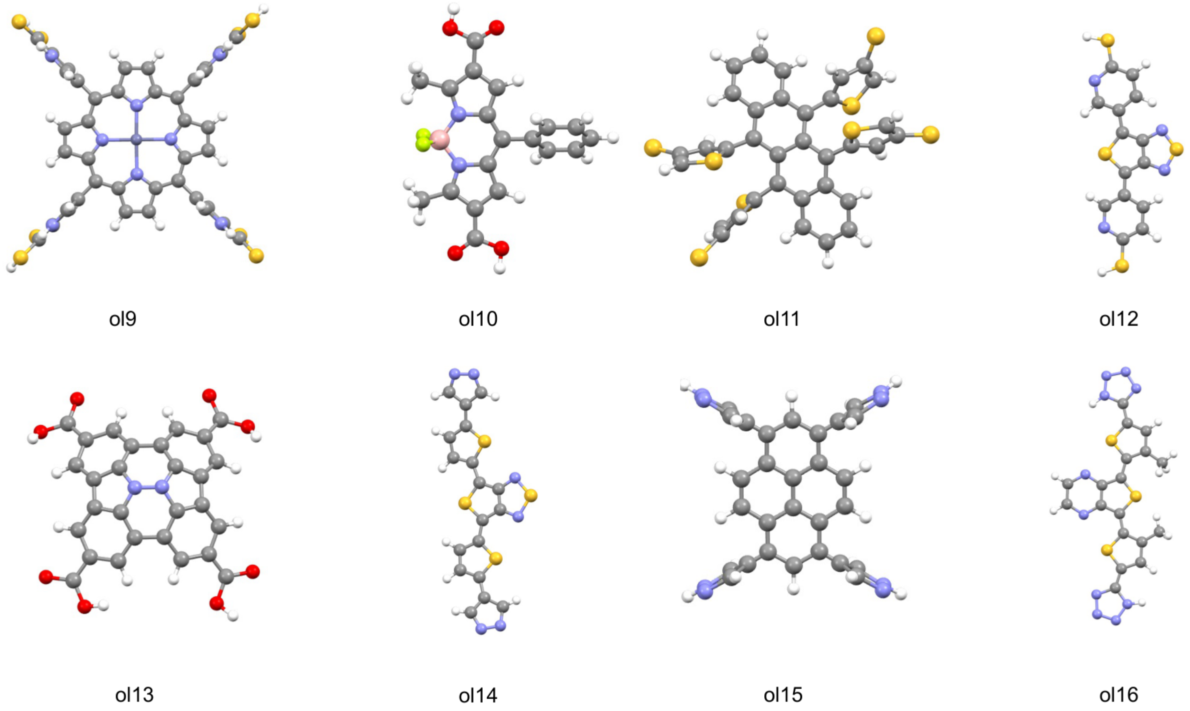

Figure S9: Structures of organic linkers used in this work (contd.).

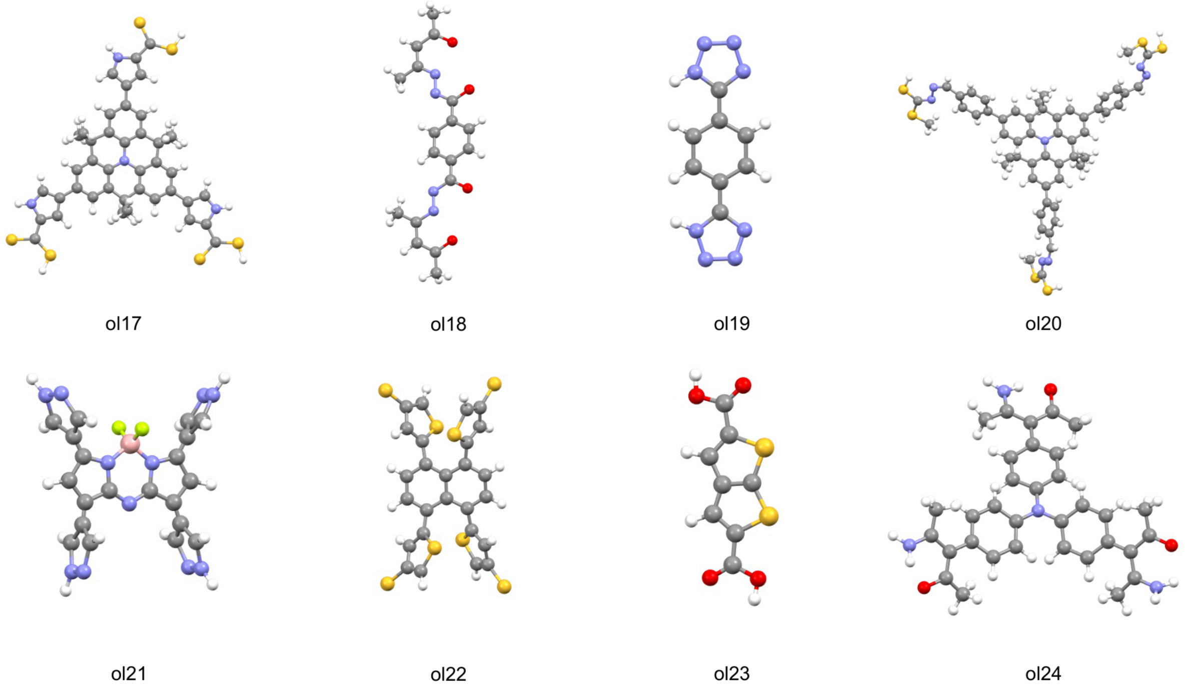

Figure S10: Structures of organic linkers used in this work (contd.).

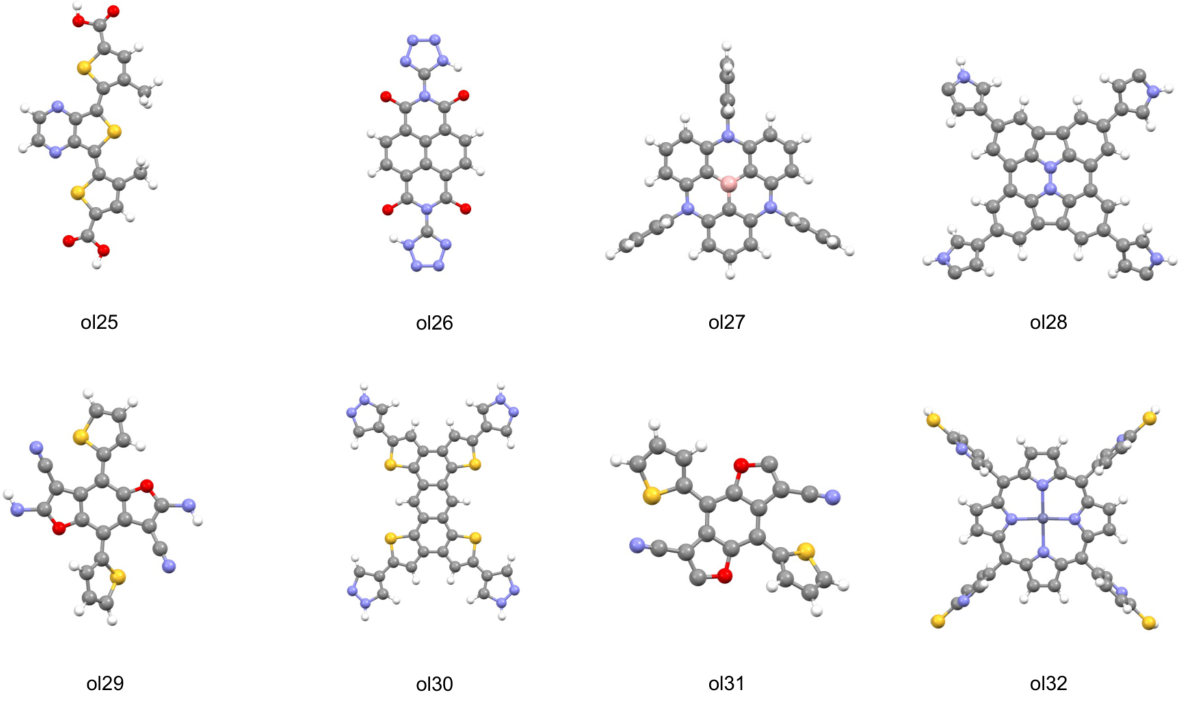

Figure S11: Structures of organic linkers used in this work (contd.).

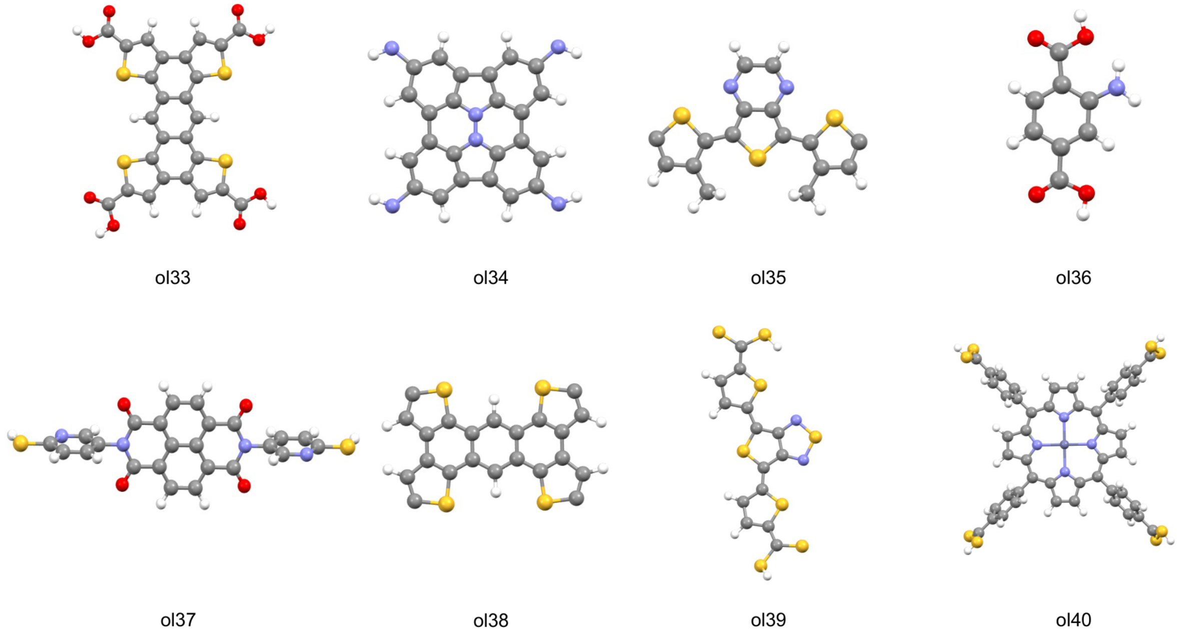

Figure S12: Structures of organic linkers used in this work (contd.).

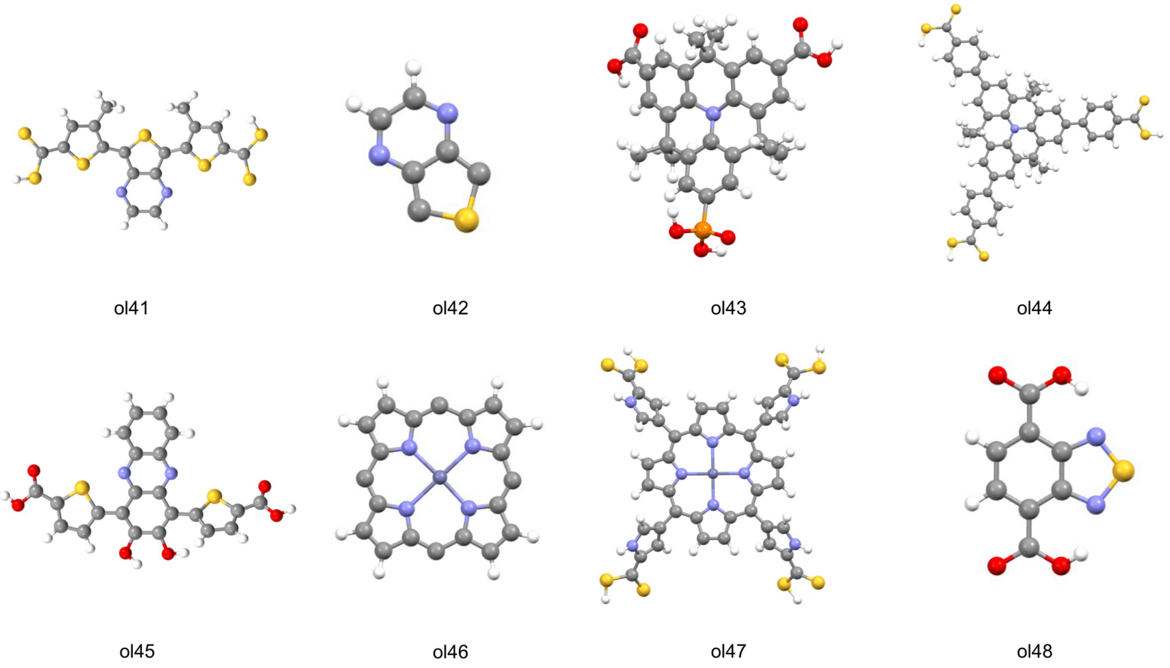

Figure S13: Structures of organic linkers used in this work (contd.).

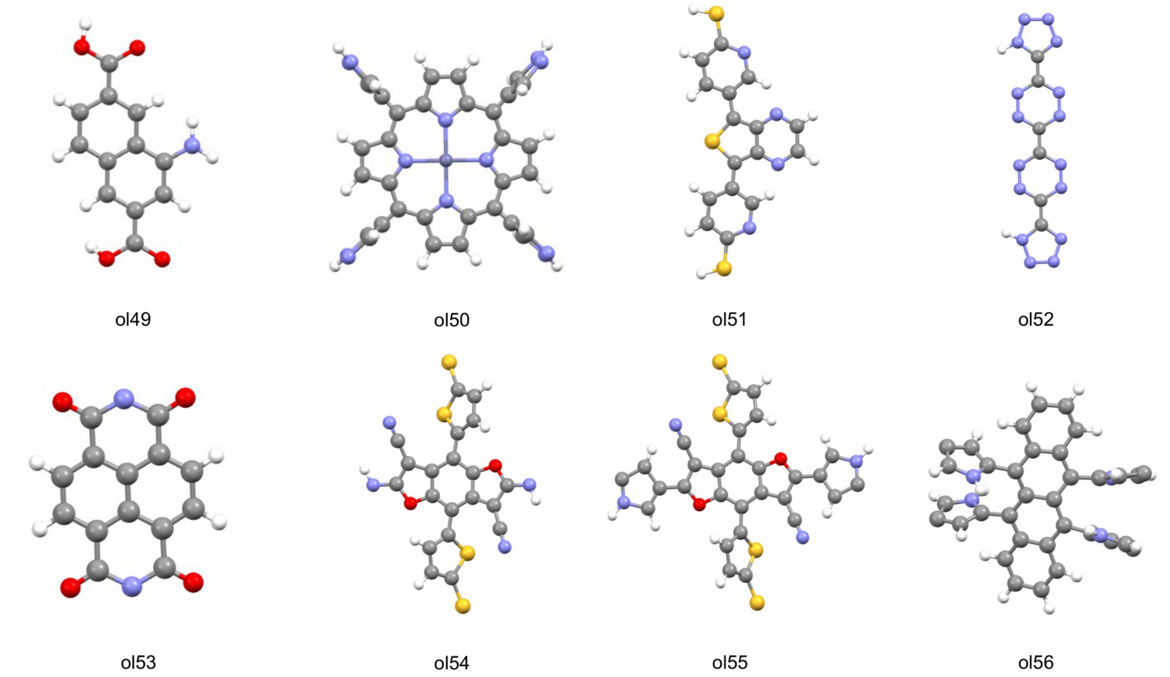

Figure S14: Structures of organic linkers used in this work (contd.).

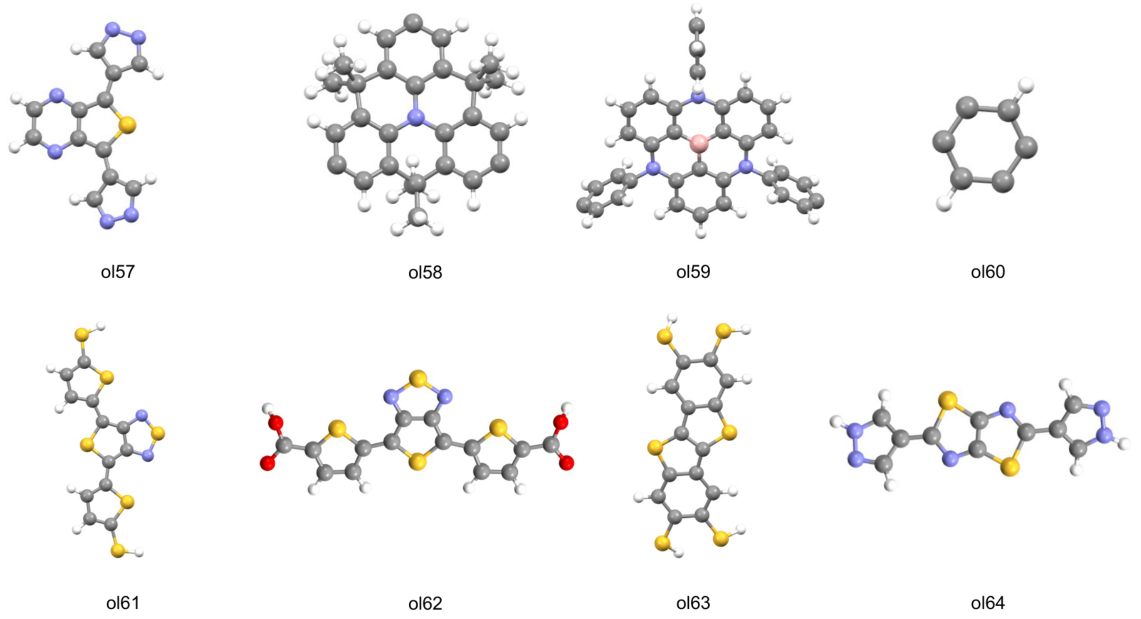

Figure S15: Structures of organic linkers used in this work (contd.).

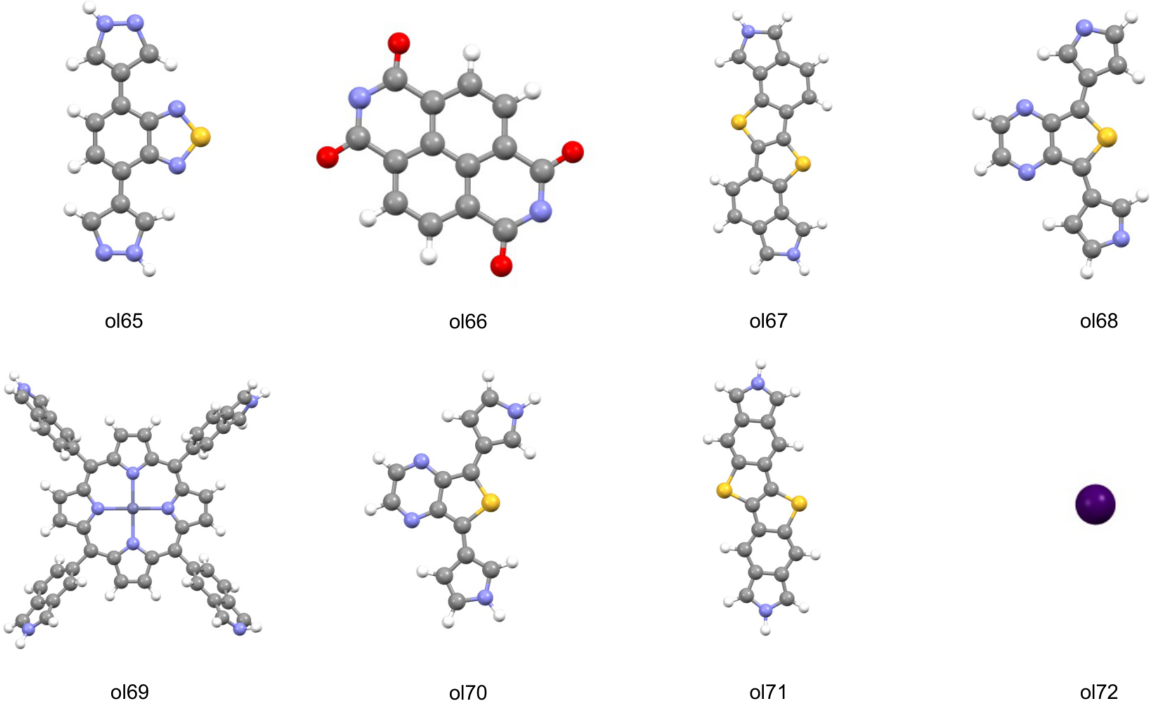

Figure S16: Structures of organic linkers used in this work (contd.). ol72 represents the node-to-node edge in ToBaCCo, which is basically a dummy atom connecting two nodes to each other.

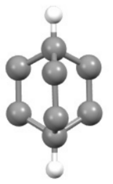

ol73

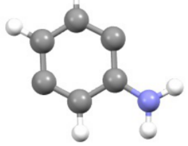

ol79

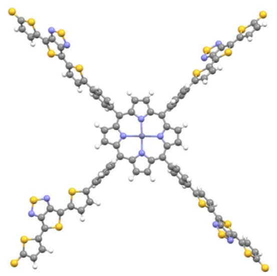

ol81

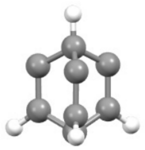

ol82

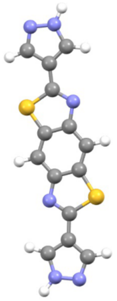

ol83

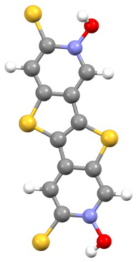

ol84

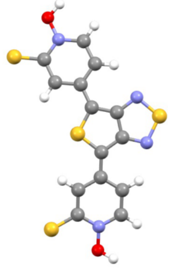

ol85

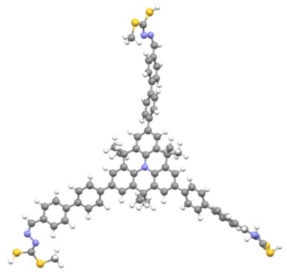

ol86

Figure S17: Structures of organic linkers used in this work (contd.).ol73 represents a bicyclooctane molecule in ToBaCCo with 6 connection points.

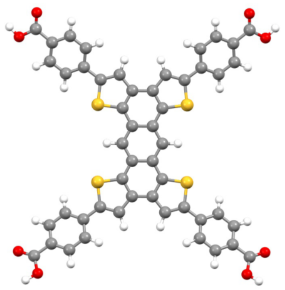

ol87

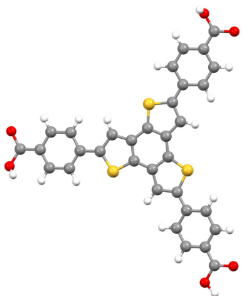

ol88

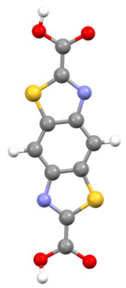

ol89

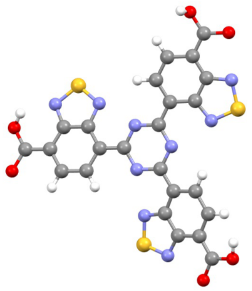

ol90

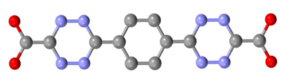

ol91

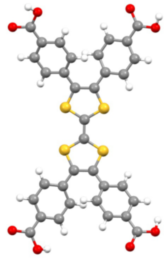

ol92

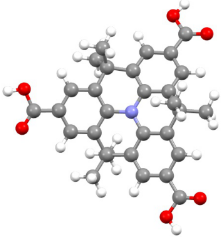

ol93

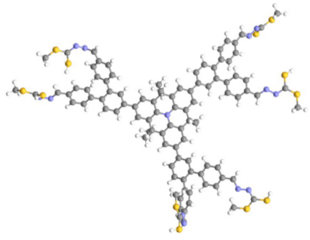

ol94

Figure S18: Structures of organic linkers used in this work (contd.).

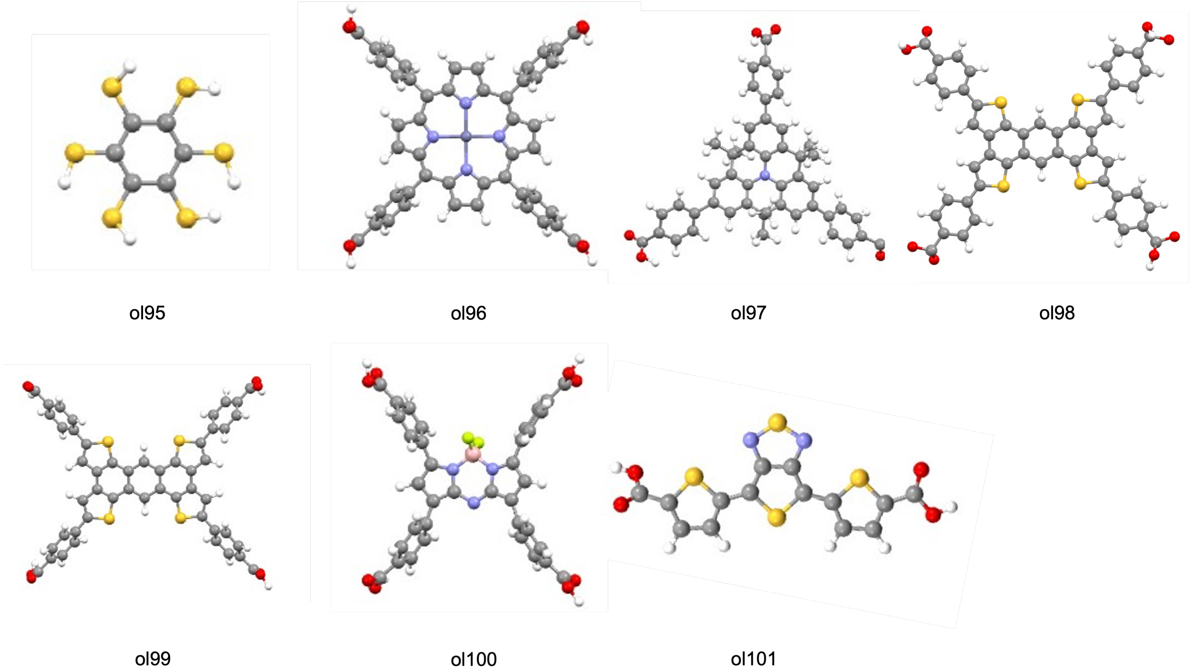

Figure S19: Structures of organic linkers used in this work (contd.).

Table S2: IUPAC names of promising organic linkers

Linker	Name
ol3	hexahydrothieno[3,2-b]thiophene-2,5-DC
ol23	hexahydrothieno[2,3-b]thiophene-2,5-DC
ol31	4,8-di(thiophen-2-yl)-3,7-dihydropyrrolo[2,3-f]indole-3,7-dicarbonitrile
ol64	2,5-bis(4,5-dihydro-1H-pyrazol-4-yl)-2,5-dihydrothiazolo[5,4-d]thiazole
ol36	2-aminoterephthalic acid
ol48	benzo[c][1,2,5]thiadiazole-4,7-DC
ol45	5,5'-(2,3-dihydroxyphenazine-1,4-diyl)bis(thiophene-2-carboxylate)
ol87	4,4',4",4"'-(anthra[1,2-b:4,3-b':5,6-b":8,7-b"']TTP-2,5,9,12-tetrayl)TBA
ol15	4,4',4",4"'-(1,8-dihydropyrene-1,3,6,8-tetrayl)tetrakis(1H-pyrazole)
ol38	2,5,9,12-tetra(1H-pyrazol-4-yl)anthra[1,2-b:4,3-b':5,6-b":8,7-b"']TTP
ol21	5-fluoro-1,3,7,9-tetra(1H-pyrazol-4-yl)-3H,5H-DP[1,2-c:2',1'-f][1,3,5,2]TAB
ol50	(1Z,4Z,6E,10Z,14Z,16E)-5,10,15,20-tetra(1H-pyrazol-4-yl)-Zn-porphyrin

DC = dicarboxylic acid/dicarboxylate; TTP = tetrathiophene; TBA = tetrabenzoic acid; DP = dipyrrolo; TAB = triazaborinine.

Some linkers like ol3, ol23, ol57, and ol38 have been used in our work with multiple metal nodes, thereby having more than one type of linker-cluster bond. For example: ol3 with mn18 forms a carboxylate connection whereas with mn30 it forms a pyrazolate connection.

1.3 Topologies

Table S3: Topologies used in this study

Topology Name	Topology Name	Topology Name	Topology Name
acs-g	bnn	bto	cdt
eta	etc	fes	ffc
hcb	hxl	lig	mco
mog	msw	pcu	pcu-h
pts-x	rtl	smt	sne
srs	twt	utk	utp
bbk	ftw	bcm	bbg
npo	vcs	vct	unj
qtz	ghw	uog	fcu
skb	wkd	vbk	pts-a
bbk	ttg	dmp	unc
uni	lon	fee	mot
bcm	unh	kgd	lig
pto	nou	wix	uoo
mco	fsy	she	ths
etd	bcu-f	hxl	ofo
qtz-h	mot	krg	sxc
krf	kag	gee	fjh

1.4 Structure generation

The *in silico* MOFs in this work were generated using the ToBaCCo algorithm[S2] using the above mentioned building blocks and topologies. For some metal nodes with very high coordination numbers, like mn21 (coordination number 16), we designed the new MOFs by keeping the original topology (of the MOF from where the metal node was extracted), and manually replacing the original organic linker with the organic linkers we used in this work. To generate the structures of rod-like MOFs with 1D metal nodes, we first identified the smallest repeating unit in the 1D metal node chain. Then these repeating units were regarded as the metal nodes like we mentioned above to generate the usual 3D MOF structures. From these generated structures, we selected the ones that represent a rod-like behavior. Eventually, we modified these topology files to make sure that the connection point between the smallest repeating units along the 1D chain is the dummy atom in ToBaCCo. This ensures that the metal node keeps connecting to itself, thus forming a rod-like MOF structure. For the much more complicated 1D metal node chains like some Ti-based clusters (mn22, mn23), we followed the same strategy as mentioned above for the metal node mn21.

Figure S20 shows an example of the naming convention used for the structures in our database. The first three (sometimes four) letters represent the topology of the structure (**pcu** in this example), metal nodes are represented by mn (node number) and organic linkers are represented by ol (linker number). 'v1' stands for vertex 1, referring to the assigned vertex of the corresponding topology graph, which in this example is the metal node mn13. '1' stands for edge 1, referring to the assigned edge of the corresponding topology graph, which in this example is the organic linker ol12. Similarly 'v2' in a structure indicates vertex 2 and '2' indicates edge 2. In the figure describing the **pcu** topology (as obtained from the the Reticular Chemistry Structure Resource Database (RCSR)[S4]), the red atoms represent the vertices and each of the vertices are connected by an edge (black lines). For more details regarding the concept of edges and vertices in a topology graph, we suggest reading the work of Colon et al.[S2]

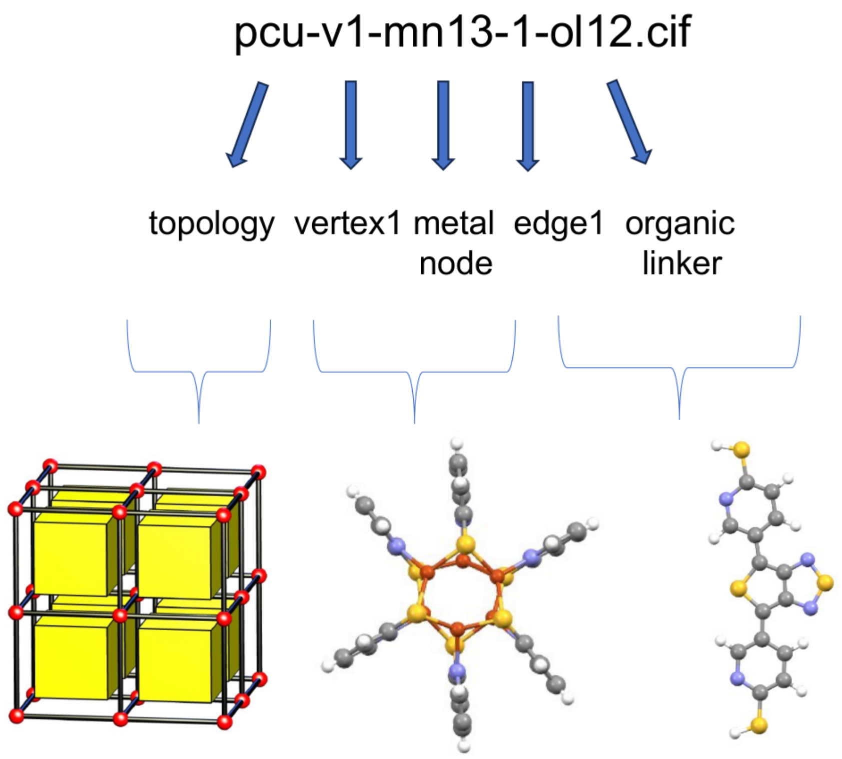

Figure S20: Illustration of the naming convention used for CDP–MOF

To generate structures containing more than one type of metal node (such as 'utp-v1-mn10-v2-mn9-3-ol29'), we modified the respective topology files to make sure that a vertex of type 'v1' can connect with another vertex of type 'v2'. And finally, for structures built manually by replacing the linkers of the original structure, such as in structures 'mn21-ol10' and 'ol8-mn22', no external topology file has been used and thus there is no topology string in the final MOF name.

After being generated, the *in silico* structures were optimized with the UFF forcefield using LAMMPS,[S5,S6] and the corresponding input/data files were created with lammps_interface.[S7,S8] In some cases, the geometry prior to forcefield optimization was preferred as the initial point for DFT calculations. Additionally, for the forcefield optimized structures, partial charges were generated using the EQEq method.[S9,S10] The forcefield optimized structures were checked using an in-house code and flagged in case the optimization did not lead to a structure with reasonable geometry. These structures (20% of our database) were then further optimized

using a machine learning based interatomic potential (MLIP) MACE-mp0[S11] as implemented in.[S12]

1.5 Diversity analysis

The MOF design space integrates experimental and *in silico* databases. Experimental structures primarily originate from the CoRE-2019 database,[S13] while *in silico* structures are drawn from sources such as ToBaCCo,[S14] Anderson *et al.*,[S15] Majumdar *et al.*,[S16] a subset of the PORMAKE database as curated by Burner *et al.*,[S17] and a diverse subset of approximately 20,000 structures from Boyd and Woo.[S18,S19] These databases were used to assess various features like pore geometry (computed with Zeo++[S20]), metal chemistry, linker chemistry, and functional groups, computed using revised autocorrelations (RACs) with a depth of 3 to enhance the description of linker-metal node bonding.[S21]

We utilized three metrics to quantify diversity: variety, balance, and disparity. We divided the high-dimensional feature spaces into 1000 unique bins using the k-means clustering method. Variety is the percentage of bins sampled by a particular database. Balance measures the uniform distribution of structures across the bins, computed using Pieolu's evenness.[S22] For disparity, we calculated the covered area of the concave hull in the map of the first two principal components by a database, which was then normalized against the total area covered by all databases combined. Further details on featurization techniques and diversity analysis can be found in our previous works.[S16,S19]

To create a diverse subset of MOFs from the QMOF database, we applied the farthest point sampling method[S23] (also known as max/min sampling), following Burner *et al.*'s approach.[S17] This selection was based on the combined features of metal chemistry and linker chemistry, which are crucial for our specific application.

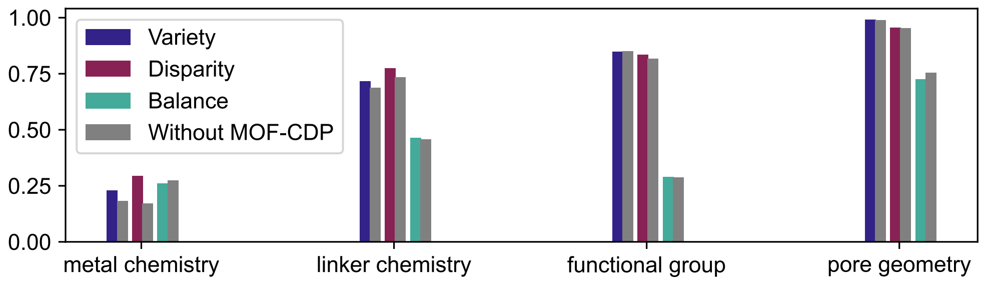

Figure S21: Diversity metrics for *in silico* MOF databases before and after adding our database – CDP–MOF. Notably CDP–MOF database improves the overall diversity of the subspace of metal chemistry and linker chemistry, arguably the two most important MOF subspaces in the overall MOF chemical space for finding promising photocatalytic structures. Pore geometry and functional group features, being already explored extensively in the MOF databases (for gas adsorption applications) do not show any improvement upon adding CDP–MOF structures.

1.6 Experimental MOFs

The chosen subset of experimental MOFs is taken from a previous study,[S24] and comprises the following MOFs: MOF5, NTU-9, MOT-74(Zn), ZSTU2, NNU-36, UiO-66, AlPMOF, MOF-Zn1, MUV-11, UiO-66(NH_2), Zn2(ttfb)m MIL-125, MOF-5(NH_2), ZSTU1, MIL-125(NH_2).

2 Photocatalysis assessment

2.1 Evaluation of photocatalytic descriptors

The Automated Interactive Infrastructure and Database for Computational Science (AiiDA,[S25,S26] v1.6.5) was used as a workflow manager to perform the DFT calculations in CP2K (v.9.1,[S27]) for 143 *in silico* MOFs. For the closed shell MOFs, we utilized a workflow developed previously to evaluate DFT-based photocatalytic descriptors for COFs.[S28] The workflow evaluates the band gap to ensure a closed-shell system/semiconducting band gap, relaxes the structure, and calculates the four DFT descriptors for photocatalysis.[S24,S29] In this work, we go further and compute information on the likelihood of charge transfer between the linker and metal node. This is done by fragmenting the relaxed MOFs into their building blocks with `moffragmentor`,[S30] and integrating separately the cube files for electron and hole injection for the linker and metal node.

A new workflow for open shell systems was built, as depicted by the flowchart in Figure S22 with similar logic to the closed shell workflows. The logic of the open shell workflow consists of employing DFT+Hubbard method to test for different spin configurations, relax the structure, and perform band structure calculations (the latter computed in Quantum ESPRESSO v.7.0[S31]) to compute the charge carrier mobility descriptor. The choice of Hubbard U values (Table S4) is discussed below. For the remaining descriptors (visible light absorption, thermodynamic feasibility, charge recombination, and the likelihood of charge transfer), a hybrid (PBE0, in CP2K) functional is used for open shell MOFs.

2.1.1 A comment on KS band gaps

PBE Kohn Sham gaps can display fortuitous agreement with experimental optical gaps for MOFs, where exciton binding energies and electron-phonon coupling can be quite strong. For example, the PBE gap of MOF-5 reported by Kshirsagar et al.[S32] (3.57) agrees reasonably with the experimental optical gap (≈ 3.8-4.1) for the specific studied case of MOF5. However,

in general, the experimental gap values are somewhere between PBE and PBE0 values (see Table S2 of Fumanal et al.[S24]).

While PBE would seem at first like the better alternative to assess experimental optical gaps due to the low cost of GGA functionals, its well-known overdelocalization errors can have a huge impact in the evaluated photocatalytic properties.

For example, PBE might fail to correctly capture the character of the conduction and valence band edges, specially if it involves localized electronic states. The incorporation of exact exchange (such as in PBE0) can mitigate this failure.[S33]

Moreover, the possibility of having charge transfer states (such as LMCT) and different degrees of delocalization might also make it more difficult to generalize the trend that PBE is closer to the optical gap.

The choice of PBE0 as a reference for band gaps is also motivated by the following points: PBE0 is also closer to the fundamental gap than PBE, and could therefore offer a better picture when looking at the band alignment (IP and EA) when compared to PBE.[S29] The charge separation descriptor computed by the overlap between charged doublets at PBE0 level is a good approximation to TDDFT@PBE0 results.[S24] Therefore, choosing PBE0 as a reference is another way of ensuring consistency among the descriptors. In this case, the only bottleneck is the effective mass, which is computed at a lower level of theory.

We highlight that the goal of this work was not to compute optical gaps accurately, but instead to use a strategy that allows us to easily (and cost-effectively) filter potential candidates for photocatalysis. If we have a short list of potential photocatalysts, more accurate studies can be done to assess their particularities and confirm their usefulness. We leave that for future work.

2.2 DFT calculations

DFT calculations were performed with the QuickStep code of CP2K 9.1.[S27] The Perdew-Burke-Ernzehof (PBE) exchange-correlation functional was employed with DFT-D3(BJ) van

der Waals correction. Plane-wave and relative energy cutoffs of 600 Ry and 60 Ry, respectively, were chosen with a 4-level multigrid mapping. The orbital transformation (OT) method was used. Calculations were performed at Γ-point over the irreducible Brillouin zone due to the large cell parameters of the MOFs. When necessary, supercells were built to ensure all cell parameters have a distance above 10 Å, and guarantee proper sampling.

Goedecker-Teter-Hutter (GTH) pseudopotentials and mixed Gaussian and plane-waves basis sets were used, with double-ζ and triple-ζ polarization MOLOPT basis sets for describing non-metals and metals, respectively. For PBE0 calculations, MOLOPT-ADMM auxiliary basis functions were employed along with the auxiliary density matrix method (ADMM pFIT for non-metals and cFIT for metals)[S34] to reduce computational cost.

At all stages of optimization, MOFs with band gaps below 0.5 eV were excluded to compensate for errors from PBE.

For band structure calculations, the choice of k-points mesh was mostly at Γ-point, but when needed, a Monkhorst-Pack scheme was chosen to guarantee proper sampling of structures with lattice parameters smaller than 10Å. The reciprocal space path within the first Brillouin zone (BZ) was computed by connecting the high-symmetry points with the Seek-path package.[S35] As default, we chose to sample 20 k-points along the line between two high-symmetry points.

2.3 Open shell workflow

The open shell workflow utilizes base calculations from the plugin aiida-cp2k and a slightly modified multistage optimization workflow from the plugin aiida-lsmo to accommodate changes in the KINDS section, such as informing a guess spin configuration, auxiliary basis sets for PBE0 calculations and the U parameter for DFT+Hubbard. The logic of the open shell workflow consists of employing the DFT+Hubbard method to test for different spin configurations, relax the structure, and perform band structure calculation (the latter in quantum espresso) to get the charge carrier mobility descriptor.

Throughout the work, we computed effective masses at the GGA level. This means that when calculating the band structure for open shell systems, we initially relied on PBE. However, the widely-known overdelocalization problem in DFT can lead to extremely small or nonexistent band gaps in open shell systems,[S33] which prevents us from correctly computing the effective masses of VB and CB. This happens due to localized partially occupied d orbitals, for which self-interaction errors are particularly pronounced.[S36,S37] Indeed, Rosen et al.[S33] shows in Fig. 2 of their work that "the use of GGA functionals like PBE may lead to incorrect qualitative comparisons between the band gaps of different materials if some have closed-shell character and others have open-shell character." For this reason, we chose DFT+U to address such overdelocalization. The employment of non-local Kohn-Sham potentials in the DFT+Hubbard approach is said to tackle issues such as the overdelocalization of electrons that is associated with a derivative discontinuity of the exchange-correlation functional.[S38–S41]

For the remaining descriptors (involving band gap, band edge alignment and spatial overlap) a hybrid (PBE0) functional is used. While DFT+U is often enough to get a semiconducting band gap for these systems, we chose PBE0 instead to be consistent with the closed shell systems, where PBE0 was the reference. Given that DFT+U affects only localized d and f orbitals and depends on the U value, a clear empirical correlation between DFT+U and PBE0 (the reference for closed-shell systems) values across diverse open-shell MOFs is not to be expected.

Firstly, we check if the electronic configuration for each cluster is already established in the literature based on the CSD reference code. If this is not the case, all possible spin configurations deriving from different crystal field splittings are tested in single-point energy calculations to find the most stable configuration with a GGA-based DFT+Hubbard approach.

Hubbard U values are chosen based on reports from the literature,[S42,S43] see Table S4. While Hubbard U values are structure-dependent and a better approach would be to compute those values from first principles, due to the screening nature of the work, we chose values from literature to mitigate computational costs. Then, structure relaxation is performed at the same level of theory. The photocatalytic descriptors are then computed.

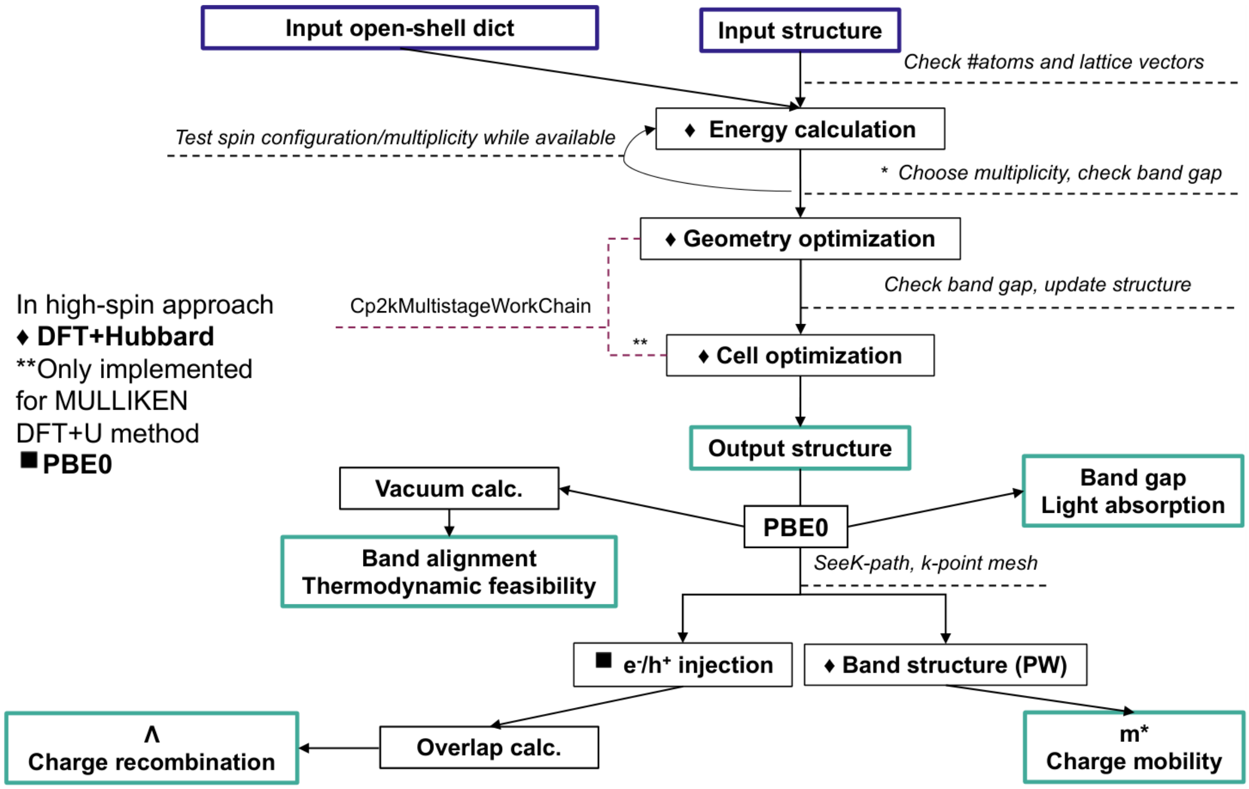

Figure S22: Workflow designed to compute the photocatalytic descriptors for open shell MOFs. Structures are tested for different spin configurations and optimized with PBE+U. Energy-based descriptors and charge recombination descriptors are obtained at PBE0 level. Charge carrier effective masses are obtained from the band structure computed at PBE+U with Quantum ESPRESSO.[S31]

Table S4: Hubbard U values[S42,S43] used for DFT+U calculations in CP2K (value in parenthesis corresponds to the value used for the cell optimization, where often a smaller U is needed) and QUANTUMESPRESSO (QE)

**Metal**	**Ox. state**	**U (CP2K)**	**U (QE)**
Ni	III	4.5 (3)	6.4
Mo	V	2	6
Co	II	4 (3.3)	4
Co	III	4 (3.3)	4
Cu	II	4	4
Fe	II	4.5 (2)	5
Fe	III	4.5 (2)	5
Cr	II	4.5 (1.8)	4
Cr	III	4.5 (1.8)	4
V	III	4 (3.5)	4
V	IV	4 (3.5)	4
Mn	II	4	4
Mn	III	4	4

2.4 Further DFT details on photocatalytic descriptors

For closed-shell MOFs, the photocatalytic descriptors are computed as described in literature.[S24,S28,S29] For energy-based and charge recombination descriptors, we performed a test for one MOF per closed-shell metal nodes with PBE0 calculations to ensure that the correlation between PBE and PBE0 values from literature holds.[S29]

The adaptation for the cases of open shell MOFs is as follows. To compute the charge separation and energy-based descriptors, DFT calculations with PBE0 hybrid functional were performed for more reliable results. To mitigate the computational cost and due to limitations in software implementation, the computation of effective masses is based on band structure calculation kept at the DFT+Hubbard level. Moreover, we chose to only evaluate open shell structures with less than 500 atoms due to higher computational cost associated with computing descriptors at PBE0 level of theory and treating open shell with the required unrestricted Kohn-Sham formalism.

Electronic chemical potentials were aligned to vacuum as previously reported by,[S29] based

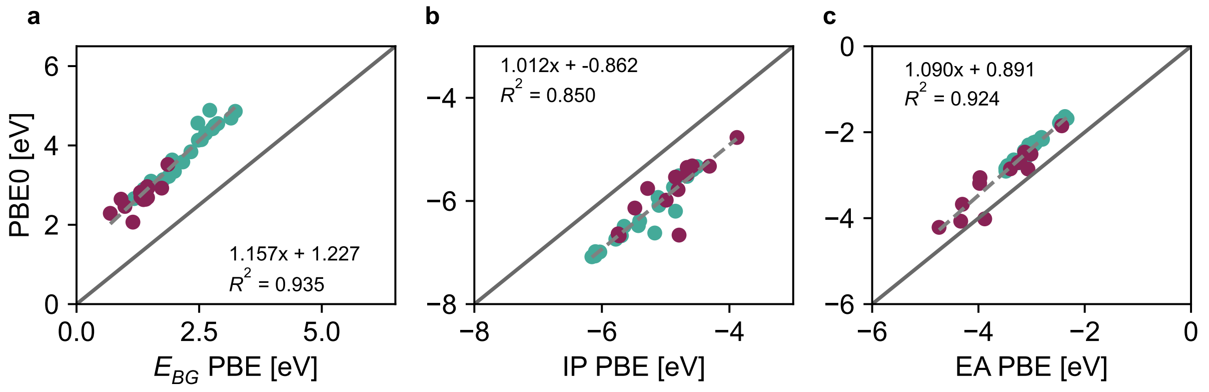

Figure S23: Correlation between PBE-based and PBE0-based DFT values for a) band gap, b) ionization potential (IP), and c) electron affinity (EA) with values from literature in green[S29] and values from a CDP–MOF subset in maroon. The subset contains 13 MOFs, i.e., one MOF per metal node for all the evaluated closed-shell metal nodes.

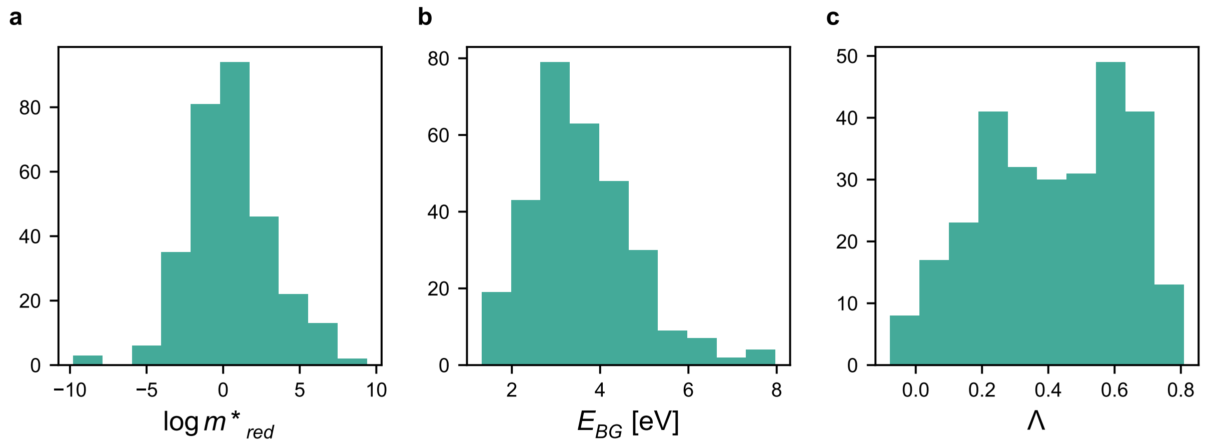

Figure S24: Distribution of computed a) reduced effective, b) PBE0-adjusted band gaps, c) charge recombination descriptor (Λ).

on the procedure established by[S44] For closed-shell MOFs, the band extrema energies were corrected with our empirical adjustment to mimic PBE0 values.[S28,S29] This was not necessary for open shell MOFs, for which calculations were already performed at PBE0 level.

Ground-state unrestricted Kohn-Sham (UKS) DFT calculations were employed for electron and hole injection to evaluate the charge separation descriptor as previously defined.[S24,S28]

For the charge separation descriptor, the `cube` python package (Section 5) was used to

compute the averaged spatial overlap of the cube files for electron and hole injection.[S45] To compute the likelihood of linker-to-metal node charge transfer, the relaxed MOF structures were fragmented into their building blocks with `moffragmentor`,[S30] and the cube files for electron and hole injection were integrated separately for linker and metal node.

The parabolic approximation implemented in sumo library with 8 fitting points around the band extrema was used to compute effective masses of electrons and holes based on the electronic bands' dispersion in the reciprocal space.[S24,S46,S47]

The distribution of band gap, m^*_{red} and Λ is shown on Figure S24.

2.5 Experimental information on promising building blocks

Table S5: Experimental information available for the isolated clusters and/or clusters already used in MOFs. The oxidation state is as reported or predicted by oximachine.[S3] T_s is the temperature of synthesis. The stability section collects information on different reports on the stability of the cluster or MOF. Information on whether single crystals were obtained for the cluster and/or MOF is also displayed

**Cluster**	**Ox. state**	T_s	**Stability**	**Single crystal?**
mn8[S48]	Au(III)	255 K[S49]	Ambient[S49]	Yes[S48,S49]
mn13[S50]	Cu(I)	413.15 K	511.15 K/photostable	Yes[S50]
mn20◇[S51]	Ti(IV)	398.15 K	473.15 K	-
mn21◇[S52]	Ti(IV)/Zr(IV)	373.15 K	Chemical (pH)	Yes[S52]
mn23◇[S53]	Ti(IV)	423.15 K	623.15 K/recyclable	Yes[S53]
mn24◇[S54]	Ni(II)	353.15 K	Chemical/thermal	-
mn29◇[S55]	Fe(II)	433.15 K	-	Yes[S55]
mn33[S56]	V(IV)	-	Air-stable[S57]	-
mn39[S58]	Mn(III)/(IV)	T_{room}	[S59,S60]	Yes[S61,S62]

◇ already used in MOFs.

Table S6: Overview of the organic linkers utilized by this work. The pKa of the protonated linker is obtained through an XGBoost machine-learning method with water as solvent.[S63] The pKa in other solvents can be obtained by inputing the SMILES into the web application.[S64]

**Organic linker**	**Linker-cluster bond**	**pKa**	**Similar usage in MOFs**
ol3	carboxylate	3.4	[S65]
ol31	pyrazole	7.9	-
ol45	carboxylate	1.7	-
ol48	carboxylate	2.5	[S66]
ol57	pyrazole	7.0	[S67]
ol80	dithiolate	6.4	[S68]
ol39	carbodithioate	5.9	[S69]
ol84	pyrithione	7.5	-
ol51	pyridine-2-thiolate	6.4	-

◇ We utilized some linkers like ol3, ol31, and ol57 allowing for more than one type of linker-cluster bond. By similar usage in MOFs, we refer to organic linkers that have similar linker-cluster bond (for e.g. dithiolate) and similar functional groups in the linker.

2.6 Comparison with experimental and QMOF subsets

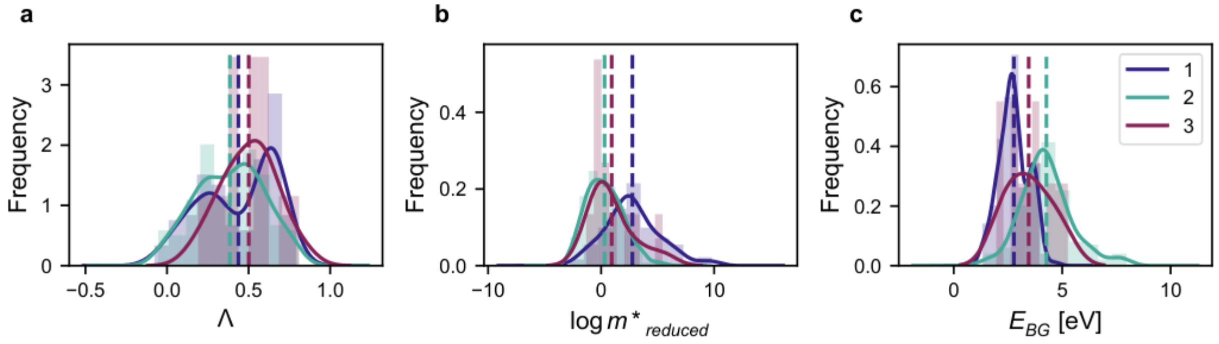

Figure S25: Distribution and kernel density estimators of a) charge recombination descriptor, b) reduced effective mass, c) PBE0-adjusted (closed shell) and PBE0 (open shell) band gaps for CDP–MOF (1, in indigo blue, 170 structures), a diverse QMOF subset (2, in jungle green, 150 structures), and a subset of reported experimental photocatalytically active MOFs (3, in maroon, 15 structures). Dashed vertical lines indicate the mean for each set of structures.

In the QMOF subset, 3 out of 154 structures (1.9%) met the criteria for all the descriptors. 12 out of 154 structures (7.8%) met the criteria for HER, m^*_{red}, Λ, VIS. Only 15.6% of the structures had band gap in the visible range after empirical correction to PBE0 values.

3 Supervised machine learning

Two models were fine-tuned and compared. GPT-J[S70] and MOFTransformer.[S71] They were used to predict the photocatalytic descriptors in the whole of CDP–MOF and QMOF databases. Among the DFT-unevaluated CDP–MOFs, 85.6% contain metal nodes that were previously evaluated at DFT level, and 66.9% were structures with more than 500 atoms in the unit cell. Therefore, most of the unevaluated CDP–MOFs were merely not evaluated due to resource limitation, which further justifies the use of fine-tuned machine learning models.

The binary classification targets used for both the models were as follows:

- VIS / not VIS: whether the PBE band gap energy (empirically adjusted to PBE0 values) for the MOF is within the visible light spectrum, i.e., between 1.6 eV and 3.2 eV. Although the computed gaps are not optical gaps, PBE0 values tend to be a good approximation to optical gaps in MOFs due to a cancellation of errors.

- HER / not HER: whether the vacuum aligned EA of the MOF is higher than the redox potential for HER, i.e., -4.4 eV.

- OER / not OER: whether the vacuum aligned IP of the MOF is lower than the redox potential for OER, i.e., -5.6 eV.

- OWS / not OWS: whether the vacuum aligned EA/IP of the MOF comprises the redox potential for OWS.

- Λ / not Λ: whether the charge separation descriptor of the MOF is above 0.5.

- $m^*_{reduced}$ / not $m^*_{reduced}$: whether the reduced effective mass of the MOF is below 1.

Each classification target returns a True (1) or False (0).

3.1 GPT-J

Since the evaluation with DFT for all MOFs is computationally not feasible, machine learning was used to predict the photocatalytic properties of the remaining unevaluated structures. As a first methodology, we fine-tuned a Large Language Model. Using natural language allows us to omit field-specific featurization of structures and use textual strings to represent the MOFs. Specific to our dataset, we hereby circumvent computationally expensive calculations of large structures. We thus used the SMILES notation of the node and the linker(s) as a representation of the MOFs. Each property was approached as a binary classification, i.e., does meet the requirement *versus* does not meet the requirement.

We combined the evaluated CDP structures (146), the evaluated QMOF structures (154) and a set of experimentally evaluated structures (14) to create the training set. We used the same methodology as ref.[S70] Here, we fine-tuned an LLM by querying simple prompts (Table S7).

Table S7: **Example prompts and completions for predicting photocatalytic properties.** `<nodes>` and `<linkers>` serves as a placeholder for the SMILES of the node(s) and linker(s), respectively. `<property>` serves as a placeholder for one of the photocatalytic properties.

prompt	completion	experimental
Example of training data		
`What is the <Property> of a MOF with nodes <nodes> and linkers <linkers>?`	0	Low
`What is the <Property> of a MOF with nodes <nodes> and linkers <linkers>?`	1	High

We used the open-source GPT-J 6B parameter model,[S72] which was trained on the Pile, a large-scale curated dataset created by EleutherAI.[S73] Although arguably an outdated model, we proved in a previous large-scale benchmarking study that this model performs well for various tasks[S74] Similar to the original work, we used 8-bit quantization[S75] and 8-bit optimizers[S76] in addition to the Low-Rank Adaptation of Large Language Models (LoRA) technique[S77] (LoRA parameters: r=16, lora_alpha=32, and lora_dropout=0.05) to use the

models on our hardware. All models were trained with 25 epochs and a learning rate of 0.0003. The fine-tuned models (one for every property) predicted the unevaluated MOFs from the CDP dataset and the QMOF database.

For every property, the GPT-J base model was fine-tuned with the DFT-evaluated structures. This fine-tuned model was then used to predict the properties of the remaining CDP-MOF and QMOF structures. The reliability of the models was assessed by splitting the evaluated structures in a train (85%) and test subset (15%), and calculating F1 scores of the test set. This metric indicates the reliability of the predictions on the unevaluated structures. When comparing to MOFTransformer, we see similar trends in F1 scores of the various properties. High values were obtained for the HER and VIS property, whereas lower values were calculated for the $m^*_{reduced}$ property.

3.2 MOFTransformer

For the MOFTransformer, we fine-tuned the base model developed by Kang *et al.*,[S71] using the same training data used in the GPT-J approach to predict the photocatalytic descriptors in a binary classification fashion. It uses an energy-grid embedding (using CH_4) and an atom-based graph embedding to represent a MOF.

The featurization procedure for the QMOFs and CDP–MOFs follows the method outlined by Kang et al.[S71], where both global and local features are extracted, and a Transformer encoder is employed to generate the feature vector used for training. During fine-tuning, MOFTransformer is initialized with the converged weights from the pre-trained model and further trained to predict the desired properties of MOFs. A single dense layer is added to the class token, and all model weights are adjusted to optimize property prediction. 20 % of the data was used as a holdout test set for model evaluation. 16 % of the data was used as a validation set to initiate early stopping. A maximum of 10 epochs was used, with a batch size of 8. See Kang et al.[S71] for more details.

The MOFTransformer was trained on each target seven times using different seeds. A

binary classification was used for each target. Although we did not use the same train/test split as in GPT-J, the training of each target seven times was consistent for both models and can mitigate potential biases.

3.3 Embeddings

Contextual embeddings The MOFTransformer is fine-tuned on a target, and the high-dimensional embeddings are extracted. UMAP is used to reduce the dimensions to two, and we refer to this space as the contextualized feature space.

The following plots highlight the chemical design space per predicted target, with a distinction between QMOFs and CDP–MOFs.

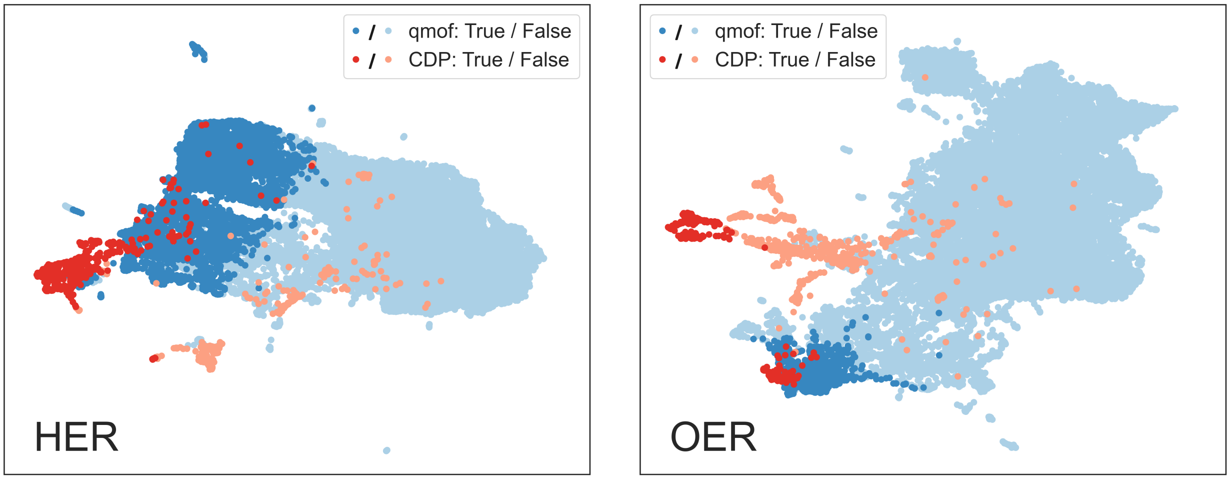

(a) (b)

Figure S26: test

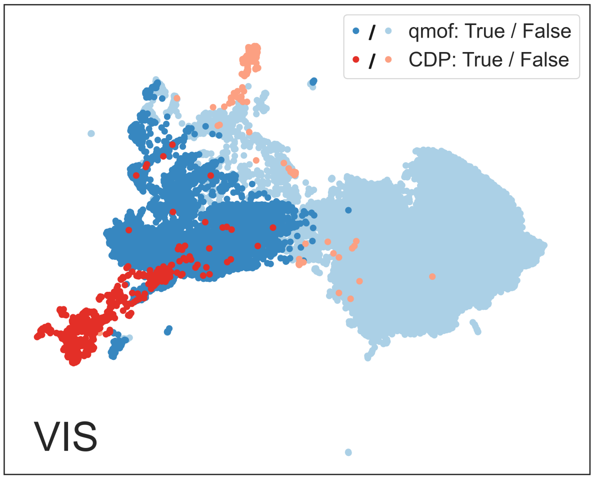

(a)

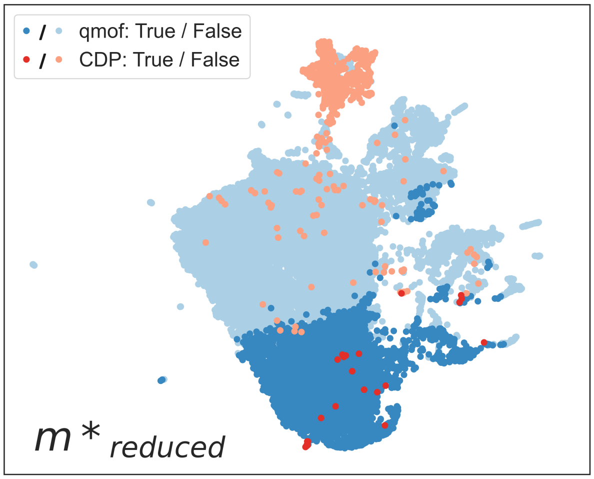

(b)

Figure S27

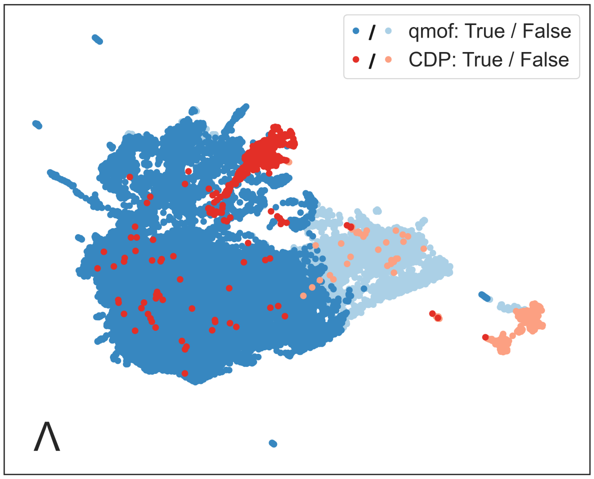

(a)

Figure S28

3.4 Model comparison

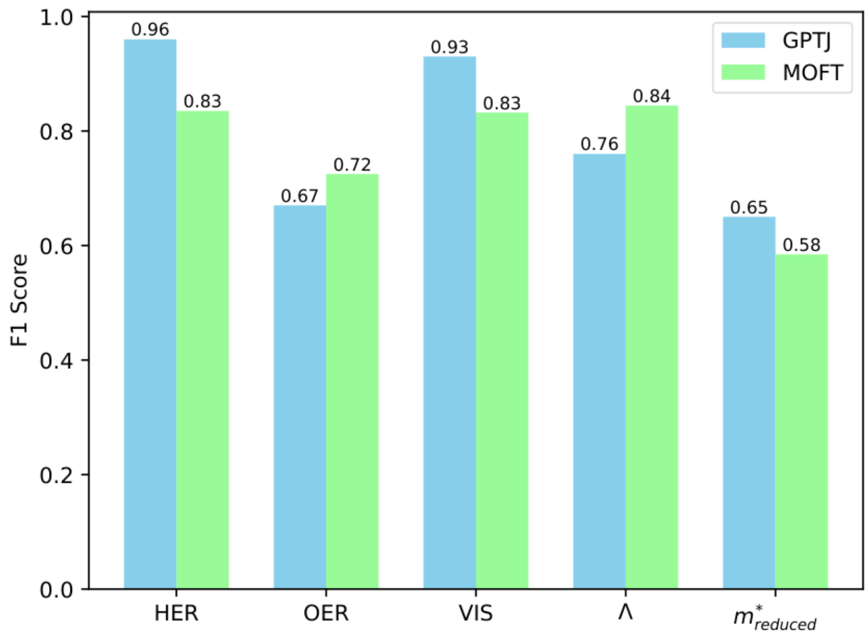

Figure S29: F1 scores of a test subset (15%) of all DFT-evaluated structures (CDP and QMOF) by GPT-J (blue) and MOFTransformer (green).

Figure S30 shows, for both models and for each descriptor, the percentage of true predictions. Here, "true" (or "1") means that the descriptor is predicted to be in the desired range, *e.g.*, band edges align with HER, or reduced $m^*_{reduced}$ is smaller than 1. We see that, for visible light absorption and alignment to HER and OER redox potentials, CDP–MOFs have a much higher percentage of true descriptors for both models, confirming the bias in our database towards photocatalytic favorable structures. The trend of a higher percentage of true descriptors in CDP–MOF is not seen in the predictions for the charge separation (Λ) and charge transport (m^*_{red}) descriptors. Test accuracies for the latter were also considerably lower (Figure S29), thus we could say that predictions are less reliable. Still, at least for Λ, a reasonable set of CDP–MOFs had favorable predictions. Altogether, Figure 4 shows that CDP–MOFs indeed stands out when it comes to meeting multiple criteria simultaneously.

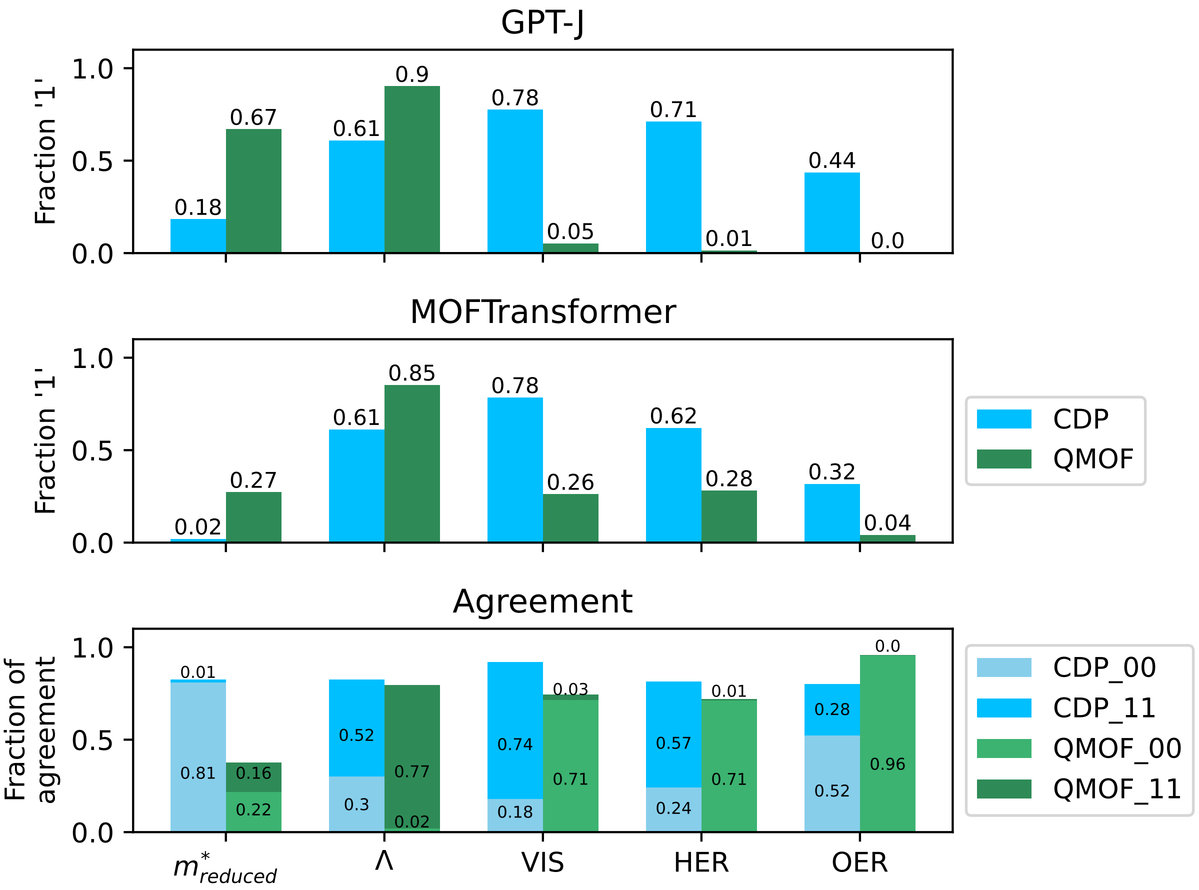

Figure S30: ML predictions of unevaluated structures of CDP-MOF (blue) and QMOF (green) by GPT-J (top) and MOFTransformer (middle). All predictions are based on binary classification of the descriptors, i.e., whether the value is in the desired range ('1') or not ('0'). Both show the percentage of favorable structure for the respective descriptor. The agreement of both models (bottom) where '00' and '11' refers to the fraction where both models predict '0' and '1', respectively.

3.5 DFT evaluation of MOFs predicted to meet all criteria

We evaluated MOFs predicted by either one of the ML models to meet all criteria. We highlight that, due to the combination of errors involved in using 5 different models to predict each criteria, we did expect that most MOFs would not really meet all criteria. Instead, the goal was to help identify a few without the need to perform brute-force screening. Of all the evaluated MOFs, 38% are aligned to HER and 46% are aligned to OER. Figure S32 shows the distribution of the remaining descriptors. Four structures actually meet all criteria, as

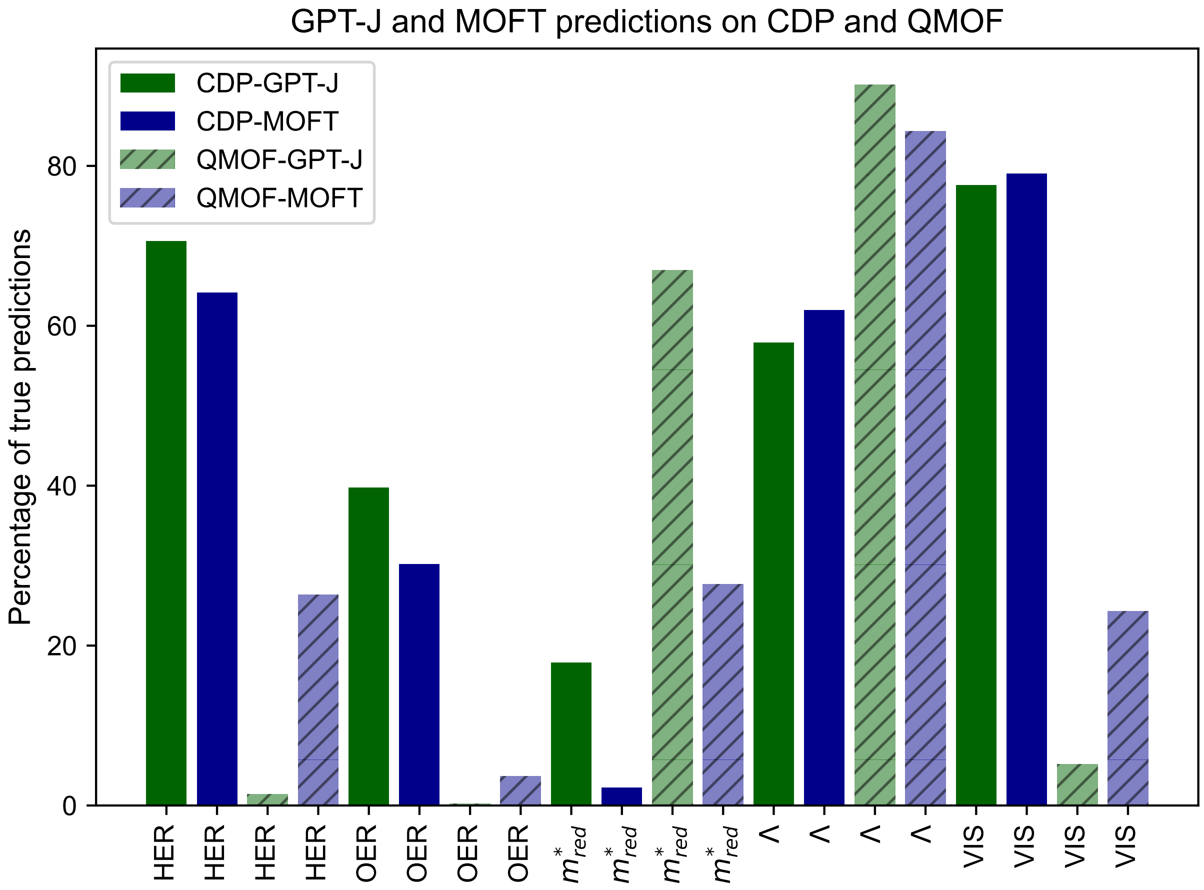

Figure S31: ML predictions of unevaluated structures of CDP-MOF (hatched) and QMOF (solid) by GPT-J (green) and MOFTransformer (blue). CDP–MOF contains ≈ 1k MOFs (752 from MOFTransformer predictions, 1007 from GPT-J predictions) and QMOF contrains ≈ 20k MOFs (20373 from MOFTransformer predictions and 17538 from GPT-J predictions).

discussed in the manuscript.

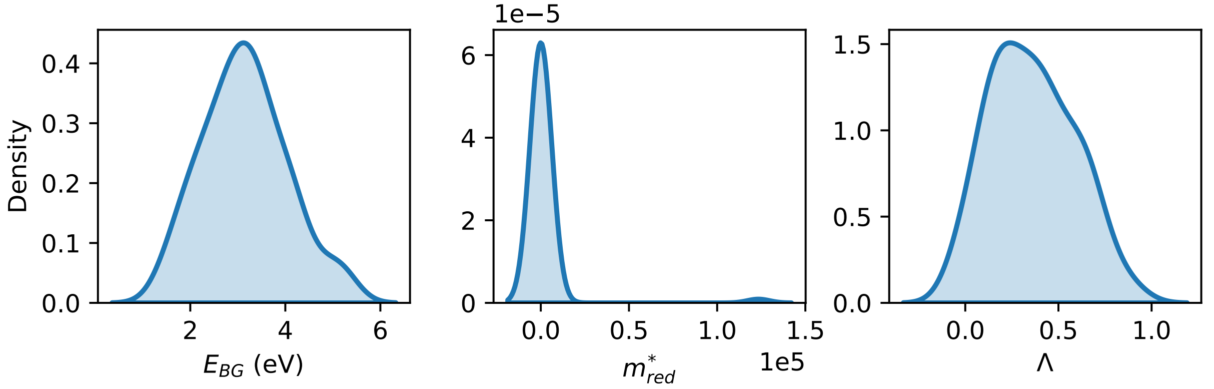

Figure S32: Distribution of DFT computed properties for the selected MOFs predicted to meet all criteria.

4 Structural analysis

4.1 Effect of CDP–MOF building blocks on the photocatalytic descriptors

Figure 2c-e shows a correlation between specific metal nodes and the MOF's thermodynamic ability to drive HER, OER, and overall water splitting (HER+OER) reactions.

Notably, Ti-based nodes like mn20 and mn21 almost unanimously exhibit suitable band edge alignment for either one or both of the processes. We highlight that MOFs with mn21, a highly connected mixed Ti-Zr node, have a lower average band gap when compared to all other MOFs without this node (Figure S47a). This observed reduction adds to the list of interesting features of Ti-based materials, *i.e.*, intriguing optoelectronic properties, low toxicity,[S78] and potential for driving HER[S79] and enhancing photocatalytic stability.[S52,S79] Finally, the case of mn21 exemplifies a possible pathway to engineer the band gaps of Ti-based materials for photocatalysis, which is often highlighted as a challenge within the scientific community.[S79]

A more pronounced reduction in the band gap is observed for MOFs containing a Au(III)-pyrazolate-based metal node (mn8, see Figure S45a). The Au-pyrazolate cyclic trinuclear

complex of mn8 has been suggested as a building block for MOFs in literature,[S80] and we adopted this suggestion to diversify the linker-metal node bond type and leverage the intriguing photocatalytic properties of Au-based materials.[S81–S84] Our results indicate that most structures with this metal node exhibit, apart from low band gaps in the visible range, appropriate band edge alignment to HER/OER reactions.

The presence of a V(IV) cluster (mn33) seems to have a similar behavior to that of mn8 regarding the band gap reduction (Figure S49a) and favorable alignment to the HER redox potential. Although there have been reports on the usage of V-MOFs for electrochemical water splitting[S85] or other photocatalytic reactions,[S86,S87] little is known about their performance as photocatalysts for HER and/or OER. Our findings suggest the possibility of utilizing V-based MOF photocatalysts for HER.

Regarding organic linkers, Figure S37 shows that the presence of ol57 (featuring pyrazine and thiophene groups), ol45 (containing hydroxyphenazine and thiophene groups), ol48 (with thiadiazole group) and ol3 (featuring thiophene group) tend to favor band edge alignment for HER and OER potentials. Among these, ol3 (Figure S51a), ol48 (Figure S53a), and ol57 (Figure S54) are associated with lower band gaps. Our choice on linkers containing thiadiazole was influenced by its prior association with lower band gap and favorable charge recombination descriptor values for COFs.[S28] A similar trend is observed here for ol48 (Figure S53), which contains the thiadiazole group.

Charge transport and charge separation Similar analyses for charge carrier effective masses and charge recombination are displayed in the Supporting Information (Figures S34–S43). For the charge carrier mobility descriptor, we observed notable trends:

MOFs with metal nodes mn13 (Cu(I) cluster, Figure S46), mn21 (Ti(IV)/Zr(IV) cluster, Figure S47b), mn33 (V(IV) cluster, Figure S49b) and mn34 (Co(III) cluster, Figure S50) exhibit on average lower electron and/or hole effective masses. Similarly, MOFs with linkers ol3 (with thiophene group, Figure S51b), ol31 (with thiophene, and carbonitrile groups,

Figure S52) showed the same trend.

Thiophene groups, known for their high π-electron density, have been widely used in MOFs and other photoactive materials.[S88] MOFs with thiophene groups reportedly display decent charge carrier mobility, which is in line with our findings that the presence of thiophene-containing ol3 linker contributes to lower electron effective masses (Figure S51b). Additionally, we observed that 2D MOFs with **hcb** topology also display lower charge carrier effective masses.[S89,S90] However, in CDP–MOFs with this topology, electrons and holes are more likely to recombine (charge separation descriptor, Figure S56b). On the other hand, the presence of Au-pyrazolate mn8 cluster correlates with more separated charge carriers (Figure S45b), but lower effective masses. As exemplified, we often notice a trade-off between charge transport and charge separation descriptors. However, our open-shell MOFs and rod MOFs displayed an average enhancement of both – leading to more separated and mobile charge carriers (Figures S58 and S59).

Another pathway to realize separated charge carriers is through metal node-to-linker (MLCT) or linker-to-metal node (LMCT) charge transfer. By analyzing electron and hole injection calculations, we notice that the Au-pyrazolate mn8 seems to be common among CDP–MOFs presenting LMCT, in agreement with recent studies stating that the pyrazolate SBU could enhance charge transfer when compared to its carboxylate counterpart.[S91] Other building blocks that are common among the MOFs that might present LMCT are metal node mn21 and organic linkers ol3 and ol57. Detailed information on the computation of the charge-transfer likelihood can be found in the Methods section.

4.1.1 Metal nodes

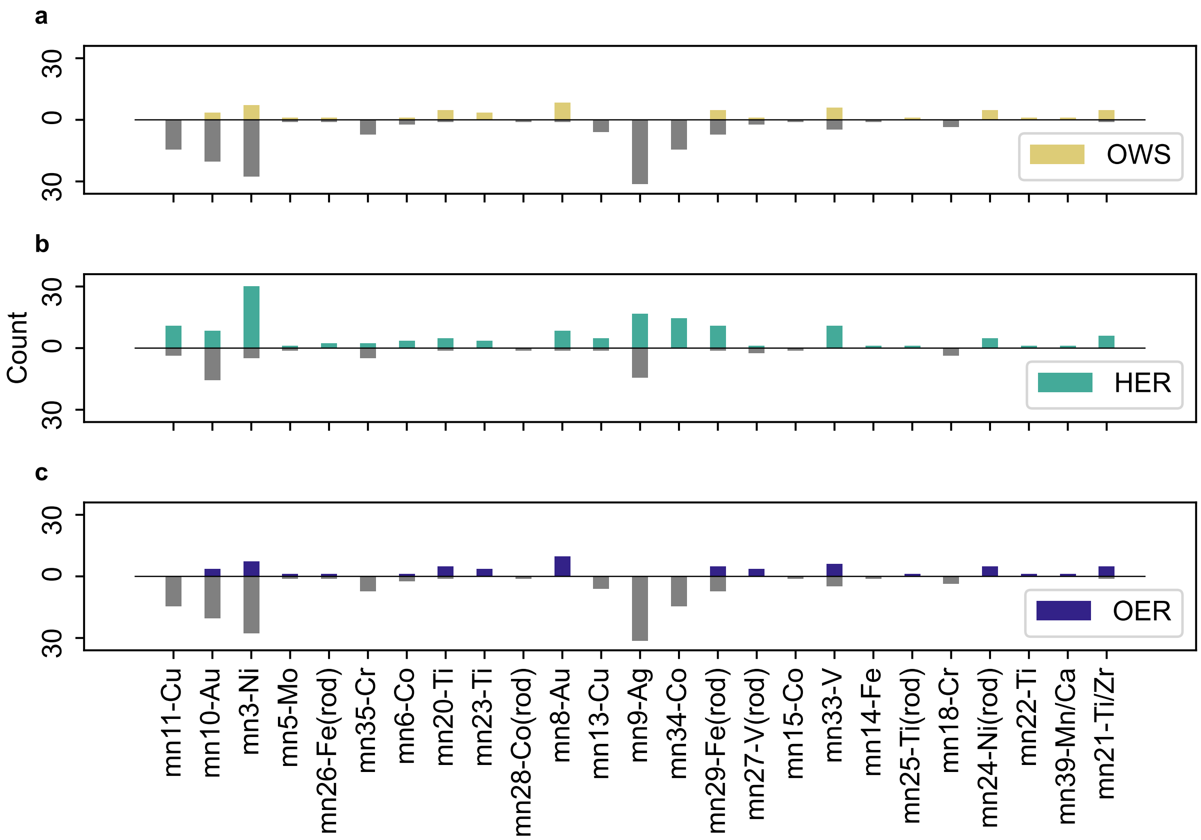

Figure S33: Impact of metal nodes on band edge alignment with redox potentials: HER (jungle green), OER (indigo blue), and both simultaneously (mustard yellow). Grey bars represent structures with misaligned band edges.

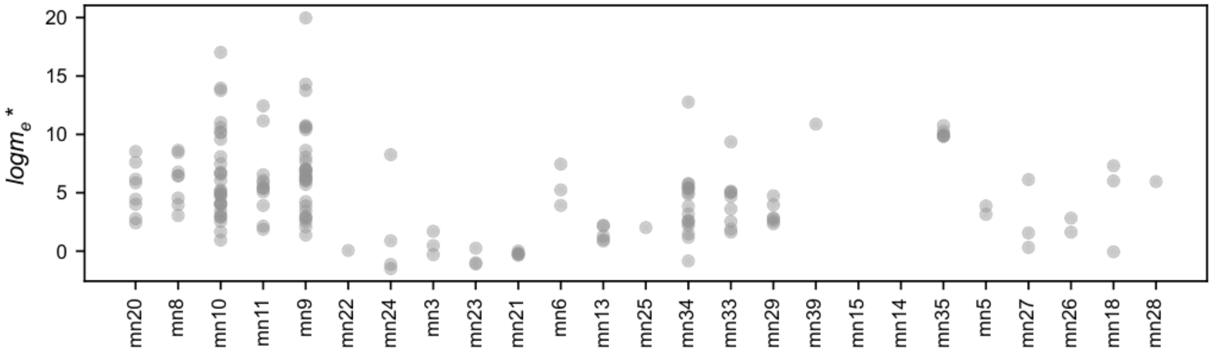

Figure S34: Effect of the presence of a specific metal node on the electron effective mass distribution.

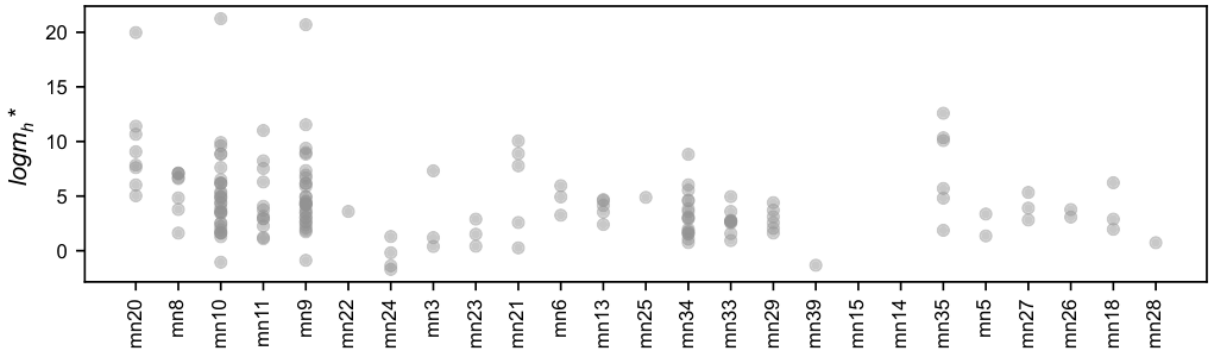

Figure S35: Effect of the presence of a specific metal node on the hole effective mass distribution.

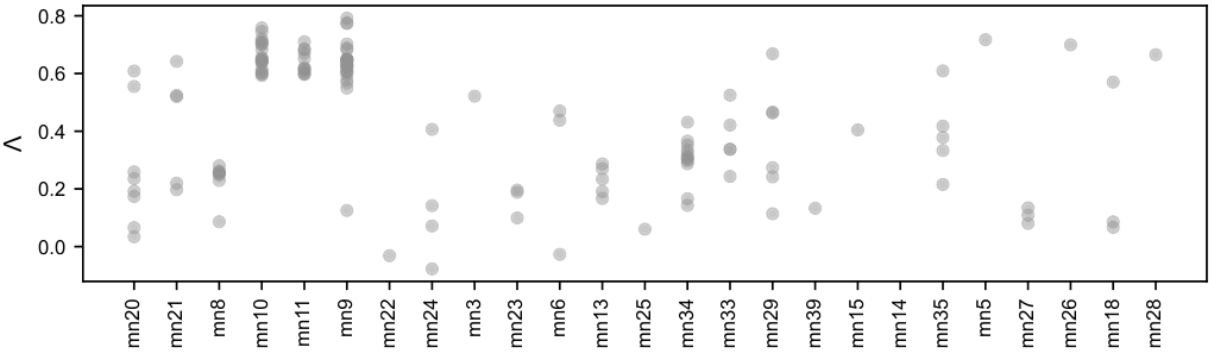

Figure S36: Effect of the presence of a specific metal node on the charge recombination descriptor (Λ).

4.1.2 Organic linkers

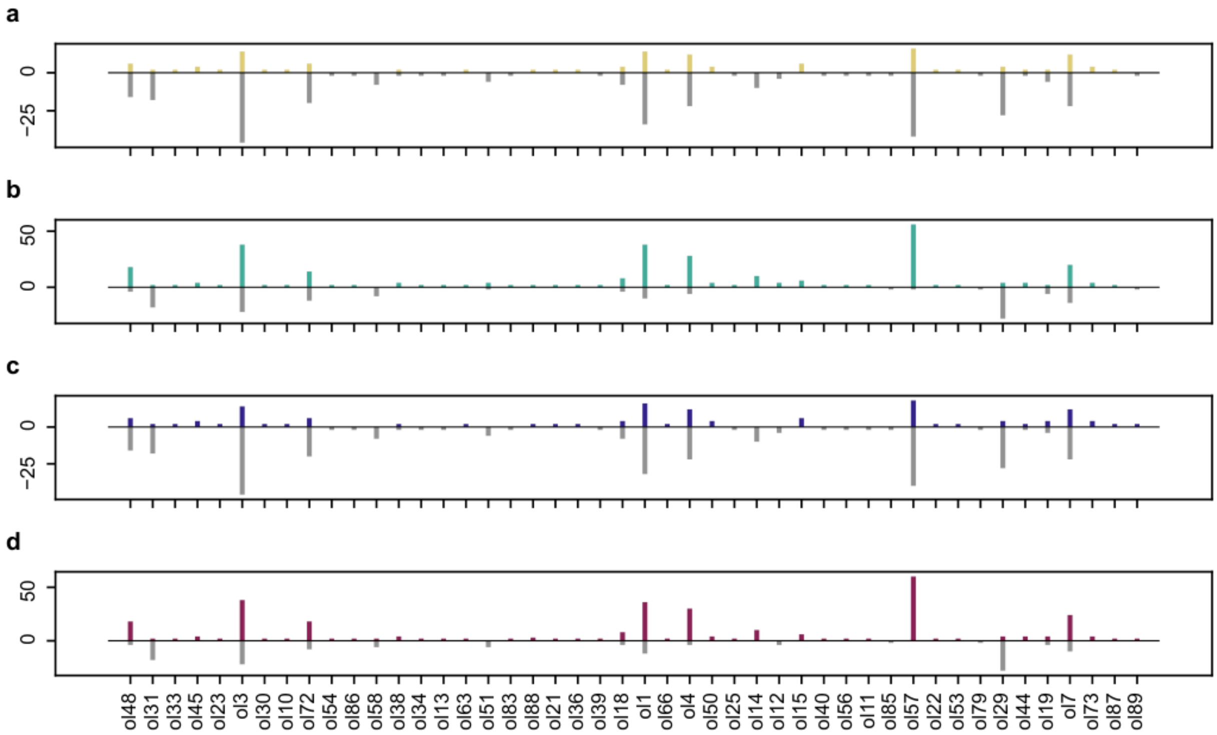

Figure S37: Effect of the presence of a specific organic linker on the band edge alignment to the redox potentials of a) HER and OER simultaneously (mustard yellow), b) HER (jungle green), and c) OER (indigo blue). d) Effect of the presence of a specific organic linker on suitable band gaps for visible light absorption (maroon). The positive height of the bars is associated with the number of structures that present the right band edge alignment. Grey bars in the negative part of the plots represent the number of structures whose band edges do not align to either HER or OER redox potentials.

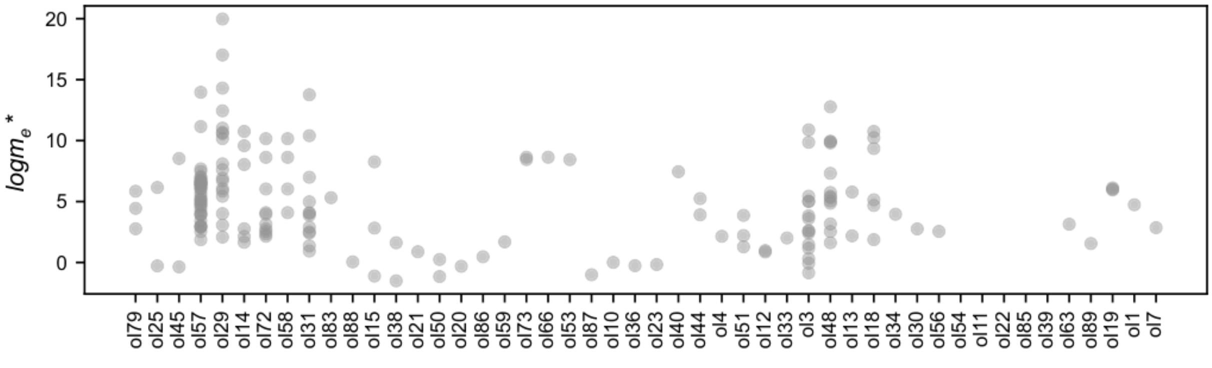

Figure S38: Effect of the presence of a specific organic linker on the electron effective mass distribution.

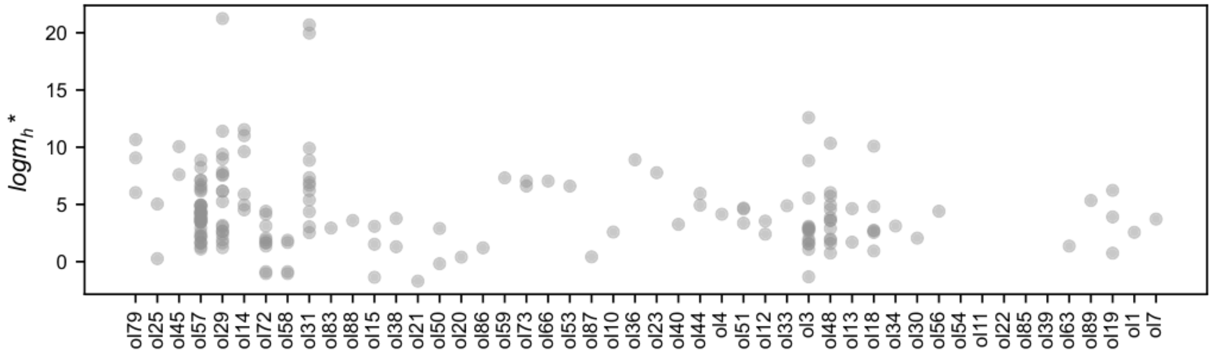

Figure S39: Effect of the presence of a specific organic linker on the hole effective mass distribution.

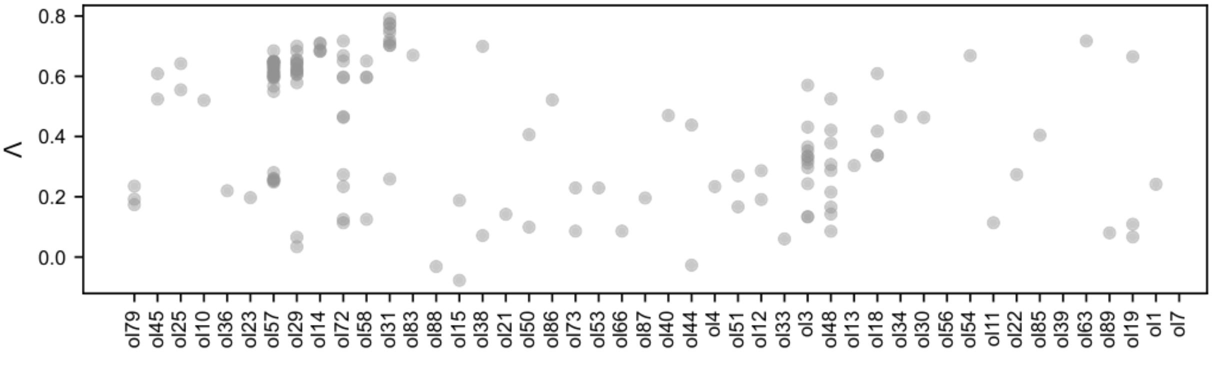

Figure S40: Effect of the presence of a specific organic linker on the charge recombination descriptor (Λ).

4.1.3 Topologies

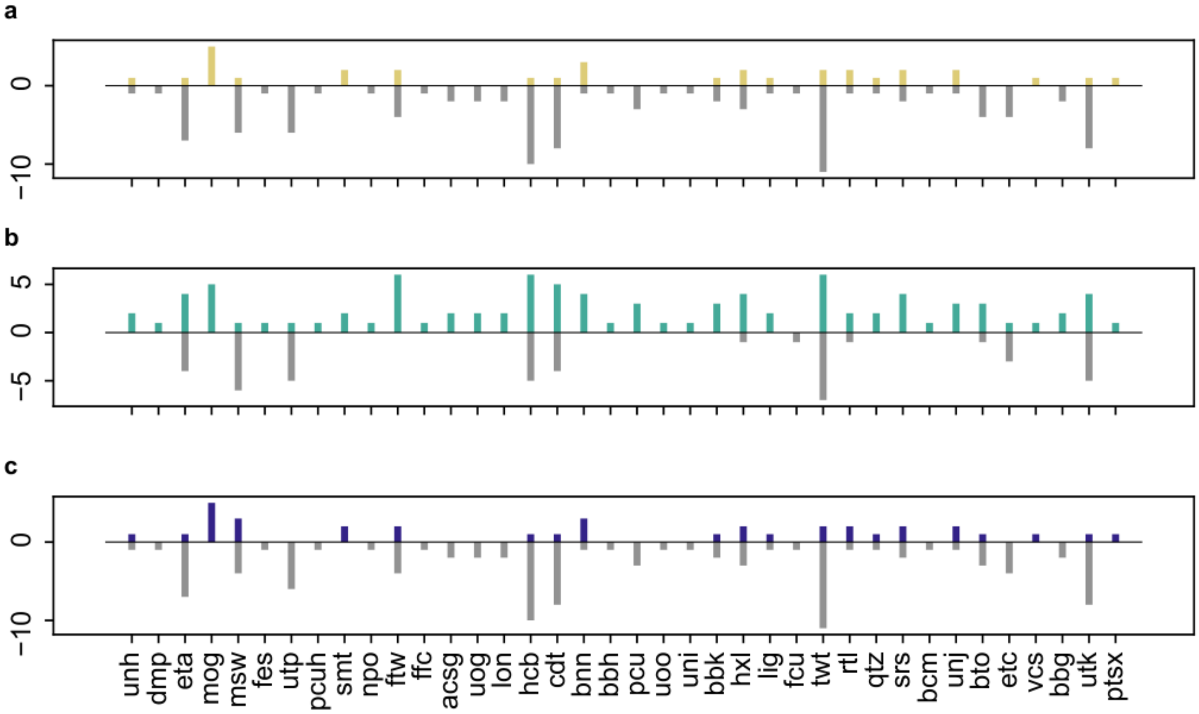

Figure S41: Effect of the presence of a specific topology on the band edge alignment to the redox potentials of a) HER and OER simultaneously (mustard yellow), b) HER (jungle green), and c) OER (indigo blue). The positive height of the bars is associated with the number of structures that present the right band edge alignment. Grey bars in the negative part of the plots represent the number of structures whose band edges do not align to either HER or OER redox potentials.

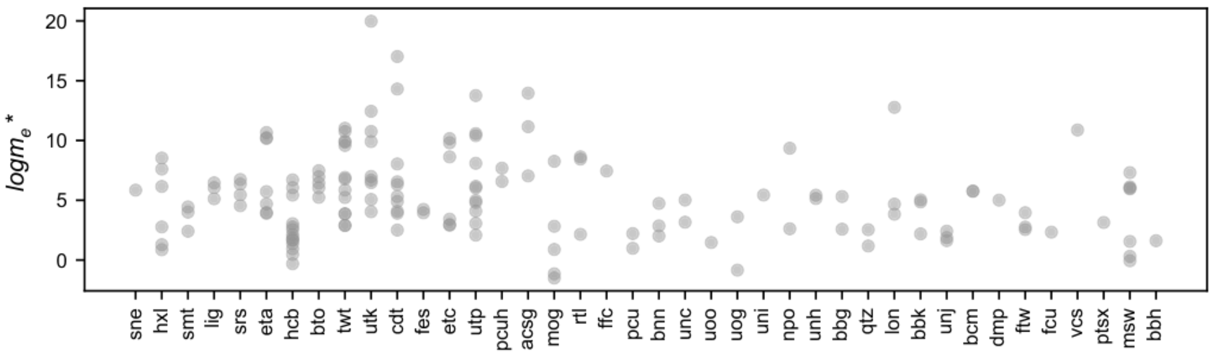

Figure S42: Effect of the presence of a specific topology on the electron effective mass distribution.

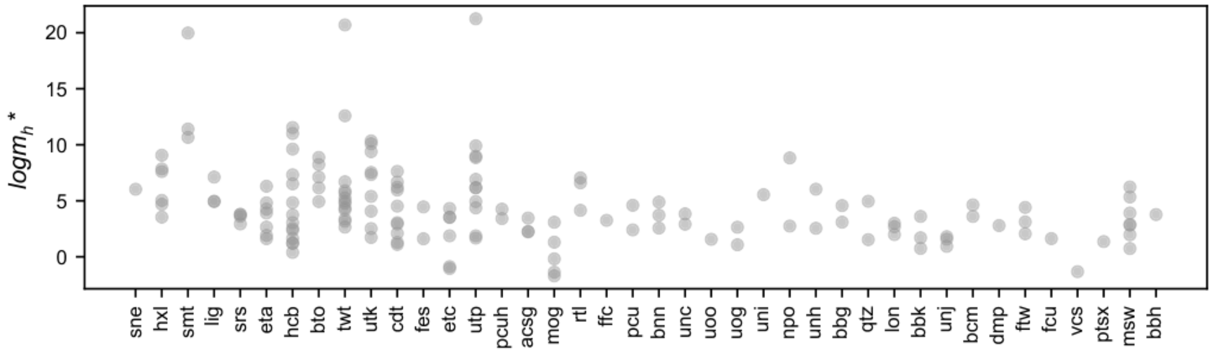

Figure S43: Effect of the presence of a specific topology on the hole effective mass distribution.

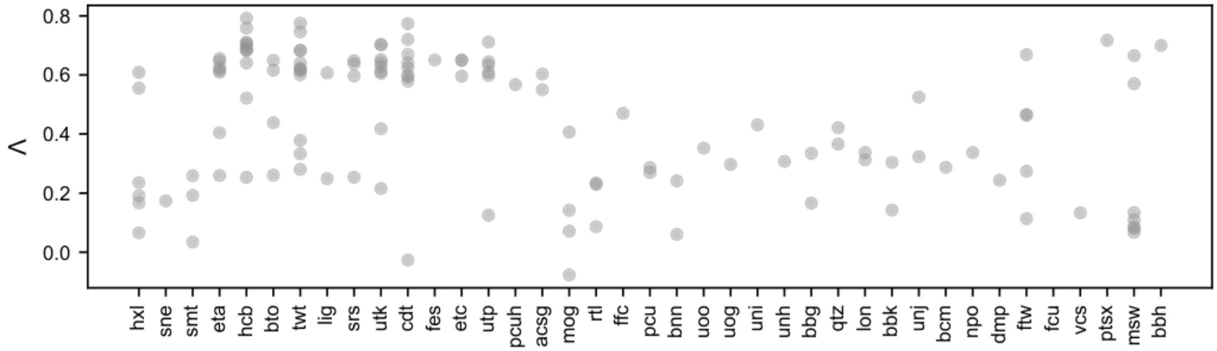

Figure S44: Effect of the presence of a specific topology on the charge recombination descriptor (Λ).

4.1.4 Bootstrapped effect sizes

We performed bootstrapped effect sizes analysis to investigate whether the presence of a building block or functional group could have positive, negative, or no correlation with our photocatalytic DFT descriptors. We used the DABEST package for quantitative estimation of effect sizes.

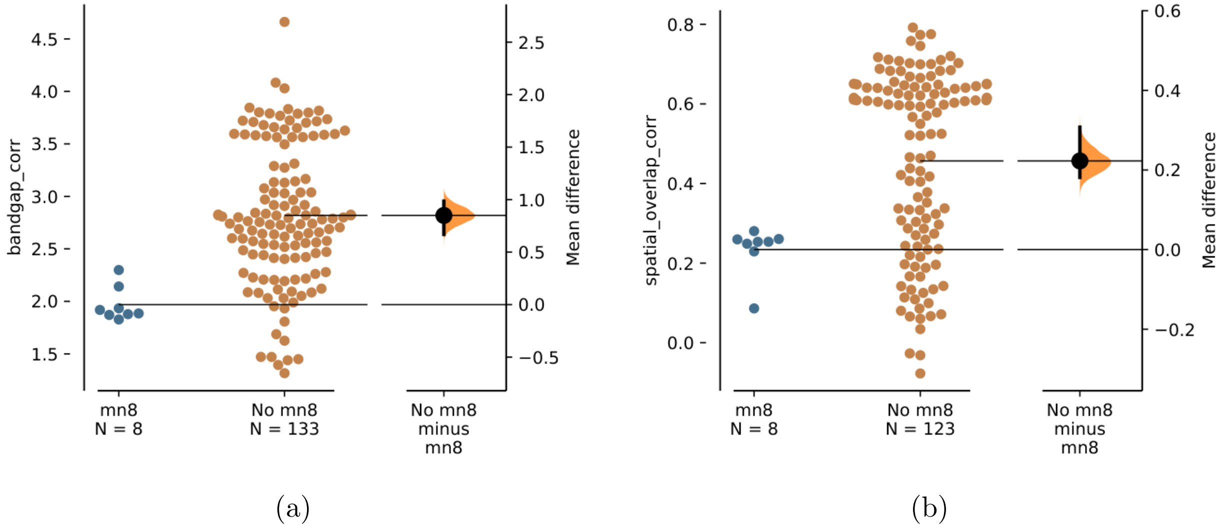

Figure S45: Bootstrapped effect sizes with DABEST[S92] comparing subsets of structures with and without the presence of mn8 w.r.t. a) band gap values, b) charge recombination descriptor (Λ).

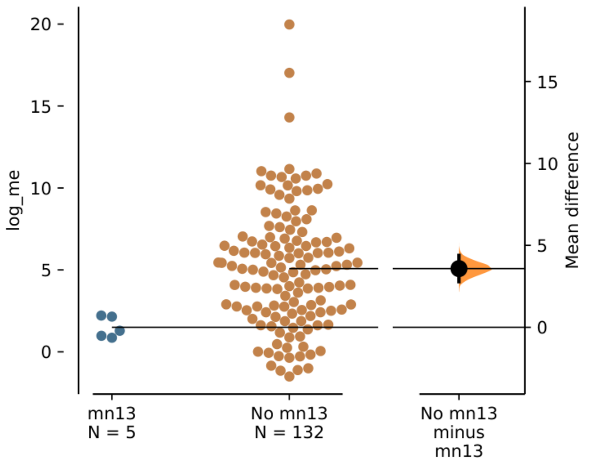

Figure S46: Bootstrapped effect sizes with DABEST[S92] comparing subsets of structures with and without the presence of mn13 w.r.t. electron effective mass values. The figure shows a lowering effect on the electron effective mass when mn13 is present.

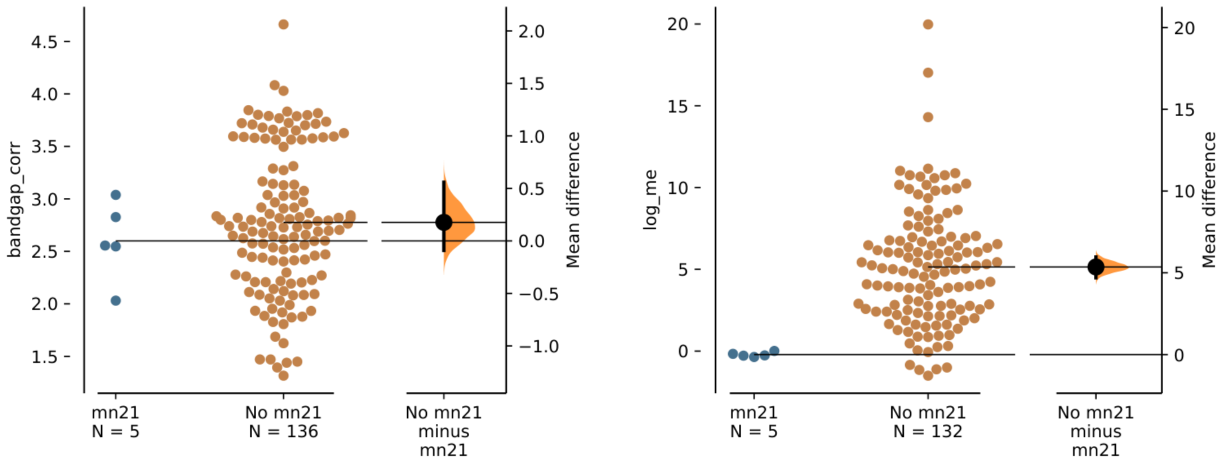

(a) (b)

Figure S47: Bootstrapped effect sizes with DABEST[S92] comparing subsets of structures with and without the presence of mn21 w.r.t. a) band gap values, b) electron effective mass ($m*_e$).

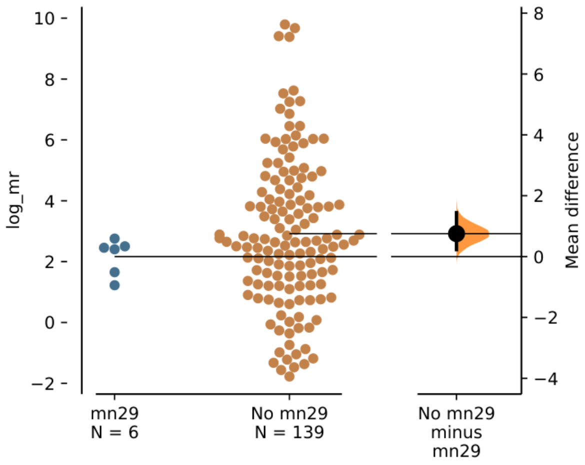

Figure S48: Bootstrapped effect sizes with DABEST[S92] comparing subsets of structures with and without the presence of mn29 w.r.t. reduced effective mass ($m*_{reduced}$).

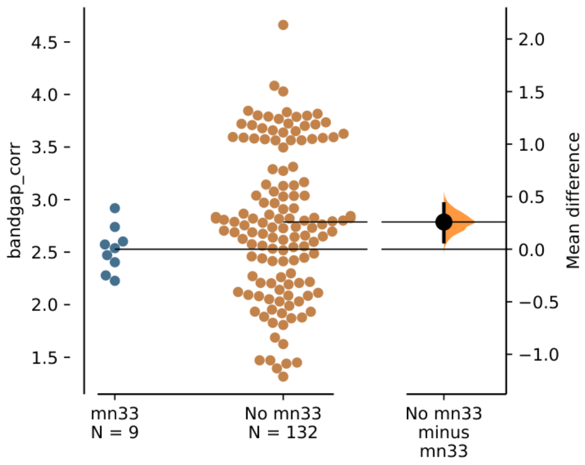

(a)

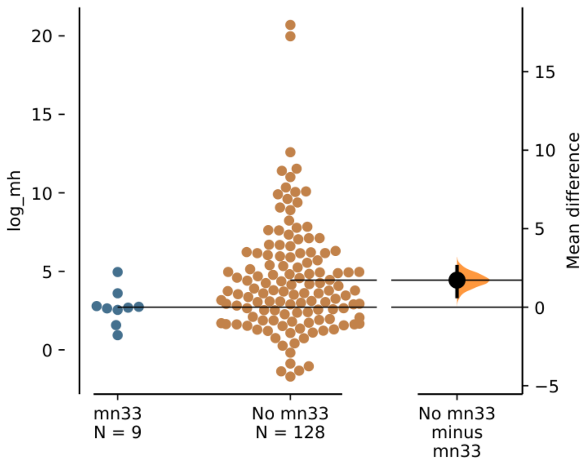

(b)

Figure S49: Bootstrapped effect sizes with DABEST[S92] comparing subsets of structures with and without the presence of mn33 w.r.t. a) band gap values, b) hole effective mass ($m*_e$).

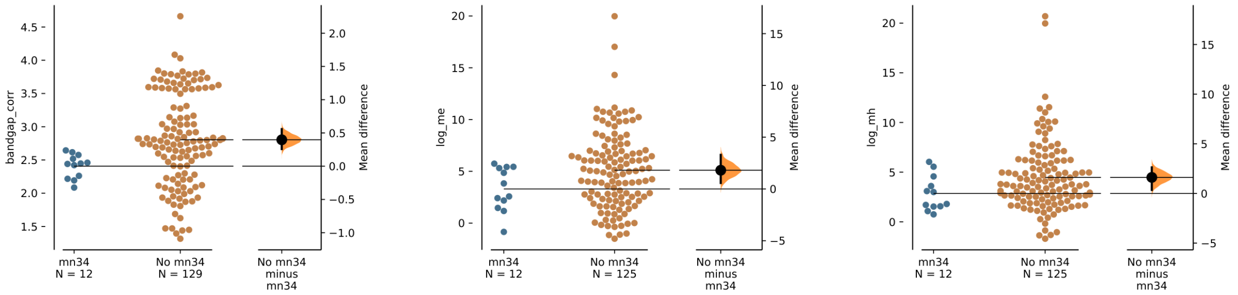

(a) (b) (c)

Figure S50: Bootstrapped effect sizes with DABEST[S92] comparing subsets of structures with and without the presence of mn34 w.r.t. a) band gap values, b) electron ($m*_e$), and c) hole ($m*_h$) effective masses.

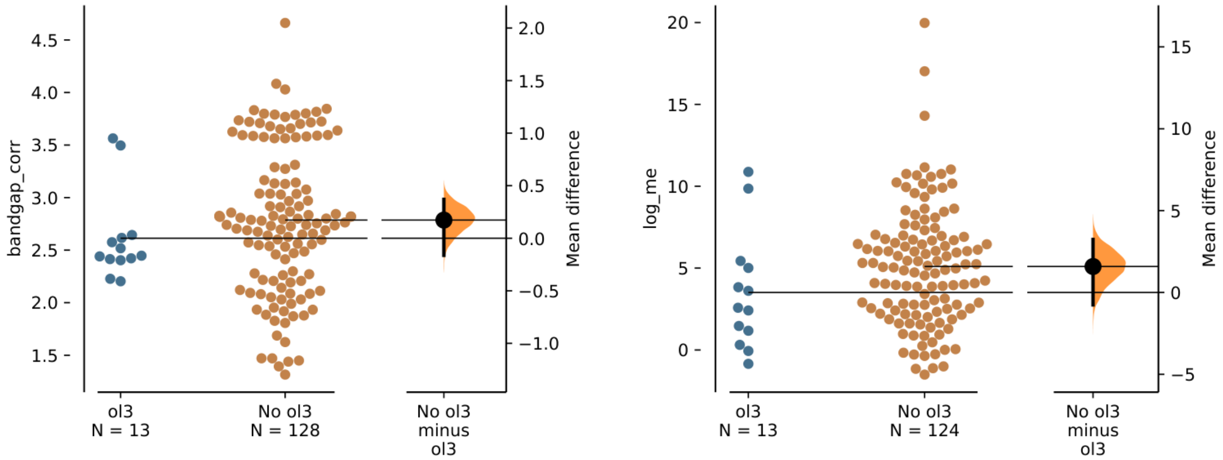

(a) (b)

Figure S51: Bootstrapped effect sizes with DABEST[S92] comparing subsets of structures with and without the presence of ol3 w.r.t. a) band gap values, b) electron effective mass ($m*_e$).

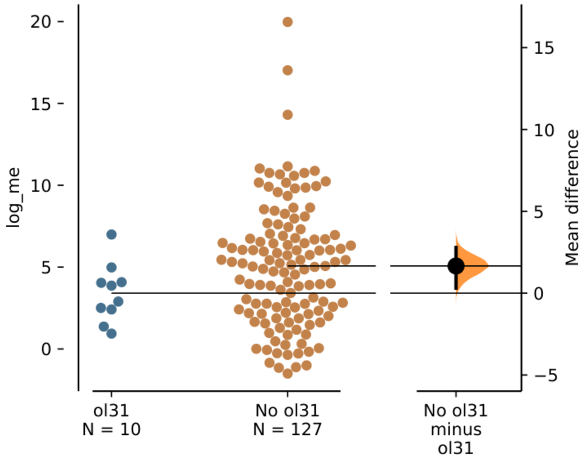

Figure S52: Bootstrapped effect sizes with DABEST[S92] comparing subsets of structures with and without the presence of ol31 w.r.t. electron effective mass values. The figure shows a lowering effect on the electron effective mass when ol31 is present.

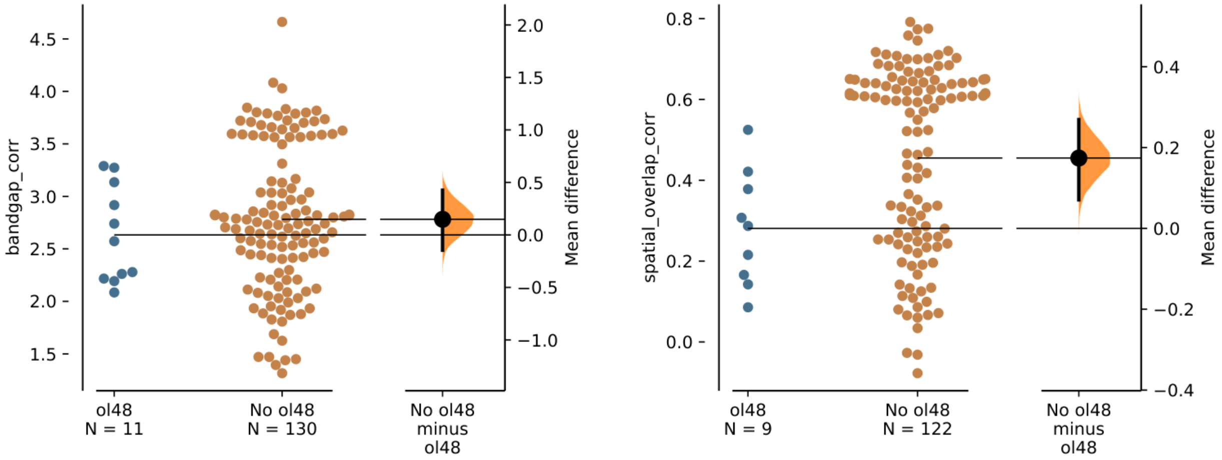

(a) (b)

Figure S53: Bootstrapped effect sizes with DABEST[S92] comparing subsets of structures with and without the presence of ol48 w.r.t. a) band gap values, b) charge recombination descriptor (Λ).

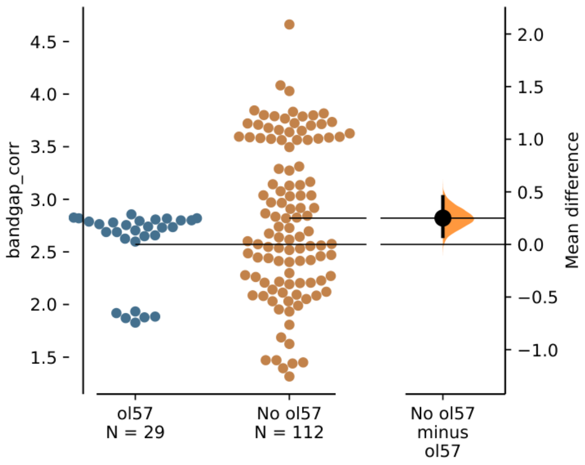

Figure S54: Bootstrapped effect sizes with DABEST[S92] comparing subsets of structures with and without the presence of ol57 w.r.t. band gap values. The figure shows a lowering effect on the band gap when ol57 is present.

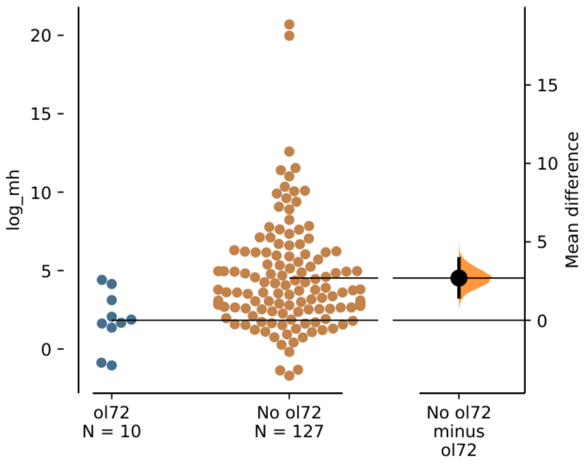

Figure S55: Bootstrapped effect sizes with DABEST[S92] comparing subsets of structures with and without the presence of ol72 w.r.t. hole effective mass values. The figure shows a lowering effect on the hole effective mass when ol72 is present.

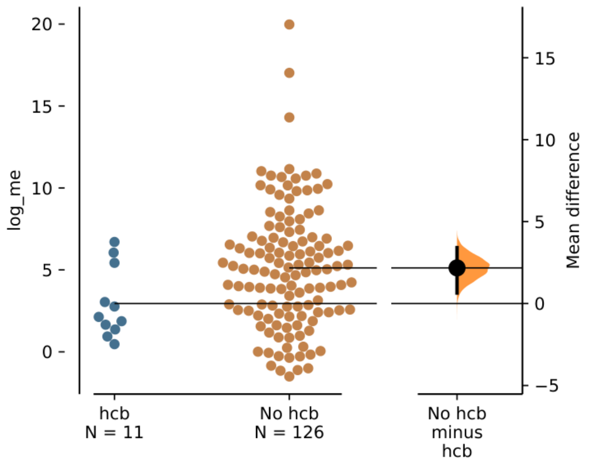

(a)

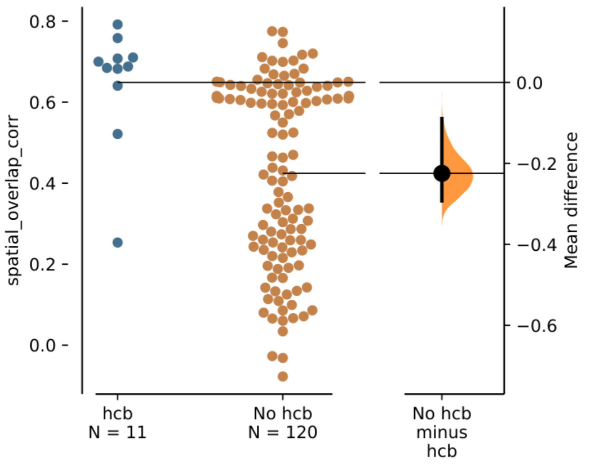

(b)

Figure S56: Bootstrapped effect sizes with DABEST[S92] comparing subsets of structures with and without the presence of **hcb** topology w.r.t. a) electron effective mass ($m*_e$), and b) charge recombination descriptor (Λ).

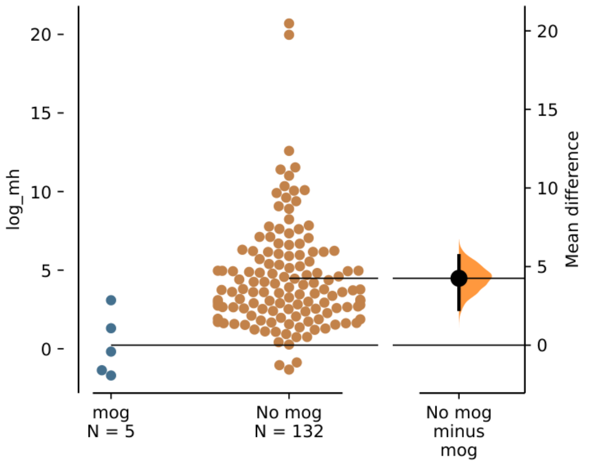

Figure S57: Bootstrapped effect sizes with DABEST[S92] comparing subsets of structures with and without the presence of **mog** topology w.r.t. hole effective mass values. The figure shows a lowering effect on the hole effective mass when **mog** topology is present.

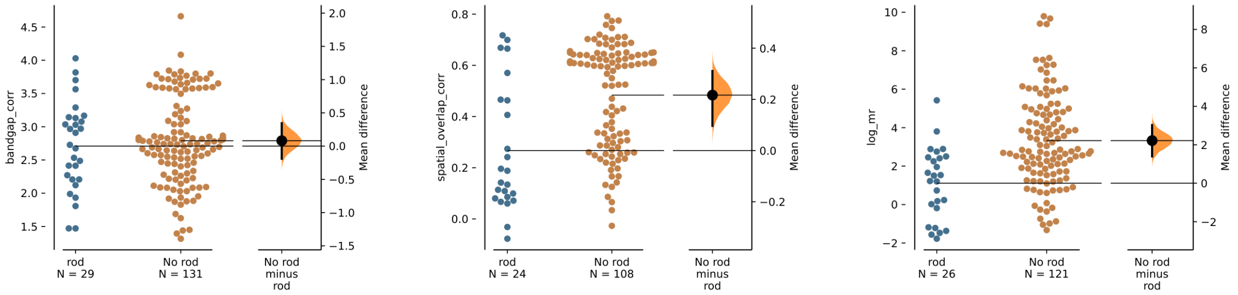

(a) (b) (c)

Figure S58: Bootstrapped effect sizes with DABEST[S92] comparing subsets of structures with and without the presence of a 1D metal node (rod-like MOF) w.r.t. a) band gap values, b) charge recombination descriptor (Λ), and c) reduced effective mass ($m*_{reduced}$).

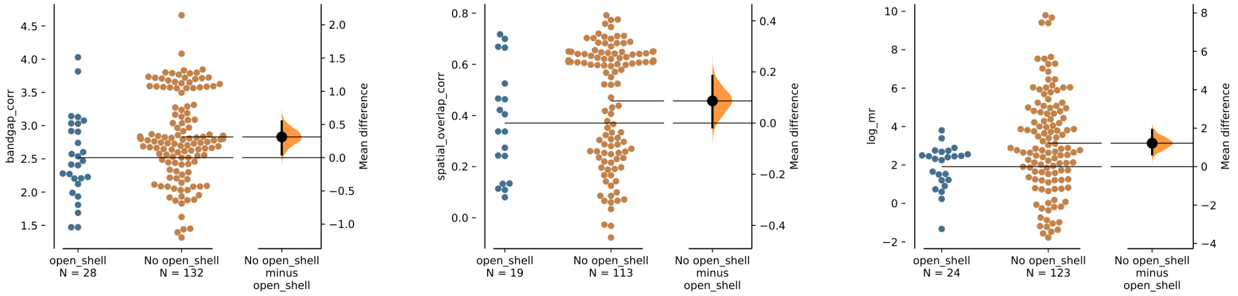

(a) (b) (c)

Figure S59: Bootstrapped effect sizes with DABEST[S92] comparing subsets of structures with and without the presence of an open shell metal w.r.t. a) band gap values, b) charge recombination descriptor (Λ), and c) reduced effective mass ($m*_{reduced}$).

4.2 Structure-property relationship analysis on all predictions

In Table 2 the building blocks that showed a statistical correlation effect with the predictions for each descriptor were highlighted. The smiles for organic linkers are shown in Table S2 and can be visualized in Figure 5.

Table S8: List of organic linkers.

ol	name
O1	[O-]C(=O)c1ccc(c2c1CC2)C(=O)[O-]
O2	[O-]C(=O)C#Cc1ccc(cc1)C#CC(=O)[O-]
O3	s1nc2c(n1)[C](S[C]2c1ccc(s1)[C]1C=NN=C1)c1ccc(s1)C1=C[N]N=C1
O4	[O-]C(=O)c1ccc(cc1)c1ccc(cc1)C(=O)[O-]
O5	[O-]C(=O)c1ccc2c(c1)ccc(c2)C(=O)[O-]
O6	n1ccncc1
O7	[O-]C(=O)c1ccc(cc1)Oc1ccc(cc1)C(=O)[O-]
O8	[O-]C(=O)c1c(F)c(F)c(c(c1F)F)C(=O)[O-]
O9	[O-]C(=O)c1cc(cc(c1)N(=O)=O)C(=O)[O-]
O10	[O-]C(=O)c1cc(cc(c1)C(=O)[O-])C(=O)[O-]
O11	[O-]C(=O)CCCC(=O)[O-]
O12	N#C[N]C#N
O13	[O-]C(=O)c1ccc(cc1)C#Cc1ccc(cc1)C(=O)[O-]
O14	[N]1N=CC(=C1)c1sc(c2c1nccn2)C1=CN=N[CH]1
O15	[O-]C(=O)C#CC#CC(=O)[O-]

The bootstrapped effect sizes analysis performed for each motif can be seen in the Metal nodes and Organic linkers sections below. The plots show the comparison between the percentage of success (*i.e.* percentage of structures for which property prediction is 1) of MOFs containing the highlighted motif *vs* MOFs that do not contain it.

4.2.1 Organic linkers

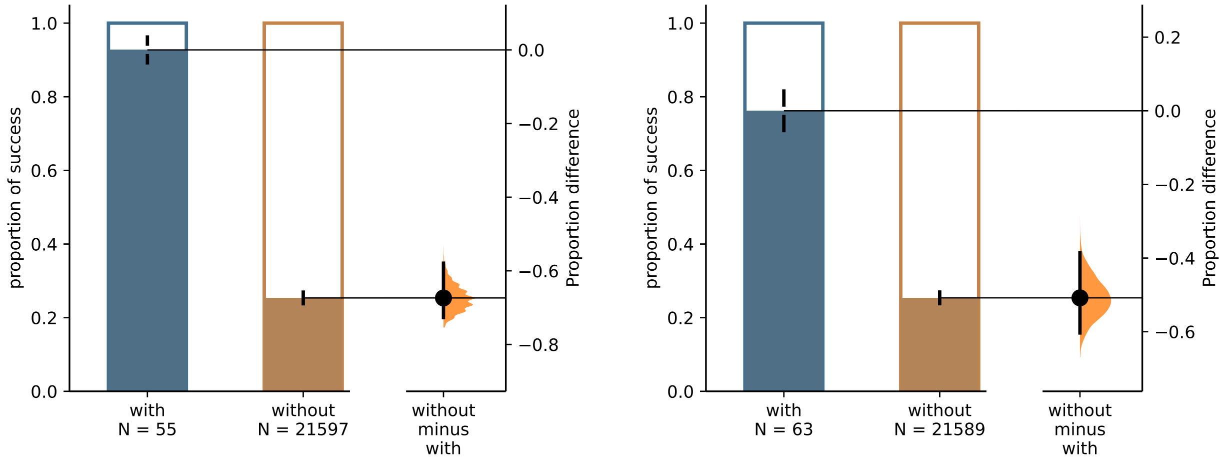

(a) (b)

Figure S60: Bootstrapped effect sizes with DABEST[S92] comparing subsets of structures with and without the presence of O1 w.r.t. visible light absorption with a. MOFTransformer and b. GPT-J predictions.

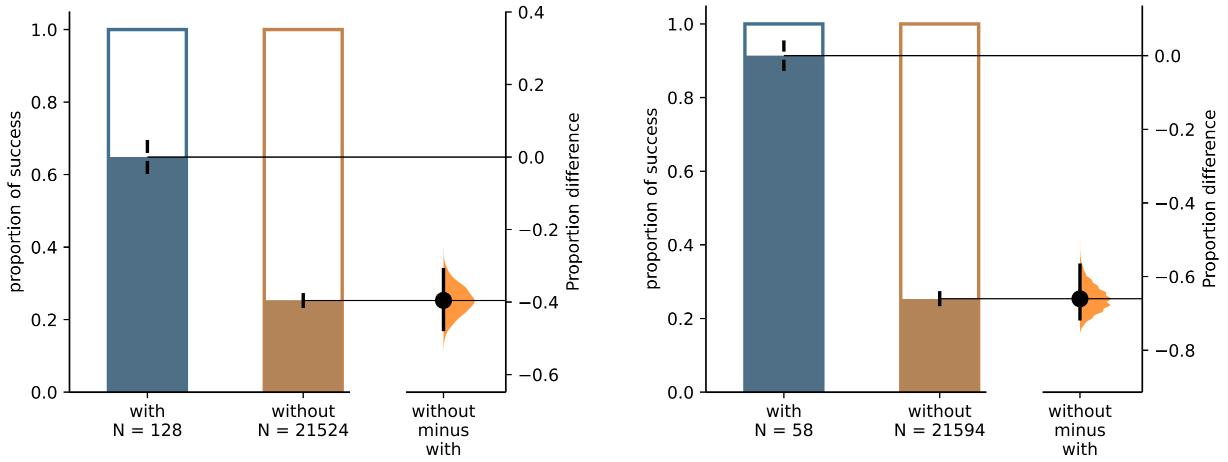

(a) (b)

Figure S61: Bootstrapped effect sizes with DABEST[S92] comparing subsets of structures with and without the presence of O2 w.r.t. visible light absorption with a. MOFTransformer and b. GPT-J predictions.

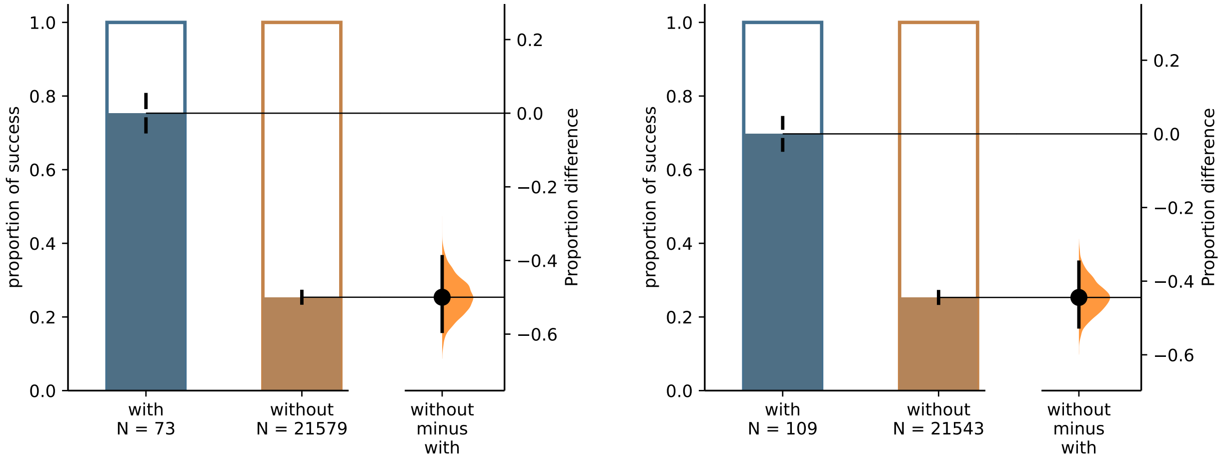

(a) (b)

Figure S62: Bootstrapped effect sizes with DABEST[S92] comparing subsets of structures with and without the presence of O3 w.r.t. visible light absorption with a. MOFTransformer and b. GPT-J predictions.

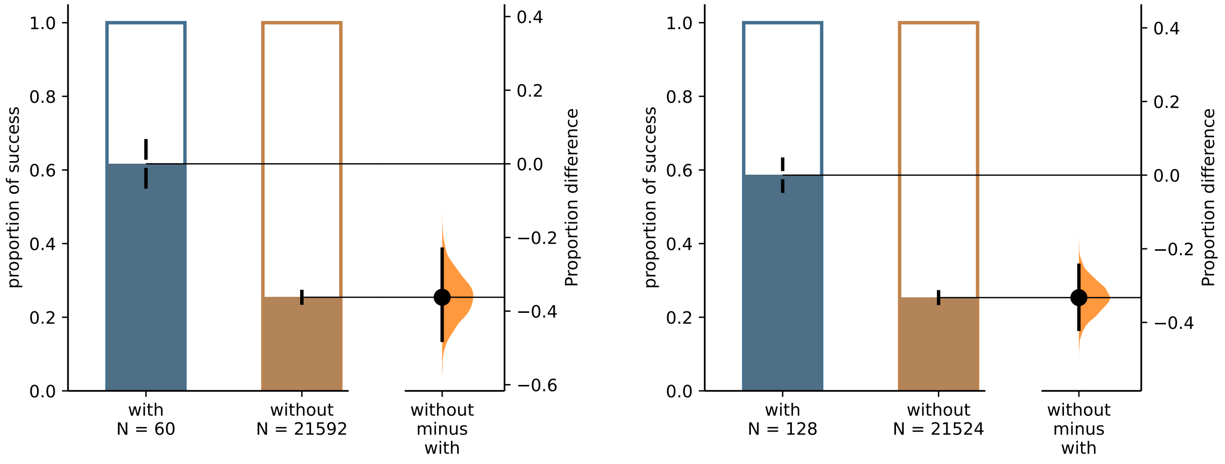

(a) (b)

Figure S63: Bootstrapped effect sizes with DABEST[S92] comparing subsets of structures with and without the presence of O4 w.r.t. visible light absorption with a. MOFTransformer and b. GPT-J predictions.

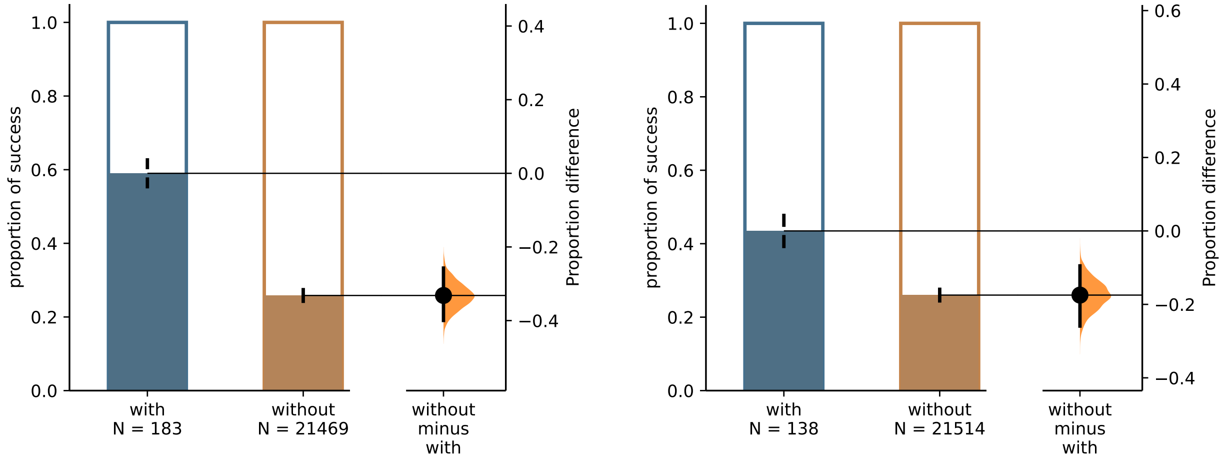

(a) (b)

Figure S64: Bootstrapped effect sizes with DABEST[S92] comparing subsets of structures with and without the presence of O5 w.r.t. m^*_{red} with a. MOFTransformer and b. GPT-J predictions.

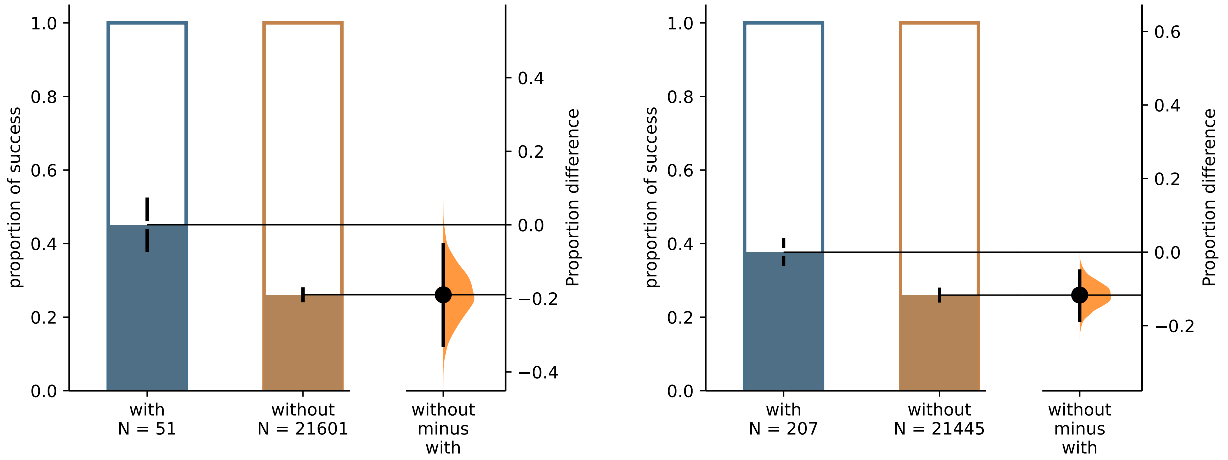

(a) (b)

Figure S65: Bootstrapped effect sizes with DABEST[S92] comparing subsets of structures with and without the presence of O6 w.r.t. m^*_{red} with a. MOFTransformer and b. GPT-J predictions.

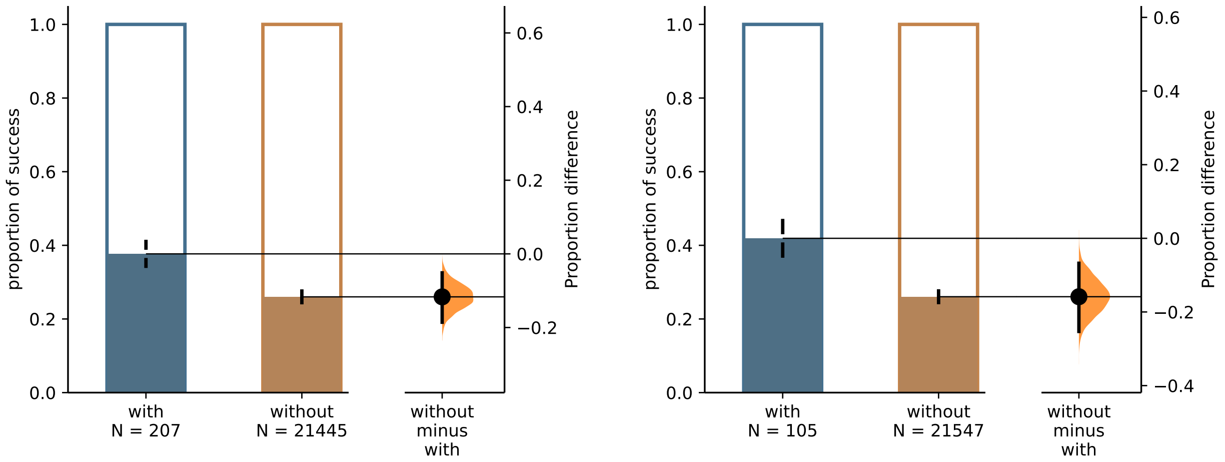

(a) (b)

Figure S66: Bootstrapped effect sizes with DABEST[S92] comparing subsets of structures with and without the presence of O7 w.r.t. m^*_{red} with a. MOFTransformer and b. GPT-J predictions.

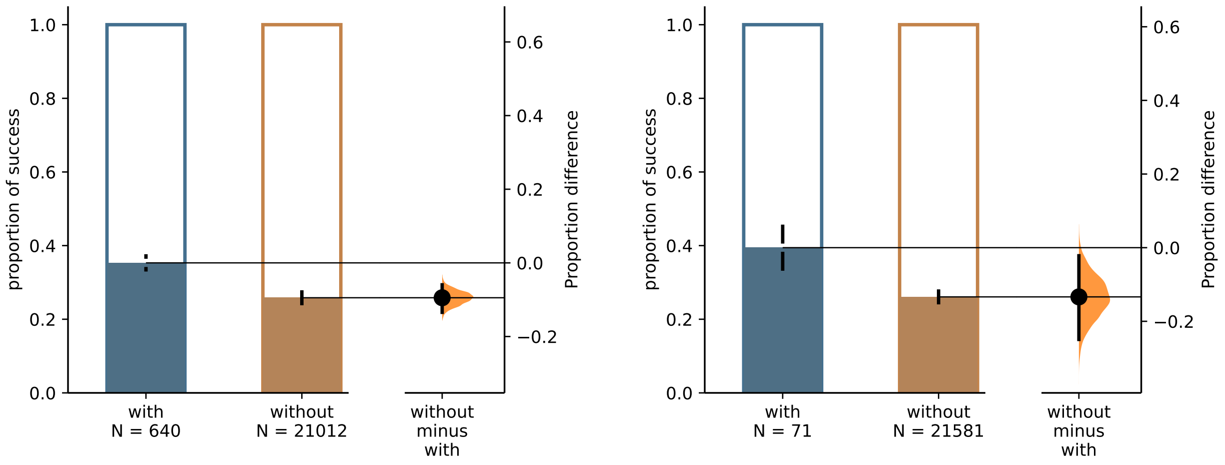

(a) (b)

Figure S67: Bootstrapped effect sizes with DABEST[S92] comparing subsets of structures with and without the presence of O8 w.r.t. m^*_{red} with a. MOFTransformer and b. GPT-J predictions.

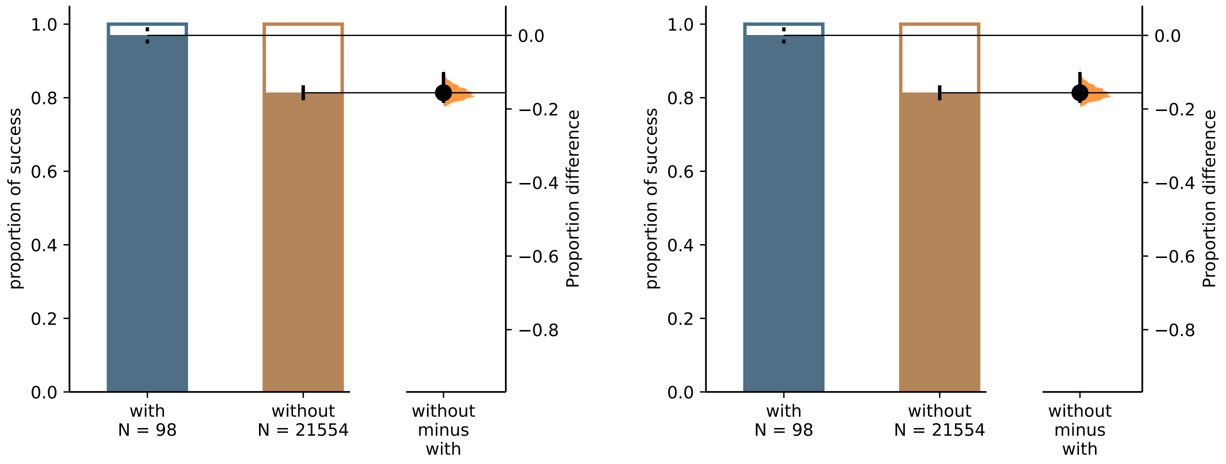

(a) (b)

Figure S68: Bootstrapped effect sizes with DABEST[S92] comparing subsets of structures with and without the presence of O9 w.r.t. Λ with a. MOFTransformer and b. GPT-J predictions.

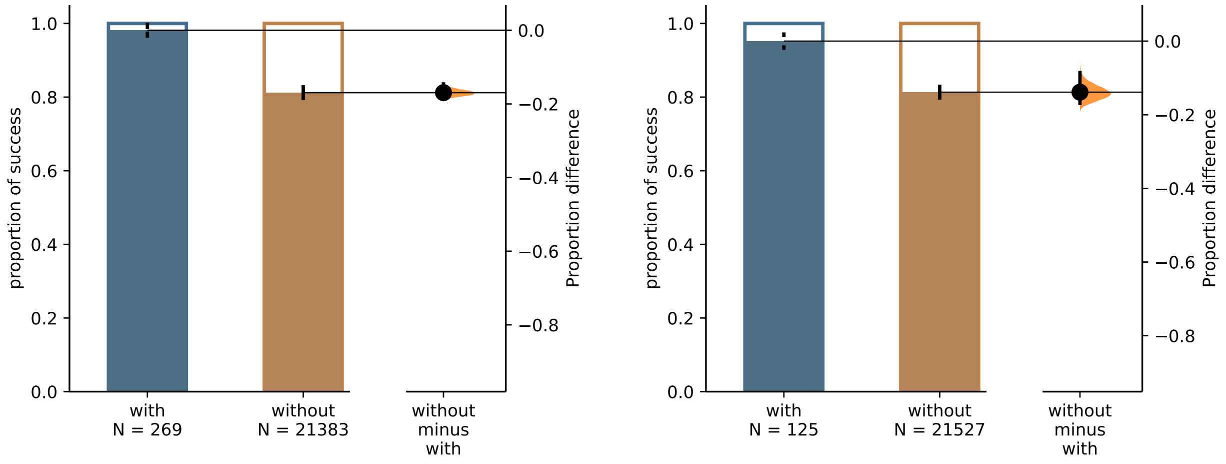

(a) (b)

Figure S69: Bootstrapped effect sizes with DABEST[S92] comparing subsets of structures with and without the presence of 10 w.r.t. Λ with a. MOFTransformer and b. GPT-J predictions.

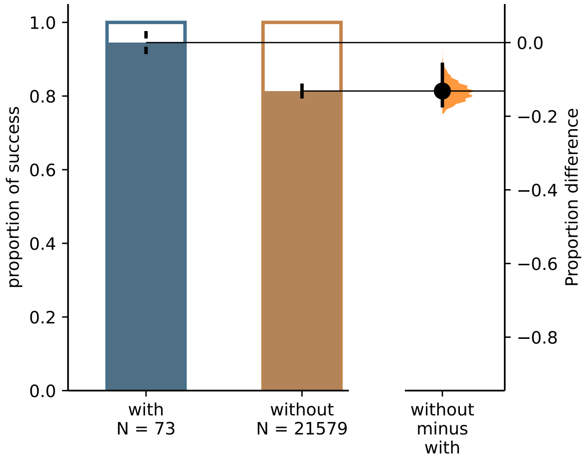

(a)

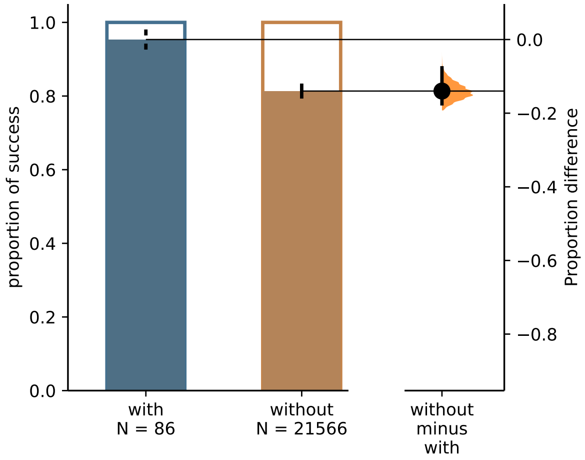

(b)

Figure S70: Bootstrapped effect sizes with DABEST[S92] comparing subsets of structures with and without the presence of O11 w.r.t. Λ with a. MOFTransformer and b. GPT-J predictions.

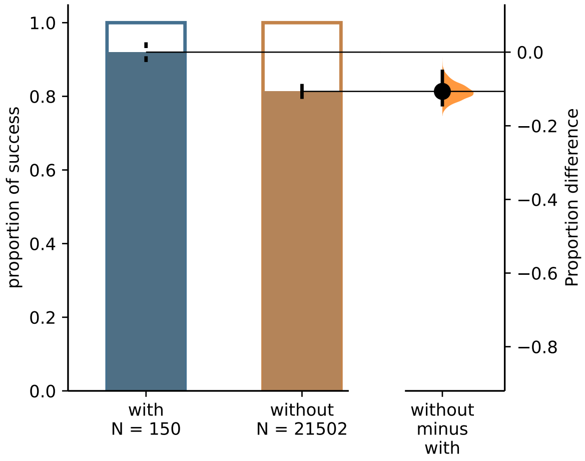

(a)

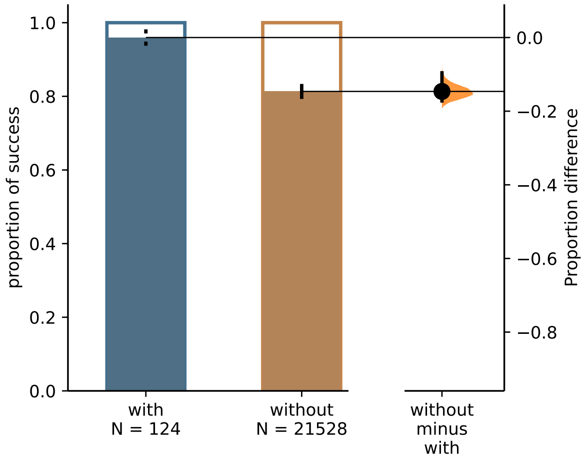

(b)

Figure S71: Bootstrapped effect sizes with DABEST[S92] comparing subsets of structures with and without the presence of O12 w.r.t. Λ with a. MOFTransformer and b. GPT-J predictions.

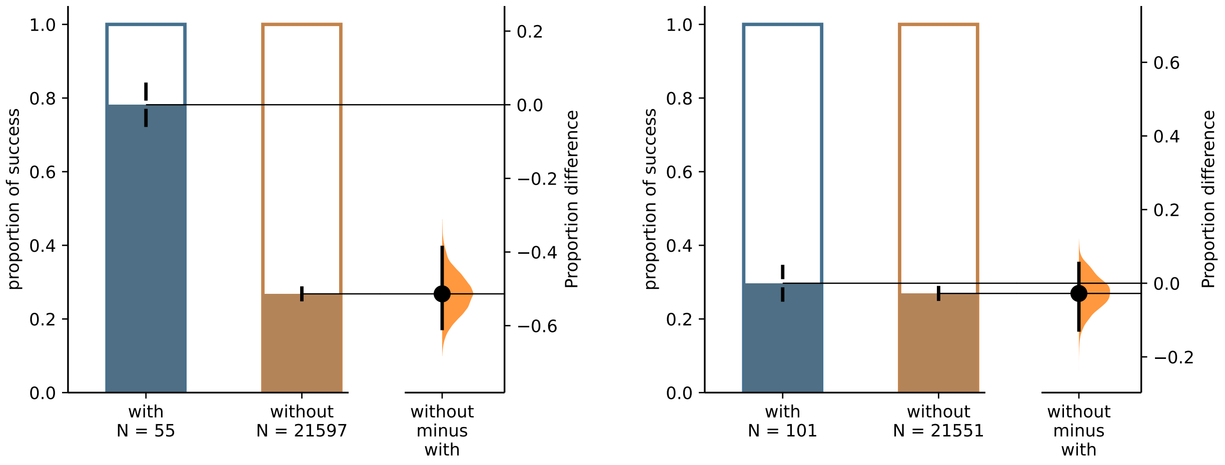

(a) (b)

Figure S72: Bootstrapped effect sizes with DABEST[S92] comparing subsets of structures with and without the presence of O1 w.r.t. HER with a. MOFTransformer and b. GPT-J predictions.

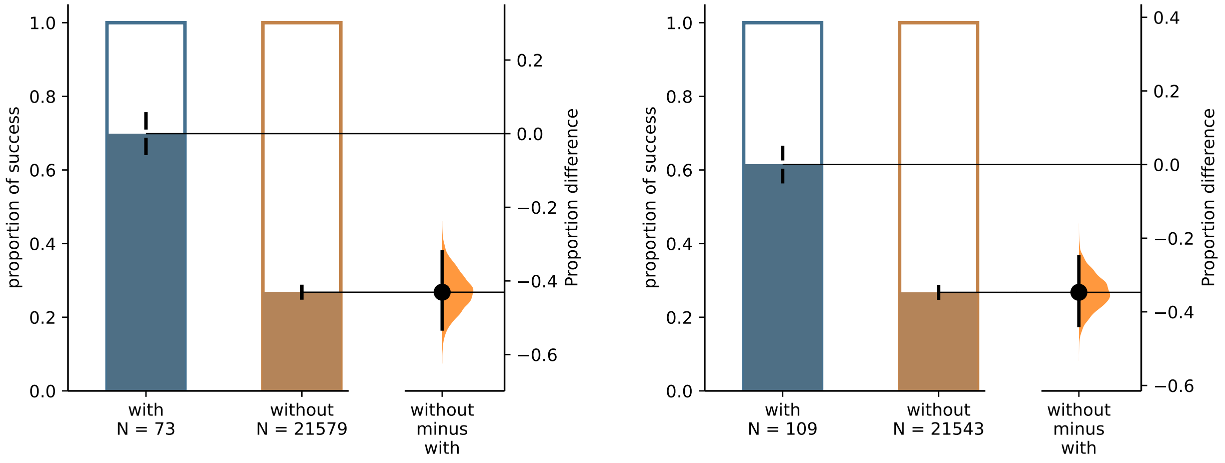

(a) (b)

Figure S73: Bootstrapped effect sizes with DABEST[S92] comparing subsets of structures with and without the presence of O3 w.r.t. HER with a. MOFTransformer and b. GPT-J predictions.

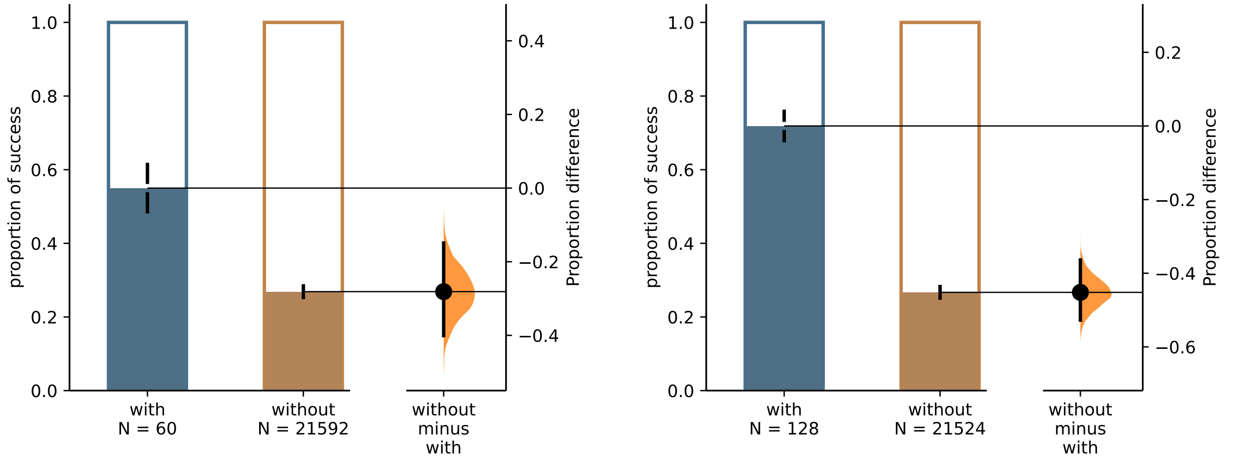

(a) (b)

Figure S74: Bootstrapped effect sizes with DABEST[S92] comparing subsets of structures with and without the presence of O4 w.r.t. HER with a. MOFTransformer and b. GPT-J predictions.

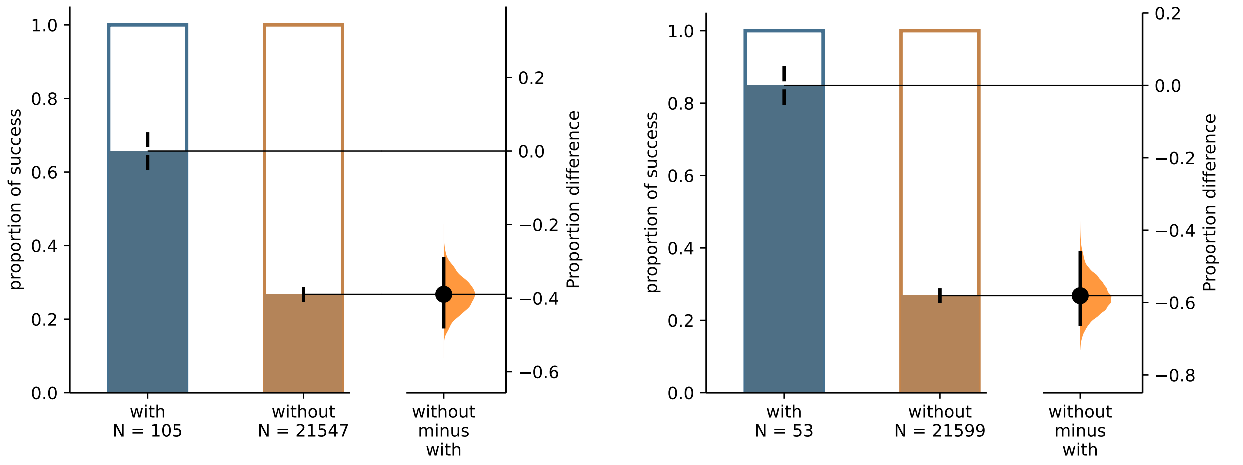

(a) (b)

Figure S75: Bootstrapped effect sizes with DABEST[S92] comparing subsets of structures with and without the presence of O13 w.r.t. HER with a. MOFTransformer and b. GPT-J predictions.

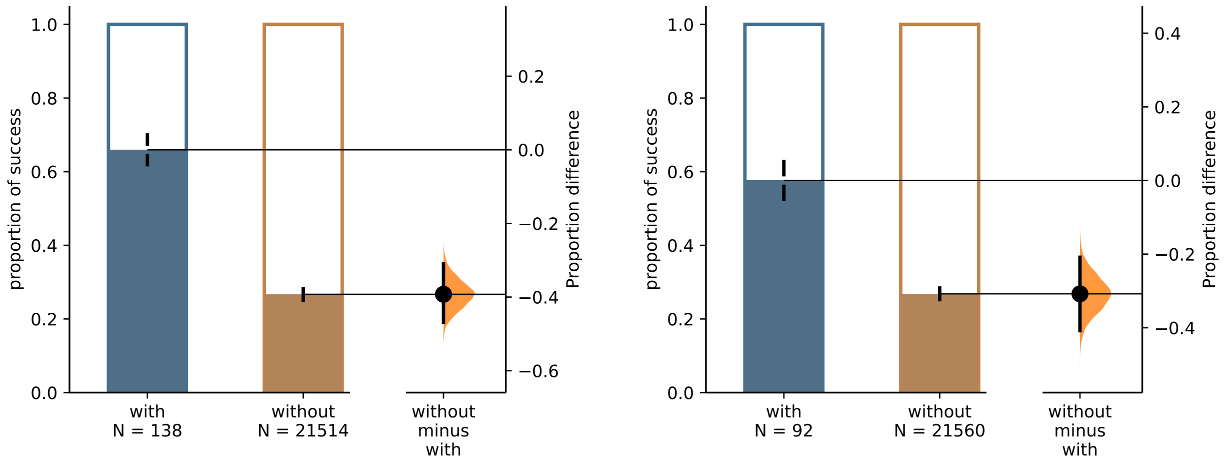

(a) (b)

Figure S76: Bootstrapped effect sizes with DABEST[S92] comparing subsets of structures with and without the presence of O14 w.r.t. HER with a. MOFTransformer and b. GPT-J predictions.

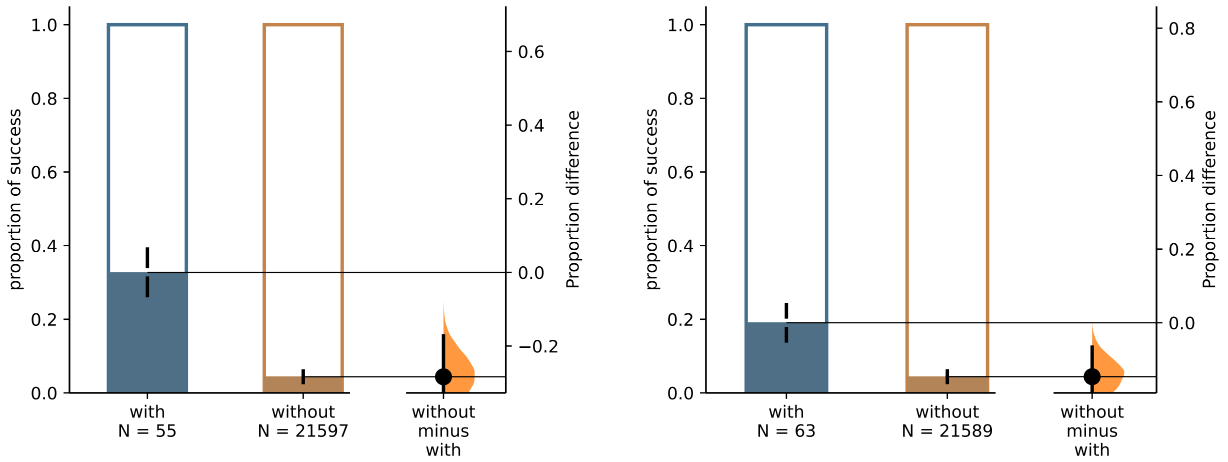

(a) (b)

Figure S77: Bootstrapped effect sizes with DABEST[S92] comparing subsets of structures with and without the presence of O1 w.r.t. OER with a. MOFTransformer and b. GPT-J predictions.

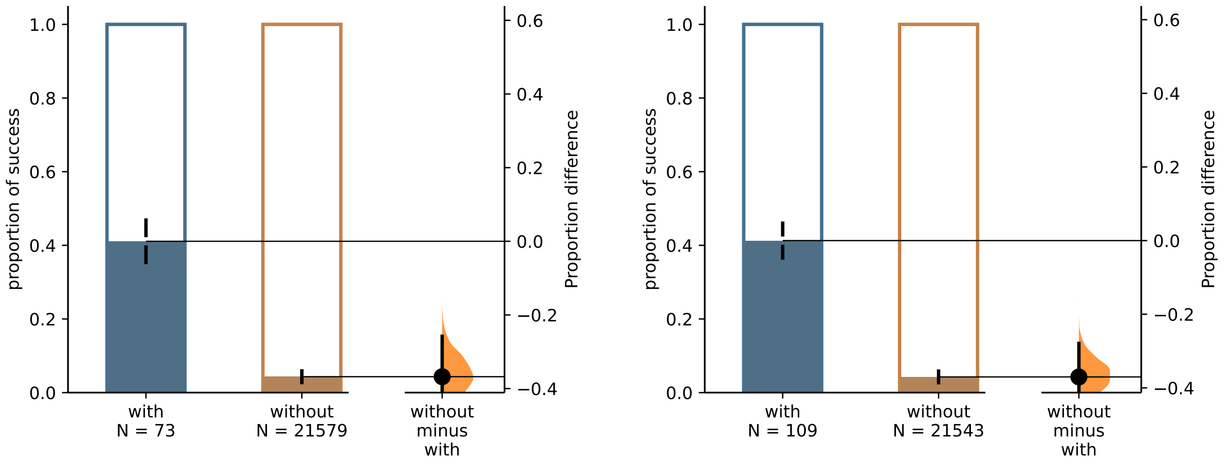

(a) (b)

Figure S78: Bootstrapped effect sizes with DABEST[S92] comparing subsets of structures with and without the presence of O3 w.r.t. OER with a. MOFTransformer and b. GPT-J predictions.

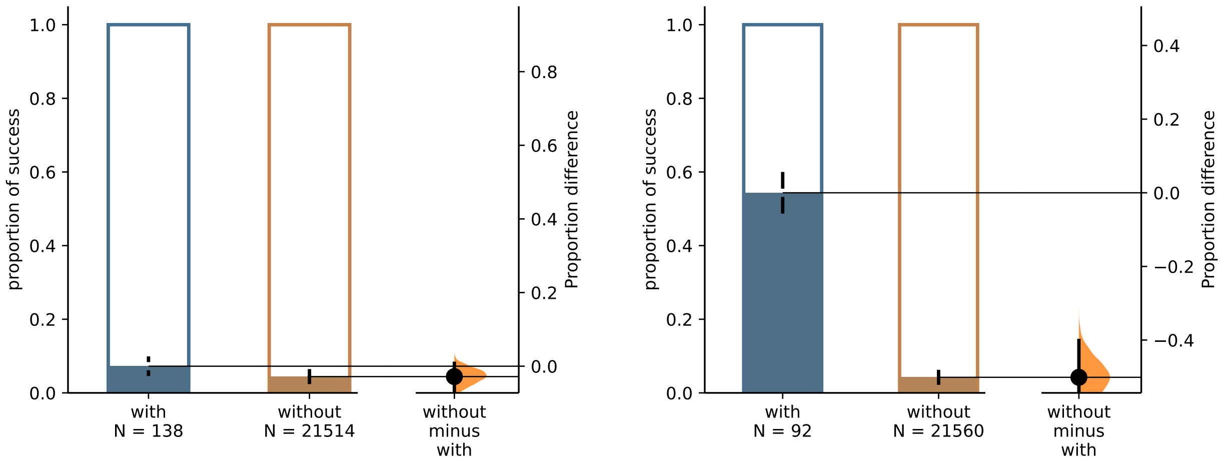

(a) (b)

Figure S79: Bootstrapped effect sizes with DABEST[S92] comparing subsets of structures with and without the presence of O14 w.r.t. OER with a. MOFTransformer and b. GPT-J predictions.

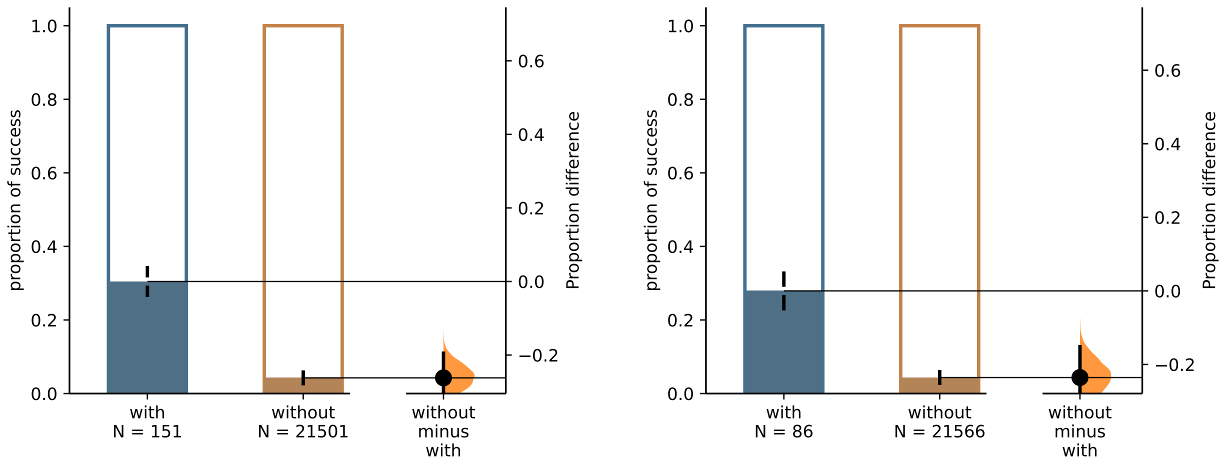

(a) (b)

Figure S80: Bootstrapped effect sizes with DABEST[S92] comparing subsets of structures with and without the presence of O15 w.r.t. OER with a. MOFTransformer and b. GPT-J predictions.

4.2.2 Metal nodes

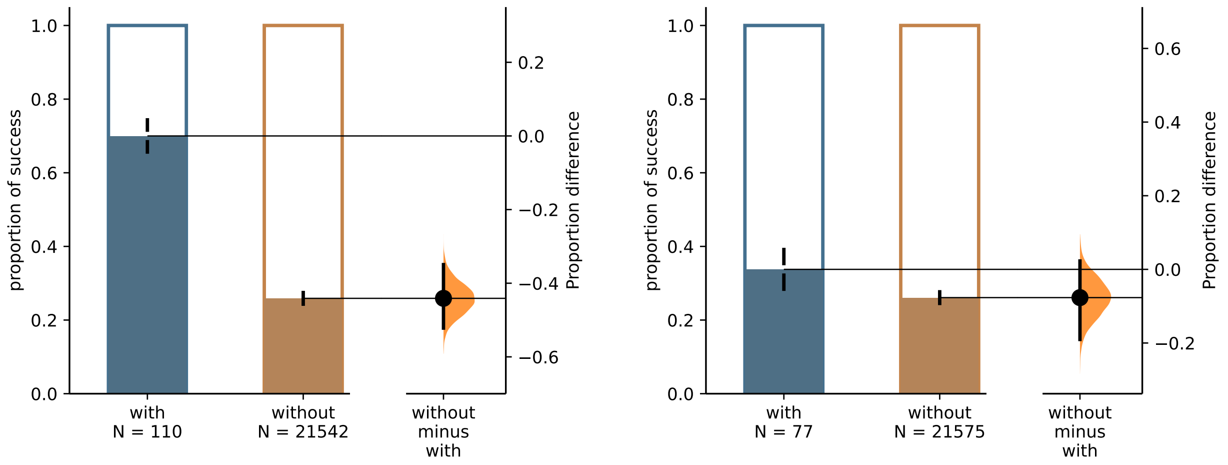

(a) (b)

Figure S81: Bootstrapped effect sizes with DABEST[S92] comparing subsets of structures with and without the presence of Cl[Zn]Cl w.r.t. m^{*}_{red} with a. MOFTransformer and b. GPT-J predictions.

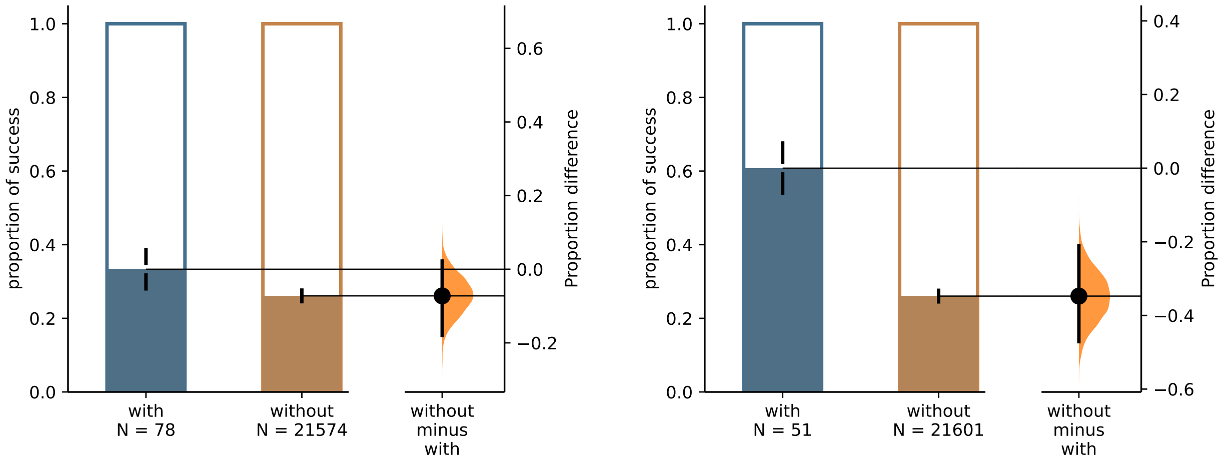

(a) (b)

Figure S82: Bootstrapped effect sizes with DABEST[S92] comparing subsets of structures with and without the presence of [Cs] w.r.t. m^*_{red} with a. MOFTransformer and b. GPT-J predictions.

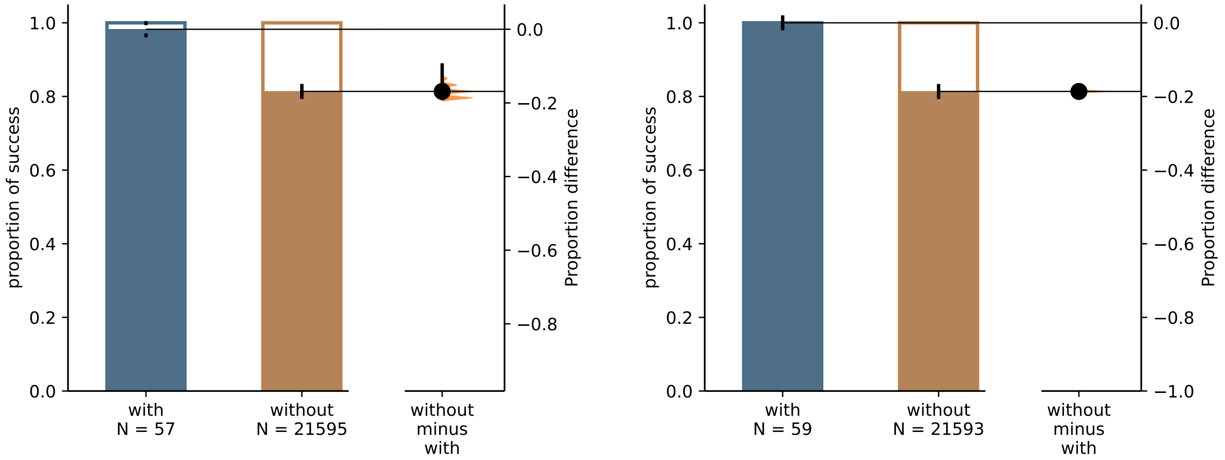

(a) (b)

Figure S83: Bootstrapped effect sizes with DABEST[S92] comparing subsets of structures with and without the presence of [OH2][Nd][OH2] w.r.t. Λ with a. MOFTransformer and b. GPT-J predictions.

4.2.3 MOFs that met all criteria

According to MOFTransformer predictions, MOFs that met all criteria (25) contained i) metal nodes with Co, Ni, Ti, TiZr, Al, and FeSe, and ii) organic linkers with thiophene

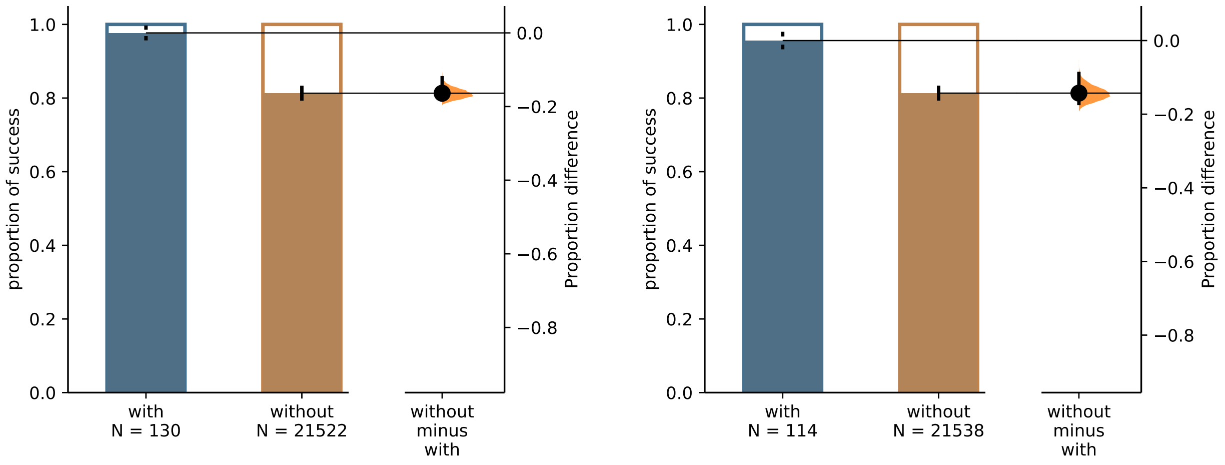

(a) (b)

Figure S84: Bootstrapped effect sizes with DABEST[S92] comparing subsets of structures with and without the presence of [OH2][Co][OH2] w.r.t. Λ with a. MOFTransformer and b. GPT-J predictions.

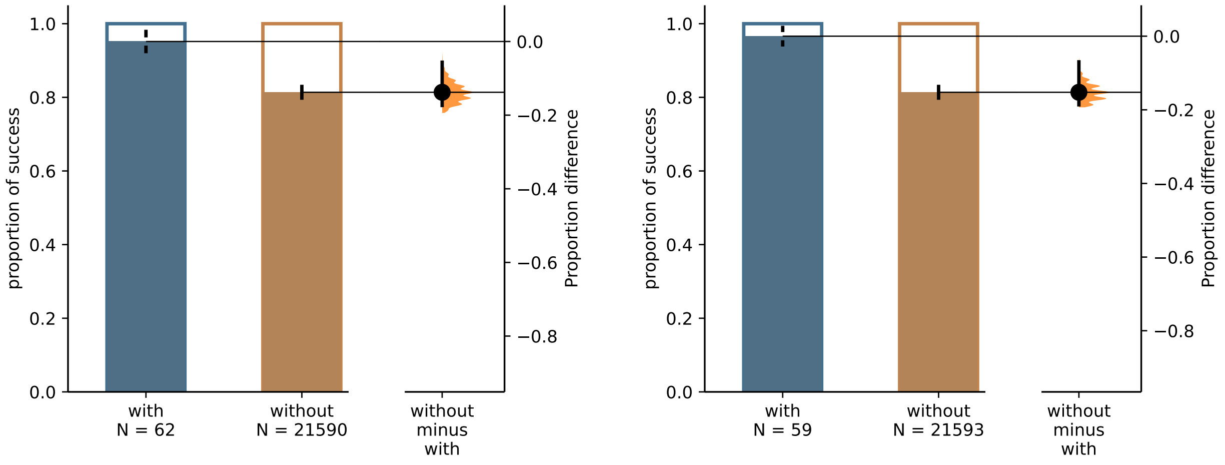

(a) (b)

Figure S85: Bootstrapped effect sizes with DABEST[S92] comparing subsets of structures with and without the presence of [Ba] w.r.t. Λ with a. MOFTransformer and b. GPT-J predictions.

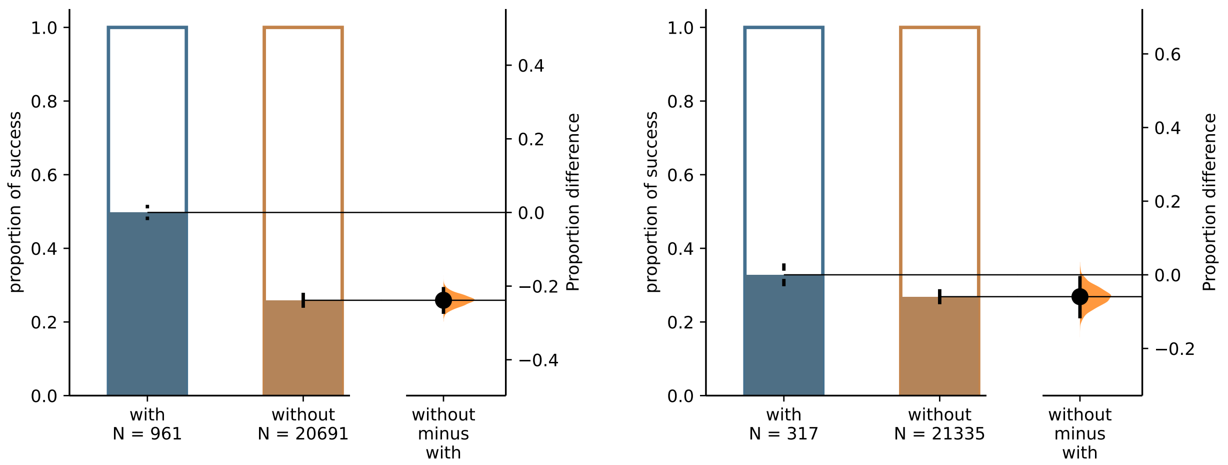

(a) (b)

Figure S86: Bootstrapped effect sizes with DABEST[S92] comparing subsets of structures with and without the presence of [OH2][Zn] w.r.t. HER with a. MOFTransformer and b. GPT-J predictions.

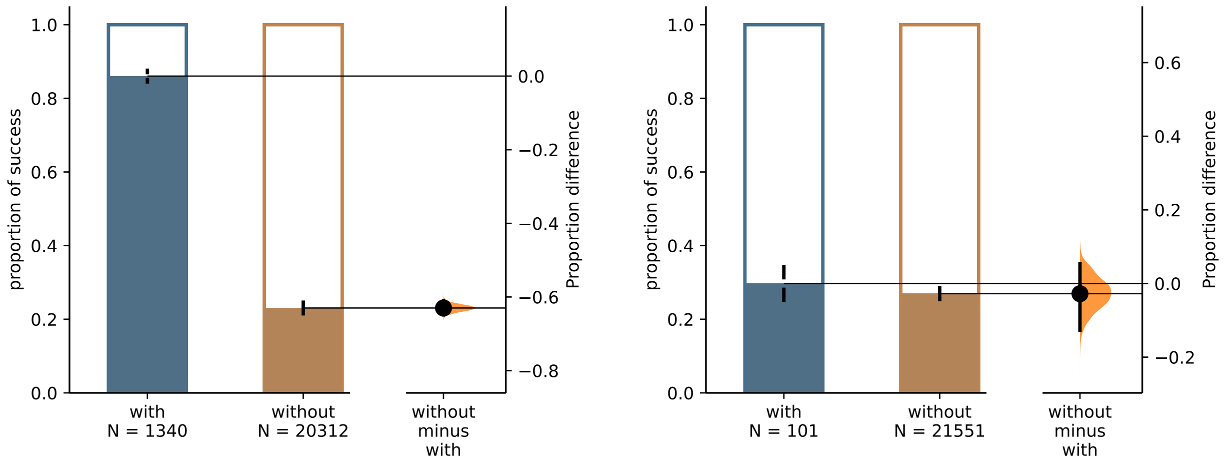

(a) (b)

Figure S87: Bootstrapped effect sizes with DABEST[S92] comparing subsets of structures with and without the presence of [OH2][Mn] w.r.t. HER with a. MOFTransformer and b. GPT-J predictions.

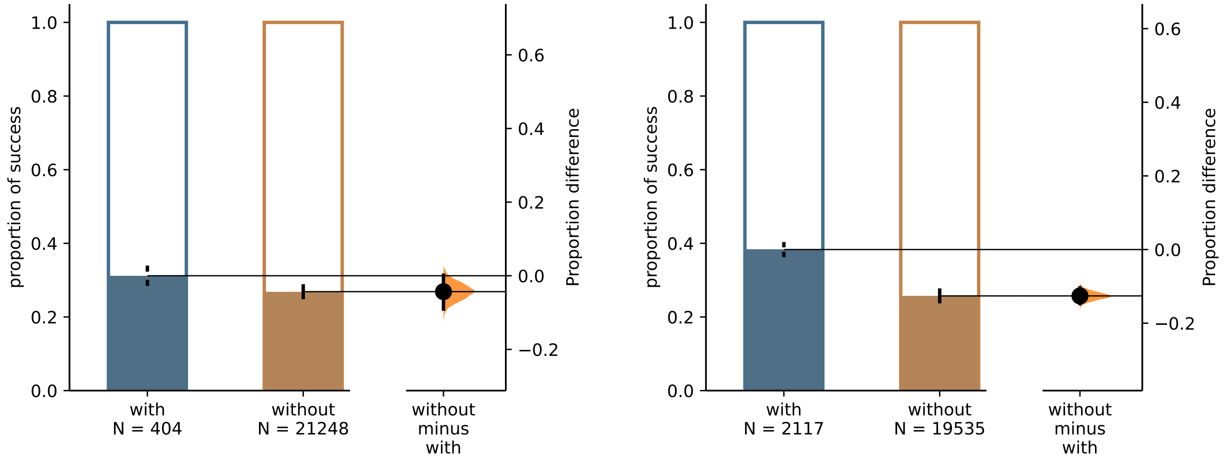

(a) (b)

Figure S88: Bootstrapped effect sizes with DABEST[S92] comparing subsets of structures with and without the presence of [Zn] w.r.t. HER with a. MOFTransformer and b. GPT-J predictions.

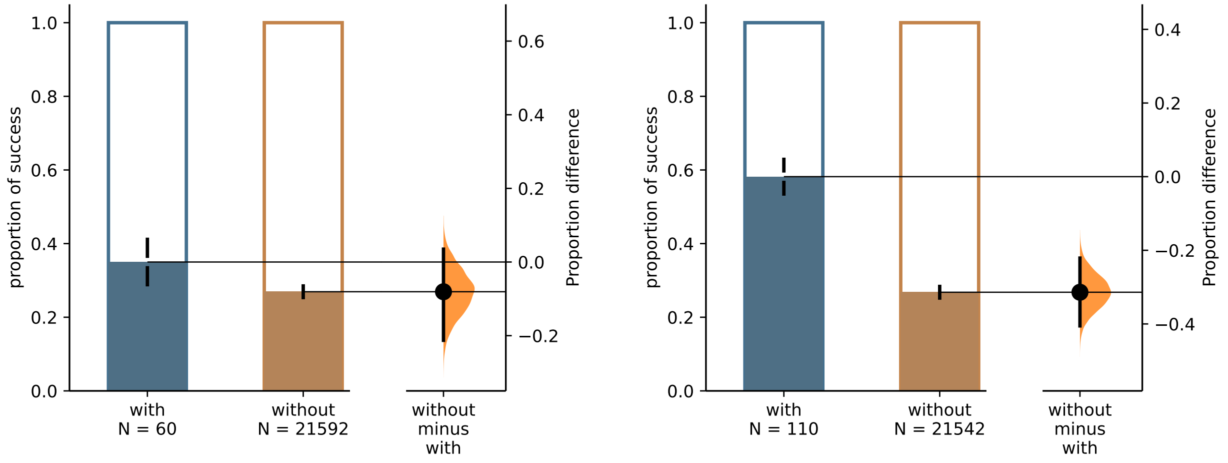

(a) (b)

Figure S89: Bootstrapped effect sizes with DABEST[S92] comparing subsets of structures with and without the presence of I[Cu][Cu]I w.r.t. HER with a. MOFTransformer and b. GPT-J predictions.

(more specifically, anthra-tetrathiophene), pyrazole, pyrene, and thiadiazole (4,6-dithiophen-2-ylthieno[3,4-c][1,2,5]thiadiazole). Considering GPT-J predictions, on the other hand, motifs that stood out among structures that met all criteria (75) were i) metal nodes with Zn, Al, Ga, Co, TiZr, Ti, V, and Ni, and ii) organic linkers with pyrene, cubane, thiophene, thiadiazole, pyridinyl and phenanthroline.

5 Packages

Packages used by this work include the cp2k-input- and cp2k-output-tools,[S93,S94] DABEST for statistical analysis, cube,[S45] pymatgen,[S95] spglib,[S96] NumPy,[S97] Pandas,[S98] SciPy,[S99] matplotlib,[S100] and bokeh.[S101]

References

(S1) Gibaldi, M.; Kwon, O.; White, A.; Burner, J.; Woo, T. K. The HEALED SBU Library of Chemically Realistic Building Blocks for Construction of Hypothetical Metal–Organic Frameworks. *ACS Applied Materials & Interfaces* **2022**, *14*, 43372–43386.

(S2) Colón, Y. J.; Gómez-Gualdrón, D. A.; Snurr, R. Q. Topologically Guided, Automated Construction of Metal-Organic Frameworks and Their Evaluation for Energy-Related Applications. *Crystal Growth & Design* **2017**, *17*, 5801–5810.

(S3) Jablonka, K. M.; Ongari, D.; Moosavi, S. M.; Smit, B. Using collective knowledge to assign oxidation states of metal cations in metal-organic frameworks. *Nature Chemistry* **2021**, *13*, 771–777.

(S4) O'keeffe, M.; Peskov, M. A.; Ramsden, S. J.; Yaghi, O. M. The Reticular Chemistry Structure Resource (RCSR) Database of, and Symbols for, Crystal Nets. *Accounts of Chemical Research* **2008**, *41*, 1782–1789.

(S5) Thompson, A. P.; Aktulga, H. M.; Berger, R.; Bolintineanu, D. S.; Brown, W. M.; Crozier, P. S.; in't Veld, P. J.; Kohlmeyer, A.; Moore, S. G.; Nguyen, T. D., et al. LAMMPS-a flexible simulation tool for particle-based materials modeling at the atomic, meso, and continuum scales. *Computer Physics Communications* **2022**, *271*, 108171.

(S6) LAMMPS. `https://github.com/lammps/lammps`, using January 7, 2022 release throughout this study.

(S7) Boyd, P. G.; Mohamad Moosavi, S.; Witman, M.; Smit, B. Force-Field Prediction of Materials Properties in Metal-Organic Frameworks. *Journal of Physical Chemistry Letters* **2017**, *8*, 357–363.

(S8) LAMMPS Interface. `https://github.com/peteboyd/lammps_interface`, using May 8, 2021 release throughout this study.

(S9) EQeq:Charge equilibration method for crystal structures. `https://github.com/danieleongari/EQeq`, using March 10, 2020 release throughout this study.

(S10) EQeq:Charge equilibration method for crystal structures. `https://github.com/danieleongari/EQeq`, using March 10, 2020 release throughout this study.

(S11) Batatia, I.; Benner, P.; Chiang, Y.; Elena, A. M.; Kovács, D. P.; Riebesell, J.; Advincula, X. R.; Asta, M.; Avaylon, M.; Baldwin, W. J., et al. A foundation model for atomistic materials chemistry. *arXiv preprint arXiv:2401.00096* **2023**,

(S12) learningmatter-mit/NeuralForceField. `https://github.com/learningmatter-mit/NeuralForceField`, Accessed: November 2024.

(S13) Chung, Y.; Haldoupis, E.; Bucior, B.; Haranczyk, M.; Lee, S.; Zhang, H.; Vogiatzis, K.; Milisavljevic, M.; Ling, S.; Camp, J.; Slater, B.; Siepmann, J.; Sholl, D.; Snurr, R. Advances, Updates, and Analytics for the Computation-Ready, Experimental Metal-Organic Framework Database: CoRE MOF 2019. *Journal of Chemical & Engineering Data* **2019**, *64*, 5985–5998.

(S14) Gómez-Gualdrón, D. A.; Colón, Y. J.; Zhang, X.; Wang, T. C.; Chen, Y.-S.; Hupp, J. T.; Yildirim, T.; Farha, O. K.; Zhang, J.; Snurr, R. Q. Evaluating Topologically Diverse Metal–Organic Frameworks for Cryo-Adsorbed Hydrogen Storage. *Energy & Environmental Science* **2016**, *9*, 3279–3289.

(S15) Anderson, R.; Gómez-Gualdrón, D. A. Increasing topological diversity during computational "synthesis" of porous crystals: how and why. *CrystEngComm* **2019**, *21*, 1653–1665.

(S16) Majumdar, S.; Moosavi, S. M.; Jablonka, K. M.; Ongari, D.; Smit, B. Diversifying Databases of Metal Organic Frameworks for High-Throughput Computational Screening. *ACS Applied Materials & Interfaces* **2021**, *13*, 61004–61014.

(S17) Burner, J.; Luo, J.; White, A.; Mirmiran, A.; Kwon, O.; Boyd, P. G.; Maley, S.; Gibaldi, M.; Simrod, S.; Ogden, V., et al. ARC–MOF: a diverse database of metal-organic frameworks with DFT-derived partial atomic charges and descriptors for machine learning. *Chemistry of Materials* **2023**, *35*, 900–916.

(S18) Boyd, P. G.; Woo, T. K. A Generalized Method for Constructing Hypothetical Hanoporous Materials of Any Net Topology from Graph Theory. *CrystEngComm* **2016**, *18*, 3777–3792.

(S19) Moosavi, S. M.; Nandy, A.; Jablonka, K. M.; Ongari, D.; Janet, J. P.; Boyd, P. G.; Lee, Y.; Smit, B.; Kulik, H. J. Understanding the diversity of the metal-organic framework ecosystem. *Nature Communications* **2020**, *11*.

(S20) Willems, T. F.; Rycroft, C. H.; Kazi, M.; Meza, J. C.; Haranczyk, M. Algorithms and Tools for High-Throughput Geometry-Based Analysis of Crystalline Porous Materials. *Microporous and Mesoporous Materials* **2012**, *149*, 134 – 141.

(S21) Janet, J. P.; Kulik, H. J. Resolving Transition Metal Chemical Space: Feature Selection for Machine Learning and Structure–Property Relationships. *The Journal of Physical Chemistry A* **2017**, *121*, 8939–8954.

(S22) Pielou, E. C. The Measurement of Diversity in Different Types of Biological Collections. *Journal of Theoretical Biology* **1966**, *13*, 131–144.

(S23) Kennard, R. W.; Stone, L. A. Computer aided design of experiments. *Technometrics* **1969**, *11*, 137–148.

(S24) Fumanal, M.; Ortega-Guerrero, A.; Jablonka, K. M.; Smit, B.; Tavernelli, I. Charge Separation and Charge Carrier Mobility in Photocatalytic Metal-Organic Frameworks. *Advanced Functional Materials* **2020**, *30*, 2003792.

(S25) Huber, S. P.; Zoupanos, S.; Uhrin, M.; Talirz, L.; Kahle, L.; Häuselmann, R.; Gresch, D.; Müller, T.; Yakutovich, A. V.; Andersen, C. W.; Ramirez, F. F.; Adorf, C. S.; Gargiulo, F.; Kumbhar, S.; Passaro, E.; Johnston, C.; Merkys, A.; Cepellotti, A.; Mounet, N.; Marzari, N.; Kozinsky, B.; Pizzi, G. AiiDA 1.0, a scalable computational infrastructure for automated reproducible workflows and data provenance. *Scientific Data* **2020**, *7*.

(S26) Uhrin, M.; Huber, S. P.; Yu, J.; Marzari, N.; Pizzi, G. Workflows in AiiDA: Engineering a high-throughput, event-based engine for robust and modular computational workflows. *Computational Materials Science* **2021**, *187*, 110086.

(S27) Kühne, T. D.; Iannuzzi, M.; Del Ben, M.; Rybkin, V. V.; Seewald, P.; Stein, F.; Laino, T.; Khaliullin, R. Z.; Schütt, O.; Schiffmann, F.; Golze, D.; Wilhelm, J.; Chulkov, S.; Bani-Hashemian, M. H.; Weber, V.; Borštnik, U.; Taillefumier, M.; Jakobovits, A. S.; Lazzaro, A.; Pabst, H.; Müller, T.; Schade, R.; Guidon, M.; Andermatt, S.; Holmberg, N.; Schenter, G. K.; Hehn, A.; Bussy, A.; Belleflamme, F.; Tabacchi, G.; Glöß, A.; Lass, M.; Bethune, I.; Mundy, C. J.; Plessl, C.; Watkins, M.; VandeVondele, J.; Krack, M.; Hutter, J. CP2K: An electronic structure and molecular dynamics software package - Quickstep: Efficient and accurate electronic structure calculations. *The Journal of Chemical Physics* **2020**, *152*, 194103.

(S28) Mourino, B.; Jablonka, K. M.; Ortega-Guerrero, A.; Smit, B. In Search of Covalent Organic Framework Photocatalysts: A DFT-Based Screening Approach. *Advanced Functional Materials* **2023**, *33*.

(S29) Fumanal, M.; Capano, G.; Barthel, S.; Smit, B.; Tavernelli, I. Energy-based descriptors

for photo-catalytically active metal-organic framework discovery. *Journal of Materials Chemistry A* **2020**, *8*, 4473–4482.

(S30) Jablonka, K. M.; Rosen, A. S.; Krishnapriyan, A. S.; Smit, B. An Ecosystem for Digital Reticular Chemistry. *ACS Central Science* **2023**, *9*, 563–581.

(S31) Giannozzi, P.; Baroni, S.; Bonini, N.; Calandra, M.; Car, R.; Cavazzoni, C.; Ceresoli, D.; Chiarotti, G. L.; Cococcioni, M.; Dabo, I.; Corso, A. D.; de Gironcoli, S.; Fabris, S.; Fratesi, G.; Gebauer, R.; Gerstmann, U.; Gougoussis, C.; Kokalj, A.; Lazzeri, M.; Martin-Samos, L.; Marzari, N.; Mauri, F.; Mazzarello, R.; Paolini, S.; Pasquarello, A.; Paulatto, L.; Sbraccia, C.; Scandolo, S.; Sclauzero, G.; Seitsonen, A. P.; Smogunov, A.; Umari, P.; Wentzcovitch, R. M. QUANTUM ESPRESSO: a modular and open-source software project for quantum simulations of materials. *Journal of Physics: Condensed Matter* **2009**, *21*, 395502.

(S32) Kshirsagar, A. R.; Blase, X.; Attaccalite, C.; Poloni, R. Strongly Bound Excitons in Metal–Organic Framework MOF-5: A Many-Body Perturbation Theory Study. *The Journal of Physical Chemistry Letters* **2021**, *12*, 4045–4051.

(S33) Rosen, A. S.; Fung, V.; Huck, P.; O'Donnell, C. T.; Horton, M. K.; Truhlar, D. G.; Persson, K. A.; Notestein, J. M.; Snurr, R. Q. High-throughput predictions of metal-organic framework electronic properties: theoretical challenges, graph neural networks, and data exploration. *npj Computational Materials* **2022**, *8*.

(S34) Guidon, M.; Hutter, J.; VandeVondele, J. Auxiliary Density Matrix Methods for Hartree-Fock Exchange Calculations. *Journal of Chemical Theory and Computation* **2010**, *6*, 2348–2364.

(S35) Hinuma, Y.; Pizzi, G.; Kumagai, Y.; Oba, F.; Tanaka, I. Band structure diagram paths based on crystallography. *Computational Materials Science* **2017**, *128*, 140–184.

(S36) Uhrin, M.; Zadoks, A.; Binci, L.; Marzari, N.; Timrov, I. Machine learning Hubbard parameters with equivariant neural networks. *npj Computational Materials* **2025**, *11*.

(S37) Timrov, I.; Kotiuga, M.; Marzari, N. Unraveling the effects of inter-site Hubbard interactions in spinel Li-ion cathode materials. *Physical Chemistry Chemical Physics* **2023**, *25*, 9061–9072.

(S38) Anisimov, V. I.; Zaanen, J.; Andersen, O. K. Band theory and Mott insulators: Hubbard U instead of Stoner I. *Physical Review B* **1991**, *44*, 943–954.

(S39) Dudarev, S. L.; Botton, G. A.; Savrasov, S. Y.; Humphreys, C. J.; Sutton, A. P. Electron-energy-loss spectra and the structural stability of nickel oxide: An LSDA+U study. *Physical Review B* **1998**, *57*, 1505–1509.

(S40) Marzari, N.; Ferretti, A.; Wolverton, C. Electronic-structure methods for materials design. *Nature Materials* **2021**, *20*, 736–749.

(S41) Mori-Sánchez, P.; Cohen, A. J. The derivative discontinuity of the exchange-correlation functional. *Physical Chemistry Chemical Physics* **2014**, *16*, 14378–14387.

(S42) Mann, G. W.; Lee, K.; Cococcioni, M.; Smit, B.; Neaton, J. B. First-principles Hubbard U approach for small molecule binding in metal-organic frameworks. *The Journal of Chemical Physics* **2016**, *144*, 174104.

(S43) Ortega-Guerrero, A.; Fumanal, M.; Capano, G.; Smit, B. From Isolated Porphyrin Ligands to Periodic Al-PMOF: A Comparative Study of the Optical Properties Using DFT/TDDFT. *The Journal of Physical Chemistry C* **2020**, *124*, 21751–21760.

(S44) Butler, K. T.; Hendon, C. H.; Walsh, A. Electronic Chemical Potentials of Porous Metal–Organic Frameworks. *Journal of the American Chemical Society* **2014**, *136*, 2703–2706, PMID: 24447027.

(S45) Jablonka, K. M. Cube analysis. https://github.com/kjappelbaum/cubes, Accessed: 2022-09-17.

(S46) Ganose, A. M.; Jackson, A. J.; Scanlon, D. O. sumo: Command-line tools for plotting and analysis of periodic *ab initio* calculations. *Journal of Open Source Software* **2018**, *3*, 717.

(S47) Muschielok, C.; Oberhofer, H. Aspects of semiconductivity in soft, porous metal-organic framework crystals. *The Journal of Chemical Physics* **2019**, *151*, 015102.

(S48) Yang, G.; Raptis, R. G. Oxidation of gold(i) pyrazolates by aqua regia. X-Ray crystal structures of the first examples of trinuclear AuIII3 and AuIAuIII2 pyrazolato complexes. *Journal of the Chemical Society, Dalton Transactions* **2002**, 3936–3938.

(S49) Smith, R. A.; Kulmaczewski, R.; Halcrow, M. A. Ligand-Directed Metalation of a Gold Pyrazolate Cluster. *Inorganic Chemistry* **2023**, *62*, 9300–9305.

(S50) Liu, G.-N.; Xu, R.-D.; Guo, J.-S.; Miao, J.-L.; Zhang, M.-J.; Li, C. Regulating the near-infrared region to visible-light emission by adjusting cuprophilic interactions for blue light-excited phosphors. *Journal of Materials Chemistry C* **2021**, *9*, 8589–8595.

(S51) Nguyen, H. L.; Gándara, F.; Furukawa, H.; Doan, T. L. H.; Cordova, K. E.; Yaghi, O. M. A Titanium-Organic Framework as an Exemplar of Combining the Chemistry of Metal- and Covalent-Organic Frameworks. *Journal of the American Chemical Society* **2016**, *138*, 4330–4333.

(S52) Yuan, S.; Qin, J.-S.; Xu, H.-Q.; Su, J.; Rossi, D.; Chen, Y.; Zhang, L.; Lollar, C.; Wang, Q.; Jiang, H.-L.; Son, D. H.; Xu, H.; Huang, Z.; Zou, X.; Zhou, H.-C. [$Ti_8Zr_2O_12(COO)_{16}$] Cluster: An Ideal Inorganic Building Unit for Photoactive Metal-Organic Frameworks. *ACS Central Science* **2017**, *4*, 105–111.

(S53) Keum, Y.; Park, S.; Chen, Y.-P.; Park, J. Titanium-Carboxylate Metal-Organic Framework Based on an Unprecedented Ti-Oxo Chain Cluster. *Angewandte Chemie International Edition* **2018**, *57*, 14852–14856.

(S54) Galli, S.; Masciocchi, N.; Colombo, V.; Maspero, A.; Palmisano, G.; López-Garzón, F. J.; Domingo-García, M.; Fernández-Morales, I.; Barea, E.; Navarro, J. A. R. Adsorption of Harmful Organic Vapors by Flexible Hydrophobic Bis-pyrazolate Based MOFs. *Chemistry of Materials* **2010**, *22*, 1664–1672.

(S55) Yan, Z.; Li, M.; Gao, H.-L.; Huang, X.-C.; Li, D. High-spin versus spin-crossover versus low-spin: geometry intervention in cooperativity in a 3D polymorphic iron(ii)-tetrazole MOFs system. *Chemical Communications* **2012**, *48*, 3960.

(S56) Diamantis, A. A.; Snow, M. R.; Vanzo, J. A. A trigonal prismatic vanadium(IV) complex: bis(acetylacetone benzoylhydrazonato)vanadium(IV), X-ray crystal structure. *Journal of the Chemical Society, Chemical Communications* **1976**, 264.

(S57) Auerbach, U.; Vedova, B. S. P. C. D.; Wieghardt, K.; Nuber, B.; Weiss, J. Syntheses and characterization of stable pseudo-octahedral tris-phenolato complexes of vanadium-(III), -(IV), and -(V). *Journal of the Chemical Society, Chemical Communications* **1990**, 1004.

(S58) Zhang, C.; Chen, C.; Dong, H.; Shen, J.-R.; Dau, H.; Zhao, J. A synthetic Mn_4Ca-cluster mimicking the oxygen-evolving center of photosynthesis. *Science* **2015**, *348*, 690–693.

(S59) Chen, C.; Chen, Y.; Yao, R.; Li, Y.; Zhang, C. Artificial Mn_4Ca Clusters with Exchangeable Solvent Molecules Mimicking the Oxygen-Evolving Center in Photosynthesis. *Angewandte Chemie International Edition* **2019**, *58*, 3939–3942.

(S60) Kusunoki, M. S1-state Mn4Ca complex of Photosystem II exists in equilibrium

between the two most-stable isomeric substates: XRD and EXAFS evidence. *Journal of Photochemistry and Photobiology B: Biology* **2011**, *104*, 100–110.

(S61) Yano, J.; Robblee, J.; Pushkar, Y.; Marcus, M. A.; Bendix, J.; Workman, J. M.; Collins, T. J.; Solomon, E. I.; George, S. D.; Yachandra, V. K. Polarized X-ray Absorption Spectroscopy of Single-Crystal Mn(V) Complexes Relevant to the Oxygen-Evolving Complex of Photosystem II. *Journal of the American Chemical Society* **2007**, *129*, 12989–13000.

(S62) Yano, J.; Kern, J.; Irrgang, K.-D.; Latimer, M. J.; Bergmann, U.; Glatzel, P.; Pushkar, Y.; Biesiadka, J.; Loll, B.; Sauer, K.; Messinger, J.; Zouni, A.; Yachandra, V. K. X-ray damage to the Mn_4Ca complex in single crystals of photosystem II: A case study for metalloprotein crystallography. *Proceedings of the National Academy of Sciences* **2005**, *102*, 12047–12052.

(S63) Yang, Q.; Li, Y.; Yang, J.-D.; Liu, Y.; Zhang, L.; Luo, S.; Cheng, J.-P. Holistic Prediction of the pKa in Diverse Solvents Based on a Machine-Learning Approach. *Angewandte Chemie* **2020**, *132*, 19444–19453.

(S64) DeepSynthesis. `http://pka.luoszgroup.com//`, last accessed 2023-09-29.

(S65) Zhao, Y.; Xu, X.; Qiu, L.; Kang, X.; Wen, L.; Zhang, B. Metal–organic frameworks constructed from a new thiophene-functionalized dicarboxylate: luminescence sensing and pesticide removal. *ACS Applied Materials & Interfaces* **2017**, *9*, 15164–15175.

(S66) Mostakim, S.; Biswas, S. A thiadiazole-functionalized Zr (IV)-based metal–organic framework as a highly fluorescent probe for the selective detection of picric acid. *CrystEngComm* **2016**, *18*, 3104–3113.

(S67) Jin, J.-K.; Wu, K.; Liu, X.-Y.; Huang, G.-Q.; Huang, Y.-L.; Luo, D.; Xie, M.; Zhao, Y.; Lu, W.; Zhou, X.-P., et al. Building a pyrazole–benzothiadiazole–pyrazole

photosensitizer into metal–organic frameworks for photocatalytic aerobic oxidation. *Journal of the American Chemical Society* **2021**, *143*, 21340–21349.

(S68) Huang, J.; He, Y.; Yao, M.-S.; He, J.; Xu, G.; Zeller, M.; Xu, Z. A semiconducting gyroidal metal-sulfur framework for chemiresistive sensing. *Journal of Materials Chemistry A* **2017**, *5*, 16139–16143.

(S69) Li, X.; Anderson, R.; Fry, H. C.; Pratik, S. M.; Xu, W.; Goswami, S.; Allen, T. G.; Yu, J.; Rajasree, S. S.; Cramer, C. J., et al. Metal–Carbodithioate-Based 3D Semiconducting Metal–Organic Framework: Porous Optoelectronic Material for Energy Conversion. *ACS Applied Materials & Interfaces* **2023**,

(S70) Jablonka, K. M.; Schwaller, P.; Ortega-Guerrero, A.; Smit, B. Leveraging large language models for predictive chemistry. *Nature Machine Intelligence* **2024**, 1–9.

(S71) Kang, Y.; Park, H.; Smit, B.; Kim, J. A multi-modal pre-training transformer for universal transfer learning in metal–organic frameworks. *Nature Machine Intelligence* **2023**, 1–10.

(S72) Wang, B.; Komatsuzaki, A. GPT-J-6B: A 6 Billion Parameter Autoregressive Language Model. `https://github.com/kingoflolz/mesh-transformer-jax`, 2021.

(S73) Gao, L.; Biderman, S.; Black, S.; Golding, L.; Hoppe, T.; Foster, C.; Phang, J.; He, H.; Thite, A.; Nabeshima, N.; Presser, S.; Leahy, C. The Pile: An 800GB Dataset of Diverse Text for Language Modeling. *arXiv preprint Arxiv-2101.00027* **2020**,

(S74) Van Herck, J.; Gil, M. V.; Jablonka, K. M.; Abrudan, A.; Anker, A. S.; Asgari, M.; Blaiszik, B. J.; Buffo, A.; Choudhury, L.; Corminboeuf, C., et al. Assessment of Fine-Tuned Large Language Models for Real-World Chemistry and Material Science Applications. *Chemical Science* **2024**,

(S75) Dettmers, T.; Lewis, M.; Belkada, Y.; Zettlemoyer, L. GPT3.int8(): 8-bit Matrix Multiplication for Transformers at Scale. Advances in Neural Information Processing Systems. 2022.

(S76) Dettmers, T.; Lewis, M.; Shleifer, S.; Zettlemoyer, L. 8-bit Optimizers via Block-wise Quantization. The Tenth International Conference on Learning Representations, ICLR. 2022.

(S77) Hu, E. J.; Shen, Y.; Wallis, P.; Allen-Zhu, Z.; Li, Y.; Wang, S.; Chen, W. LoRA: Low-Rank Adaptation of Large Language Models. International Conference On Learning Representations. 2021.

(S78) Li, L.; Wang, X.-S.; Liu, T.-F.; Ye, J. Titanium-Based MOF Materials: From Crystal Engineering to Photocatalysis. *Small Methods* **2020**, *4*, 2000486.

(S79) Kolobov, N.; Goesten, M. G.; Gascon, J. Metal-Organic Frameworks: Molecules or Semiconductors in Photocatalysis? *Angewandte Chemie International Edition* **2021**, *60*, 26038–26052.

(S80) Earl, L. D.; Nagle, J. K.; Wolf, M. O. Tuning the Extended Structure and Electronic Properties of Gold(I) Thienyl Pyrazolates. *Inorganic Chemistry* **2014**, *53*, 7106–7117.

(S81) Xia, S.; Xie, J. Energy transfer in gold photocatalysis. *Gold Bulletin* **2022**, *55*, 123–127.

(S82) Zhu, X.; Miao, H.; Shan, Y.; Gao, G.; Gu, Q.; Xiao, Q.; He, X. Two-Dimensional Janus Film with Au Nanoparticles Assembled on Trinuclear Gold(I) Pyrazolate Coordination Nanosheets for Photocatalytic H_2 Evolution. *Inorganic Chemistry* **2022**, *61*, 13591–13599.

(S83) Tanaka, A.; Ogino, A.; Iwaki, M.; Hashimoto, K.; Ohnuma, A.; Amano, F.; Ohtani, B.; Kominami, H. Gold-Titanium(IV) Oxide Plasmonic Photocatalysts Prepared by

a Colloid-Photodeposition Method: Correlation Between Physical Properties and Photocatalytic Activities. *Langmuir* **2012**, *28*, 13105–13111.

(S84) Chen, L.-W.; Hao, Y.-C.; Guo, Y.; Zhang, Q.; Li, J.; Gao, W.-Y.; Ren, L.; Su, X.; Hu, L.; Zhang, N.; Li, S.; Feng, X.; Gu, L.; Zhang, Y.-W.; Yin, A.-X.; Wang, B. Metal-Organic Framework Membranes Encapsulating Gold Nanoparticles for Direct Plasmonic Photocatalytic Nitrogen Fixation. *Journal of the American Chemical Society* **2021**, *143*, 5727–5736.

(S85) Kong, Y.; Xiong, D.; Lu, C.; Wang, J.; Liu, T.; Ying, S.; Ma, X.; Yi, F.-Y. Vanadium-Based Trimetallic Metal-Organic-Framework Family as Extremely High-Performing and Ultrastable Electrocatalysts for Water Splitting. *ACS Applied Materials & Interfaces* **2022**, *14*, 37804–37813.

(S86) Guo, K.; Jie, G.; Liu, J.; Fu, Y.; Ma, R.; Lu, X.; Zhang, F.; Zhu, W.; Fan, M. Visible-light-driven photocatalytic selective oxidation of amines and sulfides over a vanadium metal-organic framework. *Sustainable Energy & Fuels* **2022**, *6*, 5261–5267.

(S87) Nguyen, M. V.; Dong, H. C.; Truong, V. T. N.; Nguyen, H. N.; Luu, L. C.; Dang, N. N.; Nguyen, T. A. T. A new porphyrinic vanadium-based MOF constructed from infinite $V(OH_4)O$ chains: syntheses, characterization and photoabsorption properties. *New Journal of Chemistry* **2022**, *46*, 632–641.

(S88) Lopez-Magano, A.; Jimenez-Almarza, A.; Aleman, J.; Mas-Balleste, R. Metal–organic frameworks (MOFs) and covalent organic frameworks (COFs) applied to photocatalytic organic transformations. *Catalysts* **2020**, *10*, 720.

(S89) Narayan, T. C.; Miyakai, T.; Seki, S.; Dincă, M. High Charge Mobility in a Tetrathiafulvalene-Based Microporous Metal-Organic Framework. *Journal of the American Chemical Society* **2012**, *134*, 12932–12935.

(S90) Sun, L.; Miyakai, T.; Seki, S.; Dincă, M. Mn_2(2, 5-disulfhydrylbenzene-1, 4-dicarboxylate): A Microporous Metal-Organic Framework with Infinite (-Mn-S-) Chains and High Intrinsic Charge Mobility. *Journal of the American Chemical Society* **2013**, *135*, 8185–8188.

(S91) Huang, Q.-Q.; Fang, Z.-B.; Pang, K.; Qin, W.-K.; Liu, T.-F.; Cao, R. The Impact of Secondary Building Units in Metal-Organic Frameworks on Plasmonic Gold-Sensitized Photocatalysis. *Advanced Functional Materials* **2022**, *32*, 2205147.

(S92) Ho, J.; Tumkaya, T.; Aryal, S.; Choi, H.; Claridge-Chang, A. Moving beyond P values: data analysis with estimation graphics. *Nature Methods* **2019**, *16*, 565–566.

(S93) Cp2k input tools. `https://github.com/cp2k/cp2k-input-tools`, Accessed: 2022-12-07.

(S94) Cp2k output tools. `https://github.com/cp2k/cp2k-output-tools`, Accessed: 2022-12-07.

(S95) Ong, S. P.; Richards, W. D.; Jain, A.; Hautier, G.; Kocher, M.; Cholia, S.; Gunter, D.; Chevrier, V. L.; Persson, K. A.; Ceder, G. Python Materials Genomics (pymatgen): A robust, open-source python library for materials analysis. *Computational Materials Science* **2013**, *68*, 314–319.

(S96) Togo, A.; Tanaka, I. `Spglib`: a software library for crystal symmetry search. 2018; `https://arxiv.org/abs/1808.01590`.

(S97) Harris, C. R.; Millman, K. J.; van der Walt, S. J.; Gommers, R.; Virtanen, P.; Cournapeau, D.; Wieser, E.; Taylor, J.; Berg, S.; Smith, N. J.; Kern, R.; Picus, M.; Hoyer, S.; van Kerkwijk, M. H.; Brett, M.; Haldane, A.; del Río, J. F.; Wiebe, M.; Peterson, P.; Gérard-Marchant, P.; Sheppard, K.; Reddy, T.; Weckesser, W.; Abbasi, H.; Gohlke, C.; Oliphant, T. E. Array programming with NumPy. *Nature* **2020**, *585*, 357–362.

(S98) Wes McKinney, Data Structures for Statistical Computing in Python. Proceedings of the 9th Python in Science Conference. 2010; pp 56 – 61.

(S99) Virtanen, P.; Gommers, R.; Oliphant, T. E.; Haberland, M.; Reddy, T.; Cournapeau, D.; Burovski, E.; Peterson, P.; Weckesser, W.; Bright, J.; van der Walt, S. J.; Brett, M.; Wilson, J.; Millman, K. J.; Mayorov, N.; Nelson, A. R. J.; Jones, E.; Kern, R.; Larson, E.; Carey, C. J.; Polat, İ.; Feng, Y.; Moore, E. W.; VanderPlas, J.; Laxalde, D.; Perktold, J.; Cimrman, R.; Henriksen, I.; Quintero, E. A.; Harris, C. R.; Archibald, A. M.; Ribeiro, A. H.; Pedregosa, F.; van Mulbregt, P.; SciPy 1.0 Contributors, SciPy 1.0: Fundamental Algorithms for Scientific Computing in Python. *Nature Methods* **2020**, *17*, 261–272.

(S100) Hunter, J. D. Matplotlib: A 2D graphics environment. *Computing in Science & Engineering* **2007**, *9*, 90–95.

(S101) Bokeh Development Team, Bokeh: Python library for interactive visualization. 2018.
